# Interpretable Machine Learning of Hydrogen Production from Reactive Molecular Dynamics Simulations of Polyethylene Oxidative Gasification

**DOI:** 10.3390/ijms27219435

**Published:** 2026-10-22

**Authors:** Thi Be Ta Truong, Thuat T. Trinh

**Affiliations:** 1 Group of Applied Research in Advanced Materials for Sustainable Development, Faculty of Applied Sciences, Ton Duc Thang University, Ho Chi Minh City 756636, Vietnam; truongthibeta@tdtu.edu.vn; 2 Porelab, Department of Chemistry and Biomedical Science, Norwegian University of Science and Technology, NTNU, NO-7491 Trondheim, Norway

**Keywords:** polyethylene, oxidative gasification, ReaxFF, molecular dynamics, machine learning, hydrogen production, time-series forecasting, SHAP

## Abstract

Reactive molecular dynamics (ReaxFF MD) simulations of polyethylene (PE) oxidative gasification generate large trajectory datasets suited to machine learning (ML). We recast H_2_ production monitoring as a leakage-free forecasting task: given the simulated thermochemical state at time *t*, predict the time-weighted cumulative H_2_ production cumH2(t+h) a fixed horizon h=25 ps ahead. Data from 48 trajectories spanning six O_2_ loadings (1–900 molecules per 50 PE chains) and eight temperatures (1500–2900 K) yield 72,117 forecast pairs (45 simulations; three truncated trajectories excluded). Eight tabular regression models are benchmarked against persistence and physics-informed baselines under simulation-grouped nested cross-validation, a held-out test partition, temperature- and loading-holdout stress tests, and a paired ablation removing all H_2_-derived inputs. With the H_2_ history available, the best test model (random forest) attains $R^{2}=0.9999$ at RMSE 121.8 molecules·ps, a 93.0% RMSE reduction relative to persistence, robust to unseen O_2_ loadings and largely robust to unseen temperatures. Without it, no model beats persistence in any evaluation: the best ML model (an artificial neural network) is 26.0% worse, and accuracy decreases substantially under temperature extrapolation. The ablation attributes 93.3–98.3% of the tree and kernel models’ RMSE reduction to the H_2_-derived features, quantifying how much forecasting skill derives from the target history rather than instantaneous observables.

## 1. Introduction

The global energy demand and the mounting accumulation of plastic waste have emerged as two of the most pressing environmental and sustainability challenges of the 21st century [1,2]. Hydrogen (H_2_) is widely regarded as a clean energy carrier whose combustion produces only water, making it an attractive candidate for decarbonising the energy sector [3,4]. Concurrently, the world produces over 380 million tonnes of plastic per year, of which polyethylene (PE) accounts for the largest share [5]. Diverting plastic waste into hydrogen-rich gas products offers a dual benefit: it simultaneously addresses waste management and clean fuel production [6,7].

Thermochemical conversion offers several routes for transforming PE into H_2_-rich syngas. In thermal pyrolysis, the polymer backbone degrades under heat alone, whereas oxidative conversion additionally supplies oxygen: the thermo-oxidative degradation of PE follows well-characterised radical pathways whose rates depend strongly on oxygen availability [8], and controlled oxidative pyrolysis has been shown, by combined experiment and ReaxFF MD simulation, to lower the onset temperature of PE decomposition by up to 120 °C compared with inert pyrolysis [9]. Steam and supercritical-water gasification constitute a third route, in which water acts as both reactant and reaction medium and promotes steam reforming and water–gas shift reactions that favour H_2_ production [10,11,12,13]. These routes can be viewed as different oxidising atmospheres applied to the same carbonaceous feedstock, and molecular-scale insight into how the atmosphere and the temperature shape the H_2_ yield is essential for optimising process conditions and catalyst design [14,15,16]. In this work, PE–O_2_ mixtures are adopted as a well-defined model system for the oxidative branch of this family of processes: the O_2_ loading can be varied from trace to excess oxidant while the polymer feedstock and the temperature programme are held fixed, which isolates the effect of the oxidising atmosphere on H_2_ production.

Reactive molecular dynamics (ReaxFF MD) simulations [17] provide a computational tool for investigating chemical reactions at the atomistic level without predefining reaction pathways. Unlike classical force fields, ReaxFF dynamically updates bond orders during the simulation, enabling the description of bond breaking and formation events [18,19]. This capability makes ReaxFF particularly well suited for studying complex reactive systems such as hydrocarbon pyrolysis, oxidation, and gasification [20,21], including polyethylene and polystyrene gasification [22,23,24] and hydrogen dissociation thermodynamics [25].

Although ReaxFF MD yields detailed trajectory data, including time-resolved molecular counts, energy components, and bond information, the sheer volume of output (millions of timesteps across dozens of simulations) makes manual analysis intractable. Machine learning (ML) methods offer a data-driven approach to extracting predictive relationships from such high-dimensional simulation datasets [26]. One strand of research replaces the force field itself, learning interatomic potentials from quantum-mechanical data [27,28,29,30]; benchmark comparisons for metallic systems indicate that neural-network potentials can match or exceed ReaxFF accuracy at a substantially higher computational cost [30]. A second strand keeps the physics-based simulation unchanged and learns surrogate models that map simulation outputs to target quantities, as demonstrated for carbon-nanotube interface strength [31] and, more broadly, for surrogate-assisted computational science and engineering [26]. A third strand applies regression models directly to gasification data, predicting H_2_ yields from operating conditions and feedstock properties in biomass, sludge, and plastic co-gasification processes [32,33,34]. Interpretable ML approaches based on SHAP feature attribution add post hoc transparency and have been applied to chemistry and materials problems, including electron iso-density surfaces, thermoelectric properties, and anammox reactions [35,36,37].

Applying the surrogate strand to reactive trajectories requires particular care with validation. Trajectory data are strongly autocorrelated in time, so a random row-level train–test split places nearly identical snapshots on both sides of the split and yields optimistic error estimates; block cross-validation that respects such dependence structures is recommended wherever they exist [38]. Target-derived information that should not be legitimately available at prediction time can otherwise leak into the model [39], and model selection or hyperparameter tuning performed on the evaluation data itself introduces a selection bias that overstates generalisation performance [40]. These concerns coincide with the classical setting of multi-step time-series forecasting, whose strategies are defined by how they respect the temporal ordering of the data [41]. The present study addresses this methodological gap. Rather than proposing a new regression algorithm, it contributes (i) a leakage-free validation protocol for tabular surrogate models of reactive MD trajectories, in which the learning task is framed as forecasting the time-weighted cumulative H_2_ production a fixed horizon ahead from the instantaneous simulation state, all data manipulations respect the simulation-group and temporal structure, and generalisation is additionally stressed against unseen temperatures and unseen O_2_ loadings; and (ii) a within-model ablation (Case A vs. Case B) that isolates the predictive contribution of the target-history features from that of instantaneous physical observables.

In this study, we perform ReaxFF MD simulations of PE oxidative gasification across a designed matrix of thermodynamic conditions: six O_2_ loadings (*n*(O_2_) = 1–900 molecules per 50 PE chains, corresponding to O_2_:PE mass ratios from 0.15% to 136.2%) at eight temperatures between 1500 and 2900 K, yielding 48 production trajectories, 45 of which completed the full 500 ps and are used for machine learning. From the post-processed trajectories, we construct forecast-pair datasets and train a suite of eight tabular regression models under two feature scenarios. Case A admits target history, in the form of features derived from the H_2_ record up to the forecast origin, while Case B excludes all H_2_-derived features and retains only instantaneous descriptors. The models are benchmarked against persistence and physics-informed baselines under grouped, nested cross-validation with a held-out test partition, and their sensitivity to unseen conditions is probed with leave-one-temperature-out and leave-one-O_2_-loading-out protocols. We find that the two feature scenarios separate sharply: with the H_2_ history among the inputs, the best held-out test model (random forest) attains $R^{2}=0.9999$ at an RMSE of 121.8 molecules·ps, a 93.0% RMSE reduction relative to the persistence forecast, and the advantage is robust to unseen O_2_ loadings and largely robust to unseen temperatures. Without the H_2_-derived features, no machine-learning model improves on the persistence forecast in cross-validation, on the held-out test partition, or in any structured holdout, and predictive accuracy decreases substantially under temperature extrapolation. A paired Case A/Case B ablation attributes 93.3–98.3% of the RMSE reduction achieved by the tree and kernel models to the H_2_-derived features, and SHAP attributions show that a single autoregressive feature carries almost all of the Case A signal.

## 2. Results and Discussion

### 2.1. H_2_ Production Across the Simulated Conditions

**Figure 1:**
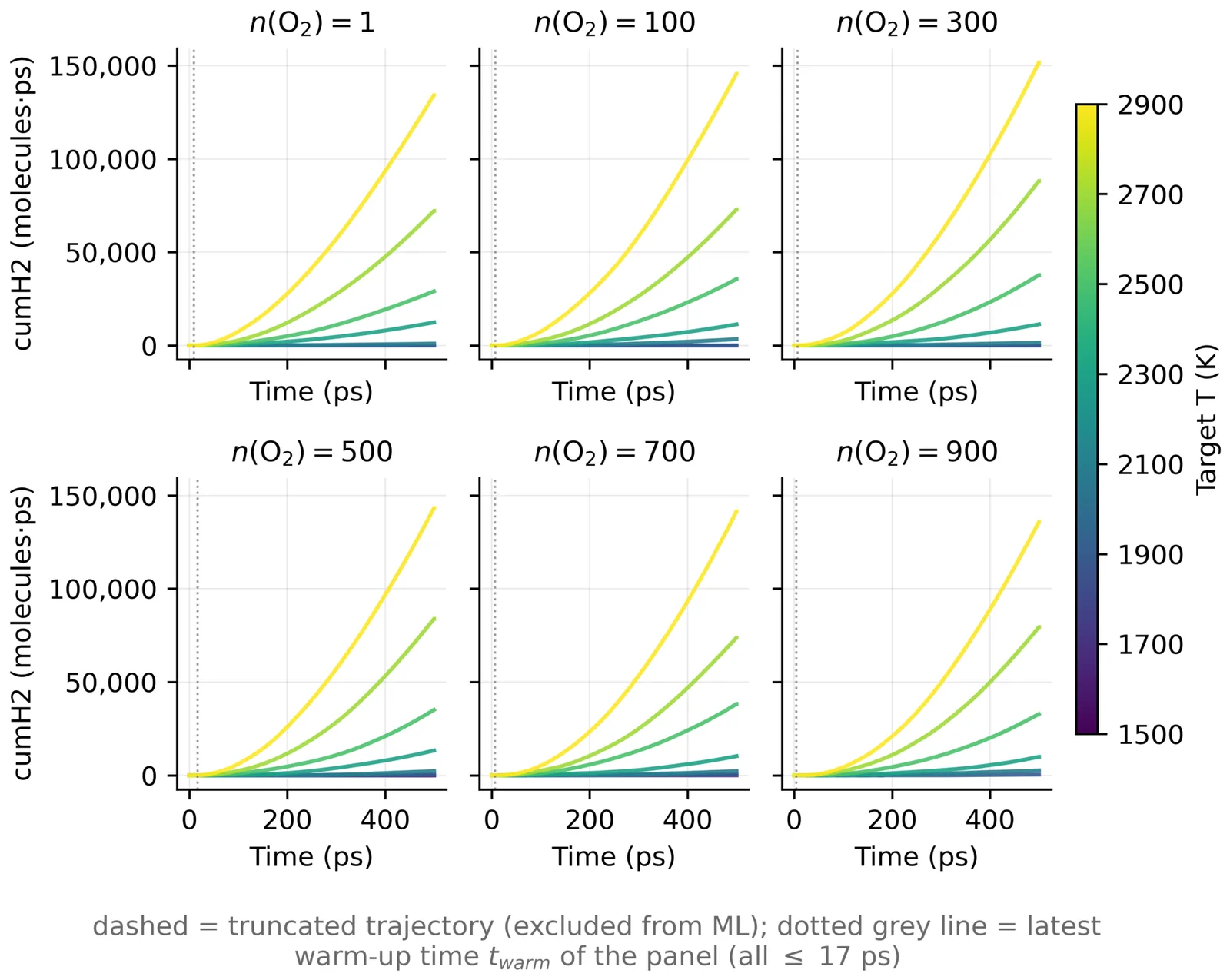
Cumulative H_2_ production from ReaxFF MD of polyethylene oxidative gasification: one panel per O_2_ loading (*n*(O_2_) = 1, 100, 300, 500, 700, and 900 molecules per 50 PE chains), eight temperature curves (1500–2900 K) per panel over 0–500 ps. Dashed curves are the three truncated runs excluded from machine learning, leaving 45 analysed simulations.

Figure 1 shows the time evolution of cumulative H_2_ production in the 45 analysed simulations, which are grouped by O_2_ loading. Production is strongly temperature-controlled. Ten simulations (four at 1500 K, five at 1700 K, and the lowest-loaded run at 1900 K) produce no H_2_ at all within the 500 ps window, so the effective onset of measurable H_2_ lies near 1900 K for this system. Above the onset, the final yield grows steeply with temperature: the largest final cumulative production attained within each temperature level rises from 524 molecules·ps at 1900 K to 3442 at 2100 K, 13,277 at 2300 K, 38,255 at 2500 K, 88,189 at 2700 K, and 151,612 molecules·ps at 2900 K, which is the maximum of the entire dataset and is reached by the *n*(O_2_) = 300 system. In molecule terms, that trajectory ends with 518 H_2_ molecules present, which is 33.4% of the stoichiometric maximum of 1550 H_2_ molecules (31 per C_30_H_62_ chain, 50 chains); terminal populations across the dataset range from 0 to 524 H_2_ molecules. Eleven simulations reach a plateau, which is defined as a production rate over the final 100 ps below 10% of the maximum rate within the trajectory; these are the ten zero-H_2_ simulations together with the $n(O_{2})=300$ simulation at 1900 K, which stalls after an early burst. The O_2_ loading modulates the production rate mainly at intermediate temperatures, whereas at the highest temperatures, all six loadings converge to steep, sustained production and the curves separate by temperature far more than by loading. Because each condition was simulated once for 500 ps, every trajectory is a single stochastic realisation of the underlying reactive dynamics; trajectory-to-trajectory variability at fixed conditions cannot be assessed from this dataset, and the performance estimates reported below consequently quantify accuracy on unseen realisations of already-sampled conditions rather than the full physical variability of the process.

After the equilibration window is excluded (median 2.0 ps, mean 3.2 ps, maximum 17.0 ps; Section 3.2), the dataset supports 72,117 origin–target pairs at the primary horizon h=25 ps, which is split into 57,658 training rows from 36 simulations and 14,459 test rows from the 9 held-out simulations (Section 3.5). The target distribution is strongly right-skewed: 18.2% of the pairs have a zero target, the mean target is 15,191 molecules·ps against a median of 2140, and the maximum is 151,612 molecules·ps. Because cumulative H_2_ is non-decreasing along a trajectory, every target satisfies cumH2(t+h)≥cumH2(t), so the persistence value is always a lower bound on the target. This monotonicity underlies the strength of the persistence forecast in slowly evolving regimes and frames the central question of this work: how much do the thermochemical observables and the H_2_ history improve on carrying the current level forward, and does any of that improvement survive when the H_2_ history is withheld entirely (Case B, Section 2.3)?

### 2.2. Case A: Forecasting with the H_2_ History

In Case A, each model receives the autoregressive feature cumulative_H2=cumH2(t) together with six instantaneous descriptors retained by the selection pipeline of Section 3.3: the simulation time *t*, the species counts $N_{C_{2}H_{4}}$ and $N_{{CH}_{4}}$, and the energy components $E_{p}$, $E_{b}$, and $E_{a}$. All inputs are computed at the origin time *t* and the target is fixed at t+25 ps, so the design is an autoregressive forecasting setup in the leakage-aware sense [39]: the model observes the present state of the trajectory, never the future it is asked to predict. Within this regime, the persistence forecast, repeat the current H_2_ level, is the minimal benchmark that any learned model must beat in the same way that naive carry-forward baselines anchor operational forecasting systems [41].

Table 1 reports the pooled out-of-fold accuracy of all eleven models under the nested grouped cross-validation protocol (Section 3.5) both for the primary first-pass run and as the mean ± standard deviation over the five repeated grouped 5-fold runs (seeds 42–46). Gaussian process regression achieves the best out-of-fold accuracy (RMSE 267.5±25.7 molecules·ps, $R^{2}=0.9999$), which is a 93.5% RMSE reduction with respect to the persistence baseline (4096 molecules·ps). The random forest performs comparably at the repeat-mean level (302.5±3.7) and is the more stable of the two: its repeat standard deviation is 1.2% of its mean versus 9.6% for GPR. The single decision tree follows (354.2±9.8), with XGBoost, the ANN, CatBoost, and MLR forming an intermediate band (RMSE 565.3–850.4 molecules·ps) that still improves on persistence by 79.2–86.2%. CatBoost shows the largest repeat instability among the top models (standard deviation 193.6, about 23.3% of its mean), so the ordering within the leading group should not be over-interpreted. SVR performs substantially less well (18,508±369, $R^{2}=0.606\pm 0.016$); we attribute this to the interaction of the ϵ-insensitive loss and the row subsampling required to make kernel training tractable (Section 3.4) with the strongly right-skewed target, although we did not investigate it further. The physics-informed MLR, restricted to the three design variables, likewise performs far worse than persistence (19,041±75): temperature, O_2_ loading, and elapsed time alone cannot reproduce the nonlinear, temperature-gated onset of H_2_ production. The physics-informed random forest is the only baseline that improves substantially on persistence (2597±39, $R^{2}=0.992$), yet it is dominated by every full-feature model except SVR.

**Figure 2:**
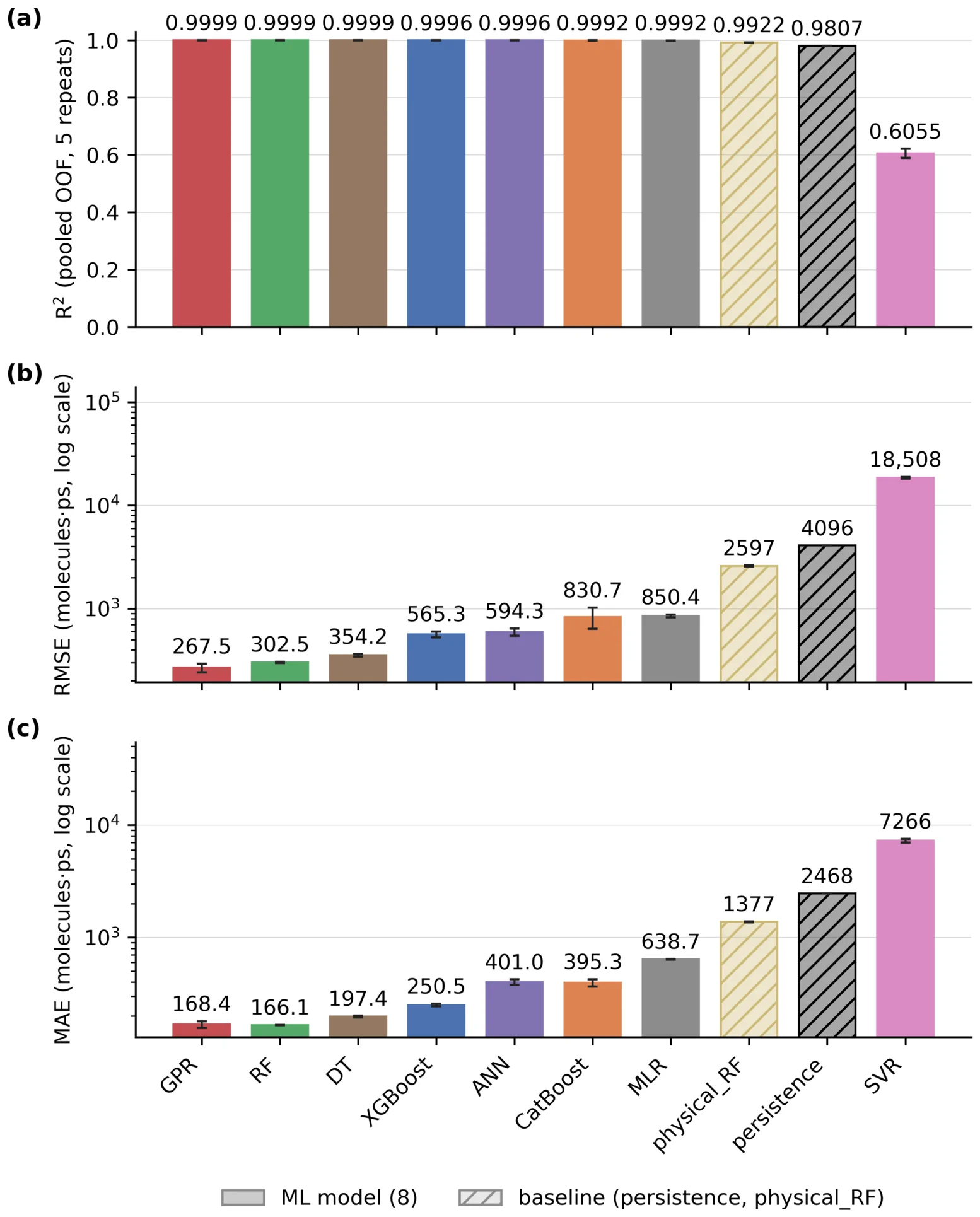
Case A (with H_2_ history) out-of-fold performance over the 36 training simulations: pooled $R^{2}$ (**a**), RMSE (**b**), and MAE (**c**) for the eight ML models and the persistence and physics-informed random-forest (RF) baselines (hatched). Bars show the mean over five stratified grouped 5-fold repeats, error bars the ±1 standard deviation, and annotations the exact means; models are sorted best-first by RMSE with RMSE and MAE on logarithmic axes. The physics-informed MLR is omitted from the figure (dominated everywhere; Table 1).

Figure 2 visualises the same out-of-fold aggregation. The bars are ordered best-first by RMSE and carry ±1 standard-deviation error bars over the five repeats; the persistence and physics-informed random-forest baselines are hatched. The physics-informed MLR is omitted from the figure to avoid crowding, as it is dominated everywhere and is reported in Table 1. The visual summary makes the two-tier structure of the benchmark apparent: seven of the eight ML models cluster two orders of magnitude below the persistence RMSE, while SVR sits above it.

Table 2 reports the held-out test performance on the nine reserved simulations (14,459 pairs). The partition spans five of the eight temperatures (1500, 1700, 1900, 2300, and 2700 K), five of the six O_2_ loadings ($n(O_{2})=1$–900), and deliberately includes two zero-H_2_ simulations, so it probes both onset and high-production regimes. All models were fitted once on the 36 training simulations with the hyperparameters frozen by the nested procedure; no refitting on test data occurred in any form (Section 3.5). Seven of the eight ML models improve on persistence by 63.4–93.0% RMSE. The random forest attains the best test accuracy ($R^{2}=0.9999$, RMSE =121.8 molecules·ps, MAE =79.7) with the nested-CV winner GPR close behind (158.6 molecules·ps); DT, XGBoost, and CatBoost follow within 262.4 molecules·ps. For the physical scale, because the horizon is fixed at h=25 ps, an RMSE of *X* molecules·ps corresponds to an RMS error of X/25 molecules in the H_2_ population averaged over the forecast window, so the random forest mispredicts the window-averaged population by about 4.9 molecules, whereas the persistence forecast (1745 molecules·ps) misses by about 70. The ANN (507.4) and MLR (638.2) remain 70.9% and 63.4% below the persistence RMSE, respectively. SVR transfers poorly (RMSE 5413, 210.2% above persistence), and the physics-informed MLR records a negative test $R^{2}$ (−0.500), i.e., it performs worse than a constant forecast at the test-mean level. The physics-informed RF improves on persistence by 31.4% but is again dominated by every full-feature ML model except SVR. We emphasise the scope of this result: the test simulations are new trajectories, but their temperature–loading combinations lie inside the sampled envelope, so the test partition measures interpolation to unseen realisations of already-sampled conditions rather than extrapolation to new operating conditions; the latter is examined separately under structured holdouts in Section 2.4.

**Figure 3:**
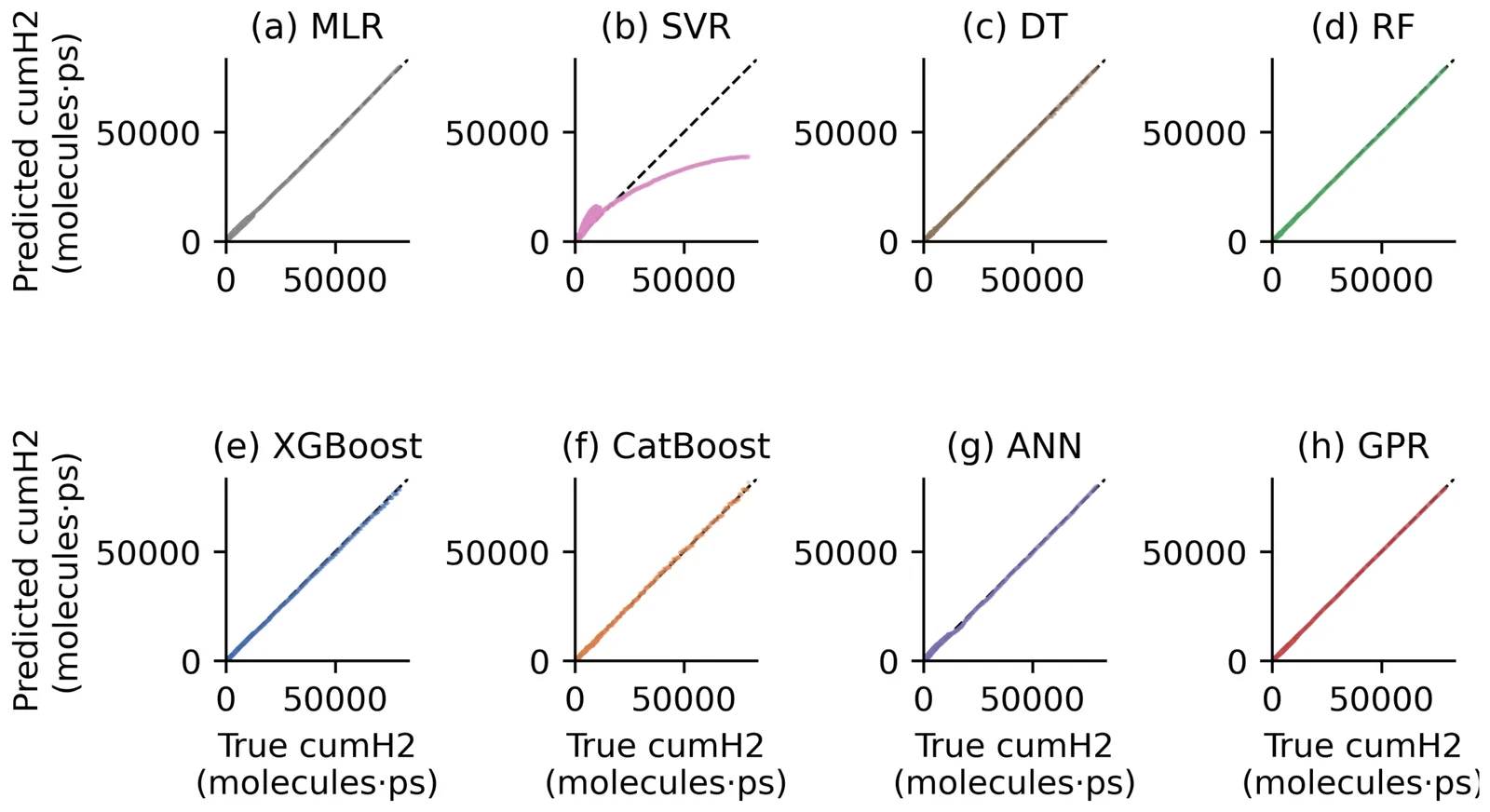
Case A (with H_2_ history) held-out test parity for the eight ML models: (**a**) MLR, (**b**) SVR, (**c**) DT, (**d**) RF, (**e**) XGBoost, (**f**) CatBoost, (**g**) ANN, and (**h**) GPR, predicting cumH2(t+25ps) over the 9 held-out simulations; the dashed line is the identity, and the test $R^{2}$ and RMSE (molecules·ps) of each model are listed in Table 2.

The parity plots of Figure 3 corroborate these observations. The predictions of RF, GPR, DT, XGBoost, and CatBoost lie on the identity line across the entire target range, which spans more than three orders of magnitude up to 151,612 molecules·ps. The ANN shows greater dispersion around the identity line at high production levels, consistent with its larger RMSE, and SVR bends systematically toward the training mean at high targets, which caps its dynamic range. MLR remains close to the identity line at low-to-intermediate levels, consistent with its high test $R^{2}$, but shows visible scatter at the highest production levels.

At the per-simulation level, the GPR test RMSE ranges from 112.5 molecules·ps ($n(O_{2})=100$ at 2300 K) to 275.5 molecules·ps ($n(O_{2})=700$ at 2300 K). On the two zero-H_2_ test simulations, the target is identically zero, so the total sum of squares in Equation (3) vanishes; under the convention adopted there, persistence, which predicts exactly zero, attains a per- simulation RMSE of 0, whereas any model with small nonzero predictions receives $R^{2}=0$ despite tiny errors (GPR: 11.9 and 15.1 molecules·ps). Persistence itself is worst on the $n(O_{2})=900$ simulation at 2700 K (4647.3 molecules·ps), the regime with the steepest production, where carrying the current level forward lags the rapidly rising target.

A recurring concern with temporally autocorrelated regression data is silent overfitting that grouped protocols may not fully expose. We therefore compared each model’s accuracy on its own training partitions with its out-of-fold accuracy. In Case A, the train-to-validation $R^{2}$ gap does not exceed 0.0003 for any model (e.g., RF decreases from 0.99999 on the training partitions to 0.9999 out of fold, and the GPR training RMSE of 38.3 molecules·ps grows to 239.3–290.2 out of fold), so within this protocol, the models do not memorise trajectory-specific patterns, and the nested estimates are not inflated by selection overfitting [40]. The same comparison in Case B reveals a different picture (Section 2.3).

The strong showing of MLR is meaningful because with the H_2_ level available at the origin, the 25 ps-ahead target is, to first order, a near-linear function of the current level, and the linear model already implements the corresponding persistence-plus-correction mapping, reaching $R^{2}=0.997$ on the test set. The additional RMSE reduction delivered by the leading flexible models relative to MLR (638.2 down to 121.8–262.4 molecules·ps) quantifies the genuinely nonlinear component of the mapping (the small, physically grounded correction that tree splits and kernel embeddings learn on top of the autoregressive anchor). This interpretation is consistent with the SHAP attribution of Section 2.5, in which cumulative_H2 dominates the feature ranking.

Finally, to verify that the headline test numbers are not driven by the zero-target convention on the two zero-H_2_ test simulations, we recomputed the test metrics on the seven nonzero-H_2_ simulations alone (13,063 pairs) and found the reported values robust: the RF RMSE is 128.1 molecules·ps and the GPR RMSE is 166.8 molecules·ps, which are values that are close to the full-partition results of Table 2.

### 2.3. Case B: Forecasting Without H_2_ History

Case B removes the autoregressive anchor: no H_2_-derived feature of any kind is available, and the selection pipeline of Section 3.3 retains five instantaneous descriptors: the energy components $E_{p}$, $E_{b}$, and $E_{a}$, the C_2_H_4_ count $N_{C_{2}H_{4}}$, and the simulation time *t*. The target remains cumH2(t+25ps), so every model must reconstruct the H_2_ production level from the instantaneous thermochemical state alone. This scenario corresponds to the practical situation in which H_2_ has not yet been observed, for example at process start-up or in a reactor without H_2_ monitoring, where a forecast must be formed from currently measurable operating and state variables. The evaluation protocol, the 36/9 grouped split, and the cross-validation seeds are identical to Case A (Section 3.5), which makes the two cases a paired comparison (Section 2.4).

Table 3 reports the pooled out-of-fold accuracy: *no* ML model improves on the persistence baseline, whose out-of-fold RMSE is 4096 molecules·ps ($R^{2}=0.981$, standard deviation zero by construction). The best-performing ML model, the ANN, reaches an out-of-fold RMSE of 6756±1864 molecules·ps ($R^{2}=0.944\pm 0.030$), 64.9% above persistence, with by far the largest repeat dispersion of the benchmark (standard deviation 27.6% of its mean), so its ranking is itself uncertain. The physics-informed RF on the three design variables (8934±480) and GPR (9005±1364) follows. The four tree-based ML models and the MLR cluster between 16,346 and 23,457 molecules·ps. Notably, for these models, the 38-feature candidate pool of Section 3.3 performs *worse* than the three-variable physics-informed baseline, indicating that without the H_2_ anchor, the additional instantaneous descriptors contribute noise rather than signal. SVR (24,786±290) and the physics-informed MLR (19,041±75, identical to Case A by construction) close the field.

**Figure 4:**
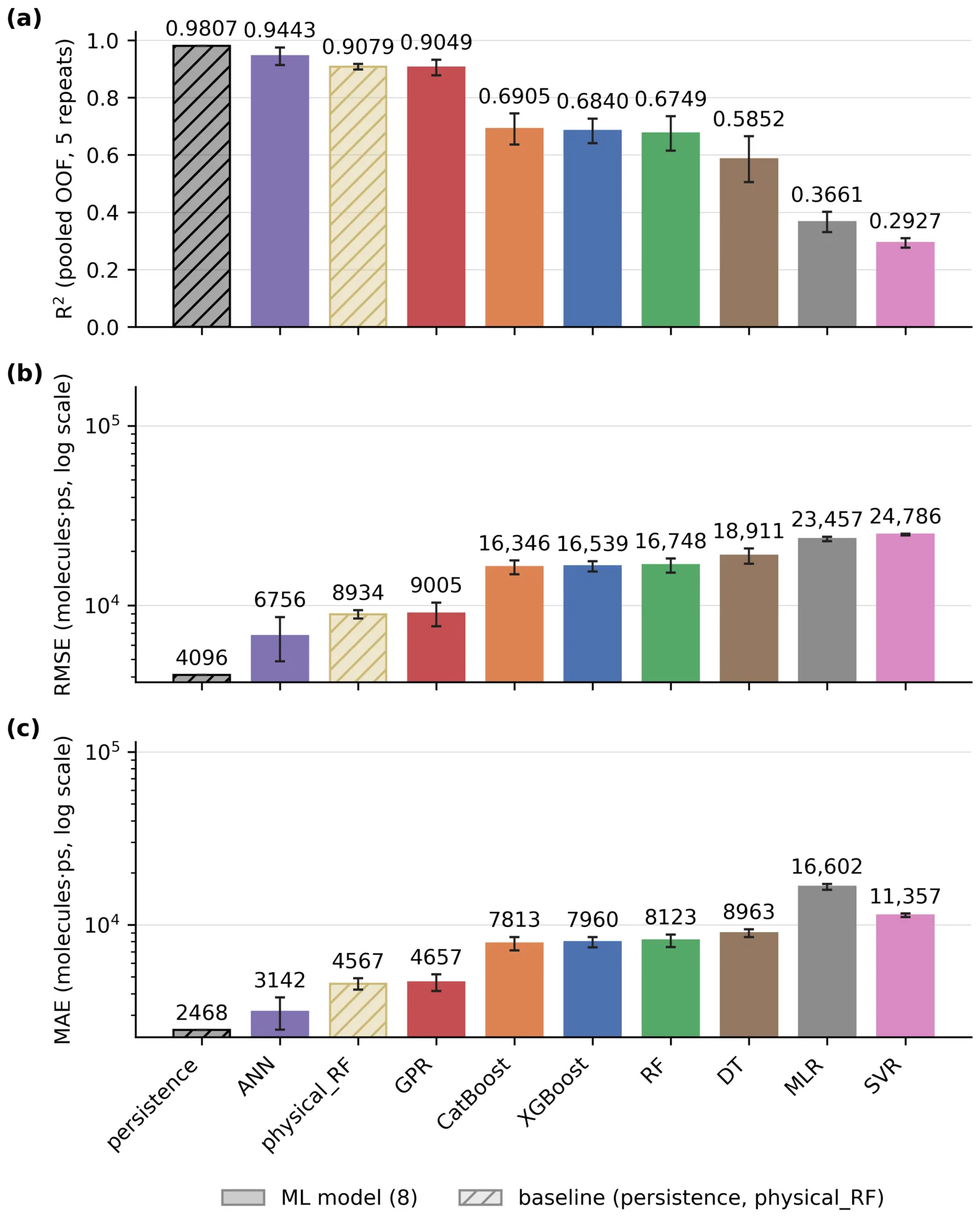
Case B (without H_2_ history) out-of-fold performance over the 36 training simulations in the format of Figure 2: pooled $R^{2}$ (**a**), RMSE (**b**), and MAE (**c**), means over five stratified grouped 5-fold repeats with ±1 standard-deviation error bars. Without the autoregressive cumulative_H2 input, no ML model reaches the persistence baseline; the physics-informed MLR is omitted from the figure (Table 3).

Figure 4 visualises the same out-of-fold aggregation in the format of Figure 2. The flat persistence bar (standard deviation zero by construction) lies below every ML bar, and the spread of the ML means, from  6756 to 24,786 molecules·ps, contrasts with the two-orders-of-magnitude compression of the same models in Case A. The figure makes the honest conclusion unavoidable: removing the H_2_-history feature, not the choice of model class or the extent of hyperparameter tuning, is what separates the two regimes.

The held-out test partition tells the same story (Table 4). Persistence again performs best (RMSE =1745 molecules·ps, $R^{2}=0.980$). The best-performing ML model, the ANN, attains $R^{2}=0.968$ at RMSE =2199 molecules·ps, still 26.0% worse than persistence, which is followed by GPR ($R^{2}=0.963$, RMSE =2361, 35.3% worse). In the same molecule reading, the ANN mispredicts the window-averaged H_2_ population by about 88 molecules versus about 70 for persistence. The remaining models degrade monotonically down to the MLR, whose test $R^{2}$ of −0.885 is worse than a constant forecast at the test-mean level. The ANN and GPR test errors, taken in isolation, look respectable ($R^{2}$ above 0.96); it is the persistence anchor that reveals their shortfall, which is precisely why skill relative to persistence is reported throughout (Section 3.5) [41]. The two models that transfer best are those least reliant on a high-capacity memorisation of the instantaneous descriptors, whereas the tree ensembles that excelled in Case A here degrade to $R^{2}=0.757$–0.830: the information content of the inputs, not the learning algorithm, is the binding constraint.

**Figure 5:**
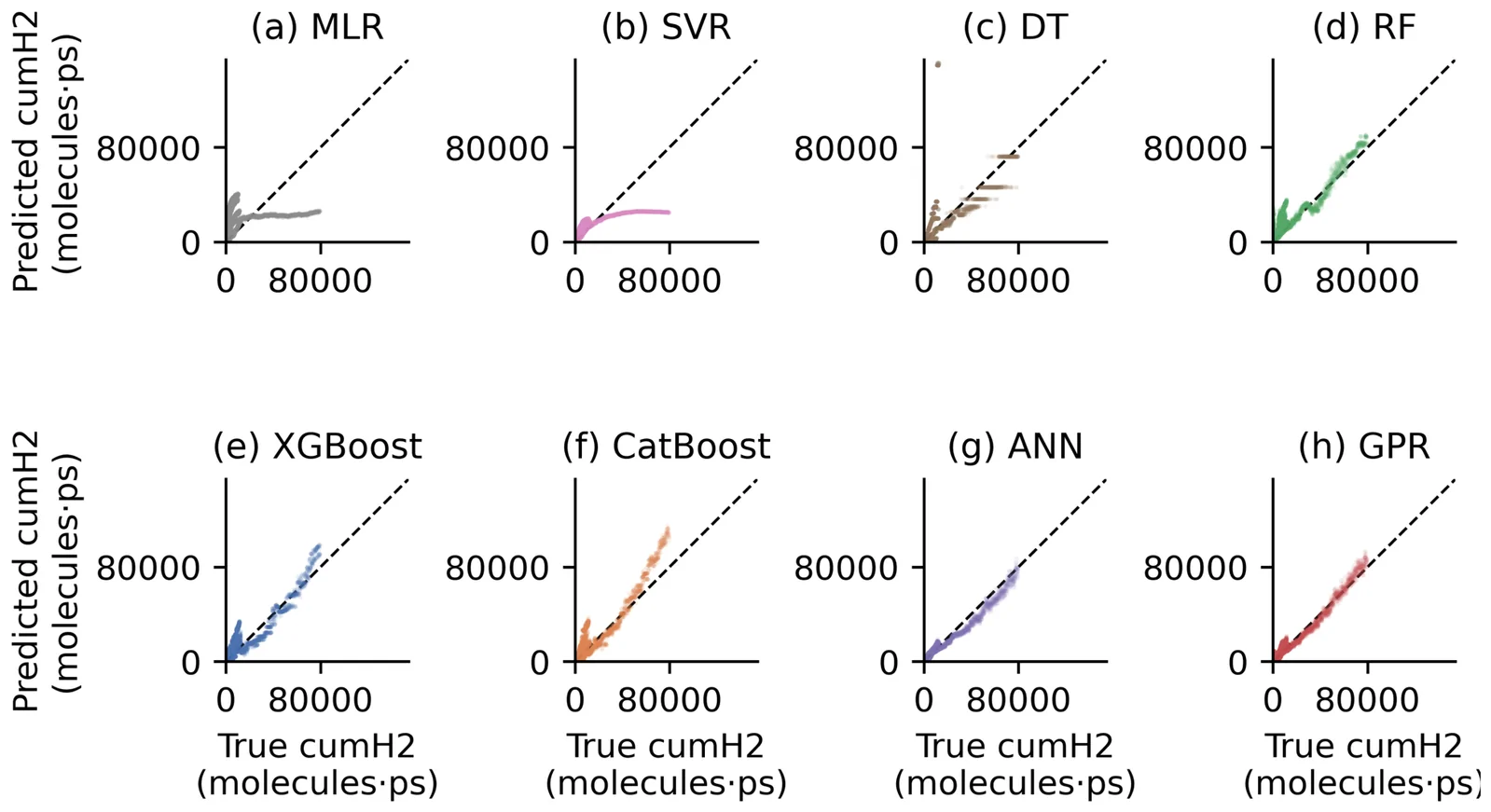
Case B (without H_2_ history) held-out test parity for the eight ML models in the format of Figure 3: (**a**) MLR, (**b**) SVR, (**c**) DT, (**d**) RF, (**e**) XGBoost, (**f**) CatBoost, (**g**) ANN, and (**h**) GPR over the same held-out test rows; the test $R^{2}$ and RMSE (molecules·ps) of each model are listed in Table 4.

The parity plots of Figure 5 expose the failure mode. In contrast to Case A, every model shows greater dispersion around the identity line, and the errors are structured rather than random: at low true values (the early-time H_2_-onset regime of the unseen test simulations), the models systematically predict positive production that has not yet occurred, whereas at high true values, the predictions lag and compress the target range. The ANN and GPR panels are the closest to the identity line, consistent with Table 4, but both retain a visible low-target bias; the tree-model panels show greater dispersion around the identity line over the full range, and the MLR panel shows essentially no exploitable correlation.

The learning diagnostics explain why the Case B models can appear strong yet transfer poorly. Unlike Case A, where the train-to-validation $R^{2}$ gap never exceeded 0.0003 (Section 2.2), the Case B models fit their training partitions almost perfectly while validating far worse: the single decision tree decreases from $R^{2}=0.9999$ on its training partitions to 0.421 out of fold, the random forest from 0.9999 to 0.624, CatBoost from 0.997 to 0.693, GPR from 0.998 to 0.670, and the ANN from 0.991 to 0.826. Gaps of this size are the signature of simulation-specific overfitting [40]: without the autoregressive anchor, each model memorises trajectory-specific signatures of the instantaneous descriptors (for example, condition-specific correlations between energy components and production) that do not survive the grouped split. The nested protocol contains this overfitting (the reported scores are honest out-of-fold numbers), but no amount of regularisation restores information that the inputs do not contain.

As in Case A, we verified that the zero-target convention does not drive these conclusions: restricted to the seven non-zero-H_2_ test simulations (13,063 pairs), the ANN RMSE is 2311 against a persistence RMSE of 1836 molecules·ps, so the best-performing ML model remains worse than persistence on the production-active simulations alone.

Taken together, the Case B results indicate that the instantaneous thermochemical observables carry only weak information about the 25 ps-ahead H_2_ level. High out-of-fold $R^{2}$ values for the ANN and GPR (0.944 and 0.905) reflect between-condition variance (hotter, more lightly loaded systems produce more H_2_) rather than the ability to locate the production level of a particular trajectory, which is what the task requires. This result is not an artefact of model choice or tuning: eight model families spanning linear, kernel, tree, ensemble, and neural architectures, each tuned inside a leakage-free nested protocol, fail to beat a one-line carry-forward rule. The practical implication is that H_2_ forecasting from ReaxFF trajectories requires either the H_2_ history itself or sequence models that can accumulate context over the observed trajectory; purely instantaneous descriptors are insufficient, and the paired ablation and structured-holdout experiments of the next section quantify exactly how much is lost.

### 2.4. Comparison of the Two Cases and Generalisation to Unseen Conditions

The two cases were evaluated on identical group partitions with identical seeds (Section 3.5), so the within-model contrast between them is a paired ablation that isolates the predictive contribution of the H_2_-derived candidate features, above all the autoregressive cumulative_H2, under an otherwise unchanged protocol. Table 5 reports, for every model, the first-pass out-of-fold RMSE in each case together with the paired RMSE reduction $\Delta {\mathrm{RMSE}}_{A|B}=100\;\left({\mathrm{RMSE}}_{B}-{\mathrm{RMSE}}_{A}\right)/{\mathrm{RMSE}}_{B}$, where positive values indicate that Case A (with H_2_ history) achieves the lower error.

Every ML model, on every repeat and on the held-out test partition, is better in Case A than in Case B under identical fold assignments. For the tree-based models and GPR, 93.3–98.3% of the Case B RMSE is removed by restoring the H_2_ history; for the ANN, the paired reduction is smaller on the test partition (76.9%) because the ANN is simultaneously the strongest and the most repeat-dependent Case B model. Two control rows verify the pairing: persistence, which uses no features, is identical in the two cases by construction, and so is the physics-informed MLR. The physics-informed RF is the one baseline that differs between the cases: in Case B, it is restricted to the three design variables (temperature, O_2_ loading, simulation time), whereas in Case A, it additionally receives the H_2_-history feature, which is consistent with the Case A information regime; its paired delta of +74.6% therefore measures the same effect in a low-capacity model. The small SVR delta (+22.2%) should not be read as evidence that SVR depends less on the H_2_ history: SVR performs poorly in both cases (Section 2.2), so its paired ratio is computed from two large errors.

Before turning to extrapolation, we address the framing of Case A explicitly, because an autoregressive feature can superficially resemble target leakage. It is not: leakage in the sense of Kaufman et al. [39] occurs when information about the evaluation target enters the training inputs, for example, when rows from the same trajectory appear on both sides of a split, or when a feature encodes the future. In Case A, every input is computed at the origin time *t* and the target is cumH2(t+25ps), so the model never sees the quantity it is asked to predict; the grouped splits further guarantee that no trajectory contributes pairs to both sides of any split. Forecasting the near future of a cumulative quantity from its own present value is the defining property of an autoregressive forecast, and the persistence forecast is exactly its zeroth-order realisation [41]. The paired ablation then answers the well-posed question: how much of the achievable skill comes from this temporal autocorrelation signal rather than from the instantaneous physical observables? For the models that perform well, the answer is 93.3–98.3%.

**Figure 6:**
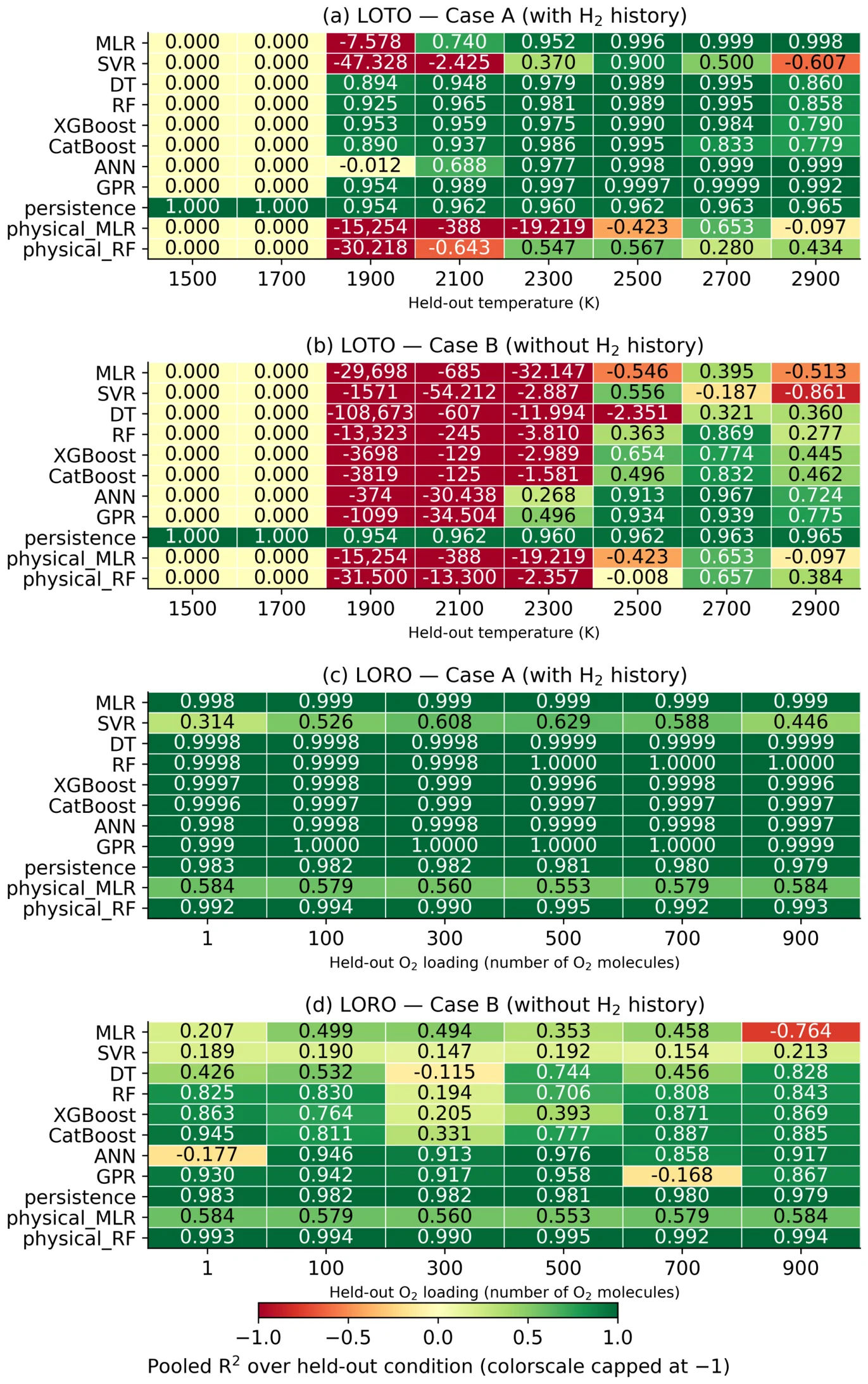
Generalisation under structured holdouts: pooled $R^{2}$ per model (rows) and held-out condition (columns) for leave-one-temperature-out (LOTO; (**a**) Case A, (**b**) Case B) and leave-one-O_2_-loading-out (LORO; (**c**) Case A, (**d**) Case B); cell annotations give the pooled $R^{2}$ of the held-out condition. Because the values span −108,673 to 1.0, the diverging colour scale is clipped at −1 for display only; the annotation always shows the true value. The T1500/T1700 LOTO columns contain only zero-H_2_ simulations, where the degenerate-target convention applies (Section 2.2).

The held-out test partition of the previous subsections samples temperature–loading combinations inside the simulated envelope. To probe extrapolation to conditions absent from training entirely, the LOTO protocol holds out all simulations of one temperature in turn (8 folds) and the LORO protocol holds out all simulations of one O_2_ loading in turn (6 folds); in both protocols, the held-out condition is absent from feature selection, hyperparameter tuning, and training, so the scores measure genuine condition-level extrapolation [38]. Figure 6 presents the full pooled-$R^{2}$ heatmaps.

In Case A (Figure 6a), the T1500 and T1700 columns contain only zero-H_2_ simulations, whose targets are identically zero; under the degenerate-target convention introduced in Section 2.2, persistence, which predicts exactly zero, scores $R^{2}=1.0$ there, while the ML models, whose small nonzero predictions incur tiny errors (e.g., GPR RMSE 7.2 molecules·ps at T1500), score $R^{2}=0.0$. These two columns are convention artefacts of empty-variance targets—not extrapolation failures. Across the informative folds, the picture is largely positive: GPR attains $R^{2}\geq 0.95$ in every informative fold, including 0.954 at T1900, where it matches persistence (0.954), and 0.989–0.9999 from T2100 through T2700. The single hardest informative fold for the tree ensembles is T2900, where RF, XGBoost, and CatBoost decrease to 0.858, 0.790, and 0.779, respectively, while the ANN reaches 0.9992 there (RMSE 1242 molecules·ps); conversely, the ANN performs less well at the two lowest informative temperatures ($R^{2}=-0.012$ at T1900 and 0.688 at T2100). Pooled over the eight folds, the ANN is the best LOTO model ($R^{2}=0.9994$, RMSE 647.1 molecules·ps), and persistence holds a uniform 0.954–0.965. The reading is that temperature extrapolation mostly, but not uniformly, preserves Case A accuracy: the H_2_ history transfers across temperature with degradation concentrated at the extreme T2900 K end for tree models.

In Case B (Figure 6b), temperature extrapolation decreases predictive accuracy substantially. At T1900, every ML model produces strongly negative pooled $R^{2}$: the decision tree reaches −108,673 ($-1.1\times 10^{5}$ in compact annotation), RF −13,323, CatBoost −3819, XGBoost −3698, GPR −1099, and the ANN −374. At T2100, all eight ML models remain negative (GPR −35, ANN −30, XGBoost −129, RF −245). Recovery begins only at T2300 (ANN 0.268, GPR 0.496) and is partial at the highest temperatures (ANN 0.967 and GPR 0.939 at T2700; ANN 0.724 and GPR 0.775 at T2900). Pooled over folds, the best Case B LOTO model is GPR ($R^{2}=0.895$, RMSE 8850 molecules·ps), but persistence, at 0.954–0.965 in every fold, dominates every ML model in every informative fold. The failure mode echoes the parity plots of Figure 5: a model that must infer the H_2_ onset from instantaneous descriptors of an unseen temperature cannot know when production actually begins, and its errors are then squared into the pooled $R^{2}$.

The LORO results (Figure 6c,d) show that the two extrapolation axes are far from equivalent. With the H_2_ history available (Figure 6c), loading extrapolation is essentially solved: every tree-based model and the GPR attain $R^{2}\geq 0.9987$ in all six folds (RF 0.9998–1.0000, GPR 0.9994–1.0000; the worst tree/GPR cell is CatBoost at *n*(O_2_) = 300, $R^{2}=0.9987$), the best pooled model is RF ($R^{2}=0.9999$, RMSE 261.5 molecules·ps), and even its worst condition (*n*(O_2_) = 1) stays at RMSE 401.7 molecules·ps, $R^{2}=0.9998$. Only SVR (0.314–0.629) and the design-variable baselines degrade. Without the H_2_ history (Figure 6d), the best pooled LORO model is the three-variable physics-informed RF ($R^{2}=0.993$, RMSE 2314 molecules·ps, per-fold range 0.990–0.995), a three-feature baseline that generalises across loadings better than any full-pool ML model, while the full-feature models fail at characteristic loadings: the intermediate *n*(O_2_) = 300 fold exposes the tree ensembles (XGBoost $R^{2}=0.205$, CatBoost 0.331, RF 0.194, DT −0.115), the sparse *n*(O_2_) = 1 fold is worst for the ANN ($R^{2}=-0.177$, RMSE 29,241 molecules·ps), and the *n*(O_2_) = 700 fold is worst for GPR ($R^{2}=-0.168$). Persistence again never falls below 0.979.

With the H_2_ history available, forecasting is robust to unseen O_2_ loadings across the entire model spectrum and mostly robust to unseen temperatures, the residual weakness being concentrated in tree ensembles at the temperature extreme (T2900 K) where production is steepest. Without the H_2_ history, no model family generalises across either axis, and the only procedures that transfer are those that assume the least: the persistence forecast and, across loadings, the physics-informed baseline. Within the analysed envelope, Case A accuracy is interpolation plus the structured extrapolation of a strongly autocorrelated signal; Case B never acquires that signal in the first place, and its apparent within-envelope skill (Section 2.3) does not survive the structured holdouts.

### 2.5. SHAP Analysis

SHapley Additive exPlanations (SHAP) were applied to the best-performing model of each case to attribute its predictions to the input features [42]. SHAP values quantify each feature’s contribution to the deviation of an individual prediction from the background expectation, and their mean absolute value provides a model-faithful importance ranking; the framework has become a standard instrument of explainable artificial intelligence [43,44,45]. Because exact TreeSHAP is defined for tree-based models, the analysis follows a documented substitution rule: the best ML model of each case was selected by first-pass out-of-fold $R^{2}$ among the eight ML models, and, where that model is not tree-based, TreeSHAP is applied to the best tree-based model of the case instead. In Case A, the overall best model is GPR, so the attribution uses the random forest (out-of-fold $R^{2}=0.9999$); in Case B, the overall best model is the ANN, so the attribution uses XGBoost (out-of-fold $R^{2}=0.730$). Main-effect attributions were computed with the interventional TreeExplainer [46], using a background sample of 100 uniformly random training rows (seed 42) and evaluated on all 14,459 held-out test rows; pairwise interaction strengths were computed with a tree-path-dependent explainer on a 2000-row random test subsample (seed 42), because the installed SHAP release does not support interventional interaction values. The exact explainer settings are collected in the Supplementary Materials. All SHAP values carry the units of the target, molecules·ps.

**Figure 7:**
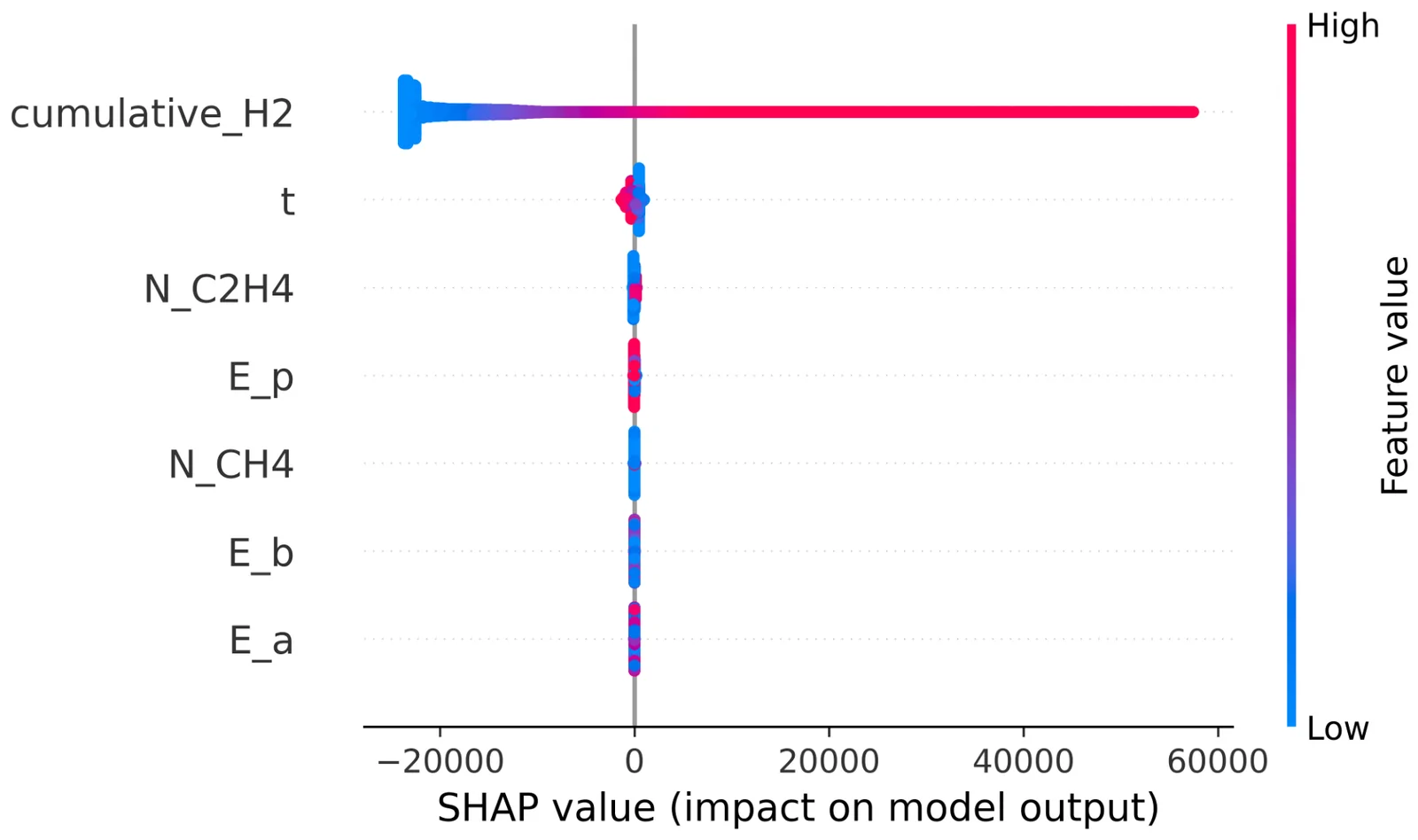
SHAP beeswarm summary for the Case A random forest, evaluated on the 14,459 held-out test rows. Each dot is one test row; features are ordered top-to-bottom by mean |SHAP|, and  colour encodes the feature value (blue = low, red = high). The  autoregressive cumulative_H2 history dominates the ranking; the values are collected in Table 6.

In Case A, the attribution is strikingly concentrated (Figure 7). The autoregressive cumulative_H2 feature dominates the ranking: its mean |SHAP| of 21,002 molecules·ps is fifty times that of the second-ranked feature, the simulation time *t* (414.3), which is followed by the C_2_H_4_ count (75.1), the potential energy (24.7), the CH_4_ count (9.5), the bond energy (8.5), and the angle energy (1.9). The dominant contribution of cumulative_H2 explains the high predictive performance in Case A: once the current H_2_ level is known, the remaining features refine a forecast that is already nearly correct, which is precisely the near-linear reducibility exploited by the MLR baseline and quantified by the paired ablation of Section 2.4. The dependence plot (Figure S6 in the Supplementary Materials) shows the SHAP value of cumulative_H2 increasing near-monotonically with the feature value: the further a trajectory has progressed, the more its own H_2_ history drives the 25 ps-ahead forecast. Consistently, the strongest pairwise interaction is (cumulative_H2, *t*) with a mean interaction strength of 272.3 molecules·ps (Figure S4 in the Supplementary Materials); the strongest diagonal main effect is cumulative_H2 at 15,742 molecules·ps. In the normalised interaction heatmap, every cell is divided by the largest off-diagonal mean interaction and the diagonal is 1.0 by definition, so the (cumulative_H2, *t*) cell is the reference off-diagonal value.

**Table 6:** Mean absolute SHAP values (molecules·ps) per feature for the best tree-based model of each case: the random forest for Case A (final refit: 7 features) and XGBoost for Case B (final refit: 5 features), evaluated on the 14,459 held-out test rows with the interventional TreeExplainer (background = 100 random training rows, seed 42). Features are sorted by the Case A mean |SHAP|; “n/a” marks features absent from the final Case B model.

Feature	Case A (RF)	Case B (XGBoost)
cumulative_H2	21,002	n/a
*t*	414.3	3564
$N_{C_{2}H_{4}}$	75.1	8337
$E_{p}$	24.7	10,620
$N_{{CH}_{4}}$	9.5	n/a
$E_{b}$	8.5	19,555
$E_{a}$	1.9	5629

**Figure 8:**
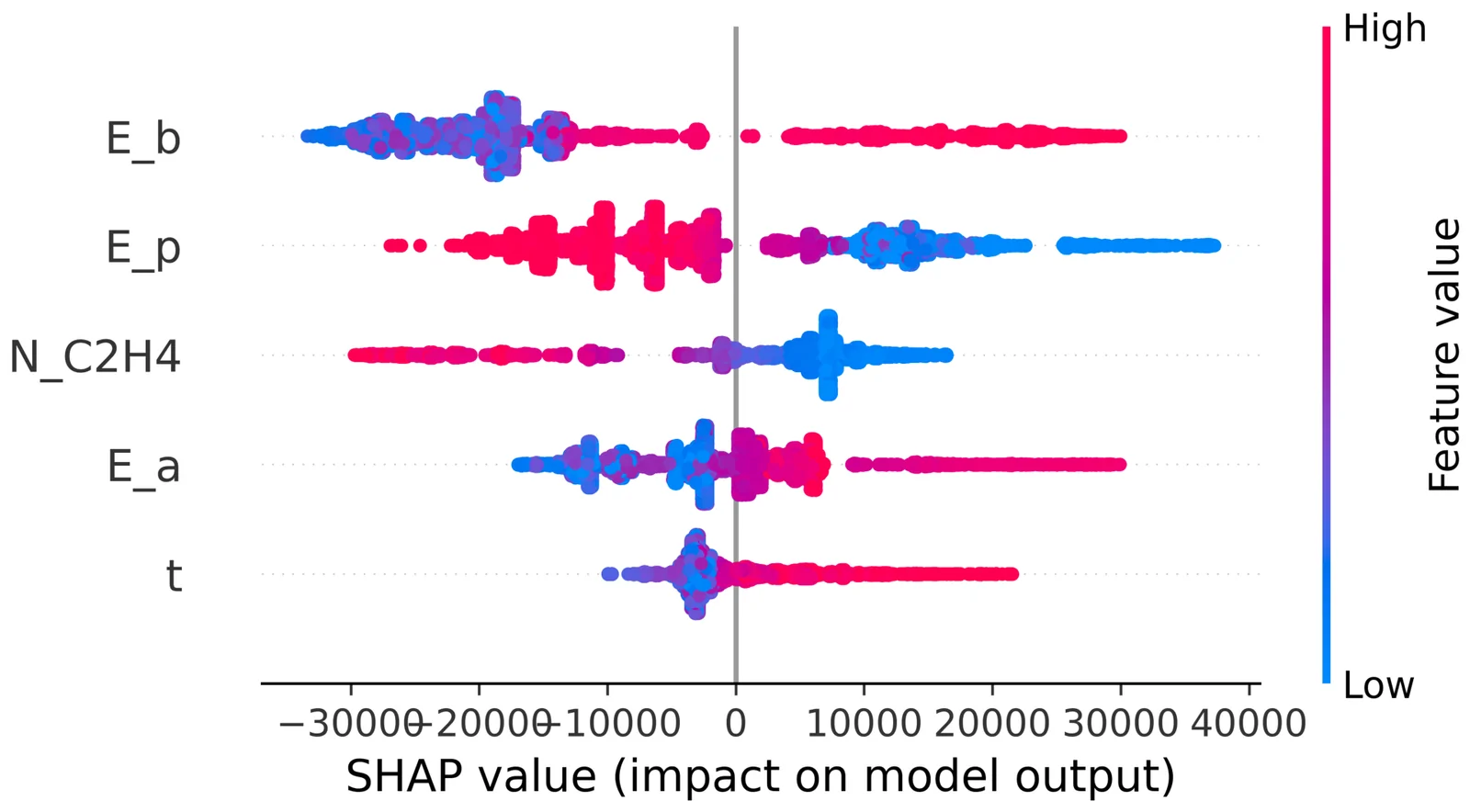
SHAP beeswarm summary for the Case B XGBoost model, evaluated on the 14,459 held-out test rows, in the format of Figure 7: without the H_2_ history, the attribution spreads over the five final features, which are ranked from $E_{b}$ (highest) to *t* (lowest).

The Case B attribution is distributed across the five instantaneous descriptors (Figure 8). The bond energy $E_{b}$ ranks first (19,555 molecules·ps), which is followed by the potential energy $E_{p}$ (10,620), the C_2_H_4_ count (8337), the angle energy $E_{a}$ (5629), and finally the simulation time *t* (3564): without the H_2_ history, time has the lowest SHAP importance of the final feature set. The models key on instantaneous thermochemistry (the energy components respond bond-by-bond to the fragmentation of the chain and therefore carry a weak, indirect onset signal) rather than on any observable that actually accumulates H_2_ production. The strongest off-diagonal interaction is the in-chain pair ($E_{p}$, $E_{a}$) at 3696 molecules·ps (Figure S5 in the Supplementary Materials) with the strongest diagonal main effect $E_{p}$ at 9817 molecules·ps, i.e., the XGBoost model combines the instantaneous potential-energy and angle-energy signatures when estimating its (weak) onset signal. The dependence plot (Figure S7 in the Supplementary Materials) shows steeper, more threshold-like transitions than in Case A (Figure S6), which is consistent with a signal that switches on as the degradation chemistry accelerates.

Three caveats bound the interpretation. First, SHAP attributions are conditional on the model and its feature set: they describe how the fitted model associates predictions with features, not causal physical mechanisms, and a feature’s importance can reflect correlations with unobserved drivers. Second, the features are not statistically independent: cumulative_H2 and *t* both grow along a trajectory and are strongly correlated, as are the energy components in Case B. Interventional TreeSHAP mitigates, but does not eliminate, the redistribution of importance among correlated features [46,47], so the attributions of such feature groups should be read jointly; the dominance of cumulative_H2 in Case A is robust to this concern because its information content is corroborated independently by the paired ablation (93.3–98.3% of RMSE, Section 2.4). Third, the substitution rule means the attributions characterise the best tree-based model of each case (RF in Case A, XGBoost in Case B) rather than the overall best model (GPR and the ANN, respectively); both substituted models track their case winners closely (Table 1 and Table 3), but the substitution is documented wherever it applies.

Read together, the two attribution profiles mirror the accuracy results. With the H_2_ history, one feature carries almost all of the predictive signal, and every capable model learns essentially the same mapping, which is why the Case A model ranking is tight and the MLR baseline already reaches $R^{2}=0.997$. Without it, the models lean on weak, correlated proxies of reaction progress, no feature dominates, and the same architectures fail to beat a carry-forward forecast. The SHAP analysis therefore provides a mechanistic explanation, at the level of the learned models, of why the two cases differ so sharply.

### 2.6. Stability of the Estimates and Descriptive Statistics

In accordance with the evaluation protocol (Section 3.5), the model comparisons of Section 2.2, Section 2.3 and Section 2.4 are purely descriptive: every cross-validation score is summarised as the mean ± standard deviation across the five stratified grouped 5-fold repeats (seeds 42–46), no significance tests are performed, no *p*-values are reported, and effect sizes are read directly from the grouped-CV spreads. With five repeats per protocol, the distributional assumptions of inferential rank tests are difficult to justify, and the between-model differences that carry the conclusions are in any case large relative to the within-model dispersion. Three complementary stability checks substantiate this approach: the variation of the scores across cross-validation repeats, the variation induced by the row subsampling used to train the GPR and SVR models, and the per-simulation distribution of the forecast errors.

Figure S1 in the Supplementary Materials (Section S7) displays the pooled OOF RMSE of every model on each of the five repeats separately. In Case A, GPR attains the leading score on every repeat (239.3–290.2 molecules·ps) and RF the second-best score on every repeat (296.9–306.6 molecules·ps); the worst GPR repeat is still better than the best RF repeat, although the two models are close at the mean level (267.5±25.7 versus 302.5±3.7 molecules·ps) and RF is the more stable of the two, its repeat standard deviation being only 1.2% of its mean against 9.6% for GPR. The remaining models show little repeat-to-repeat movement (DT 354.2±9.8; XGBoost 565.3±36.6; ANN 594.3±48.8; MLR 850.4±25.8 molecules·ps). The one exception is CatBoost, whose repeat standard deviation of 193.6 molecules·ps amounts to 23.3% of its 830.7 molecules·ps mean, the largest relative dispersion among the Case A models (the corresponding $R^{2}$ spread is 0.9992), with individual repeat scores between 722.4 and 1175 molecules·ps. SVR is stable (18,508±369 molecules·ps) but an order of magnitude behind the leading group. The persistence score carries a standard deviation of exactly zero (4096 molecules·ps): the persistence forecast involves no training, so its pooled OOF score is identical whatever partition the repeated cross-validation happens to draw.

In Case B, the same figure makes the negative result visually unambiguous: the flat persistence line at 4096 molecules·ps lies below every ML model in every repeat, the smallest ML score observed on any repeat being 4817 molecules·ps (ANN). The best ML model, ANN, averages 6756±1864 molecules·ps, a repeat standard deviation of 27.6% of the mean, with individual repeat scores ranging from 4817 to 9458 molecules·ps; the corresponding $R^{2}$ spread is 0.944±0.030. ANN is the best ML model on four of the five repeats, and on the remaining repeat, the three-feature physical RF baseline is the only model ahead of it. GPR averages 9005±1364 molecules·ps and the tree models populate a band roughly four times higher still (CatBoost 16,346±1438; XGBoost 16,539±1105; RF 16,748±1530; DT 18,911±1840 molecules·ps), while the physical RF baseline sits at 8934±480 molecules·ps. No repeat, for any model, produces a score at or below the persistence line, so the Case B conclusion does not rest on a favourable partition.

Because the GPR and SVR fits are trained on group-balanced, temporally thinned subsamples of the training rows (Section 3.4), their scores could in principle depend on the subsample draw. Repeating the first-pass folds over five subsample seeds (42–46) gives pooled OOF RMSE values of 294.3±12.1 molecules·ps for GPR in Case A (4.11% of the mean; $R^{2}=0.9999$), 15,960±75 molecules·ps for GPR in Case B (0.47%), 19,698±15 molecules·ps for SVR in Case A (0.08%), and 25,312±10 molecules·ps for SVR in Case B (0.04%). The largest of these relative spreads, the 4.11% observed for GPR in Case A, remains an order of magnitude smaller than the differences that separate the leading models from the remaining field, and none of the four values alters any model ranking. The subsampling procedure therefore does not drive any conclusion of Section 2.2, Section 2.3 and Section 2.4.

The pooled RMSE weights simulations by their number of forecast pairs; the per-simulation view of Section 3.5 complements it with an equally weighted perspective. Figure S2 in the Supplementary Materials summarises the 36 per-simulation first-pass OOF RMSE values of every Case A model. The median per-simulation persistence RMSE is 739.3 molecules·ps (the mean is much larger because the per-simulation persistence distribution is strongly right-skewed, which is why the median serves as the reference line). Every ML model except SVR has a per-simulation median below this line, with GPR lowest at 134.6 molecules·ps, which is followed by RF (161.6), XGBoost (164.9), DT (191.1), CatBoost (280.9), ANN (437.2), and MLR (566.8 molecules·ps); SVR (1953 molecules·ps) and the physical RF baseline (971.2 molecules·ps) lie above it. The heavy right tails visible for the tree and GPR models originate in the low-temperature H_2_-onset simulations, precisely the regime in which forecasting adds the most value over persistence, which propagates slowly through the onset transition.

The same per-simulation view sharpens the Case B negative result (Figure S3 in the Supplementary Materials). The best ML median (ANN, 3667 molecules·ps) sits almost five times above the persistence line of 739.3 molecules·ps, and the persistence line lies below every ML box: without the H_2_ history, not even the equally weighted per-simulation perspective reveals an ML advantage. The three-feature physical RF baseline attains a per-simulation median of 1299 molecules·ps, again below the medians of all eight full-feature ML models, mirroring the pooled ranking in which the reduced physical input set outperforms the larger feature pool for the tree family (Section 2.3).

The per-simulation RMSE values span exact zeros to roughly $6\times 10^{4}$ molecules·ps, which is why Figures S2 and S3 use a symlog axis (linear below 1) rather than a purely logarithmic one. The exact zeros deserve comment. Persistence attains a per-simulation RMSE of exactly zero on all eight zero-H_2_ training simulations in both cases: the targets are identically zero, and the persistence forecast, which repeats the current value, predicts zero exactly. Among the ML models, only the decision tree produces such exact zeros: on one model–simulation combination in Case A and on four in Case B. These zeros are genuine point predictions rather than convention artefacts; the related degenerate-target convention for $R^{2}$ on all-zero targets is described in Section 2.2.

Taken together, the three checks show that both headline findings of this work are stable under every resampling axis examined. The Case A advantage over persistence holds on every cross-validation repeat, every subsampling seed, and in both the pooled and the equally weighted per-simulation aggregates, and the Case B shortfall is equally robust: no resampling, aggregation, or subsampling variant produces a single configuration in which an ML model matches the persistence forecast without the H_2_-derived features.

## 3. Materials and Methods

### 3.1. ReaxFF MD Simulation Setup

All reactive molecular dynamics simulations were performed using the large-scale atomic/molecular massively parallel simulator (LAMMPS, 23 June 2022 stable release) [48] with the ReaxFF reactive force-field method [17,18,20,49].

Six polyethylene–oxygen (PE–O_2_) systems were constructed, each containing 50 linear PE chains of 30 monomers (chain formula C_30_H_62_) plus $n(O_{2})$ oxygen molecules, with $n\left(O_{2}\right)\in \left\{1,100,300,500,700,900\right\}$. The polymer fraction is identical in all six systems: exactly 1500 carbon and 3100 hydrogen atoms, corresponding to a PE mass of 21,141.3 amu, so the total atom count ranges from 4602 to 6400 atoms comprising carbon, hydrogen, and oxygen (Table 7). The six systems therefore span O_2_:PE mass ratios from 0.15% (a trace oxidant) to 136.2% (a more than 1:1 oxidant-to-polymer mass ratio). Initial configurations were generated with Packmol by randomly inserting the required numbers of O_2_ molecules and linear −(CH_2_−CH_2_)n− chains into cubic boxes under periodic boundary conditions; the box side lengths (56.1–75.1 Å) were chosen to give initial densities of approximately 0.20 g/cm^3^ in every system. The original Packmol input files, including the packing tolerances, are no longer available in the project archive; the verbatim LAMMPS input and force-field files used for every simulation are instead provided in the Supplementary Materials.

The interatomic interactions were described by the CHON-2017_weak parameter set [50], which is a reparametrised ReaxFF variant optimised for C/H/N/O systems with attenuated hydrogen-bond strength to suppress excessively reactive recombination pathways previously reported for hydrocarbon–water mixtures. The parameter set has since been benchmarked against the thermophysical properties of water, where it outperformed the water-2017 force field [51], and has been applied to reactive MD studies of plastic and biomass gasification in supercritical-water and oxidative environments [9,14,15].

In the ReaxFF formalism, the total potential energy of the system is expressed as the sum of bonded and nonbonded contributions [17,52]:

(1) $$\begin{array}{cc} E_{\mathrm{system}}=\; & E_{\mathrm{bond}}+E_{\mathrm{lp}}+E_{\mathrm{over}}+E_{\mathrm{under}}\\ \; & +E_{\mathrm{val}}+E_{\mathrm{pen}}+E_{\mathrm{coa}}+E_{C2}+E_{\mathrm{triple}}\\ \; & +E_{\mathrm{tors}}+E_{\mathrm{conj}}+E_{\mathrm{Hbond}}+E_{\mathrm{vdW}}+E_{\mathrm{Coulomb}}, \end{array}$$

where $E_{\mathrm{bond}}$ is the bond-order-dependent bond energy, $E_{\mathrm{lp}}$ the lone-pair energy, $E_{\mathrm{over}}$ and $E_{\mathrm{under}}$ the over- and under-coordination penalties, $E_{\mathrm{val}}$ the valence-angle energy, $E_{\mathrm{pen}}$ the double-bond to single-bond conversion penalty, $E_{\mathrm{coa}}$ the three-body conjugation term, $E_{C2}$ and $E_{\mathrm{triple}}$ corrections to C–C single- and triple-bond energies, $E_{\mathrm{tors}}$ the torsion energy, $E_{\mathrm{conj}}$ the conjugation energy, $E_{\mathrm{Hbond}}$ the hydrogen-bond energy, and $E_{\mathrm{vdW}}$ and $E_{\mathrm{Coulomb}}$ the nonbonded van der Waals and electrostatic interactions evaluated between all atom pairs. All bonded terms depend on a distance-dependent bond order $BO_{ij}$ that smoothly tends to zero as bonds dissociate, enabling seamless transitions between bonding regimes without predefined connectivity; the terms above were evaluated on the fly at each integration step. Atomic partial charges were updated via the charge-equilibration (QEq) method with a convergence tolerance of $10^{-6}$, which was applied at every timestep.

The simulation protocol consisted of two sequential stages. In the first (equilibration) stage, each system was energy-minimised and then equilibrated at 300 K in the canonical (NVT) ensemble for $4\times 10^{5}$ timesteps with a timestep of 0.25 fs, corresponding to a total physical time of 100 ps. The Nosé–Hoover thermostat with a damping constant of 100 fs maintained the target temperature. Minimisation used the LAMMPS conjugate-gradient algorithm with an energy tolerance of $10^{-4}$, a force tolerance of $10^{-6}$, and at most 100 iterations or 1000 force evaluations. The relaxed configuration produced by this stage served as the single initial state for all subsequent production runs. A small number of O_2_ molecules dissociated spontaneously during the 300 K equilibration (intact O_2_ counts at the production start: 98, 699, and 899 for the systems with *n*(O_2_) = 100, 700, and 900, respectively, together with a few free H and O atoms); no water molecules were present in any system, either in the initial configuration or at the production start.

**Figure 9:**
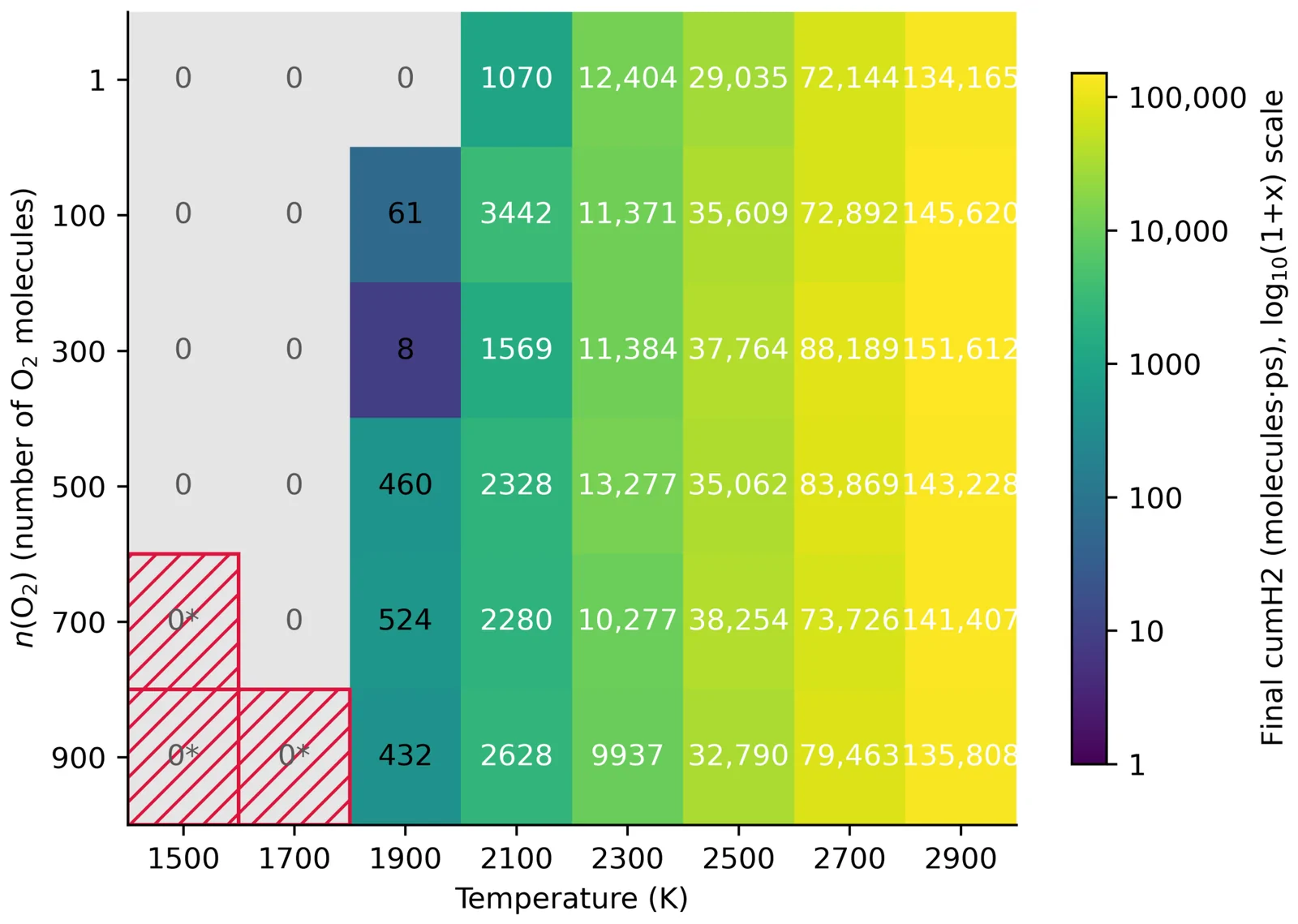
Simulation matrix of the 48 production trajectories: six O_2_ loadings (*n*(O_2_) = 1–900) at eight temperatures (1500–2900 K). Colour encodes the final cumulative H_2_ production cumH2 (molecules·ps; Section 3.2) on a $\log_{10}\left(1+x\right)$ scale with the value printed in each cell; grey “0” cells produced no H_2_ within 500 ps, and asterisked cells mark the three truncated runs (93.25, 11.0, and 183.5 ps) excluded from machine learning.

In the second (production) stage, the equilibrated configuration of each system was re-minimised briefly (same tolerances) and then propagated in NVT for $2\times 10^{6}$ timesteps (0.25 fs, 500 ps) at each of eight target temperatures: 1500, 1700, 1900, 2100, 2300, 2500, 2700 and 2900 K, spanning the kinetically accessible regime for PE oxidation and gasification in ReaxFF MD. The Nosé–Hoover damping constant was again 100 fs. Atomic velocities were carried over from the equilibrated configuration at every temperature, and no velocity re-initialisation or random seeding was performed at any stage, so the protocol is deterministic given the equilibrated initial state. Six distinct O_2_ loadings were simulated at every temperature, yielding a 6×8 matrix of 48 thermodynamic conditions (Figure 9). Three production runs terminated early (durations 93.25, 11.0, and 183.5 ps for *n*(O_2_) = 700 at 1500 K, *n*(O_2_) = 900 at 1500 K, and *n*(O_2_) = 900 at 1700 K, respectively); these truncated trajectories are reported for completeness but excluded from all machine-learning analyses (Section 3.2).

Thermodynamic data (temperature, pressure, and density) were written every 100 steps. Bond-order data and energy components were recorded every 100 steps, whereas Cartesian coordinates were saved every 1000 steps.

Because all production runs were performed at constant volume, the density of each system is fixed by its composition and ranges from 0.196 to 0.204 g/cm^3^ across the six systems (Table 7). The mean production temperature stayed within ±5% of the target in every run, and the time-averaged pressure varied with temperature and O_2_ loading from approximately 68 atm (the coldest, least-oxidised conditions) to approximately 2930 atm (the hottest condition of the *n*(O_2_) = 1 system); the full per-condition statistics are provided in the Supplementary Materials. These high-temperature, high-pressure NVT states should be interpreted as a designed scan of the kinetic accessibility of PE oxidation chemistry rather than as a representation of any single industrial process: industrial gasification operates near 650–900 K at residence times of milliseconds to seconds, whereas the simulations probe 1500–2900 K over 500 ps. Within this framing, the simulation temperature acts as a kinetic accelerator that shifts the relevant reaction timescales into the computable window. Absolute rates measured under these conditions should therefore not be transferred to process conditions, whereas relative trends across O_2_ loading and temperature are the quantities of interest. No claim is made regarding supercritical-water states: the simulated systems are PE–O_2_ mixtures that initially contain no water, and water appears only as a reaction product at the highest temperatures.

### 3.2. Dataset Construction

The 48 production trajectories were post-processed into tabular datasets suitable for supervised regression. Molecular identities were assigned from the bond-order logs written by fix reax/c/bonds every 100 steps: at each recorded step, the atoms were partitioned into molecules by connected-component analysis of the bond graph, using the ReaxFF bond-identification cutoff of 0.3 applied by our in-house Python (version 3.13) code for LAMMPS analysis. This yields time-resolved counts of small-molecule species such as H_2_, CH_4_, C_2_H_4_, C_2_H_2_, C_2_H_6_, CO and CO_2_. No transient-species filtering was applied: a molecule counts whenever the bond graph contains it at a recorded step, so a re-formed H_2_ molecule is counted again after dissociation and recombination.

Species rows are written on an event basis, that is, only when the molecular composition changes, so the sampling interval Δt varies within and between trajectories: the least reactive low-temperature runs contribute as few as 446 rows over 500 ps (the *n*(O_2_) = 300 system at 1500 K) with inter-frame gaps reaching 29 ps, whereas reactive high-temperature runs are sampled densely. Thermodynamic data (temperature, pressure, density, and the 14 ReaxFF energy components), written every 100 steps, were merged with each species row by nearest recorded step (agreement within 100 steps). Every row is indexed by the unique identifier of its parent simulation and its simulation time *t* in picoseconds.

Three data-hygiene rules were applied before any learning. First, duplicated rows, which arise because every simulation repeats its final-step row in the species log, were removed (76,865 raw rows → 76,817). Second, four unique rows carrying sentinel values (N_H2 =−1, an analyser artefact at the final step of four simulations) were removed (76,817 → 76,813). Third, the three truncated simulations identified in Section 3.1 were excluded from machine learning altogether, leaving 45 simulations with 76,398 rows. Finally, the initial thermal transient of each trajectory was removed: the warm-up time is the first time at which the measured temperature stays within ±5% of the target, where an out-of-band run of two or more consecutive rows resets the warm-up whereas isolated single-row excursions after the transient are treated as thermostat noise and ignored. The resulting warm-up durations are short (median 2.00 ps, mean 3.20 ps, maximum 17.0 ps), and 76,334 rows remain after warm-up removal. For four simulations, the robust rule differs from a strict literal reading of the ±5% criterion; the most consequential case is a single-row excursion 0.14 K above the band edge at t=199.0 ps in one 2900 K run, which the strict rule would penalise by discarding roughly 40% of that trajectory. The robust values are used throughout.

Because the species sampling is event-based with variable Δt, the regression target is defined as the *time-weighted* cumulative H_2_ production,

(2) $$\mathrm{cumH}2\left(t_{j}\right)\;=\;\sum_{s\leq j}N_{H2}\left(t_{s}\right)\;\Delta t_{s},\;\Delta t_{s}=t_{s+1}-t_{s},$$

in units of molecules·ps, where $N_{H_{2}}\left(t_{s}\right)$ is the instantaneous H_2_ count at recorded step *s* and the interval $\Delta t_{s}$ extends to the next recorded frame (Δt=0 at the final frame of each trajectory). In physical terms, one molecule·ps corresponds to a single H_2_ molecule persisting for one picosecond, so cumH2(t) is the area under the H_2_ population curve over [0,t], that is, the time-integrated amount of H_2_ present in the simulation box. The target is monotone non-decreasing by construction and integrates the H_2_ record over physical time so that densely and sparsely sampled trajectory segments contribute proportionally to their duration. A plain frame-count would instead weight each recorded state equally and conflate the sampling density with the production rate. Thirteen of the 48 trajectories produce no H_2_ at all within 500 ps (all simulations at 1500 and 1700 K, plus the *n*(O_2_) = 1 system at 1900 K); these zero-production trajectories are retained, and the corresponding matrix cells appear as grey zero entries in Figure 9.

The learning task is a fixed-horizon forecast: given the simulation state at an origin time $t_{i}$ (the feature row), predict $y=\mathrm{cumH}2(t_{j})$, where $t_{j}$ is the first recorded time with $t_{j}\geq t_{i}+h$ for a forecast horizon h∈{2.5,25,50} ps, with h=25 ps as the primary horizon. An origin is skipped if no such $t_{j}$ exists (the trajectory ends within *h*) or if the realised gap exceeds the tolerance $t_{j}-t_{i}>h+5$ ps, which protects against event-sparse low-temperature trajectories in which the next available frame is too distant. All features are taken strictly from the origin row *i*; no information from times later than $t_{i}$ enters the input, and the final analyser row of each trajectory can only ever serve as a forecast target, never as an origin. As a reference point, the persistence forecast $\mathrm{cumH}2(t_{i})$, which carries the target history forward unchanged, is stored alongside every pair as the baseline that any learned model must beat [39,41]. Table 8 summarises the hygiene chain and the number of forecast pairs constructed at each horizon.

Two candidate feature pools are defined on these pairs. Case A contains 48 candidate features: the instantaneous thermodynamic and chemical descriptors of the origin row (temperature, simulation time, pressure, density, the 14 ReaxFF energy components, instantaneous small-molecule counts, elemental and structural ratios, radical counts, rates of change, rolling statistics, and the water–gas shift equilibrium constant), together with the O_2_ loading, a per-simulation design constant, and ten features derived from the H_2_ record up to the origin time (the plain cumulative count cumulative_H2, one- and two-step lags N_H2_lag1 and N_H2_lag2, the finite differences dN_H2 and dN_H2_dt, the rolling statistics rolling_mean_H2 and rolling_std_H2, the selectivity and ratio measures H2_selectivity and H_C_ratio, and the running apparent activation energy running_Ea). Case B contains the remaining 38 candidates: the same instantaneous descriptors and the O_2_ loading, with all ten H_2_-derived features excluded. The instantaneous H_2_ count $N_{H_{2}}$ is an input feature in neither case, and the persistence value $\mathrm{cumH}2(t_{i})$ is used only as the baseline forecast, never as a model input.

### 3.3. Feature Selection

A six-stage feature-selection pipeline reduces the candidate pool of each case to the input set used by the regression models. The pipeline was implemented with scikit-learn utilities [53] and, most importantly, was re-run from scratch on the training rows of every cross-validation fold and every training partition (Section 3.5). Feature selection is itself a modelling decision: running it on the full dataset before splitting would leak evaluation information into the input set and overstate the reported performance [38,39,40].

The six stages are applied sequentially to the candidate pool of the given case:

1. A variance-threshold filter removes near-constant columns (variance <0.01);
2. A Pearson correlation filter discards one feature from every pair with |ρ|>0.8;
3. A mutual-information ranking against the target retains the top 50% of the remaining candidates;
4. Permutation importance scoring with a preliminary random forest [54] (10 repeats) retains the top 25 candidates;
5. Recursive feature elimination (RFE) with a random-forest estimator reduces the set to 15 candidates;
6. A TreeSHAP ranking with a preliminary XGBoost model [42,55] selects the final 12  input features.

The same pipeline is applied to Case A (48 candidates) and Case B (38 candidates). Case A admits the ten H_2_-derived history features of Section 3.2 into its candidate pool. Case B excludes them so that any Case B model must infer the H_2_ trajectory shape from instantaneous physical descriptors alone. The selection is data-driven throughout and is not constrained to retain any particular descriptor; the per-fold selected sets and the stage-wise survival of the temperature, the O_2_ loading, and the simulation time across folds are documented in the Supplementary Materials.

### 3.4. Machine Learning Models

Eight tabular regression models were trained on the forecast-pair datasets of both cases. All implementations used scikit-learn [53] except XGBoost [55] and CatBoost [56], which were called through their dedicated Python (version 3.13) interfaces. The suite spans the principal families of regression algorithms with one line of motivation per model: multiple linear regression (MLR) as a parameter-free linear reference; a single decision tree (DT) with axis-parallel splits; a random forest (RF) [54], a bagged ensemble of decorrelated trees; XGBoost, a regularised gradient-boosted tree ensemble; CatBoost, an ordered boosting variant designed to resist overfitting on small-to-moderate datasets; an artificial neural network (ANN, the scikit-learn MLPRegressor), a nonlinear function approximator trained by backpropagation; support vector regression (SVR) with an RBF kernel [57,58,59]; and Gaussian process regression (GPR) with a composite Matérn plus white-noise kernel [60], which additionally provides predictive uncertainty estimates. Bagging and boosting are each represented once because tree ensembles are consistently among the strongest performers on tabular scientific data [61].

Three baselines anchor the benchmark, starting with the persistence forecast (Section 3.2), which carries $\mathrm{cumH}2(t_{i})$ forward unchanged and is the minimal autoregressive reference that any learned model must beat. Two physics-informed baselines, an MLR and an RF restricted to the three design variables (temperature, O_2_ loading, and simulation time) quantify how much of the target is predictable from the design matrix alone without using any trajectory observables.

Feature scaling was applied only to the scale-sensitive models (SVR, ANN, and GPR) through a StandardScaler step wrapped with the regressor in a single Pipeline object; the scaler was fitted strictly within each training partition, never on evaluation data. The tree-based models and MLR were trained on the unscaled features.

Because the training cost of the kernel methods grows superlinearly with the number of training rows, their training partitions were subsampled: GPR was trained on approximately 2000 rows and SVR on approximately 5000 rows per training partition. The subsamples were group-balanced, so that every simulation in the partition remained represented in proportion to its row count, and temporally thinned so that consecutive near-duplicate rows were preferentially removed and the trajectory shapes were preserved. Five subsample seeds (42–46) were drawn per repeat, and the variability across subsamples is reported alongside the mean performance. The remaining six models used the full training partition, so the cross-model comparison mixes unequal effective training sizes for the two kernel methods; this is acknowledged in the interpretation of their results.

The hyperparameters of all eight models were carried over from the grids tuned in the authors’ previous study of these trajectories. The full tuning grids, the selected values for each case, and the software versions are documented verbatim in the Supplementary Materials. No hyperparameter was re-tuned on evaluation data in this work; within the nested protocol of Section 3.5, all tuning is confined to the inner folds of the training partition.

### 3.5. Evaluation Protocol

All accuracy estimates were produced by protocols that respect the simulation-group structure of the data with the simulation identifier as the grouping variable: every forecast pair of a given simulation always lies on the same side of any split [38,39]. Row-level random splits are never used, because rows sampled from one trajectory are strongly autocorrelated, and a random row split would place near-identical states on both sides of the split.

A single grouped train–test split (scikit-learn GroupShuffleSplit with test_size =0.2 and random_state =42) first reserved 9 of the 45 simulations as a held-out test partition, leaving 36 training simulations. The held-out partition was evaluated exactly once, after the feature-selection behaviour, the model choice, and all hyperparameters had been frozen on the training partition alone; the simulation identifiers of both partitions are listed in the Supplementary Materials.

Within the training partition, model development used a nested grouped cross-validation scheme. The outer loop is a 5-fold GroupKFold over the 36 training simulations. Within each outer training part, an inner 3-fold GroupKFold drives the feature selection of Section 3.3 and the hyperparameter selection from the fixed grids so that these modelling decisions are made without ever touching the corresponding outer-validation simulations [40]. With the hyperparameters frozen, the stability of the grouped estimates was quantified by five repeats of a shuffled grouped 5-fold cross-validation (StratifiedGroupKFold, shuffle = True, seeds 42–46), which was stratified by the discrete temperature label of each simulation (a design variable, not a binning of the target). All grouped-CV values are reported as the mean ± standard deviation across the five repeats.

Model accuracy was quantified with four complementary regression metrics. The coefficient of determination $R^{2}$ measures the fraction of variance explained,

(3) $$R^{2}\;=\;1\;-\;\frac{\sum_{i=1}^{n}{\left(y_{i}-{\hat{y}}_{i}\right)}^{2}}{\sum_{i=1}^{n}{\left(y_{i}-\bar{y}\right)}^{2}},$$

where $y_{i}$, ${\hat{y}}_{i}$ and $\bar{y}$ denote the observed, predicted and mean target values, respectively. The root mean square error (RMSE) penalises large deviations quadratically,

(4) $$\mathrm{RMSE}\;=\;\sqrt{\frac{1}{n}\sum_{i=1}^{n}{\left(y_{i}-{\hat{y}}_{i}\right)}^{2}}.$$

The mean absolute error (MAE) and mean absolute percentage error (MAPE) provide robust and scale-independent views of the same residual distribution,

(5) $$\mathrm{MAE}\;=\;\frac{1}{n}\sum_{i=1}^{n}\left|y_{i}-{\hat{y}}_{i}\right|,\;\mathrm{MAPE}\;=\;\frac{100}{n}\sum_{i=1}^{n}\frac{\left|y_{i}-{\hat{y}}_{i}\right|}{\max(\epsilon ,\;|y_{i}|)},$$

with a floor ϵ in the denominator of MAPE. MAPE is treated as a secondary metric in this work: 18.2% of the primary-horizon forecast pairs have y=0, originating from the genuine zero-H_2_ trajectories (Section 3.2). The floor is set to ϵ=1 molecule·ps, which suppresses the divergence of the percentage error on near-zero targets, but MAPE values on this subset are interpreted with care, and the zero-target pairs are retained throughout. RMSE and MAE carry the target units of molecules·ps.

To prevent long, densely sampled trajectories from dominating the aggregate statistics, metrics are computed in two ways, and both views are reported side by side: pooled over all forecast pairs of a partition and per simulation followed by an equally weighted mean over simulations. Forecast skill is additionally expressed relative to the persistence baseline as the percentage reduction in RMSE, $\Delta \mathrm{RMSE}=100\;\left({\mathrm{RMSE}}_{p}-{\mathrm{RMSE}}_{m}\right)/{\mathrm{RMSE}}_{p}$, and the corresponding difference in explained variance, $\Delta R^{2}=R_{m}^{2}-R_{p}^{2}$, where the subscripts m and p denote the model and the persistence forecast, respectively.

The Case A versus Case B comparison is a paired ablation: both cases are evaluated on identical group partitions and identical seeds so that the within-model differences isolate the predictive contribution of the ten H_2_-derived features under an otherwise unchanged evaluation protocol.

Two stress protocols probe generalisation beyond the sampled conditions. Leave-one-temperature-out (LOTO) cross-validation holds out all simulations of one temperature in turn (8 folds), and leave-one-O_2_-loading-out (LORO) cross-validation holds out all simulations of one O_2_ loading in turn (6 folds). In both protocols, the held-out simulations are absent from the feature-selection and tuning data entirely, so the corresponding scores measure generalisation to an unseen thermodynamic condition rather than to unseen time slices of already-seen simulations [38].

Model comparisons rely on descriptive statistics (mean ± standard deviation across the five repeated grouped cross-validation runs) rather than inferential rank tests: with five repeats per protocol, the distributional assumptions of such tests are difficult to justify, and the effect sizes of interest are visible directly in the grouped-CV spreads.

## 4. Conclusions

This study recast the monitoring of H_2_ production in ReaxFF MD simulations of polyethylene oxidative gasification as a leakage-free forecasting task: given the thermochemical state of a simulation at time *t*, predict the time-weighted cumulative H_2_ production 25 ps ahead. Eight tabular regression models were benchmarked against a persistence forecast and physics-informed baselines on 72,117 forecast pairs from 45 trajectories, under simulation-grouped nested cross-validation, a held-out test partition, temperature- and loading-holdout stress tests, and a paired ablation that removes all H_2_-derived inputs.

The value of machine learning for this task decomposes along a single axis: almost all of the attainable skill rests on the H_2_ history itself. With that history among the inputs, autoregressive forecasting is accurate and robust, and the near-linear regression baseline indicates that the learned mapping is a persistence forecast plus a correction, which is sharpened rather than reinvented by the nonlinear models. Without it, no machine-learning model beats the persistence forecast in cross-validation, on the held-out test partition, or in any structured holdout, under any of the five cross-validation repeats or subsampling seeds examined. A reduced physics-informed baseline rivals the full feature pool for the tree family, so the instantaneous thermochemical observables add little short-horizon signal once the H_2_ history is removed.

For process monitoring, wherever H_2_ production has been observed and is tracked, as in an analysis pipeline that post-processes ReaxFF trajectories, autoregressive tabular models provide accurate short-horizon forecasts. Where no H_2_ history exists, the persistence forecast is the robust default, and none of the tabular models studied here offers a better alternative. The results also argue for reporting persistence-relative skill as a mandatory baseline in learning studies of reactive MD trajectories, since conventional $R^{2}$ values can mask its absence.

Several limitations bound these conclusions, the first being that the benchmark quantifies interpolation within the analysed grid of temperatures and loadings and robustness under the structured holdouts, not extrapolation. The system scope is polyethylene and O_2_. Each condition is a single 500 ps trajectory, so the variance between replicate trajectories at fixed conditions remains unquantified. SHAP attributions identify associations rather than causal mechanisms. Wherever SHAP values are computed, the GPR and ANN are replaced by their best tree-based counterparts, which is a substitution documented in the results that affects no accuracy claim.

Future work will pursue sequence-learning architectures that build history representations internally so that forecasts no longer require an explicitly observed H_2_ history. Independent replica trajectories per condition would separate condition-level from trajectory-level variance. Extending the leakage-free forecasting protocol to further polymer feedstocks and mixed plastic waste streams would move the approach toward calibrated surrogates for reactor design and operating-condition optimisation.

## Figures and Tables

**Table 1:** Case A (with H_2_ history) pooled out-of-fold performance over the 36 training simulations under the nested grouped cross-validation protocol. The first-pass column reports the primary run; the remaining columns are the mean ± standard deviation over five stratified grouped 5-fold cross-validation repeats (seeds 42–46). RMSE and MAE are in molecules·ps; models are sorted by repeat-mean RMSE.

Model	First-Pass RMSE	RMSE (Mean ± SD)	MAE (Mean ± SD)
GPR	290.2	267.5±25.7	168.4±11.4
RF	305.1	302.5±3.7	166.1±0.7
DT	357.4	354.2±9.8	197.4±3.5
XGBoost	616.7	565.3±36.6	250.5±6.7
ANN	577.2	594.3±48.8	401.0±22.7
CatBoost	637.2	830.7±193.6	395.3±29.3
MLR	941.6	850.4±25.8	638.7±4.9
Physical RF	2540	2597±39	1377±12
Persistence	4096	4096±0	2468±0
SVR	19,702	18,508±369	7266±267
Physical MLR	18,905	19,041±75	14,420±32

**Table 2:** Case A (with H_2_ history) held-out test performance on the 9 reserved simulations (14,459 forecast pairs, h=25 ps). RMSE and MAE are in molecules·ps. ΔRMSE is the percentage RMSE reduction with respect to the persistence forecast, $\Delta \mathrm{RMSE}=100\;\left({\mathrm{RMSE}}_{p}-{\mathrm{RMSE}}_{m}\right)/{\mathrm{RMSE}}_{p}$ (Section 3.5); positive values indicate that the model improves on persistence. Models are sorted by RMSE.

Model	RMSE	MAE	ΔRMSE vs. Persistence (%)
RF	121.8	79.7	+93.0
GPR	158.6	109.2	+90.9
DT	183.0	106.8	+89.5
XGBoost	202.4	115.1	+88.4
CatBoost	262.4	160.5	+85.0
ANN	507.4	343.9	+70.9
MLR	638.2	509.3	+63.4
Physical RF	1198	631.3	+31.4
Persistence	1745	815.8	0
SVR	5413	2263	−210.2
Physical MLR	15,054	12,549	−762.6

**Table 3:** Case B (without H_2_ history) pooled out-of-fold performance over the 36 training simulations under the nested grouped cross-validation protocol. The first-pass column reports the primary run; the remaining columns are the mean ± standard deviation over five stratified grouped 5-fold cross-validation repeats (seeds 42–46). RMSE and MAE are in molecules·ps; models are sorted by repeat-mean RMSE. No ML model improves on the persistence baseline.

Model	First-Pass RMSE	RMSE (Mean ± SD)	MAE (Mean ± SD)
Persistence	4096	4096±0	2468±0
ANN	11,700	6756±1864	3142±662
Physical RF	9984	8934±480	4567±340
GPR	15,966	9005±1364	4657±516
CatBoost	15,507	16,346±1438	7813±697
XGBoost	15,308	16,539±1105	7960±557
RF	17,209	16,748±1530	8123±668
DT	21,369	18,911±1840	8963±470
Physical MLR	18,905	19,041±75	14,420±32
MLR	22,955	23,457±653	16,602±668
SVR	25,318	24,786±290	11,357±270

**Table 4:** Case B (without H_2_ history) held-out test performance on the 9 reserved simulations (14,459 forecast pairs, h=25 ps; same partition as Case A). RMSE and MAE are in molecules·ps. ΔRMSE is the percentage RMSE reduction with respect to the persistence forecast ($\Delta \mathrm{RMSE}=100\;\left({\mathrm{RMSE}}_{p}-{\mathrm{RMSE}}_{m}\right)/{\mathrm{RMSE}}_{p}$); negative values indicate that the model performs worse than persistence. Models are sorted by RMSE. The persistence baseline performs best; no ML model improves on it.

Model	RMSE	MAE	ΔRMSE vs. Persistence (%)
Persistence	1745	815.8	0
ANN	2199	1180	−26.0
GPR	2361	1485	−35.3
Physical RF	4566	2085	−161.6
XGBoost	5070	3135	−190.5
CatBoost	5205	2512	−198.2
RF	6061	3364	−247.3
SVR	8111	3917	−364.7
DT	9815	3011	−462.4
Physical MLR	15,054	12,549	−762.6
MLR	16,874	13,232	−866.9

**Table 5:** Paired Case A versus Case B ablation on identical folds. $\Delta {\mathrm{RMSE}}_{A|B}=100\;\left({\mathrm{RMSE}}_{B}-{\mathrm{RMSE}}_{A}\right)/{\mathrm{RMSE}}_{B}$ is the percentage of RMSE reduction attributable to the H_2_-history features (positive: Case A better), reported for the first-pass out-of-fold run, as the mean ± standard deviation over the five stratified repeats (seeds 42–46), and on the held-out test partition. RMSE values are in molecules·ps; models are sorted by the first-pass $\Delta {\mathrm{RMSE}}_{A|B}$. Persistence and the physics-informed MLR use no H_2_-derived input in either case and are identical by construction.

Model	Case A RMSE (First-Pass)	Case B RMSE (First-Pass)	ΔRMSE (%) First-Pass	ΔRMSE (%), 5 Repeats	ΔRMSE (%), Test
DT	357.4	21,369	+98.3	+98.1±0.1	+98.1
RF	305.1	17,209	+98.2	+98.2±0.1	+98.0
GPR	290.2	15,966	+98.2	+97.0±0.3	+93.3
XGBoost	616.7	15,308	+96.0	+96.6±0.2	+96.0
MLR	941.6	22,955	+95.9	+96.4±0.1	+96.2
CatBoost	637.2	15,507	+95.9	+94.8±1.6	+95.0
ANN	577.2	11,700	+95.1	+90.6±2.6	+76.9
Physical RF	2540	9984	+74.6	+70.9±1.7	+73.8
SVR	19,702	25,318	+22.2	+25.3±1.0	+33.3
Persistence	4096	4096	0	0	0
Physical MLR	18,905	18,905	0	0	0

**Table 7:** Composition of the six simulated PE–O_2_ systems. Each system contains 50 PE chains (C_30_H_62_: 1500 C and 3100 H atoms in total; PE mass 21,141.3 amu). Production densities are the mean densities over the production trajectories under constant-volume (NVT) conditions.

*n*(O_2_)	Total Atoms	Box Side (Å)	O_2_:PE Mass Ratio (%)	O_2_ per PE Chain	Production ρ (g/cm^3^)
1	4602	56.11	0.15	0.02	0.1998
100	4800	59.02	15.1	2	0.1964
300	5200	63.00	45.4	6	0.2038
500	5600	68.04	75.7	10	0.1957
700	6000	71.03	106.0	14	0.2016
900	6400	75.10	136.2	18	0.1961

**Table 8:** Data-hygiene chain and forecast-pair counts. “No future” skips are origins whose trajectory ends within the horizon; “tolerance” skips are origins whose next frame lies more than h+5 ps ahead. Zero-target pairs originate from the genuine zero-H_2_ trajectories and are retained.

Processing Stage	Rows	Simulations
Raw species tables	76,865	48
After removing duplicated rows	76,817	48
After removing sentinel rows	76,813	48
ML dataset (truncated excluded)	76,398	45
After removing warm-up rows	76,334	45
*Forecast pairs constructed per horizon*		
h=2.5 ps	75,725	45
h=25 ps (primary)	72,117	45
h=50 ps	68,122	45

## Data Availability

The datasets generated and/or analysed during the current study are available from the corresponding author on reasonable request.

## Supplementary Materials

The following supporting information can be downloaded at https://www.mdpi.com/article/10.3390/ijms27219435/s1.
