# Supplementary material for: Interpretable Machine Learning of Hydrogen Production from Reactive Molecular Dynamics Simulations of Polyethylene Oxidative Gasification
Source: Int J Mol Sci. 2026 Oct 22;27(21):9435. doi: 10.3390/ijms27219435 (PMC13762597; doi:10.3390/ijms27219435)
Supplement: Supplementary file 1 [file ijms-27-09435-s001.zip › ijms-4548269-supplementary.pdf]

Supporting Information:

Interpretable Machine Learning of Hydrogen Production from Reactive Molecular Dynamics Simulations of Polyethylene Oxidative Gasification

Thi Be Ta Truong [1] and Thuat T. Trinh [2,*]

1 Group of Applied Research in Advanced Materials for Sustainable Development, Faculty of Applied Sciences, Ton Duc Thang University, Ho Chi Minh City 756636, Vietnam; truongthibeta@tdtu.edu.vn
2 Porelab, Department of Chemistry and Biomedical Science, Norwegian University of Science and Technology, NTNU, NO-7491 Trondheim, Norway
* Correspondence: thuat.trinh@ntnu.no

This Supporting Information provides (S1) the verbatim LAMMPS/ReaxFF inputs and control files used for the molecular dynamics simulations together with a basic Packmol input script for the initial configurations, (S2) the composition of the six simulated systems, (S3) the per-trajectory statistics of all 48 production runs, (S4) the construction of the machine-learning dataset and the train/test partition, (S5) the full feature-selection record, (S6) the hyperparameter grids and selected values, (S7) the per-simulation model metrics together with the stability figures referenced in the main text (Figures S1–S3), (S8) the settings of the SHAP analyses together with the dependence plots and interaction heatmaps referenced in the main text (Figures S4–S7), and (S9) the software versions.

S1. ReaxFF simulation setup

All 48 trajectories were produced with the LAMMPS molecular dynamics package (version 23 June 2022) using the ReaxFF reactive force field as implemented in the `reax/c` pair style of LAMMPS. Each of the six systems `f-50-1`, `f-50-100`, `f-50-300`, `f-50-500`, `f-50-700`, and `f-50-900` (50 polyethylene chains per system; $n(O_2)$ = 1, 100, 300, 500, 700, and 900 O_2 molecules; Table S1) was simulated at eight target temperatures (1500, 1700, 1900, 2100, 2300, 2500, 2700, and 2900 K), giving $6 \times 8 = 48$ production runs. The workflow per system and temperature consists of (i) an initial configuration built with Packmol, a standard program for packing molecular systems, invoked by our in-house Python building script (a basic Packmol input script is reproduced in Listing S1), (ii) a 100 ps NVT equilibration at 300 K (`ineq.run`, Listing S3), which reads the Packmol configuration `data.lmp` and writes the production starting configuration `data2.lmp`, and (iii) the 500 ps NVT production run at the target temperature (`in.run`, Listing S2), which reads `data2.lmp`.

```
# Basic Packmol input script (example: the f-50-100 system)

tolerance 2.0
filetype xyz
output data.xyz

# 50 PE chains (C30H62)
structure pe.xyz
  number 50
  inside box 0. 0. 0.  59.02 59.1 59.016
end structure

# 100 O2 molecules
structure o2.xyz
  number 100
  inside box 0. 0. 0.  59.02 59.1 59.016
end structure
```

Listing S1: Basic Packmol input script for building an initial configuration (example: the `f-50-100` system; cell dimensions as in Table S1). The `pe.xyz` and `o2.xyz` structure files are written, and Packmol 20.14.0 is invoked, by the in-house Python building script.

Listing S2 reproduces the production input verbatim. It is a run template: the `myT` placeholder in line 3 takes the target temperature of each run; apart from this substitution the same input is used for every temperature. The input (i) declares `units real` and `atom_style charge` and reads `data2.lmp`; (ii) activates `pair_style reax/c control.reax_c` with `pair_coeff * * ffield C H O`, i.e., the C/H/O element subset of the force-field file is used; (iii) computes the 14 ReaxFF energy components (`compute reax all pair reax/c`); (iv) minimizes the energy with charge equilibration (`fix qeq/reax` at every step plus `minimize 1.0e-4 1.0e-6 100 1000`) and then removes the minimization fix; (v) integrates NVT dynamics at the target temperature with a Nosé–Hoover thermostat (damping constant `${rate}` = 100 in `real`-unit time, i.e., fs), with charge equilibration re-applied at every step (`fix 2 all qeq/reax 1 0.0 10.0 1.0e-6 reax/c`); (vi) writes bond information every 100 steps (`fix reax/c/bonds 100 bonds.reaxff`); (vii) logs thermo output every 100 steps (step, temperature, density, pair and total energies, pressure, and the 14 ReaxFF energy components); (viii) uses a time step of 0.25 fs; (ix) writes an xyz trajectory every 1000 steps (250 fs) to `trj.xyz`; (x) runs 2,000,000 steps (500 ps); and (xi) writes the final configuration to `datanvt.lmp`. Three runs terminated before the requested 500 ps (Section S3). The header comments of these files ("ReaxFF potential for RDX system") are inherited from the LAMMPS ReaxFF example (`reax/in.reax.rdx`) from which the workflow was adapted; they have no effect on the calculation.

Across the six loading directories the inputs are byte-identical, with a single exception: line 11 of `f-50-500/in.run` reads `pair_style reax/c control.reax_c safezone 1.6 mincap 100`, i.e., the safe-zone/minimum-capacity neighbor-list options of the `reax/c` implementation are enabled for the $n(O_2)$ = 500 system, whereas all other systems use

the bare `pair_style reax/c control.reax_c`. The files `ineq.run`, `control.reax_c`, and `ffield` are byte-identical in all six directories.

Because the 300 K equilibration precedes the production run, the O_2 inventory at the start of production differs slightly from the initial packing for some loadings: the equilibrated configurations contain 1, 98, 300, 500, 699, and 899 intact O_2 pairs for $n(O_2) = 1$, 100, 300, 500, 700, and 900, respectively (the remaining O atoms are bound in other species; no free O atoms are present at the production start; Table S1).

The control file (Listing S4) fixes the `reax/c` numerical parameters: long-range tabulation with 10,000 points, a neighbor-list cutoff of 4.5 Å, a hydrogen-bond cutoff of 6.0 Å, a bond-graph cutoff of 0.3, a three-body cutoff of 0.001, and a ReaxFF output frequency of 100 steps (atom, bond, and angle information; `simulation_name RDX_example` is another legacy string from the original example and is not used). The force-field file (Listing S5) identifies itself in its header line as "CHON-2017_weak reparametrized for bond-boost", carrying the DOI 10.1021/acs.jpca.8b03826 of the study for which the reparametrization was made; the production runs use its C/H/O subset. The initial (`data.lmp`) and equilibrated (`data2.lmp`) configuration files are not reproduced in this document; the compositions of the production configurations are listed in Section S2.

```
# ReaxFF potential for RDX system
# this run is equivalent to reax/in.reax.rdx
variable T equal myT
variable rate equal 100.0

units           real

atom_style      charge
read_data       data2.lmp
#replicate 3 2 2
pair_style      reax/c control.reax_c
#pair_coeff      * * ffield O C H N
#pair_coeff      * * ffield O C H
pair_coeff      * * ffield C H O

```

```
compute reax all pair reax/c

variable eb      equal c_reax[1]
variable ea      equal c_reax[2]
variable elp     equal c_reax[3]
variable emol    equal c_reax[4]
variable ev      equal c_reax[5]
variable epen    equal c_reax[6]
variable ecoa    equal c_reax[7]
variable ehb     equal c_reax[8]
variable et      equal c_reax[9]
variable eco     equal c_reax[10]
variable ew      equal c_reax[11]
variable ep      equal c_reax[12]
variable efi     equal c_reax[13]
variable eqeq    equal c_reax[14]

neighbor        2.5 bin
neigh_modify    every 10 delay 0 check no

fix             fqeq all qeq/reax 1 0.0 10.0 1.0e-6 reax/c
minimize 1.0e-4 1.0e-6 100 1000
unfix fqeq

##fix           1 all nve
#fix 1 all nvt temp $T $T 100.0
fix 1 all nvt temp $T $T ${rate}
fix             2 all qeq/reax 1 0.0 10.0 1.0e-6 reax/c
#fix 3 all reaxff/species 10 10 100 species.out
fix 4 all reax/c/bonds 100 bonds.reaxff

thermo          100
thermo_style    custom step temp density epair etotal press &
                v_eb v_ea v_elp v_emol v_ev v_epen v_ecoa &
                v_ehb v_et v_eco v_ew v_ep v_efi v_eqeq

timestep        0.25

#dump           1 all atom 10 dump.reaxc.rdx
```

```

#dump           2 all image 25 image.*.jpg type type &
#               axes yes 0.8 0.02 view 60 -30
#dump_modify    2 pad 3

#dump           3 all movie 25 movie.mpg type type &
#               axes yes 0.8 0.02 view 60 -30
#dump_modify    3 pad 3
dump 1 all xyz 1000 trj.xyz
#dump_modify      1 element O C H N
dump_modify      1 element  C H O
run             2000000

write_data datanvt.lmp
```

Listing S2: Production run input `in.run` (run template; the `myT` placeholder in line 3 takes the run temperature). Byte-identical in all six loading directories except for line 11 of the `f-50-500` copy (see text).

```
1 # ReaxFF potential for RDX system
2 # this run is equivalent to reax/in.reax.rdx
3 variable T equal 300.0
4 variable rate equal 100.0
5
6 units           real
7
8 atom_style      charge
9 read_data       data.lmp
10 #replicate 3 2 2
11 pair_style      reax/c control.reax_c
12 #pair_coeff      * * ffield O C H N
13 #pair_coeff      * * ffield O C H
14 pair_coeff      * * ffield C H O
15
16
17
18 compute reax all pair reax/c
19
20 variable eb      equal c_reax[1]
21 variable ea      equal c_reax[2]
22 variable elp     equal c_reax[3]
```

```
variable emol    equal c_reax[4]
variable ev      equal c_reax[5]
variable epen    equal c_reax[6]
variable ecoa    equal c_reax[7]
variable ehb     equal c_reax[8]
variable et      equal c_reax[9]
variable eco     equal c_reax[10]
variable ew      equal c_reax[11]
variable ep      equal c_reax[12]
variable efi     equal c_reax[13]
variable eqeq    equal c_reax[14]

neighbor        2.5 bin
neigh_modify    every 10 delay 0 check no

fix             fqeq all qeq/reax 1 0.0 10.0 1.0e-6 reax/c
minimize 1.0e-4 1.0e-6 100 1000
unfix fqeq

##fix           1 all nve
#fix 1 all nvt temp $T $T 100.0
fix 1 all nvt temp $T $T ${rate}
fix             2 all qeq/reax 1 0.0 10.0 1.0e-6 reax/c
#fix 3 all reaxff/species 10 10 100 species.out
fix 4 all reax/c/bonds 100 bonds.reaxff

thermo          100
thermo_style    custom step temp density epair etotal press &
                v_eb v_ea v_elp v_emol v_ev v_epen v_ecoa &
                v_ehb v_et v_eco v_ew v_ep v_efi v_eqeq

timestep        0.25

#dump           1 all atom 10 dump.reaxc.rdx

#dump           2 all image 25 image.*.jpg type type &
#               axes yes 0.8 0.02 view 60 -30
#dump_modify    2 pad 3

#dump           3 all movie 25 movie.mpg type type &
#               axes yes 0.8 0.02 view 60 -30
```

```
#dump_modify    3 pad 3
dump 1 all xyz 1000 trj.xyz
#dump_modify      1 element O C H N
dump_modify      1 element  C H O
run             400000

write_data data2.lmp
```

Listing S3: Equilibration input `ineq.run` (300 K NVT, 400,000 steps = 100 ps; reads `data.lmp`, writes `data2.lmp`). Byte-identical in all six loading directories.

```
simulation_name         RDX_example ! output files will carry this name + their specific ext
tabulate_long_range     10000 ! denotes the granularity of long range tabulation, 0 means no tabulation
energy_update_freq      1

nbrhood_cutoff          4.5  ! near neighbors cutoff for bond calculations in A
hbond_cutoff            6.0  ! cutoff distance for hydrogen bond interactions
bond_graph_cutoff       0.3  ! bond strength cutoff for bond graphs
thb_cutoff              0.001 ! cutoff value for three body interactions

write_freq              100    ! write trajectory after so many steps
traj_title              RDX ! (no white spaces)
atom_info               1    ! 0: no atom info, 1: print basic atom info in the trajectory file
atom_forces             1    ! 0: basic atom format, 1: print force on each atom in the trajectory file
atom_velocities         0    ! 0: basic atom format, 1: print the velocity of each atom in the trajectory file
bond_info               1    ! 0: do not print bonds, 1: print bonds in the trajectory file
angle_info              1    ! 0: do not print angles, 1: print angles in the trajectory file
```

Listing S4: ReaxFF control file `control.reax_c`. Byte-identical in all six loading directories.

```
  CHON-2017_weak reparametrized for bond-boost DOI: 10.1021/acs.jpca.8b03826
 41        ! Number of general parameters
   50.0000 !Overcoordination parameter
    9.5469 !Overcoordination parameter
    1.6725 !Valency angle conjugation parameter
    1.7224 !Triple bond stabilisation parameter
    6.8702 !Triple bond stabilisation parameter
   60.4850 !C2-correction
    1.0588 !Povun6 Undercoordination Eq(12)
    4.6000 !Triple bond stabilisation parameter
   12.1176 !Povun7 Undercoordination Eq(12)
```

```
  13.3056 !Povun8 Undercoordination Eq(12)
 -20.0000 !Triple bond stabilization energy
   0.0000 !Lower Taper-radius
  10.0000 !Upper Taper-radius
   2.8793 !Fe dimer correction
  33.8667 !Valency undercoordination
   6.0891 !Plp1 Lone pair param in Eq(8)
   1.0563 !Valency angle
   2.0384 !Valency angle parameter
   6.1431 !Fe dimer correction
   6.9290 !Double bond/angle parameter
   0.3989 !Double bond/angle parameter: overcoord
   3.9954 !Double bond/angle parameter: overcoord
  -2.4837 !Fe dimer correction
   5.7796 !Torsion/BO parameter
  10.0000 !Torsion overcoordination
   1.9487 !Torsion overcoordination
  -1.2327 !Reserved
   2.1645 !Conjugation
   1.5591 !vdWaals shielding
   0.1000 !Cutoff for bond order (*100)
   1.7602 !Valency angle conjugation parameter
   0.6991 !Povun4 Over/Undercoordination Eq(11b)
  50.0000 !Povun3 Over/Undercoordination Eq(11b)
   1.8512 !Valency/lone pair parameter
   0.5000 !ACKS2 softness parameter
  20.0000 !Scale factor (d) in dispersion (LJ)
   5.0000 !Gauss exponent for explicit electrons
   0.0000 !1: disable undecoord term in val angle
   0.7903 !Valency angle conjugation parameter
   0.0000 !1: triple bond stabilization for all bonds
   0.0000 !eReax taper radius for electrons and holes
11    ! Nr of atoms; cov.r; valency;a.m;Rvdw;Evdw;gammaEEM;cov.r2;#
           alfa;gammavdW;valency;Eunder;Eover;chiEEM;etaEEM;n.u.
           cov r3;Elp;Heat inc.;bo131;bo132;bo133;softcut;n.u.
           ov/un;val1;n.u.;val3,vval4
C    1.3727   4.0000  12.0000   2.0270   0.1113   0.5516   1.1706   4.0000
     9.2293   4.5389   4.0000  30.0000  79.5548   4.4087   7.0601   0.0000
     1.1168   0.0000 181.0000  14.5210  24.9431   6.7313   0.8563   0.0000
    -6.7437   5.6329   1.0564   4.0000   2.9663   0.0000   0.0000   0.0000
H    0.8924   1.0000   1.0080   1.6791   0.0709   0.7390  -0.1000   1.0000
```

```
     8.3519  39.1732   1.0000   0.0000 121.1250   3.5442   9.3848   1.0000
    -0.1000   0.0000  61.6606   2.8222   2.1441   0.0003   1.0698   0.0000
   -18.1423   5.3143   1.0338   1.0000   2.8793   0.0000   0.0000   0.0000
 O   1.2450   2.0000  15.9990   2.3396   0.1000   1.1000   1.0548   6.0000
     9.3187  12.5083   4.0000  37.5000 116.0768   8.5000   8.4783   2.0000
     0.9049   0.1000  59.0626   3.4340   0.7722   0.0021   0.9745   0.0000
    -3.5352   3.2703   1.0493   4.0000   2.9225   0.0000   0.0000   0.0000
 N   1.2333   3.0000  14.0000   1.9690   0.1513   1.0000   1.1748   5.0000
     9.6326   9.1048   4.0000  32.4838 100.0000   6.6335   7.1473   2.0000
     1.0433   2.3175 119.9837   0.4648   9.7074   2.2521   0.9745   0.0000
    -7.0000   4.0000   1.0183   4.0000   2.8793   0.0000   0.0000   0.0000
 S   1.9673   2.0000  32.0600   2.1729   0.3000   1.0336   1.5359   6.0000
    10.3008   4.9055   4.0000  52.9998 112.1416   6.5000   8.2545   2.0000
     1.4601   9.7177  71.1843   5.7487  23.2859  12.7147   0.9745   0.0000
   -11.0000   2.7466   1.0338   6.2998   2.8793   0.0000   0.0000   0.0000
 Mg  1.8315   2.0000  24.3050   2.2464   0.1806   0.5020   1.0000   2.0000
    10.9186  27.1205   3.0000  38.0000   0.0000   0.9499   5.6130   0.0000
    -1.3000   0.0000 220.0000  49.9248   0.3370   0.0000   0.0000   0.0000
    -1.0823   2.3663   1.0564   6.0000   2.9663   0.0000   0.0000   0.0000
 P   1.5994   3.0000  30.9738   1.7000   0.1743   1.0000   1.3000   5.0000
     9.1909  12.0377   5.0000   0.0000   0.0000   1.7681   6.9748   0.0000
    -1.0000  13.1397 125.6300   0.2526  16.9194  24.7770   0.0000   0.0000
    -9.7896   3.3929   1.0338   5.0000   2.8793   0.0000   0.0000   0.0000
 Na  2.0300   1.0000  22.9898   2.3334   0.1481   0.8765  -1.0000   1.0000
    11.0000   9.8000   1.0000   0.0000   0.0000  -3.8501   5.9459   0.0000
    -1.0000   0.0000  67.5458 100.0000  10.0000   0.2500   0.8563   0.0000
    -2.5766   2.5000   1.0338   6.0000   2.5791   0.0000   0.0000   0.0000
 Cu  1.9202   2.0000  63.5460   1.9221   0.2826   1.0000   0.1000   1.0000
    10.9889 100.0000   1.0000   0.0000   0.0000   2.7875   6.0000   0.0000
    -1.0000   0.0000  80.7000  34.9555   0.4988   0.0000   0.8563   0.0000
    -5.1872   3.1491   1.0000   4.0000   2.5791   0.0000   0.0000   0.0000
 Cl  1.7140   1.0000  35.4500   1.9139   0.2000   0.3837  -1.0000   7.0000
    11.5345  10.1330   1.0000   0.0000   0.0000   9.9614   6.5316   0.0000
    -1.0000   3.5750 143.1770   6.2293   5.2294   0.1542   0.8563   0.0000
   -10.2080   2.9867   1.0338   6.2998   2.5791   0.0000   0.0000   0.0000
 X  -0.0998   2.0000   1.0080   2.0000   0.0000   1.0000  -0.1000   6.0000
    10.0000   2.5000   4.0000   0.0000   0.0000   8.5000   1.5000   0.0000
    -0.1000   0.0000  -2.3700   8.7410  13.3640   0.6690   0.9745   0.0000
   -11.0000   2.7466   1.0338   4.0000   2.8793   0.0000   0.0000   0.0000
 43    ! Nr of bonds; Edis1;LPpen;n.u.;pbe1;pbo5;13corr;pbo6
                         pbe2;pbo3;pbo4;n.u.;pbo1;pbo2;ovcorr
```

1	1	91.8588	120.2415	50.6788	0.3723	-0.1000	1.0000	34.6637	0.7015
		6.8558	-0.1605	7.9139	1.0000	-0.0719	6.9788	1.0000	0.0000
1	2	185.0817	0.0000	0.0000	-0.4949	0.0000	1.0000	6.0000	0.6864
		8.2839	1.0000	0.0000	1.0000	-0.0847	6.4150	0.0000	0.0000
2	2	165.6186	0.0000	0.0000	-0.4194	0.0000	1.0000	6.0000	0.6197
		6.3245	1.0000	0.0000	1.0000	-0.0879	6.0634	0.0000	0.0000
1	3	148.9918	115.2723	93.5449	-0.6834	-0.3639	1.0000	18.9178	0.6790
		0.4084	-0.4385	7.7418	1.0000	-0.1798	4.9342	0.0000	0.0000
2	3	159.4722	0.0000	0.0000	-0.5725	0.0000	1.0000	6.0000	0.5505
		1.1150	1.0000	0.0000	0.0000	-0.0920	4.2790	0.0000	0.0000
3	3	142.2858	85.0000	0.0000	0.2506	-0.1000	1.0000	29.7503	0.6051
		0.3451	-0.1055	9.0000	1.0000	-0.1225	5.5000	1.0000	0.0000
1	4	186.9647	90.5539	69.0633	-1.7565	-0.1960	1.0000	28.5338	0.1967
		0.0446	-0.3644	6.5414	1.0000	-0.2803	4.6293	1.0000	0.0000
3	4	128.8596	167.8643	40.0000	0.3819	-0.1539	1.0000	34.9972	0.1900
		1.0110	-0.3716	7.0805	1.0000	-0.1265	6.8843	1.0000	0.0000
4	4	160.1592	82.5526	153.9884	0.4110	-0.0934	1.0000	12.4304	0.5899
		0.1538	-0.1473	11.9187	1.0000	-0.0753	5.4371	1.0000	0.0000
2	4	173.4757	0.0000	0.0000	-0.2904	0.0000	1.0000	6.0000	0.6709
		0.5347	1.0000	0.0000	1.0000	-0.1855	5.2649	0.0000	0.0000
1	5	150.8132	59.3363	55.2528	-0.0628	-0.5211	1.0000	18.9617	0.3219
		0.3317	-0.2289	7.5946	1.0000	-0.1946	5.9455	1.0000	0.0000
2	5	143.4377	0.0000	0.0000	-0.2944	0.0000	1.0000	6.0000	0.6034
		9.5627	1.0000	0.0000	1.0000	-0.0516	7.0960	1.0000	0.0000
3	5	0.0000	0.0000	0.0000	0.5563	-0.4038	1.0000	49.5611	0.6000
		0.4259	-0.4577	12.7569	1.0000	-0.1100	7.1145	1.0000	0.0000
4	5	0.0000	0.0000	0.0000	0.4438	-0.2034	1.0000	40.3399	0.6000
		0.3296	-0.3153	9.1227	1.0000	-0.1805	5.6864	1.0000	0.0000
5	5	140.8887	84.9350	68.6860	-0.4111	-0.4781	1.0000	17.8574	-0.1336
		0.2881	-0.2494	9.8436	1.0000	-0.1806	7.4732	1.0000	0.0000
2	6	58.6896	0.0000	0.0000	-0.0203	-0.1418	1.0000	13.1260	0.0230
		8.2136	-0.1310	0.0000	1.0000	-0.2692	6.4254	0.0000	24.4461
3	6	87.0227	0.0000	43.3991	0.0030	-0.3000	1.0000	36.0000	0.0250
		0.0087	-0.2500	12.0000	1.0000	-0.0439	6.6073	1.0000	24.4461
6	6	32.3808	0.0000	0.0000	-0.0076	-0.2000	0.0000	16.0000	0.2641
		4.8726	-0.2000	10.0000	1.0000	-0.0729	4.6319	0.0000	0.0000
1	7	0.0000	0.0000	0.0000	0.2171	-0.1418	1.0000	13.1260	0.6000
		0.3601	-0.2500	20.0000	1.0000	-0.2000	10.0000	1.0000	0.0000
2	7	0.0000	0.0000	0.0000	0.2250	-0.1418	1.0000	13.1260	0.6000
		0.3912	-0.1310	0.0000	1.0000	-0.2000	10.0000	0.0000	0.0000
3	7	250.0000	182.8454	0.0000	0.0607	-0.5000	1.0000	25.0000	0.4547

		0.7012	-0.2197	15.9047	1.0000	-0.2026	6.4270	1.0000	0.0000
4	7	130.0000	0.0000	0.0000	0.2171	-0.1418	1.0000	13.1260	0.6000
		0.3601	-0.1310	10.7257	1.0000	-0.0869	5.3302	1.0000	0.0000
6	7	0.1000	0.0000	0.0000	0.2500	-0.5000	1.0000	35.0000	0.6000
		0.5000	-0.5000	20.0000	1.0000	-0.2000	10.0000	1.0000	0.0000
7	7	0.0000	0.0000	0.0000	0.2171	-0.5000	1.0000	35.0000	0.6000
		0.5000	-0.5000	20.0000	1.0000	-0.2000	10.0000	1.0000	0.0000
2	8	0.0000	0.0000	0.0000	-1.0000	-0.3000	1.0000	36.0000	0.7000
		10.1151	-0.3500	25.0000	1.0000	-0.1053	8.2003	1.0000	0.0000
3	8	76.0753	0.0000	0.0000	-0.4452	-0.3000	1.0000	36.0000	0.6433
		5.6834	-0.3500	25.0000	1.0000	-0.0539	8.0273	1.0000	0.0000
4	8	0.0000	0.0000	0.0000	-1.0000	-0.3000	1.0000	36.0000	0.7000
		10.1151	-0.3500	25.0000	1.0000	-0.1053	8.2003	1.0000	0.0000
6	8	0.1000	0.0000	0.0000	0.2500	-0.5000	1.0000	35.0000	0.6000
		0.5000	-0.5000	20.0000	1.0000	-0.2000	10.0000	1.0000	0.0000
7	8	0.1000	0.0000	0.0000	0.2500	-0.5000	1.0000	35.0000	0.6000
		0.5000	-0.5000	20.0000	1.0000	-0.2000	10.0000	1.0000	0.0000
8	8	27.8052	0.0000	0.0000	0.4022	0.3000	0.0000	25.0000	0.4894
		0.6222	-0.4000	12.0000	1.0000	-0.0500	5.3362	0.0000	0.0000
4	6	0.0000	0.0000	0.0000	-1.0000	-0.3000	1.0000	36.0000	0.7000
		10.1151	-0.3500	25.0000	1.0000	-0.1053	8.2003	1.0000	0.0000
1	9	0.0000	0.0000	0.0000	0.2000	-0.1418	1.0000	13.1260	0.5000
		0.5000	-0.2000	20.0000	1.0000	-0.1000	9.0000	0.0000	0.0000
2	9	0.0000	0.0000	0.0000	0.2000	-0.1418	1.0000	13.1260	0.5000
		0.5000	-0.2000	20.0000	1.0000	-0.1000	9.0000	0.0000	0.0000
3	9	81.4346	0.0000	0.0000	-0.1594	-0.3000	1.0000	36.0000	0.0025
		0.2904	-0.2500	12.0000	1.0000	-0.0742	9.3638	0.0000	0.0000
4	9	96.5322	0.0000	0.0000	0.9970	-0.3000	1.0000	36.0000	0.5095
		0.7247	-0.2500	12.0000	1.0000	-0.1175	9.9985	0.0000	0.0000
9	9	73.6263	0.0000	0.0000	0.0209	-0.2000	0.0000	16.0000	0.3414
		0.4703	-0.2000	15.0000	1.0000	-0.1319	5.9254	0.0000	0.0000
2	10	109.1686	0.0000	0.0000	-0.1657	-0.2000	0.0000	16.0000	1.2500
		2.8463	-0.2000	15.0000	1.0000	-0.1111	5.2687	0.0000	0.0000
3	10	0.0000	0.0000	0.0000	0.5000	-0.2000	0.0000	16.0000	0.5000
		1.0001	-0.2000	15.0000	1.0000	-0.1000	10.0000	0.0000	0.0000
9	10	118.3052	0.0000	0.0000	-0.1168	-0.2000	0.0000	16.0000	0.0697
		2.9176	-0.2000	15.0000	1.0000	-0.1316	5.3624	0.0000	0.0000
10	10	0.2500	0.0000	0.0000	0.1803	-0.2000	0.0000	16.0000	0.3356
		0.9228	-0.2000	15.0000	1.0000	-0.1178	5.6715	0.0000	0.0000
1	8	0.0000	0.0000	0.0000	-1.0000	-0.3000	1.0000	36.0000	0.7000
		10.1151	-0.3500	25.0000	1.0000	-0.1053	8.2003	1.0000	0.0000

```
  1 10   0.0000   0.0000   0.0000   0.5000  -0.2000   0.0000  16.0000   0.5000
         1.0001  -0.2000  15.0000   1.0000  -0.1000  10.0000   0.0000   0.0000
  4 10   0.0000   0.0000   0.0000   0.5000  -0.2000   0.0000  16.0000   0.5000
         1.0001  -0.2000  15.0000   1.0000  -0.1000  10.0000   0.0000   0.0000
 24    ! Nr of off-diagonal terms; Ediss;Ro;gamma;rsigma;rpi;rpi2
  1  2   0.1163   1.3831   9.9402   1.1471  -1.0000  -1.0000
  1  3   0.0729   2.0641   9.6888   1.4359   1.1638   1.0702
  2  3   0.0283   1.2853  10.9173   0.9259  -1.0000  -1.0000
  1  4   0.1268   1.9685   9.7237   1.3409   1.2578   1.1715
  2  4   0.1140   1.4837   9.0464   1.0812  -1.0000  -1.0000
  3  4   0.2000   1.8111   9.3860   1.4308   1.0787   1.2599
  1  5   0.1618   1.7943  10.1042   1.7489   1.3150   1.4031
  2  5   0.0764   1.5838  10.1462   1.4206  -1.0000  -1.0000
  3  5   0.1022   1.9887  10.0605   1.5799   1.4000  -1.0000
  4  5   0.1505   1.9000  10.5104   1.8000   1.4000  -1.0000
  2  6   0.0100   1.6000  13.2979   1.8670  -1.0000  -1.0000
  3  6   0.0809   1.7000  11.4606   1.5177  -1.0000  -1.0000
  1  7   0.2291   1.7500  10.0393  -1.0000  -1.0000  -1.0000
  2  7   0.3000   1.9262  10.0302  -1.0000  -1.0000  -1.0000
  3  7   0.1573   1.8644  10.1395   1.7435   1.4412  -1.0000
  6  7   0.1801   1.8566   9.8498   0.1000  -1.0000  -1.0000
  3  8   0.1592   1.8283  11.7256   1.6655  -1.0000  -1.0000
  1  9   0.0500   1.7500  12.3500   0.1000  -1.0000  -1.0000
  2  9   0.0300   1.5200  12.5000   0.1000  -1.0000  -1.0000
  3  9   0.0348   1.7637  12.3562   1.7228  -1.0000  -1.0000
  4  9   0.0478   1.7704  12.8051   1.6100  -1.0000  -1.0000
  2 10   0.0568   1.6740   9.6297   1.2200  -1.0000  -1.0000
  3 10   0.1927   2.2551  11.2308  -1.0000  -1.0000  -1.0000
  9 10   0.1402   2.1604  10.9786   1.7505  -1.0000  -1.0000
105    ! Nr of angles;at1;at2;at3;Thetao,o;ka;kb;pv1;pv2
  1  1  1  66.0347  45.0000   1.1248   0.0000   3.0000  70.0000   2.4142
  1  1  2  58.9292  15.1099   4.4631   0.0000   0.0287   0.0000   1.8837
  2  1  2  64.8992  17.4932   1.9470   0.0000   3.0000   0.0000   1.0135
  1  2  2   0.0000   0.0000   6.0000   0.0000   0.0000   0.0000   1.0400
  1  2  1   0.0000   7.5000   5.0000   0.0000   0.0000   0.0000   1.0400
  2  2  2   0.0000  27.9213   5.8635   0.0000   0.0000   0.0000   1.0400
  1  1  3  51.7494  42.2658   0.7914   0.0000   4.0000  58.6562   1.7461
  3  1  3  72.7766   9.2311   7.5000 -20.0000   1.1419   0.0000   1.4603
  1  1  4  58.4756  41.7584   0.6211   0.0000   1.4838   0.0000   1.6722
  3  1  4  79.3051  13.4668   1.9350   0.0000   0.4036  32.5000   1.3503
  4  1  4  65.2722   4.5000   0.4741   0.0000   0.9683   0.0000   2.1819
```

2	1	3	59.1567	27.4483	1.1736	0.0000	0.1000	0.0000	1.6111
2	1	4	61.4983	11.5971	2.1170	0.0000	0.1000	0.0000	1.2079
1	2	4	0.0000	0.0019	6.3000	0.0000	0.0000	0.0000	1.0400
1	3	1	68.7170	6.5547	0.5383	0.0000	2.2753	0.0000	1.8466
1	3	3	82.6613	45.0000	1.3380	0.0000	1.1034	68.1072	1.6232
1	3	4	90.0000	18.7269	1.4975	0.0000	3.0479	0.0000	1.0965
3	3	3	90.0000	25.0984	1.6910	0.0000	1.6310	50.0000	1.0000
3	3	4	82.4170	34.0230	1.0276	0.0000	2.6572	0.0000	1.4288
4	3	4	72.6356	45.0000	1.1114	0.0000	3.0072	0.0000	1.0000
1	3	2	90.0000	8.9828	2.3727	0.0000	1.2146	0.0000	3.0000
2	3	3	79.5453	50.0000	2.1630	0.0000	3.0000	0.0000	1.2391
2	3	4	69.4868	16.3078	4.4440	0.0000	0.2502	0.0000	1.8860
2	3	2	85.7876	9.3298	2.0747	0.0000	2.8632	0.0000	1.6905
1	4	1	81.6738	11.0200	1.6084	0.0000	2.7249	0.0000	1.4096
1	4	3	88.8926	9.1088	4.8453	0.0000	2.1900	0.0000	3.0000
1	4	4	71.4079	13.7291	2.6469	0.0000	2.9290	0.0000	3.0000
3	4	3	74.7164	43.0000	1.1816	-18.0069	3.0584	0.0000	1.1322
3	4	4	81.1388	40.7000	0.9549	-0.9193	3.0760	0.0000	1.0000
4	4	4	72.1841	29.8125	1.9658	0.0000	2.9239	0.0000	2.9000
1	4	2	88.7155	6.5761	1.8952	0.0000	0.2894	0.0000	1.1321
2	4	3	76.0014	10.1041	7.3108	0.0000	0.4346	0.0000	3.0000
2	4	4	90.0000	42.7711	1.4427	0.0000	0.1000	0.0000	1.1775
2	4	2	83.5503	12.3251	4.8243	0.0000	0.1777	0.0000	1.0020
1	2	3	0.0000	17.0624	0.9927	0.0000	0.6225	0.0000	1.0000
1	2	4	0.0000	19.3874	0.2427	0.0000	0.2078	0.0000	1.1303
1	2	5	0.0000	15.0000	3.0000	0.0000	0.0000	0.0000	1.0400
3	2	3	0.0000	1.0000	2.5054	0.0000	0.0000	0.0000	4.0000
3	2	4	0.0000	12.4279	2.9774	0.0000	0.1224	0.0000	1.8030
4	2	4	0.0000	20.0000	3.6106	0.0000	0.1460	0.0000	1.0000
2	2	3	0.0000	5.8938	3.0000	0.0000	0.0000	0.0000	1.0629
2	2	4	0.0000	0.0019	6.0000	0.0000	0.0000	0.0000	1.0400
1	1	5	74.4180	33.4273	1.7018	0.1463	0.5000	0.0000	1.6178
1	5	1	79.7037	28.2036	1.7073	0.1463	0.5000	0.0000	1.6453
2	1	5	63.3289	29.4225	2.1326	0.0000	0.5000	0.0000	3.0000
1	5	2	85.9449	38.3109	1.2492	0.0000	0.5000	0.0000	1.1000
1	5	5	80.0000	25.0000	2.0000	0.0000	0.5000	0.0000	1.3830
2	5	2	85.0000	15.1317	2.0000	0.0000	0.5000	0.0000	2.0000
2	5	5	97.0064	32.1121	2.0242	0.0000	0.5000	0.0000	2.8568
2	2	5	0.0000	0.0019	6.0000	0.0000	0.0000	0.0000	1.0400
5	4	5	62.0000	33.4273	1.7018	0.1463	0.5000	0.0000	1.0500
3	5	3	77.0699	39.4349	2.1313	-30.0000	0.9567	0.0000	1.1483

1	5	3	70.0000	35.0000	3.4223	0.0000	1.3550	0.0000	1.2002
1	5	4	70.0000	35.0000	3.4223	0.0000	1.3550	0.0000	1.2002
4	1	5	60.0000	35.0000	3.0223	0.0000	2.3550	0.0000	1.2002
3	5	4	70.0000	35.0000	3.4223	0.0000	1.3550	0.0000	1.2002
5	1	7	70.0000	35.0000	3.4223	0.0000	1.3550	0.0000	1.2002
1	3	5	73.0990	33.8942	1.2098	0.0000	0.8161	0.0000	1.1776
3	3	5	83.9753	31.0715	3.5590	0.0000	0.8161	0.0000	1.1776
2	3	5	76.9521	20.0000	2.0903	0.0000	1.0000	0.0000	1.0400
2	6	2	0.0000	49.8261	0.2093	0.0000	2.0870	0.0000	2.2895
2	2	6	0.0000	39.7818	3.1505	0.0000	1.1296	0.0000	1.1110
6	2	6	0.0000	0.5047	0.8000	0.0000	0.8933	0.0000	4.6650
2	6	6	0.0000	8.7037	0.0827	0.0000	3.5597	0.0000	1.1198
3	6	3	0.0000	9.2317	0.1000	0.0000	1.0000	0.0000	1.0920
6	3	6	0.0008	25.0000	8.0000	0.0000	1.0000	0.0000	3.0000
2	3	6	66.0423	5.0000	1.0000	0.0000	1.0000	0.0000	1.2500
2	6	3	0.0000	0.5000	0.1000	0.0000	1.0000	0.0000	3.0000
3	3	6	70.0000	20.0000	1.0000	0.0000	1.0000	0.0000	1.2500
3	7	3	90.0000	6.2396	1.3578	-24.9000	1.5156	0.0000	1.6302
2	3	7	100.0000	11.5040	1.5163	0.0000	3.6982	0.0000	1.0400
3	3	7	60.0000	40.0000	4.0000	0.0000	1.0000	0.0000	1.0400
3	2	7	0.0000	10.0000	1.0000	0.0000	1.0000	0.0000	1.0400
6	3	7	41.7961	3.4184	7.4578	0.0000	-0.2958	0.0000	1.0542
7	3	7	90.0000	16.5250	7.0313	0.0000	-0.0396	0.0000	3.0000
1	3	7	56.0610	4.2889	4.5235	0.0000	2.6593	0.0000	2.4280
2	7	3	75.0000	25.0000	2.0000	0.0000	1.0000	0.0000	1.2500
3	7	7	70.0000	25.0000	2.0000	0.0000	1.0000	0.0000	1.2500
3	9	3	96.2265	4.5610	12.0000	0.0000	0.3211	0.0000	1.5204
3	9	3	0.0000	9.1552	7.9919	0.0000	0.1660	0.0000	1.5386
9	3	9	100.0000	10.1065	6.0000	0.0000	1.0000	0.0000	3.6601
2	3	9	55.0417	3.5032	3.9979	0.0000	1.5171	0.0000	1.0400
3	3	9	70.0000	30.0000	2.0000	0.0000	1.0000	0.0000	1.2500
3	9	9	66.7783	14.3146	0.7911	0.0000	1.0000	0.0000	1.2333
3	9	10	96.6924	9.4823	5.7883	0.0000	0.2248	0.0000	2.2640
3	9	10	0.0000	3.8549	3.7230	0.0000	0.1482	0.0000	1.0400
9	10	9	0.0000	11.2336	6.8851	0.0000	1.0000	0.0000	1.0893
9	9	10	90.0000	5.0811	5.2147	0.0000	1.0000	0.0000	1.8538
10	9	10	0.0100	21.1482	0.3506	0.0000	1.0000	0.0000	1.4361
3	2	10	0.0000	0.0100	0.5211	0.0000	0.0000	0.0000	1.3859
3	9	4	100.0000	28.1532	12.0000	0.0000	0.2932	0.0000	1.6489
3	9	4	0.0000	22.7457	2.9039	0.0000	0.5593	0.0000	1.9764
4	9	4	87.0081	27.6432	3.9735	0.0000	4.0000	0.0000	1.4578

```
  4  9  4   0.0000  22.8998   3.1077   0.0000   3.0000   0.0000   1.0696
  9  4  9 100.0000  10.1065   6.0000   0.0000   1.0000   0.0000   3.6601
  2  4  9  80.0000   3.5601   3.3645   0.0000   1.5171   0.0000   1.0400
  3  4  9  70.0000  30.0000   2.0000   0.0000   1.0000   0.0000   1.2500
  4  3  9  70.0000  30.0000   2.0000   0.0000   1.0000   0.0000   1.2500
  4  4  9  70.0000  30.0000   2.0000   0.0000   1.0000   0.0000   1.2500
  4  9  9  66.7783  14.3146   0.7911   0.0000   1.0000   0.0000   1.2333
  4  9 10  95.2122   5.7090  12.0000   0.0000   0.2248   0.0000   2.8936
  4  9 10   0.0000   9.0054   7.9511   0.0000   0.1482   0.0000   1.6245
  4  2 10   0.0000  15.0000   2.8900   0.0000   0.0000   0.0000   2.8774
  1  3  9  55.0000  15.0000   1.0000   0.0000   1.0000   0.0000   1.5000
  1  4  9  55.0000  15.0000   1.0000   0.0000   1.0000   0.0000   1.5000
 66    ! Nr of torsions;at1;at2;at3;at4;;V1;V2;V3;V2(BO);vconj;n.u;n
  1  1  1  1   0.3619   5.0000   0.2257  -9.0000  -2.4400   0.0000   0.0000
  1  1  1  2   0.2330  56.0330  -0.0369  -6.3749  -1.6179   0.0000   0.0000
  2  1  1  2  -0.0662  44.7480   0.3945  -6.5879  -3.0000   0.0000   0.0000
  1  1  1  3   2.0835  80.0000   0.2861  -5.8354  -1.1000   0.0000   0.0000
  2  1  1  3   2.5000  15.1318   1.0000  -6.3682  -1.0978   0.0000   0.0000
  3  1  1  3  -1.4902  25.2456  -1.0000  -2.5000  -0.8614   0.0000   0.0000
  1  1  3  1  -1.0000  -5.0000  -0.5000  -2.5000  -0.9000   0.0000   0.0000
  1  1  3  2  -2.1885   5.0000  -0.9943  -9.0000  -0.9000   0.0000   0.0000
  2  1  3  1   0.8086  63.4490  -0.1261  -9.0000  -0.9000   0.0000   0.0000
  2  1  3  2  -2.5000  73.5049   0.8863  -4.0386  -1.1000   0.0000   0.0000
  2  1  3  3  -0.9280 100.0000   1.0000  -3.9313  -2.8274   0.0000   0.0000
  3  1  3  1  -2.5000   9.3090  -1.0000  -8.2407  -3.0437   0.0000   0.0000
  3  1  3  2   2.5000  21.4615  -0.0315  -2.5000  -3.0476   0.0000   0.0000
  1  3  3  2  -2.5000   8.2305  -0.2363  -2.7513  -2.9498   0.0000   0.0000
  2  3  3  2  -2.5000 -25.0000  -1.0000  -2.5000   0.0000   0.0000   0.0000
  1  3  3  3   1.5042 -24.6836   1.0000  -2.5000  -0.9972   0.0000   0.0000
  2  3  3  3   0.2942  70.1282  -0.7126  -6.1638   0.0000   0.0000   0.0000
  3  3  3  3  -2.5000 -25.0000   1.0000  -2.5000  -0.9000   0.0000   0.0000
  1  1  4  2   0.7954  67.9069   0.3504  -8.0000  -1.9825   0.0000   0.0000
  2  1  4  2   0.4566  46.1133   0.4807  -5.7278  -2.1051   0.0000   0.0000
  3  1  4  2   1.0000  26.3717   1.0000  -3.5214  -2.5261   0.0000   0.0000
  3  1  1  4  -0.5802   7.2544   1.0000  -2.5000  -0.9511   0.0000   0.0000
  4  1  1  4  -0.2416  15.8213  -0.5000  -3.2327  -1.7241   0.0000   0.0000
  1  1  4  1  -1.0000  51.5166   0.1228  -6.5755  -1.6589   0.0000   0.0000
  3  1  4  1  -1.0000  14.9858   1.0000  -8.0000  -1.8038   0.0000   0.0000
  2  1  1  4   1.0000   0.0100   0.8120  -2.8086  -1.9000   0.0000   0.0000
  4  1  4  2   1.0000  51.6090  -0.5000  -3.9390  -2.0202   0.0000   0.0000
  2  1  4  1  -0.2342  68.6969   0.1904  -5.3454  -0.0100   0.0000   0.0000
```

```
4  1  3  1  -0.8484  15.6632  -0.3865  -2.5000  -3.0437   0.0000   0.0000
4  1  3  2   0.0107   3.7120  -0.5000  -7.9280  -3.0476   0.0000   0.0000
1  1  1  4   0.9187  -5.0000   1.0000  -6.3298  -2.0000   0.0000   0.0000
4  1  4  1  -0.8232  16.3430   0.5477  -7.0692  -2.0051   0.0000   0.0000
0  1  2  0   0.0000   0.0000   0.0000  -4.0000   0.0000   0.0000   0.0000
0  2  2  0   0.0000   0.0000   0.0000  -4.0000   0.0000   0.0000   0.0000
0  2  3  0   0.0000   0.1000   0.0200  -4.0000   0.0000   0.0000   0.0000
0  1  1  0   0.0000  50.0000   0.3000  -4.0000  -2.0000   0.0000   0.0000
0  3  3  0   0.5511  25.4150   1.1330  -5.1903  -1.0000   0.0000   0.0000
0  1  4  0   0.2176  40.4126   0.3535  -3.9875  -2.0051   0.0000   0.0000
0  2  4  0   0.0000   0.1000   0.0100  -5.0000   0.0000   0.0000   0.0000
0  3  4  0   1.1397  61.3225   0.5139  -3.8507  -2.7831   0.0000   0.0000
0  4  4  0   0.7265  44.3155   1.0000  -4.4046  -2.0000   0.0000   0.0000
4  1  4  4  -0.0949   8.7582   0.3310  -7.9430  -2.0000   0.0000   0.0000
0  1  5  0   0.8251  92.1468   0.7176  -4.2341   0.0000   0.0000   0.0000
0  5  5  0   0.1291  -5.0000   0.9649  -5.0903   0.0000   0.0000   0.0000
0  2  5  0   0.0000   0.0000   0.0000   0.0000   0.0000   0.0000   0.0000
0  6  6  0   0.0000   0.0000   0.1200  -2.4426   0.0000   0.0000   0.0000
0  2  6  0   0.0000   0.0000   0.1200  -2.4847   0.0000   0.0000   0.0000
0  3  6  0   0.0000   0.0000   0.1200  -2.4703   0.0000   0.0000   0.0000
1  1  3  3  -0.0002  20.1851   0.1601  -9.0000  -2.0000   0.0000   0.0000
1  3  3  1   0.0002  80.0000  -1.5000  -4.4848  -2.0000   0.0000   0.0000
3  1  3  3  -0.1583  20.0000   1.5000  -9.0000  -2.0000   0.0000   0.0000
2  3  9  3  -1.5000   6.8333  -0.1978  -1.4683   0.0000   0.0000   0.0000
2  3  9  4  -0.6181   7.1542  -0.0047  -1.6577   0.0000   0.0000   0.0000
2  4  9  3  -1.5000   1.7820  -1.0000  -5.4916   0.0000   0.0000   0.0000
2  4  9  4  -0.1959   2.3626  -1.0000  -3.0702   0.0000   0.0000   0.0000
2  1  4  9   0.0000  10.0000   0.3000  -6.0000  -1.0000   0.0000   0.0000
2  3  9 10   0.1589  12.5000   0.4388  -1.5000   0.0000   0.0000   0.0000
7  1  1  7   1.0657  78.7067   0.6978  -8.4833  -1.7255   0.0000   0.0000
0  1  7  0   1.4891  35.1275   0.8254  -2.8518   0.0000   0.0000   0.0000
0  7  7  0  -0.3570  38.0764   0.1676  -6.7257   0.0000   0.0000   0.0000
1  1  1  7  -0.1661  19.8637   0.2351 -10.8000  -1.7255   0.0000   0.0000
2  1  3  7  -1.5000  99.6206   0.0516  -8.1129   0.0000   0.0000   0.0000
2  3  7  3  -1.5000  -1.0000   0.2523  -2.7183   0.0000   0.0000   0.0000
1  3  7  3  -1.5000  50.2166   0.9871  -3.6395   0.0000   0.0000   0.0000
7  3  7  3   1.5000  -0.9128   0.0100  -6.2667   0.0000   0.0000   0.0000
1  1  3  7   0.0000   0.1000   0.0100  -4.0000   0.0000   0.0000   0.0000
9    ! Nr of hydrogen bonds;at1;at2;at3;Rhb;Dehb;vhb1
3  2  3   2.1493  -3.8387   1.5230  28.0017
3  2  4   2.0543  -6.3062   1.4500  19.5000
```

```
4  2  3   1.7500  -1.8134   1.4500  19.5000
4  2  4   1.8886  -1.8024   1.4500  19.5000
3  2  5   1.5000  -2.0000   1.4500  19.5000
4  2  5   1.5000  -2.0000   1.4500  19.5000
5  2  3   1.5000  -2.0000   1.4500  19.5000
5  2  4   1.5000  -2.0000   1.4500  19.5000
5  2  5   1.5000  -2.0000   1.4500  19.5000
```

Listing S5: ReaxFF force-field file `ffield` (C/H/O subset used via `pair_coeff * * ffield C H O`). The header line identifies the parameterization as "CHON-2017_weak reparametrized for bond-boost" (DOI 10.1021/acs.jpca.8b03826). Byte-identical in all six loading directories.

S2. System composition

Table S1 lists the composition of the six systems. Every system contains 50 polyethylene chains of $C_{30}H_{62}$ (1500 C atoms, 3100 H atoms; PE mass 21,141.3 amu) and $n(O_2)$ O_2 molecules. The simulation cells are not cubic. The initial mass densities span 0.1957–0.2038 $\mathrm{g\,cm^{-3}}$ (NVT: the initial and production densities are identical by construction). The O_2-to-PE mass ratios span 0.15% to 136.2%, and the O:C atomic ratios span 0.0013 to 1.2. No water molecules are present in any initial or production-start configuration; water occurs only as a reaction product during the production runs.

Table S1: Composition of the six PE/O_2 systems. Every system contains 50 $C_{30}H_{62}$ chains (1500 C atoms and 3100 H atoms; PE mass 21,141.3 amu); $n(O_2)$ is the number of O_2 molecules inserted initially. Box: initial cell dimensions $L_x \times L_y \times L_z$; V: cell volume; $\rho_{\mathrm{init}}/\rho_{\mathrm{prod}}$: mass densities of the initial/production configurations (identical by construction: NVT); O_2:PE: O_2-to-PE mass ratio; O_2/chain: O_2 molecules per PE chain; O/C: O:C atom ratio; O_2@prod: O_2 pairs at the start of the production run (O_2 partially dissociates during the 300 K equilibration for $n(O_2)$ = 100, 700, and 900; free O atoms at the production start: zero in all systems); H_2O: water molecules in the initial configuration (zero in all systems; water occurs only as a reaction product).

System	$n(O_2)$	Atoms	C	H	O	Box (Å)	V (Å^3)	Mass (amu)	ρ_{init} ($\mathrm{g\,cm^{-3}}$)	ρ_{prod} ($\mathrm{g\,cm^{-3}}$)	O_2:PE mass	O_2/chain	O/C atomic	O_2@prod pairs	H_2O molec.
f-50-1	1	4602	1500	3100	2	56.106 × 56.144 × 55.87	175,992.9	21,173.3	0.1998	0.19978	0.0015	0.02	0.0013	1	0
f-50-100	100	4800	1500	3100	200	59.02 × 59.1 × 59.016	205,851.9	24,341.1	0.1964	0.19635	0.1514	2	0.1333	98	0

continued on next page

Table S1 (continued)

System	$n(O_2)$	Atoms	C	H	O	Box (Å)	V (Å^3)	Mass (amu)	ρ_{init} (g cm^{-3})	ρ_{prod} (g cm^{-3})	O_2:PE mass	O_2/chain	O/C atomic	O_2@prod pairs	H_2O molecules
f-50-300	300	5200	1500	3100	600	62.997 × 63.001 × 63.1	250,437.8	30,740.7	0.2038	0.20383	0.4541	6	0.4	300	0
f-50-500	500	5600	1500	3100	1000	68.042 × 68.001 × 68.1	315,092.4	37,140.3	0.1957	0.19573	0.7568	10	0.6667	500	0
f-50-700	700	6000	1500	3100	1400	71.033 × 71.086 × 70.996	358,492.7	43,539.9	0.2017	0.20163	1.0595	14	0.9333	699	0
f-50-900	900	6400	1500	3100	1800	75.1 × 75.046 × 75.018	422,799.9	49,939.5	0.1961	0.1961	1.3622	18	1.2	899	0

S3. Trajectory statistics

Table S2 lists the per-trajectory statistics of all 48 production runs. Of the 48 runs, 45 (the "ML" runs) completed the full 500 ps and form the machine-learning dataset; three runs are truncated and excluded from the dataset: `f-50-700_T1500` (ends at 93.25 ps), `f-50-900_T1500` (11.0 ps), and `f-50-900_T1700` (183.5 ps). Their trailing analyzer rows carry $T = \rho = P = 0$ (thermo merge artifacts).

Row bookkeeping. The per-frame records are event-sampled (variable Δt): the nominal frame spacing is 0.25 ps, but the analyzer writes a row only at events, with inter-frame gaps up to 29 ps in the coldest runs; the raw counts in Table S2 therefore vary across simulations. Across all 48 runs the raw analyzer output contains 76,865 rows. Hygiene filtering then proceeds in two steps: (i) deduplication on (simulation, time step), which removes 48 rows, since every simulation repeats its final step-2,000,000 row; and (ii) a sentinel filter on $N_{H_2} < 0$, which removes 8 raw rows in total, namely 4 unique rows, occurring as duplicated pairs at the final analyzer step of the four runs marked † (the sentinel row is one of the duplicated final-step rows there, so deduplication and sentinel filtering together remove 2 rows per such run and 1 row in every other run; net removal 52 rows). This leaves 76,813 retained rows, of which 76,679 remain after warm-up-row removal. For the 45 ML runs the corresponding counts are 76,447 raw, 76,398 retained, and 76,334 post-warm-up rows. The final-step analyzer artifact row (written post-run at $t = 500$ ps with partial species perception) is *kept*: its $\Delta t = 0$ contributes exactly zero to the integral target of Section S4, it can never serve as a feature row, and the N_{H_2} column of Table S2 reports the last *valid* frame.

Warm-up. The warm-up estimate t_{warm} is the first time the measured temperature stays within a ±5% band around the target temperature. The robust rule (used throughout) ignores isolated single-row out-of-band excursions after the initial heating prefix: out-of-band runs of ≥ 2 consecutive rows (or the initial heating prefix itself) reset the estimate; the strict literal rule is also reported ($t^{\mathrm{st}}_{\mathrm{warm}}$). Among the ML runs, t_{warm} has median 2.00 ps, mean 3.20 ps, minimum 1.50 ps, and maximum 17.00 ps. The robust and strict estimates differ for four ML runs: `f-50-1_T1700` (2.00 vs. 4.25 ps), `f-50-100_T2500` (1.75 vs. 2.50 ps), `f-50-1_T2900` (2.00 vs. 199.25 ps), and `f-50-900_T1500` (8.75 ps vs. undefined; excluded from ML in any case). In `f-50-1_T2900` the discrepancy is caused by a single isolated temperature excursion at $t = 199.0$ ps (3045.14 K, i.e., 0.14 K above the 3045.0 K band edge around the 2900 K target), which the robust rule ignores as thermostat noise and the strict rule treats as the end of the warm-up.

Production state. By construction the post-warm-up production means $\langle T \rangle$ stay within ±5% of the target, and the mass densities are those of Table S1. The post-warm-up production pressures of the ML runs span 68.45 atm (`f-50-100_T1500`) to 2928.31 atm (`f-50-1_T2900`); pressures are LAMMPS `real`-unit values (atm). These high-temperature, high-pressure NVT states are a designed scan of the kinetic accessibility of PE oxidation chemistry, as discussed in the main text.

H_2 outcome measures. N_{H_2} is the instantaneous H_2 count at the last valid frame; cumH2 is the time-integrated cumulative H_2 production (molecule·ps; Section S4). A run is flagged "plateau" when the mean H_2 production rate over the final 100 ps is below 10% of the maximum instantaneous N_{H_2}. Thirteen of the 48 trajectories produce no H_2 at all (all six 1500 K runs, all six 1700 K runs, and `f-50-1_T1900`; 10 of these are ML runs and 3 are the truncated runs), and 14 of the 48 are flagged plateau (the 13 no-H_2 runs plus `f-50-300_T1900`, where H_2 first appears at 366.25 ps). The largest cumulative production is 151,612 molecule·ps (`f-50-300_T2900`); the largest final cumH2 among the ML runs at each temperature is 0 (1500 K), 0 (1700 K), 524 (1900 K), 3442 (2100 K), 13,277 (2300 K),

38,255 (2500 K), 88,189 (2700 K), and 151,612 (2900 K) molecule·ps.

Table S2: Per-trajectory statistics of the 48 production runs (6 O_2 loadings × 8 temperatures). ML: included in the machine-learning dataset (45 runs; the remaining 3 are truncated runs marked ‡). t_{end}: last retained trajectory time. $t_{\mathrm{warm}}/t^{\mathrm{st}}_{\mathrm{warm}}$: robust/strict warm-up estimates (robust: ±5% band around the run's mean temperature, warm-up ends at the last out-of-band row with ≥2 consecutive out-of-band rows required to reset the estimate, isolated single-row excursions ignored; strict: same band without the excursion rule). raw/ret./post: analyzer rows before hygiene filtering / after duplicate and sentinel-row removal / after warm-up-row removal. $\langle\cdot\rangle$: post-warm-up production means (P in atm, LAMMPS `real` units). N_{H_2}: instantaneous H_2 count at the last valid frame; cumH2: time-integrated cumulative H_2 (molecule·ps, left-Riemann sum of N_{H_2} over the retained trajectory); t_{first}: first time with $N_{H_2} > 0$; no-H_2: the trajectory produces no H_2; plat.: plateau flag, i.e., the mean H_2 production rate over the final 100 ps is below 10% of the maximum instantaneous N_{H_2}. †: run contains final-step sentinel frames ($N_{H_2} = -1$) that were excluded during hygiene filtering; ‡: truncated trajectory (trailing analyzer artifact removed). "–": not available.

Simulation	$n(O_2)$	T (K)	ML	t_{end} (ps)	t_{warm} (ps)	$t^{\mathrm{st}}_{\mathrm{warm}}$ (ps)	raw rows	ret. rows	post rows	$\langle T\rangle$ (K)	$\langle\rho\rangle$ (g cm^{-3})	$\langle P\rangle$ (atm)	N_{H_2}	cumH2 (molec. ps)	t_{first} (ps)	no-H_2	plat.
f-50-1_T1500	1	1500	yes	500	2.5	2.5	166	165	164	1499.53	0.19978	129.42	0	0	–	yes	yes
f-50-1_T1700	1	1700	yes	500	2	4.25	429	428	427	1698.32	0.19978	126.31	0	0	–	yes	yes
f-50-1_T1900	1	1900	yes	500	9.25	9.25	1395	1394	1393	1900.82	0.19978	235.59	0	0	–	yes	yes
f-50-1_T2100	1	2100	yes	500	2.25	2.25	1918	1917	1915	2100.58	0.19978	546.34	5	1070	66	–	–
f-50-1_T2300	1	2300	yes	500	1.5	1.5	1989	1988	1987	2299.93	0.19978	1160.16	46	12,404	3.75	–	–
f-50-1_T2500†	1	2500	yes	499.75	2	2	1996	1994	1992	2500.22	0.19978	1696.95	105	29,035	17.75	–	–
f-50-1_T2700	1	2700	yes	500	2	2	1996	1995	1993	2700.8	0.19978	2299.34	263	72,144	7	–	–
f-50-1_T2900†	1	2900	yes	499.75	2	199.25	1997	1995	1992	2900.93	0.19978	2928.31	429	134,165	5.75	–	–
f-50-100_T1500	100	1500	yes	500	7	7	253	252	251	1501.03	0.19635	68.45	0	0	–	yes	yes
f-50-100_T1700	100	1700	yes	500	4.25	4.25	726	725	724	1701.81	0.19635	206.79	0	0	–	yes	yes
f-50-100_T1900	100	1900	yes	500	3	3	1556	1555	1554	1900.35	0.19635	304.9	1	61	438.75	–	–
f-50-100_T2100	100	2100	yes	500	2.25	2.25	1928	1927	1925	2100.07	0.19635	646.47	15	3442	31	–	–
f-50-100_T2300	100	2300	yes	500	2	2	1993	1992	1989	2300.15	0.19635	1231.57	47	11,371	10.25	–	–
f-50-100_T2500	100	2500	yes	500	1.75	2.5	1994	1993	1991	2501.28	0.19635	1669.87	139	35,609	15.75	–	–
f-50-100_T2700	100	2700	yes	500	2	2	1995	1994	1993	2700.46	0.19635	2159.55	280	72,892	5	–	–
f-50-100_T2900	100	2900	yes	500	1.75	1.75	1997	1996	1993	2899.6	0.19635	2754.59	497	145,620	4.5	–	–
f-50-300_T1500	300	1500	yes	500	6.5	6.5	446	445	444	1498.35	0.20383	162.59	0	0	–	yes	yes
f-50-300_T1700	300	1700	yes	500	2.5	2.5	1309	1308	1307	1701.25	0.20383	289.53	0	0	–	yes	yes
f-50-300_T1900	300	1900	yes	500	3.25	3.25	1664	1663	1662	1901.3	0.20383	425.19	1	8	366.25	–	yes
f-50-300_T2100	300	2100	yes	500	4	4	1971	1970	1969	2100.12	0.20383	895.76	6	1569	100.25	–	–
f-50-300_T2300	300	2300	yes	500	2	2	1991	1990	1989	2300.24	0.20383	1368.93	47	11,384	7.25	–	–
f-50-300_T2500	300	2500	yes	500	1.5	1.5	1996	1995	1994	2500.43	0.20383	1785.81	163	37,764	25.75	–	–
f-50-300_T2700	300	2700	yes	500	1.75	1.75	1995	1994	1993	2701.63	0.20383	2253.43	337	88,189	4.75	–	–
f-50-300_T2900	300	2900	yes	500	1.5	1.5	1998	1997	1995	2901.66	0.20383	2674.99	518	151,612	3	–	–
f-50-500_T1500	500	1500	yes	500	17	17	481	480	479	1499.88	0.19573	207.54	0	0	–	yes	yes
f-50-500_T1700	500	1700	yes	500	9.5	9.5	1408	1407	1406	1699.64	0.19573	311.27	0	0	–	yes	yes
f-50-500_T1900	500	1900	yes	500	2	2	1893	1892	1891	1899.99	0.19573	566.54	3	460	297.5	–	–
f-50-500_T2100	500	2100	yes	500	1.5	1.5	1988	1987	1986	2100.95	0.19573	926.31	16	2328	97.5	–	–
f-50-500_T2300	500	2300	yes	500	1.5	1.5	1993	1992	1991	2300.39	0.19573	1295.83	63	13,277	25.75	–	–
f-50-500_T2500†	500	2500	yes	499.75	1.5	1.5	1995	1993	1992	2500.63	0.19573	1709.41	162	35,062	11.25	–	–
f-50-500_T2700	500	2700	yes	500	1.5	1.5	1997	1996	1995	2699.65	0.19573	2080.91	331	83,869	4.75	–	–

continued on next page

Table S2 (continued)

Simulation	$n(O_2)$	T (K)	ML	t_{end} (ps)	t_{warm} (ps)	t^{st}_{warm} (ps)	raw rows	ret. rows	post rows	$\langle T \rangle$ (K)	$\langle \rho \rangle$ (g cm^{-3})	$\langle P \rangle$ (atm)	N_{H_2}	cumH2 (molec. ps)	t_{first} (ps)	no-H_2	plat.
f-50-500_T2900	500	2900	yes	500	2	2	1995	1994	1993	2900.6	0.19573	2411.26	504	143,228	5.25	–	–
f-50-700_T1500‡	700	1500	no	93.25	–	–	69	68	0	–	–	–	0	0	–	yes	yes
f-50-700_T1700	700	1700	yes	500	6.5	6.5	1251	1250	1249	1700.95	0.20168	408.56	0	0	–	yes	yes
f-50-700_T1900	700	1900	yes	500	5	5	1914	1913	1912	1900.11	0.20168	652.26	2	524	144.25	–	–
f-50-700_T2100	700	2100	yes	500	1.75	1.75	1977	1976	1974	2100.32	0.20168	971.99	11	2280	84.75	–	–
f-50-700_T2300	700	2300	yes	500	3.25	3.25	1987	1986	1985	2299.98	0.20168	1358.89	51	10,277	8	–	–
f-50-700_T2500	700	2500	yes	500	1.75	1.75	1997	1996	1994	2500.51	0.20168	1723.11	163	38,255	9.5	–	–
f-50-700_T2700	700	2700	yes	500	2	2	1995	1994	1993	2699.71	0.20168	2055.71	286	73,726	10	–	–
f-50-700_T2900	700	2900	yes	500	1.75	1.75	1996	1995	1994	2900.3	0.20168	2399.68	524	141,407	2.25	–	–
f-50-900_T1500‡	900	1500	no	11	8.75	–	6	5	4	1131.18	0.1471	121.05	0	0	–	yes	yes
f-50-900_T1700‡	900	1700	no	183.5	10.5	10.5	343	342	341	1699.41	0.19614	454.74	0	0	–	yes	yes
f-50-900_T1900	900	1900	yes	500	1.5	1.5	1940	1939	1938	1899.89	0.19614	707.12	1	432	288	–	–
f-50-900_T2100	900	2100	yes	500	2.25	2.25	1978	1977	1976	2100.69	0.19614	991.16	9	2629	147.75	–	–
f-50-900_T2300	900	2300	yes	500	3.75	3.75	1984	1983	1982	2300.07	0.19614	1329.2	54	9937	25.25	–	–
f-50-900_T2500†	900	2500	yes	499.75	3	3	1987	1985	1984	2501.03	0.19614	1643.4	141	32,791	21	–	–
f-50-900_T2700	900	2700	yes	500	2.5	2.5	1995	1994	1991	2699.84	0.19614	1951.79	329	79,463	5.75	–	–
f-50-900_T2900	900	2900	yes	500	2	2	1998	1997	1993	2900.09	0.19614	2284.9	511	135,808	6	–	–

S4. Dataset construction and train/test partition

Target. The forecasting target is the time-weighted cumulative H_2 production

$$\mathrm{cumH2}(t_k) \;=\; \sum_{s \le k} N_{\mathrm{H}_2}(t_s)\,\Delta t_s, \qquad \Delta t_s = t_{s+1} - t_s, \tag{S1}$$

a left Riemann sum in which each recorded frame is weighted by the interval to the *next* recorded frame (event-based sampling, variable Δt; Section S3). The final recorded frame has no forward interval ($\Delta t = 0$) and contributes zero. Units are molecule·ps, and cumH2 is monotone non-decreasing (verified for all 48 simulations). The column `cumulative_H2` (a plain H_2 count) survives only as an H2-history input feature of Case A, never as a target.

Forecast pairs. For each of the 45 ML simulations and each horizon $h \in \{2.5, 25, 50\}$ ps (primary: $h = 25$ ps), every post-warm-up origin row i is paired with j, the first row with $t_j \ge t_i + h$ (searchsorted with a 10^{-6} ps round-off snap). The pair is skipped if no such j exists (the origin lies within the final h of the trajectory) or if $t_j - t_i > h + 5$ ps (event-gap protection). Features are taken strictly at row i; the target is $y = \mathrm{cumH2}(t_j)$; the

persistence baseline is cumH2(t_i), i.e., the assumption of zero production over the horizon. The final analyzer row ($t = 500$ ps, $\Delta t = 0$) can only ever serve as j, never as i. At the primary horizon this yields **72,117 pairs** (189 skipped by the event-gap tolerance, 4028 skipped for lack of a future row; realized horizon gap mean 25.044 ps, maximum 30.0 ps; 95.4% of pairs on the exact time grid), with target mean 15,191, median 2140, and maximum 151,612 molecule·ps. The horizon variants give 75,725 pairs at $h = 2.5$ ps and 68,122 pairs at $h = 50$ ps. The target equals the persistence value (zero production) for 13,113 of the 72,117 primary pairs (18.2%); these come from the genuine no-H_2 trajectories and are kept. $y \geq$ persistence holds for every pair by construction. MAPE is computed with an absolute floor of $\varepsilon = 1$ molecule·ps in the denominator (Section S7).

Feature pools. The Case A candidate pool contains 48 trajectory-derived features; Case B removes all ten features derived from the H_2 signal, leaving 38. The pool difference is: `cumulative_H2`, `N_H2_lag1`, `N_H2_lag2`, `dN_H2`, `dN_H2_dt`, `rolling_mean_H2`, `rolling_std_H2`, `H2_selectivity`, `H_C_ratio`, and `running_Ea`. The instantaneous H_2 count N_{H_2} is an input in *neither* case. Metadata (simulation identifier, temperature, the O_2 loading, the horizon, and the t_i/t_j stamps) and the two target/baseline columns are never used as model inputs. The O_2 loading is a per-simulation constant, shared between the metadata block and both feature pools.

Train/test partition. The 45 ML simulations are split into simulation groups by a single `GroupShuffleSplit` with test size 0.2 and random state 42: **36 training simulations** (57,658 primary-horizon rows) and **9 test simulations** (14,459 rows); 57,658 + 14,459 = 72,117. The split is identical for Case A and Case B, and no simulation's rows are ever divided between training and evaluation. The simulation identifiers of both partitions are listed in Table S3; the condition coverage is summarized in Table S4. All eight temperatures and all six loadings are represented in the train set; the test set contains no 2100, 2500, or 2900 K run, no $n(O_2) = 300$ run, and includes two zero-H_2 simulations (`f-50-1_T1500` and `f-50-500_T1700`). The test partition was evaluated exactly once, after feature selection,

model choice, and all hyperparameters had been frozen on the training partition alone (Sections S5–S7); splitting and all model fitting respect simulation groups to preclude leakage.

Kernel/SVR subsampling. To keep kernel and support-vector fits tractable, GPR and SVR are trained on deterministic per-simulation subsamples: for simulation i with n_i rows out of N fitting rows, $c_i = \lceil n_i S/N \rceil$ rows are drawn with a fixed stride and random phase, with $S = 2000$ for GPR and $S = 5000$ for SVR.

Table S3: Train/test partition of the 45 machine-learning simulations, identical for Case A and Case B (GroupShuffleSplit with test size 0.2 and random state 42, split on simulation groups). n_{rows}: number of $h = 25$ ps feature rows contributed by the simulation (post warm-up).

Simulation	$n(O_2)$	T (K)	n_{rows}	Partition
f-50-1_T1700	1	1700	357	Train
f-50-1_T1900	1	1900	1318	Train
f-50-1_T2100	1	2100	1816	Train
f-50-1_T2500	1	2500	1892	Train
f-50-1_T2700	1	2700	1893	Train
f-50-1_T2900	1	2900	1892	Train
f-50-100_T1500	100	1500	175	Train
f-50-100_T1700	100	1700	663	Train
f-50-100_T1900	100	1900	1458	Train
f-50-100_T2100	100	2100	1827	Train
f-50-100_T2500	100	2500	1891	Train
f-50-100_T2700	100	2700	1893	Train
f-50-100_T2900	100	2900	1893	Train
f-50-300_T1500	300	1500	408	Train
f-50-300_T1700	300	1700	1215	Train
f-50-300_T1900	300	1900	1562	Train
f-50-300_T2100	300	2100	1869	Train
f-50-300_T2300	300	2300	1889	Train
f-50-300_T2500	300	2500	1894	Train

continued on next page

Table S3 (continued)

Simulation	$n(O_2)$	T (K)	n_{rows}	Partition
f-50-300_T2700	300	2700	1893	Train
f-50-300_T2900	300	2900	1895	Train
f-50-500_T1500	500	1500	434	Train
f-50-500_T2100	500	2100	1886	Train
f-50-500_T2300	500	2300	1891	Train
f-50-500_T2500	500	2500	1892	Train
f-50-500_T2700	500	2700	1895	Train
f-50-500_T2900	500	2900	1893	Train
f-50-700_T1700	700	1700	1154	Train
f-50-700_T1900	700	1900	1812	Train
f-50-700_T2100	700	2100	1874	Train
f-50-700_T2500	700	2500	1894	Train
f-50-700_T2700	700	2700	1893	Train
f-50-700_T2900	700	2900	1894	Train
f-50-900_T2100	900	2100	1876	Train
f-50-900_T2500	900	2500	1884	Train
f-50-900_T2900	900	2900	1893	Train
f-50-1_T1500	1	1500	87	Test
f-50-1_T2300	1	2300	1887	Test
f-50-100_T2300	100	2300	1889	Test
f-50-500_T1700	500	1700	1309	Test
f-50-500_T1900	500	1900	1791	Test
f-50-700_T2300	700	2300	1885	Test
f-50-900_T1900	900	1900	1838	Test
f-50-900_T2300	900	2300	1882	Test
f-50-900_T2700	900	2700	1891	Test

Table S4: Condition coverage of the partition of Table S3: number of simulations per temperature level and per O_2 loading level. The train set contains 36 simulations (57,658 rows), the test set 9 simulations (14,459 rows).

Factor	Level	Train	Test
Temperature	1500 K	3	1
Temperature	1700 K	4	1
Temperature	1900 K	4	2
Temperature	2100 K	6	0
Temperature	2300 K	2	4
Temperature	2500 K	6	0
Temperature	2700 K	5	1
Temperature	2900 K	6	0
$n(O_2)$	1	6	2
$n(O_2)$	100	7	1
$n(O_2)$	300	8	0
$n(O_2)$	500	6	2
$n(O_2)$	700	6	1
$n(O_2)$	900	3	3

S5. Feature selection

Feature selection is a six-stage cascade re-run inside every training fold (and inside every inner tuning fold), so no evaluation data ever take part in the selection. The stages are:

S1 variance filter, threshold 0.01 (`VarianceThreshold`);

S2 Pearson correlation filter: for every pair with $|\rho| > 0.8$, the member with the smaller variance is dropped (ties drop the second member);

S3 mutual-information ranking (`mutual_info_regression`, `random_state=42`, `n_neighbors=3`), keeping the top 50% (rounded down);

S4 random-forest permutation importance (`n_estimators=100`, `max_depth=20`, seed 42, 10 repeats), keeping the top 25 (all features kept if fewer than 25 remain);

S5 recursive feature elimination with a random forest, to 15 features (skipped if at most 15 remain);

S6 XGBoost TreeSHAP importance, keeping the top 12 (skipped if at most 12 remain; computed on a 5000-row subsample, seed 42).

Because the largest post-S3 count is 8 features (Case A) and 6 (Case B), the S4–S6 guards bind in *every* run: each run's selected set equals its stage-3 survivor set. Table S5 lists the stage-by-stage feature counts of all 92 fold-level selection runs (15 inner folds, 5 outer folds, and 25 stratified-repeat folds per case, plus the final refit); Table S6 lists the per-fold selected sets.

Stage-1 drops. Stage 1 removes the same eight features in every run of both cases: `rho`, `O_C_ratio`, `bond_fraction`, `charge_fraction`, `Hbond_bond_ratio`, `conformational_frac`, `dC_conv_dt`, and `WGS_Keq` (variance below the 0.01 threshold).

Temperature, O_2 loading, and simulation time. The temperature T and the O_2 loading are eliminated at *stage 2* (not by the later model-based stages) in every one of the 31 replicable selection runs of each case, and also in both final refits; the simulation time t survives the full cascade in every run of both cases and is a member of every selected set. In the final refit (full 36-simulation training set), T exceeds the 0.8 threshold with four partners: the conjugation energy `E_co` ($|\rho| = 0.831$; variances 8.81×10^5 vs. 1.55×10^5, so T is dropped), the conversion `C_conv` ($|\rho| = 0.813$), the selectivity `H2_selectivity` ($|\rho| = 0.819$), and the H/C ratio `H_C_ratio` ($|\rho| = 0.810$) (in the last three pairs T has the larger variance and the partner is dropped). The O_2 loading exceeds the threshold with the pair, total, and bond energies: `E_pair` ($|\rho| = 0.976$), `E_tot` ($|\rho| = 0.964$), and `E_b` ($|\rho| = 0.861$), whose variances dominate in every case, so the loading is dropped. Table S8 summarizes the stage-wise fate of the three descriptors across all runs.

Final refit sets. The final refit chains run 48 → 40 → 15 → 7 (Case A) and 38 → 30 → 11 → 5 (Case B). The stage-2 survivor sets of the final refit are, for Case A (15): `t`, `P`, `E_b`, `E_a`, `E_p`, `N_CH4`, `N_C2H4`, `dE_b_dt`, `dN_H2_dt`, `dE_qeq_dt`, `running_Ea`, `cumulative_H2`, `energy_dissipated`, `N_OH_radical`, `rolling_std_H2` (25 drops: `C_conv`, `Coulomb_VdW_ratio`, `E_co`, `E_coa`, `E_hb`, `E_lp`, `E_pair`, `E_pen`, `E_qeq`, `E_t`, `E_tot`, `E_v`, `E_w`, `H2_selectivity`, `H_C_ratio`, `N_C2H2`, `N_C2H6`, `N_CO2`, `N_H2_lag1`, `N_H2_lag2`, `N_H_radical`, `T`, `dN_H2`, `rolling_mean_H2`, `O2_loading`); and for Case B (11): `t`, `P`, `E_b`, `E_a`, `E_p`, `N_CH4`, `N_C2H4`, `dE_b_dt`, `dE_qeq_dt`, `energy_dissipated`, `N_OH_radical` (19 drops). Stage 3 keeps the top half of each, giving the final selected sets {`cumulative_H2`, `t`, `N_C2H4`, `E_p`, `N_CH4`, `E_b`, `E_a`} (Case A) and {`E_p`, `E_b`, `E_a`, `N_C2H4`, `t`} (Case B); see the "final" rows of Table S6. Only 12 of the 48 Case A features and 9 of the 38 Case B features are ever selected in any fold; Table S7 lists the selection frequency of every pool feature across the outer folds, inner folds, stratified-repeat folds, and the final refit.

Table S5: Stage-by-stage feature counts of all 92 fold-level selection runs, exactly as logged during training: pool → S1 (variance filter, threshold 0.01) → S2 (Pearson correlation filter, $|\rho| > 0.8$) → S3 (mutual-information ranking, top 50%) → S4 (random-forest permutation importance, top 25) → S5 (RFE with a random forest, to 15) → S6 (TreeSHAP with XG-Boost, top 12). Labels: `outer`k`-inner`j = inner fold j of outer fold k of the first nested pass; `outer`k = outer-fold refit of the first pass; `strat`s`-`k = fold k of the stratified repeat with seed s; `final` = refit on the full 36-simulation train set. In every run the stage-4–6 guards bind after stage 3 (largest post-S3 count: 8 features in Case A, 6 in Case B), so each selected set equals the stage-3 survivor set of its run.

Case	Run	Pool	S1	S2	S3	S4	S5	S6
A	outer1-inner1	48	40	14	7	7	7	7
A	outer1-inner2	48	40	14	7	7	7	7
A	outer1-inner3	48	40	15	7	7	7	7
A	outer2-inner1	48	40	16	8	8	8	8
A	outer2-inner2	48	40	14	7	7	7	7
A	outer2-inner3	48	40	15	7	7	7	7
A	outer3-inner1	48	40	15	7	7	7	7
A	outer3-inner2	48	40	14	7	7	7	7
A	outer3-inner3	48	40	15	7	7	7	7

continued on next page

Table S5 (continued)

Case	Run	Pool	S1	S2	S3	S4	S5	S6
A	outer4-inner1	48	40	16	8	8	8	8
A	outer4-inner2	48	40	16	8	8	8	8
A	outer4-inner3	48	40	15	7	7	7	7
A	outer5-inner1	48	40	16	8	8	8	8
A	outer5-inner2	48	40	15	7	7	7	7
A	outer5-inner3	48	40	15	7	7	7	7
A	outer1	48	40	14	7	7	7	7
A	outer2	48	40	15	7	7	7	7
A	outer3	48	40	15	7	7	7	7
A	outer4	48	40	15	7	7	7	7
A	outer5	48	40	15	7	7	7	7
A	strat42-1	48	40	15	7	7	7	7
A	strat42-2	48	40	15	7	7	7	7
A	strat42-3	48	40	15	7	7	7	7
A	strat42-4	48	40	16	8	8	8	8
A	strat42-5	48	40	14	7	7	7	7
A	strat43-1	48	40	15	7	7	7	7
A	strat43-2	48	40	15	7	7	7	7
A	strat43-3	48	40	15	7	7	7	7
A	strat43-4	48	40	15	7	7	7	7
A	strat43-5	48	40	14	7	7	7	7
A	strat44-1	48	40	15	7	7	7	7
A	strat44-2	48	40	15	7	7	7	7
A	strat44-3	48	40	15	7	7	7	7
A	strat44-4	48	40	15	7	7	7	7
A	strat44-5	48	40	15	7	7	7	7
A	strat45-1	48	40	15	7	7	7	7
A	strat45-2	48	40	15	7	7	7	7
A	strat45-3	48	40	15	7	7	7	7
A	strat45-4	48	40	15	7	7	7	7
A	strat45-5	48	40	14	7	7	7	7
A	strat46-1	48	40	15	7	7	7	7
A	strat46-2	48	40	15	7	7	7	7
A	strat46-3	48	40	15	7	7	7	7
A	strat46-4	48	40	15	7	7	7	7
A	strat46-5	48	40	14	7	7	7	7
A	final	48	40	15	7	7	7	7

continued on next page

Table S5 (continued)

Case	Run	Pool	S1	S2	S3	S4	S5	S6
B	outer1-inner1	38	30	10	5	5	5	5
B	outer1-inner2	38	30	10	5	5	5	5
B	outer1-inner3	38	30	11	5	5	5	5
B	outer2-inner1	38	30	12	6	6	6	6
B	outer2-inner2	38	30	10	5	5	5	5
B	outer2-inner3	38	30	11	5	5	5	5
B	outer3-inner1	38	30	11	5	5	5	5
B	outer3-inner2	38	30	10	5	5	5	5
B	outer3-inner3	38	30	11	5	5	5	5
B	outer4-inner1	38	30	12	6	6	6	6
B	outer4-inner2	38	30	12	6	6	6	6
B	outer4-inner3	38	30	11	5	5	5	5
B	outer5-inner1	38	30	12	6	6	6	6
B	outer5-inner2	38	30	11	5	5	5	5
B	outer5-inner3	38	30	11	5	5	5	5
B	outer1	38	30	10	5	5	5	5
B	outer2	38	30	11	5	5	5	5
B	outer3	38	30	11	5	5	5	5
B	outer4	38	30	11	5	5	5	5
B	outer5	38	30	11	5	5	5	5
B	strat42-1	38	30	11	5	5	5	5
B	strat42-2	38	30	11	5	5	5	5
B	strat42-3	38	30	11	5	5	5	5
B	strat42-4	38	30	12	6	6	6	6
B	strat42-5	38	30	10	5	5	5	5
B	strat43-1	38	30	11	5	5	5	5
B	strat43-2	38	30	11	5	5	5	5
B	strat43-3	38	30	11	5	5	5	5
B	strat43-4	38	30	11	5	5	5	5
B	strat43-5	38	30	10	5	5	5	5
B	strat44-1	38	30	11	5	5	5	5
B	strat44-2	38	30	11	5	5	5	5
B	strat44-3	38	30	11	5	5	5	5
B	strat44-4	38	30	11	5	5	5	5
B	strat44-5	38	30	11	5	5	5	5
B	strat45-1	38	30	11	5	5	5	5
B	strat45-2	38	30	11	5	5	5	5

continued on next page

Table S5 (continued)

Case	Run	Pool	S1	S2	S3	S4	S5	S6
B	strat45-3	38	30	11	5	5	5	5
B	strat45-4	38	30	11	5	5	5	5
B	strat45-5	38	30	10	5	5	5	5
B	strat46-1	38	30	11	5	5	5	5
B	strat46-2	38	30	11	5	5	5	5
B	strat46-3	38	30	11	5	5	5	5
B	strat46-4	38	30	11	5	5	5	5
B	strat46-5	38	30	10	5	5	5	5
B	final	38	30	11	5	5	5	5

Table S6: Per-fold selected feature sets from the training runs. `outer`k: input set of the outer-fold model of the first nested pass; `outer`k`-inner`j: input set used for hyperparameter tuning inside inner fold j of outer fold k; `strat`s-k: input set of fold k in stratified repeat with seed s; `final`: refit set used for the test-set evaluation. The sets are the stage-3 survivor sets of each run (guards bind, Table S5). Case A sets always contain cumulative_H_2, t, and N_C_2H_4 plus energy terms; Case B sets draw on the same energy and species terms without the H_2-history features.

Case	Selection run	Selected feature set
A	outer1-inner1	cumulative_H2, t, N_C2H4, running_Ea, E_a, energy_dissipated, E_pair
A	outer1-inner2	cumulative_H2, t, N_C2H4, energy_dissipated, E_b, E_a, E_p
A	outer1-inner3	cumulative_H2, t, N_C2H4, E_a, energy_dissipated, E_pair, N_C2H6
A	outer1	cumulative_H2, t, N_C2H4, energy_dissipated, E_a, running_Ea, E_pair
A	outer2-inner1	cumulative_H2, t, N_C2H4, E_b, E_p, E_a, energy_dissipated, N_CH4
A	outer2-inner2	cumulative_H2, t, N_C2H4, energy_dissipated, E_pair, running_Ea, E_a
A	outer2-inner3	cumulative_H2, t, N_C2H4, N_CH4, E_b, E_a, E_p
A	outer2	cumulative_H2, t, N_C2H4, E_a, N_CH4, E_p, E_b
A	outer3-inner1	cumulative_H2, t, N_C2H4, energy_dissipated, E_a, E_p, E_b
A	outer3-inner2	cumulative_H2, t, N_C2H4, energy_dissipated, E_a, E_pair, N_C2H6
A	outer3-inner3	cumulative_H2, t, N_C2H4, N_CH4, E_b, E_p, E_a
A	outer3	cumulative_H2, t, N_C2H4, energy_dissipated, E_p, E_b, E_a
A	outer4-inner1	cumulative_H2, t, N_C2H4, N_CH4, E_p, E_pair, E_a, E_b
A	outer4-inner2	cumulative_H2, t, N_C2H4, energy_dissipated, E_a, N_CH4, E_p, E_b
A	outer4-inner3	cumulative_H2, t, N_C2H4, E_v, E_b, N_CH4, E_a
A	outer4	cumulative_H2, t, N_C2H4, E_p, E_b, N_CH4, E_a
A	outer5-inner1	cumulative_H2, t, N_C2H4, energy_dissipated, E_b, E_v, E_a, E_p
A	outer5-inner2	cumulative_H2, t, N_C2H4, N_CH4, E_b, energy_dissipated, E_a

continued on next page

Table S6 (continued)

Case	Selection run	Selected feature set
A	outer5-inner3	cumulative_H2, t, N_C2H4, energy_dissipated, E_p, E_a, E_b
A	outer5	cumulative_H2, t, N_C2H4, N_CH4, E_p, E_b, E_a
A	strat42-1	cumulative_H2, t, N_C2H4, energy_dissipated, E_p, E_a, E_b
A	strat42-2	cumulative_H2, t, N_C2H4, E_p, E_b, N_CH4, E_a
A	strat42-3	cumulative_H2, t, N_C2H4, energy_dissipated, E_p, E_b, E_a
A	strat42-4	cumulative_H2, t, N_C2H4, E_v, E_p, N_CH4, E_b, E_a
A	strat42-5	cumulative_H2, t, N_C2H4, energy_dissipated, E_pair, N_CH4, E_a
A	strat43-1	cumulative_H2, t, N_C2H4, E_p, E_b, energy_dissipated, E_a
A	strat43-2	cumulative_H2, t, N_C2H4, energy_dissipated, E_a, E_p, E_b
A	strat43-3	cumulative_H2, t, N_C2H4, N_CH4, E_p, E_b, E_a
A	strat43-4	cumulative_H2, t, N_C2H4, energy_dissipated, E_a, E_p, E_b
A	strat43-5	cumulative_H2, t, N_C2H4, energy_dissipated, E_pair, N_CH4, E_a
A	strat44-1	cumulative_H2, t, N_C2H4, energy_dissipated, E_p, E_a, E_b
A	strat44-2	cumulative_H2, t, N_C2H4, E_b, E_p, N_CH4, E_a
A	strat44-3	cumulative_H2, t, N_C2H4, energy_dissipated, E_a, E_p, E_b
A	strat44-4	cumulative_H2, t, N_C2H4, energy_dissipated, E_v, E_pair, E_a
A	strat44-5	cumulative_H2, t, N_C2H4, energy_dissipated, E_p, E_b, E_a
A	strat45-1	cumulative_H2, t, N_C2H4, energy_dissipated, E_b, E_p, E_a
A	strat45-2	cumulative_H2, t, N_C2H4, energy_dissipated, E_p, E_a, E_b
A	strat45-3	cumulative_H2, t, N_C2H4, energy_dissipated, E_p, E_b, E_a
A	strat45-4	cumulative_H2, t, N_C2H4, N_CH4, E_p, E_b, E_a
A	strat45-5	cumulative_H2, t, N_C2H4, energy_dissipated, E_pair, N_CH4, E_a
A	strat46-1	cumulative_H2, t, N_C2H4, E_p, E_b, N_CH4, E_a
A	strat46-2	cumulative_H2, t, N_C2H4, energy_dissipated, E_p, E_a, E_b
A	strat46-3	cumulative_H2, t, N_C2H4, energy_dissipated, E_a, E_p, E_b
A	strat46-4	cumulative_H2, t, N_C2H4, N_CH4, E_p, E_b, E_a
A	strat46-5	cumulative_H2, t, N_C2H4, energy_dissipated, E_pair, running_Ea, E_a
A	final	cumulative_H2, t, N_C2H4, E_p, N_CH4, E_b, E_a
B	outer1-inner1	t, energy_dissipated, E_pair, E_a, N_C2H4
B	outer1-inner2	E_p, E_b, E_a, t, N_C2H4
B	outer1-inner3	E_pair, t, E_a, energy_dissipated, N_C2H4
B	outer1	energy_dissipated, t, E_pair, E_a, N_C2H4
B	outer2-inner1	E_p, E_b, E_a, t, N_C2H4, energy_dissipated
B	outer2-inner2	energy_dissipated, t, E_pair, E_a, N_C2H4
B	outer2-inner3	E_b, E_p, t, E_a, N_C2H4
B	outer2	E_b, E_p, E_a, t, N_C2H4
B	outer3-inner1	E_b, N_C2H4, E_p, E_a, t

continued on next page

Table S6 (continued)

Case	Selection run	Selected feature set
B	outer3-inner2	t, energy_dissipated, E_pair, N_C2H4, E_a
B	outer3-inner3	N_CH4, t, E_p, N_C2H4, E_b
B	outer3	E_p, E_b, E_a, t, N_C2H4
B	outer4-inner1	N_CH4, E_p, N_C2H4, t, E_b, E_pair
B	outer4-inner2	E_p, N_CH4, t, E_b, N_C2H4, E_a
B	outer4-inner3	E_v, t, E_b, N_CH4, N_C2H4
B	outer4	N_CH4, t, N_C2H4, E_p, E_b
B	outer5-inner1	E_v, t, E_b, E_p, N_C2H4, E_a
B	outer5-inner2	t, energy_dissipated, E_b, E_a, N_C2H4
B	outer5-inner3	E_b, E_p, t, E_a, N_C2H4
B	outer5	N_CH4, N_C2H4, t, E_b, E_p
B	strat42-1	E_p, E_a, E_b, t, N_C2H4
B	strat42-2	E_p, E_b, E_a, N_C2H4, t
B	strat42-3	E_p, E_b, E_a, N_C2H4, t
B	strat42-4	E_v, t, E_p, E_b, N_C2H4, E_a
B	strat42-5	N_CH4, N_C2H4, t, E_pair, E_a
B	strat43-1	E_p, E_b, E_a, N_C2H4, t
B	strat43-2	E_b, E_p, N_C2H4, E_a, t
B	strat43-3	E_p, E_b, E_a, N_C2H4, t
B	strat43-4	E_p, E_b, N_C2H4, t, E_a
B	strat43-5	N_CH4, N_C2H4, t, E_pair, E_a
B	strat44-1	E_b, E_p, N_C2H4, E_a, t
B	strat44-2	E_p, E_b, E_a, t, N_C2H4
B	strat44-3	E_p, E_b, t, N_C2H4, E_a
B	strat44-4	E_v, E_pair, t, E_a, N_C2H4
B	strat44-5	E_p, E_b, E_a, t, N_C2H4
B	strat45-1	E_p, E_b, E_a, t, N_C2H4
B	strat45-2	E_p, E_a, E_b, t, N_C2H4
B	strat45-3	E_b, E_p, E_a, t, N_C2H4
B	strat45-4	E_b, E_p, E_a, N_C2H4, t
B	strat45-5	N_CH4, N_C2H4, t, E_pair, E_a
B	strat46-1	E_p, E_a, E_b, t, N_C2H4
B	strat46-2	E_b, E_p, N_C2H4, E_a, t
B	strat46-3	E_p, E_b, N_C2H4, t, E_a
B	strat46-4	E_p, E_b, E_a, N_C2H4, t
B	strat46-5	energy_dissipated, t, E_pair, N_C2H4, E_a
B	final	E_p, E_b, E_a, N_C2H4, t

Table S7: Selection frequency of every candidate feature across the fold-level selections: the 5 first-pass outer folds, the 15 inner folds (3 per outer fold), the 25 stratified-repeat folds (5 folds × 5 seeds), and the final refit. Only 12 of the 48 Case A features and 9 of the 38 Case B features are ever selected; the remaining features of the pool are never chosen (shown for completeness). "yes": member of the final refit set.

Case	Feature	Outer folds	Inner folds	Stratified folds	Final refit
A	E_a	5	15	25	yes
A	N_C2H4	5	15	25	yes
A	cumulative_H2	5	15	25	yes
A	t	5	15	25	yes
A	E_b	4	11	20	yes
A	E_p	4	9	20	yes
A	energy_dissipated	2	11	18	—
A	N_CH4	3	7	10	yes
A	E_pair	1	5	5	—
A	E_v	0	2	2	—
A	running_Ea	1	2	1	—
A	N_C2H6	0	2	0	—
A	C_conv	0	0	0	—
A	Coulomb_VdW_ratio	0	0	0	—
A	E_co	0	0	0	—
A	E_coa	0	0	0	—
A	E_hb	0	0	0	—
A	E_lp	0	0	0	—
A	E_pen	0	0	0	—
A	E_qeq	0	0	0	—
A	E_t	0	0	0	—
A	E_tot	0	0	0	—
A	E_w	0	0	0	—
A	H2_selectivity	0	0	0	—
A	H_C_ratio	0	0	0	—
A	Hbond_bond_ratio	0	0	0	—
A	N_C2H2	0	0	0	—
A	N_CO2	0	0	0	—
A	N_H2_lag1	0	0	0	—
A	N_H2_lag2	0	0	0	—
A	N_H_radical	0	0	0	—
A	N_OH_radical	0	0	0	—
A	O_C_ratio	0	0	0	—

continued on next page

Table S7 (continued)

Case	Feature	Outer folds	Inner folds	Stratified folds	Final refit
A	P	0	0	0	—
A	T	0	0	0	—
A	WGS_Keq	0	0	0	—
A	bond_fraction	0	0	0	—
A	charge_fraction	0	0	0	—
A	conformational_frac	0	0	0	—
A	dC_conv_dt	0	0	0	—
A	dE_b_dt	0	0	0	—
A	dE_qeq_dt	0	0	0	—
A	dN_H2	0	0	0	—
A	dN_H2_dt	0	0	0	—
A	rho	0	0	0	—
A	rolling_mean_H2	0	0	0	—
A	rolling_std_H2	0	0	0	—
A	O2_loading	0	0	0	—
B	N_C2H4	5	15	25	yes
B	t	5	15	25	yes
B	E_a	3	12	25	yes
B	E_b	4	11	20	yes
B	E_p	4	9	20	yes
B	E_pair	1	5	5	—
B	N_CH4	2	4	3	—
B	energy_dissipated	1	6	1	—
B	E_v	0	2	2	—
B	C_conv	0	0	0	—
B	Coulomb_VdW_ratio	0	0	0	—
B	E_co	0	0	0	—
B	E_coa	0	0	0	—
B	E_hb	0	0	0	—
B	E_lp	0	0	0	—
B	E_pen	0	0	0	—
B	E_qeq	0	0	0	—
B	E_t	0	0	0	—
B	E_tot	0	0	0	—
B	E_w	0	0	0	—
B	Hbond_bond_ratio	0	0	0	—
B	N_C2H2	0	0	0	—

continued on next page

Table S7 (continued)

Case	Feature	Outer folds	Inner folds	Stratified folds	Final refit
B	N_C2H6	0	0	0	—
B	N_CO2	0	0	0	—
B	N_H_radical	0	0	0	—
B	N_OH_radical	0	0	0	—
B	O_C_ratio	0	0	0	—
B	P	0	0	0	—
B	T	0	0	0	—
B	WGS_Keq	0	0	0	—
B	bond_fraction	0	0	0	—
B	charge_fraction	0	0	0	—
B	conformational_frac	0	0	0	—
B	dC_conv_dt	0	0	0	—
B	dE_b_dt	0	0	0	—
B	dE_qeq_dt	0	0	0	—
B	rho	0	0	0	—
B	O2_loading	0	0	0	—

Table S8: Stage-wise survival of the temperature (T), the O_2 loading ($n(O_2)$), and the simulation time (t) through the six-stage cascade, in every fold-level selection run (31 replicable runs per case: 5 first-pass outer folds, 25 stratified-repeat folds, and the final refit). "S2": eliminated by the stage-2 Pearson filter ($|\rho| > 0.8$ with a higher-variance member of the pool): T is collinear with the conjugation energy E_co ($\rho = 0.83$) and $n(O_2)$ with the pair ($\rho = 0.98$), total ($\rho = 0.96$), and bond ($\rho = 0.86$) energies; "selected": survives the full cascade and enters the fold's selected set. t is retained in all 31 runs of both cases.

Case	Selection run	T	$n(O_2)$	t
A	outer fold 1	S2	S2	selected
A	outer fold 2	S2	S2	selected
A	outer fold 3	S2	S2	selected
A	outer fold 4	S2	S2	selected
A	outer fold 5	S2	S2	selected
A	strat42 (folds 1–5)	S2 (5/5)	S2 (5/5)	selected (5/5)
A	strat43 (folds 1–5)	S2 (5/5)	S2 (5/5)	selected (5/5)
A	strat44 (folds 1–5)	S2 (5/5)	S2 (5/5)	selected (5/5)

continued on next page

Table S8 (continued)

Case	Selection run	T	$n(O_2)$	t
A	strat45 (folds 1–5)	S2 (5/5)	S2 (5/5)	selected (5/5)
A	strat46 (folds 1–5)	S2 (5/5)	S2 (5/5)	selected (5/5)
A	final refit	S2	S2	selected
B	outer fold 1	S2	S2	selected
B	outer fold 2	S2	S2	selected
B	outer fold 3	S2	S2	selected
B	outer fold 4	S2	S2	selected
B	outer fold 5	S2	S2	selected
B	strat42 (folds 1–5)	S2 (5/5)	S2 (5/5)	selected (5/5)
B	strat43 (folds 1–5)	S2 (5/5)	S2 (5/5)	selected (5/5)
B	strat44 (folds 1–5)	S2 (5/5)	S2 (5/5)	selected (5/5)
B	strat45 (folds 1–5)	S2 (5/5)	S2 (5/5)	selected (5/5)
B	strat46 (folds 1–5)	S2 (5/5)	S2 (5/5)	selected (5/5)
B	final refit	S2	S2	selected

S6. Models and hyperparameter tuning

The eight machine-learning models are multiple linear regression (MLR), decision tree (DT), random forest (RF), XGBoost, CatBoost, artificial neural network (ANN), support vector regression (SVR), and Gaussian process regression (GPR); all scikit-learn estimators are used through the training pipeline. The three baselines are the persistence baseline (Section S4) and MLR/RF restricted to the three physical descriptors T, the O_2 loading, and t. The inputs of only the ANN, SVR, and GPR are standardized by a fold-fitted `StandardScaler` pipeline; tree models and MLR receive raw features. GPR and SVR fits use the per-simulation subsampling of Section S4 ($S = 2000$ and 5000 rows, respectively).

Hyperparameters were selected *once*, in the first nested pass of the grouped cross-validation (outer 5-fold `GroupKFold` over simulation groups; inner 3-fold `GroupKFold`;

scoring: negative root-mean-square error), and were then reused unchanged for the stratified repeats (seeds 42–46). Within each inner-fold search, grids with at most 100 combinations are enumerated exhaustively; larger grids are reduced to 20 combinations drawn without replacement (NumPy `RandomState(42)`; the fixed seed implies the same 20 combinations in every search of a given grid). The winning combination of each outer fold was combined across the five outer folds by majority vote, with ties broken by the mean inner-CV neg-RMSE and then by first occurrence. Nested tuning with all selection confined to the inner folds guards against the model-selection overfitting and subsequent evaluation bias that arise when hyperparameters and performance are estimated on the same data. No hyperparameter was re-tuned on evaluation data; the selected values (Table S11) were frozen before the single test-set evaluation (Section S7).

Table S9 reproduces the full tuning grids verbatim from the training code; Table S10 summarizes the grid sizes, the number of evaluated combinations per search, and the vote counts behind each selection; Table S11 lists the selected values for both cases, transcribed from the selection file (`best_params.json`).

Table S9: Hyperparameter tuning grids, transcribed verbatim from the training code (`R1_common.py`, function `param_grids`, non-smoke mode). Model abbreviations as in the main text. The kernel tags `RBF+WhiteKernel` and `Matern+WhiteKernel` denote the scikit-learn kernels `RBF() + WhiteKernel()` and `Matern() + WhiteKernel()`, respectively; `None` denotes an unlimited setting (tree depth or feature count). MLR (multiple linear regression) has no tunable hyperparameters, so its candidate list contains a single empty configuration. During tuning, grids with more than 100 combinations are not enumerated exhaustively; instead 20 combinations are drawn without replacement per search (NumPy `RandomState(42)`; the seed is fixed, so the same 20 combinations are evaluated in every search of a given grid).

Model	Hyperparameter	Candidate values
MLR	—	none (empty grid; the model has no tunable hyperparameters)
DT	`max_depth`	3, 5, 10, 15, 20, 30, `None`
	`min_samples_split`	2, 5, 10, 20

continued on next page

Table S9 (continued)

Model	Hyperparameter	Candidate values
	min_samples_leaf	1, 2, 5, 10
RF	n_estimators	50, 100, 200, 500
	max_depth	5, 10, 20, 50, None
	max_features	sqrt, log2, None
	min_samples_leaf	1, 2, 5, 10
XGBoost	n_estimators	50, 100, 200, 500
	max_depth	3, 5, 7, 10, 15
	learning_rate	0.01, 0.05, 0.1, 0.2, 0.3
	subsample	0.6, 0.8, 1.0
	colsample_bytree	0.6, 0.8, 1.0
	reg_alpha	0, 1, 10
	reg_lambda	0, 1, 10
CatBoost	iterations	50, 100, 200, 500
	depth	4, 6, 8, 10
	learning_rate	0.01, 0.05, 0.1, 0.2, 0.3
	l2_leaf_reg	1, 3, 5, 10
ANN	hidden_layer_sizes	(64), (128), (64, 64), (128, 64), (128, 128, 64)
	activation	relu, tanh
	alpha	1×10^{-4}, 1×10^{-3}, 0.01
	learning_rate	constant, adaptive
	max_iter	500, 1000, 2000
SVR	C	1, 10, 100
	gamma	scale, auto, 1×10^{-3}, 0.1
	kernel	rbf
	epsilon	0.05, 0.1, 0.5

continued on next page

Table S9 (continued)

Model	Hyperparameter	Candidate values
GPR	`kernel`	`RBF+WhiteKernel`, `Matern+WhiteKernel`
	`alpha`	1×10^{-6}, 1×10^{-4}, 0.01
	`n_restarts_optimizer`	0

Table S10: Summary of hyperparameter selection. Hyperparameters were selected once, in the first nested pass (outer 5-fold `GroupKFold` over simulation groups; inner 3-fold `GroupKFold`; scoring: negative root-mean-square error), and were then reused unchanged for the stratified repeats (seeds 42–46). Grid size: number of combinations in the grid of Table S9. Evaluated per search: combinations actually evaluated in each grid search (an inner fold of an outer fold); grids with at most 100 combinations were enumerated exhaustively, and larger grids were reduced to 20 combinations drawn without replacement (NumPy `RandomState(42)`). The winning combination of each outer fold was combined across the five outer folds by majority vote; ties were broken by the mean inner-CV neg-RMSE and then by first occurrence. Votes: number of outer folds (of five) whose winning combination equals the recorded one; for MLR the vote is vacuous because the model has no tunable hyperparameters (the value 5 is recorded in the selection file and shown for completeness). The selected combinations are listed in Table S11.

Model	Grid size	Evaluated per search	Votes (case A)	Votes (case B)
MLR	—	no tuning	5	5
DT	112	20 of 112	2	1
RF	240	20 of 240	3	2
XGBoost	8100	20 of 8100	2	2
CatBoost	320	20 of 320	3	3
ANN	180	20 of 180	4	1
SVR	36	all 36	3	2
GPR	6	all 6	5	5

Table S11: Hyperparameter values selected by the first nested pass of the outer/inner cross-validation and reused unchanged for the stratified repeats (seeds 42–46), transcribed from the selection file (`best_params.json`). Case A and case B refer to the two prediction targets of the dataset (Section S4). Kernel notation as in Table S9; `None` denotes an unlimited setting. The vote counts behind each selection are given in Table S10.

Model	Hyperparameter	Case A	Case B
MLR	no tunable hyperparameters (`LinearRegression`)		
DT	`max_depth`	10	30
	`min_samples_split`	20	2
	`min_samples_leaf`	10	1
RF	`n_estimators`	100	200
	`max_depth`	10	20
	`max_features`	`None`	`log2`
	`min_samples_leaf`	10	1
XGBoost	`n_estimators`	100	200
	`max_depth`	10	3
	`learning_rate`	0.1	0.2
	`subsample`	0.8	0.8
	`colsample_bytree`	1.0	1.0
	`reg_alpha`	1	0
	`reg_lambda`	10	10
CatBoost	`iterations`	500	500
	`depth`	4	4
	`learning_rate`	0.1	0.2
	`l2_leaf_reg`	10	1
ANN	`hidden_layer_sizes`	(128, 128, 64)	(128, 64)
	`activation`	`relu`	`relu`
	`alpha`	1×10^{-4}	1×10^{-3}

continued on next page

Table S11 (continued)

Model	Hyperparameter	Case A	Case B
	`learning_rate`	`constant`	`adaptive`
	`max_iter`	500	500
SVR	`C`	100	100
	`gamma`	0.1	`scale`
	`kernel`	`rbf`	`rbf`
	`epsilon`	0.5	0.05
GPR	`kernel`	Matern() + WhiteKernel()	Matern() + WhiteKernel()
	`alpha`	1×10^{-6}	0.01
	`n_restarts_optimizer`	0	0

S7. Per-simulation model metrics

For each model and each simulation, the four metrics are computed over that simulation's primary-horizon ($h = 25$ ps) rows: the coefficient of determination R^2, the root-mean-square error (RMSE, molecule·ps), the mean absolute error (MAE, molecule·ps), and the mean absolute percentage error (MAPE, %), the latter with an absolute floor of $\varepsilon = 1$ molecule·ps in the denominator. For degenerate simulations whose target is constant (the zero-H_2 trajectories; $ss_{tot} < 10^{-12}$), R^2 is reported as 1 when the sum of squared residuals is also zero and as 0 otherwise.

Table S12 lists the per-simulation *out-of-fold* metrics on the 36 training simulations for all six cross-validation passes, namely the first nested pass plus the five stratified repeats (seeds 42–46; feature selection re-run per fold, hyperparameters fixed), for the 11 models (8 machine-learning models + 3 baselines) and both cases: $2 \times 11 \times 6 \times 36 = 4752$ rows. Out-of-fold predictions exist only for training-partition simulations; test-partition simulations have none by construction. Table S13 lists the per-simulation metrics of the single test-set

evaluation: the final refit models (majority-vote hyperparameters of Table S11 and the final refit feature sets of Table S6) applied once to each of the 9 test simulations, $2 \times 11 \times 9 = 198$ rows.

Figures S1–S3 visualise the stability of these metrics. Figure S1 shows the pooled out-of-fold RMSE of every model on each of the five stratified grouped 5-fold repeats (seeds 42–46), and Figures S2 and S3 show the per-simulation distributions of the first-pass out-of-fold RMSE for Case A and Case B, respectively; in both box plots the dashed reference line marks the median per-simulation persistence RMSE (739.3 molecules·ps).

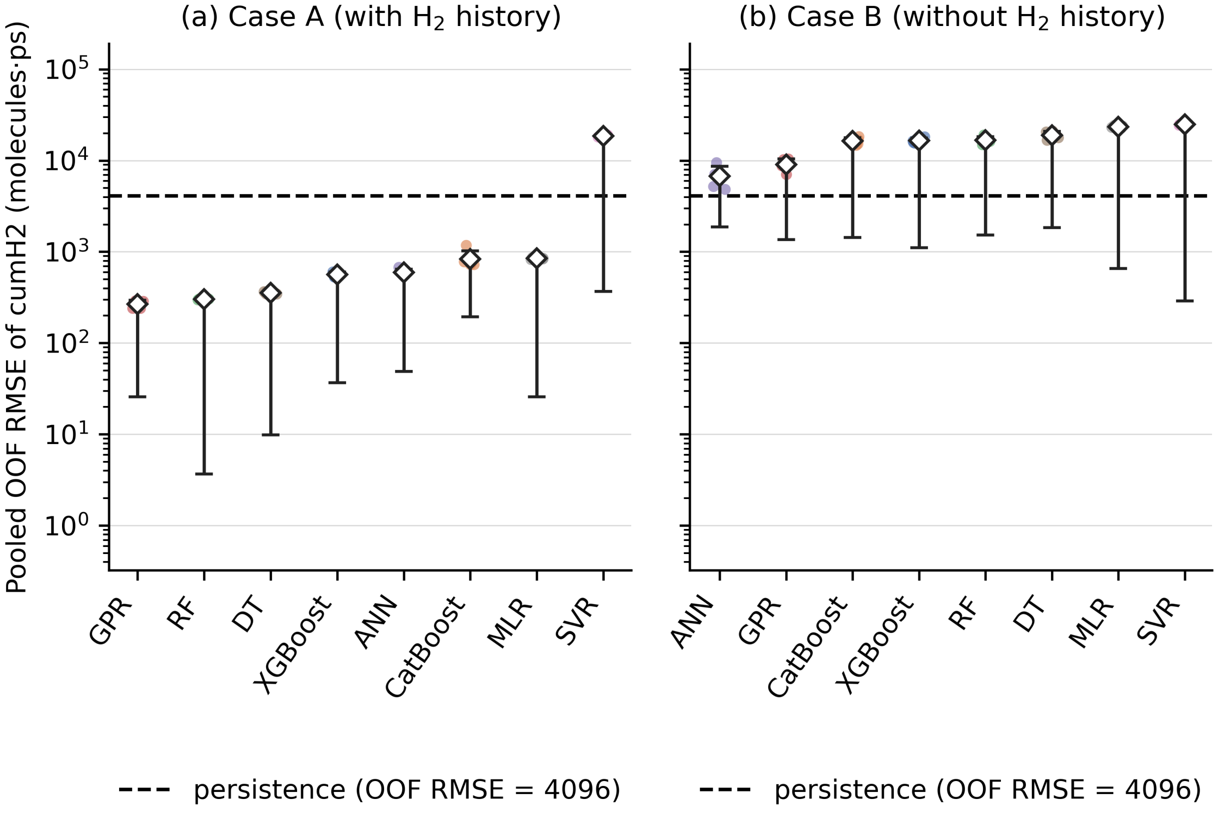

Figure S1: Stability of the nested cross-validation benchmark over the five stratified repeats (seeds 42–46) for (a) Case A and (b) Case B: jittered points show the per-repeat pooled OOF RMSE, diamonds the repeat means, and error bars the ±1 standard deviation. The dashed line marks the persistence score (4096 molecules·ps), constant across repeats by construction; note the logarithmic axis.

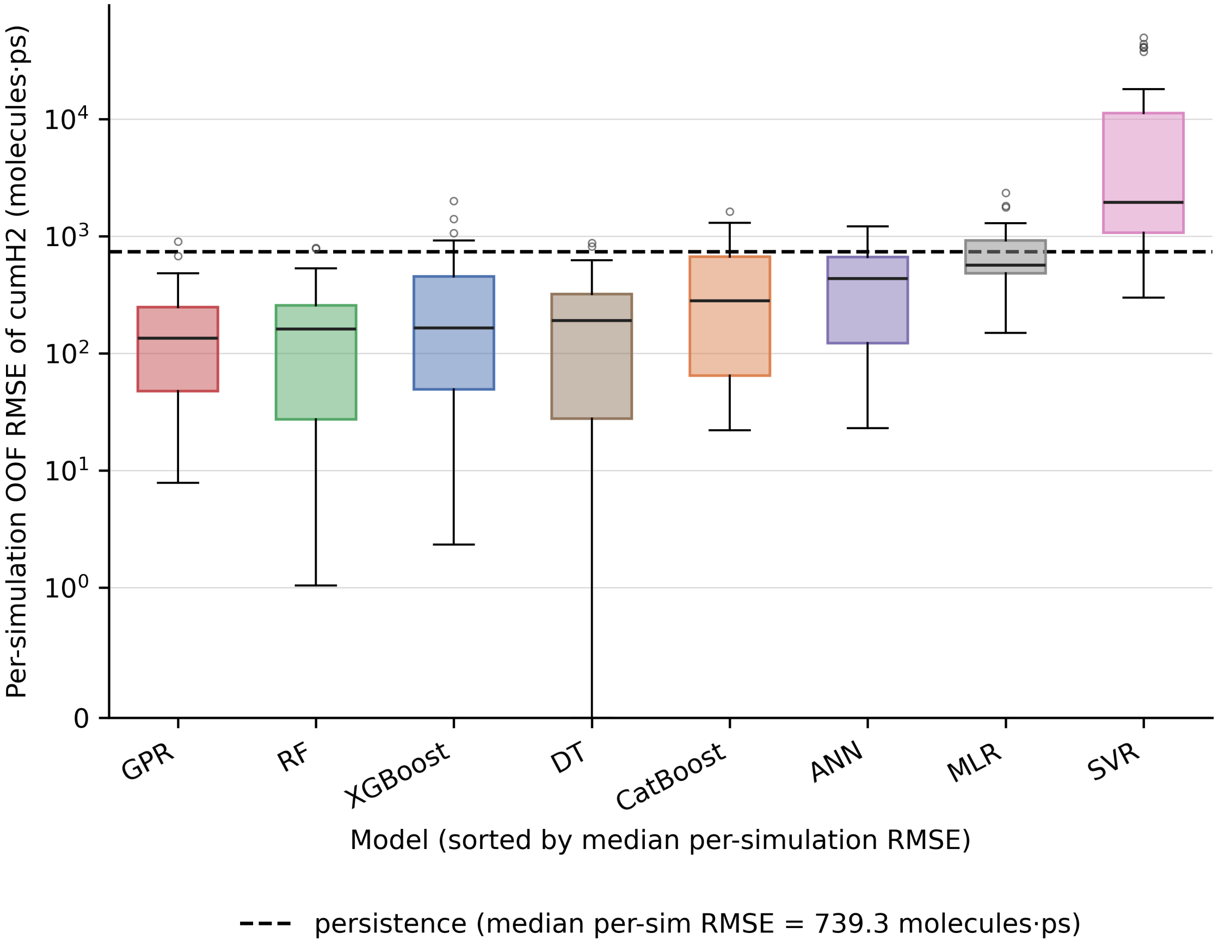

Figure S2: Per-simulation OOF RMSE for Case A (with the H_2 history): each box summarises the 36 training simulations of the first-pass nested cross-validation, with models sorted by median; the dashed line marks the median per-simulation persistence RMSE (739.3 molecules·ps). A symlog axis (linear below 1) accommodates per-simulation RMSE values from exact zeros to $\sim 6 \times 10^4$ molecules·ps.

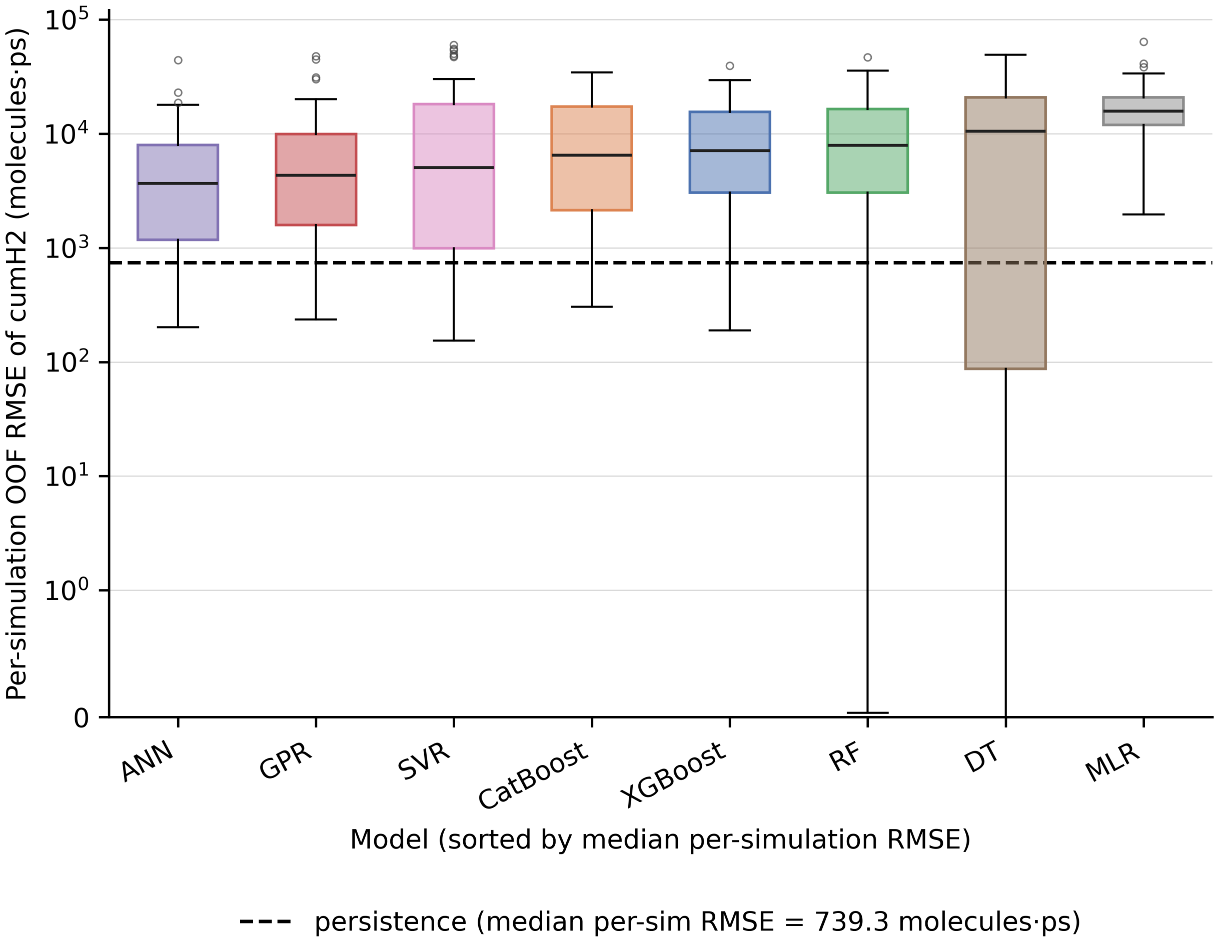

Figure S3: Per-simulation OOF RMSE for Case B (without the H_2 history), in the format of Figure S2: the dashed persistence line (739.3 molecules·ps) lies below every ML box, and the best ML median (ANN, 3667 molecules·ps) sits almost five times above it.

Table S12: Per-simulation out-of-fold metrics of the 11 models (8 machine-learning models and the 3 baselines) on the 36 train simulations, for each of the six cross-validation passes: the first nested pass and the five stratified-repeat passes (seeds 42–46). First pass: 5-fold `GroupKFold`; each outer fold uses its own fold-specific feature set and its own inner-tuned hyperparameters. Stratified repeats: 5-fold `StratifiedGroupKFold` (shuffled, stratified by temperature); each fold uses its own fold-specific feature set and the fixed majority-vote hyperparameters of Table S11. R^2: coefficient of determination on the simulation's out-of-fold predictions (degenerate convention for zero-H_2 simulations with $ss_{tot} < 10^{-12}$: $R^2 = 1$ if the prediction is exact, $R^2 = 0$ otherwise); RMSE and MAE in molecule·ps; MAPE in % with a floor of $\epsilon = 1$ molecule·ps on $|y|$ in the denominator. Values are displayed rounded.

Simulation	$n(O_2)$	T (K)	n_{rows}	R^2	RMSE (molecule·ps)	MAE (molecule·ps)	MAPE (%)
**Repeat: firstpass Case: A Model: MLR**							
f-50-1_T1700	1	1700	357	0.0000	247.0	208.7	20,869
f-50-1_T1900	1	1900	1318	0.0000	149.7	123.4	12,336
f-50-1_T2100	1	2100	1816	0.4794	235.1	233.7	2276
f-50-1_T2500	1	2500	1892	0.9969	483.9	418.7	136.0
f-50-1_T2700	1	2700	1893	0.9987	789.8	567.5	18.0
f-50-1_T2900	1	2900	1892	0.9980	1820	1575	7.6
f-50-100_T1500	100	1500	175	0.0000	568.7	482.6	48,263
f-50-100_T1700	100	1700	663	0.0000	493.5	484.1	48,408
f-50-100_T1900	100	1900	1458	-1423.7	501.3	422.4	33,285
f-50-100_T2100	100	2100	1827	0.7557	506.2	429.9	1478
f-50-100_T2500	100	2500	1891	0.9972	568.6	518.2	66.3
f-50-100_T2700	100	2700	1893	0.9994	514.0	424.8	25.5
f-50-100_T2900	100	2900	1893	0.9991	1292	1162	7.0
f-50-300_T1500	300	1500	408	0.0000	541.7	446.3	44,630
f-50-300_T1700	300	1700	1215	0.0000	586.3	499.8	49,979
f-50-300_T1900	300	1900	1562	-512,466.0	479.3	415.5	40,500
f-50-300_T2100	300	2100	1869	0.3571	391.7	352.4	10,345
f-50-300_T2300	300	2300	1889	0.9775	494.5	482.1	273.1
f-50-300_T2500	300	2500	1894	0.9966	659.8	613.4	642.4
f-50-300_T2700	300	2700	1893	0.9980	1177	1036	27.0
f-50-300_T2900	300	2900	1895	0.9973	2359	2051	10.5
f-50-500_T1500	500	1500	434	0.0000	727.9	591.0	59,099
f-50-500_T2100	500	2100	1886	0.6196	420.6	402.6	4648
f-50-500_T2300	500	2300	1891	0.9658	737.9	714.6	295.4
f-50-500_T2500	500	2500	1892	0.9946	746.6	686.2	35.1
f-50-500_T2700	500	2700	1895	0.9997	439.5	381.2	19.3

continued on next page

Table S12 (continued)

Simulation	$n(O_2)$	T (K)	n_{rows}	R^2	RMSE (molecule·ps)	MAE (molecule·ps)	MAPE (%)
f-50-500_T2900	500	2900	1893	0.9991	1268	1064	5.5
f-50-700_T1700	700	1700	1154	0.0000	1172	1090	108,994
f-50-700_T1900	700	1900	1812	-6.5266	440.4	385.0	15,267
f-50-700_T2100	700	2100	1874	0.9052	211.4	177.7	1006
f-50-700_T2500	700	2500	1894	0.9945	839.1	786.2	21.7
f-50-700_T2700	700	2700	1893	0.9994	547.0	434.6	12.6
f-50-700_T2900	700	2900	1894	0.9991	1250	1036	8.0
f-50-900_T2100	900	2100	1876	0.5673	565.1	485.6	11,361
f-50-900_T2500	900	2500	1884	0.9849	1192	1077	104.5
f-50-900_T2900	900	2900	1893	0.9982	1765	1553	34.7
**Repeat: firstpass Case: A Model: DT**							
f-50-1_T1700	1	1700	357	1.0000	0.0	0.0	0.0
f-50-1_T1900	1	1900	1318	0.0000	30.9	14.0	1403
f-50-1_T2100	1	2100	1816	0.8979	104.2	72.5	141.2
f-50-1_T2500	1	2500	1892	0.9989	292.7	240.8	11.6
f-50-1_T2700	1	2700	1893	0.9998	307.6	236.5	5.6
f-50-1_T2900	1	2900	1892	0.9995	876.1	647.0	2.3
f-50-100_T1500	100	1500	175	0.0000	2.3	0.4	36.9
f-50-100_T1700	100	1700	663	0.0000	2.2	2.0	202.9
f-50-100_T1900	100	1900	1458	0.8895	4.4	1.4	11.4
f-50-100_T2100	100	2100	1827	0.9178	293.6	166.7	65.8
f-50-100_T2500	100	2500	1891	0.9993	291.0	222.2	6.3
f-50-100_T2700	100	2700	1893	0.9997	370.7	290.7	4.8
f-50-100_T2900	100	2900	1893	0.9998	612.8	413.8	2.0
f-50-300_T1500	300	1500	408	0.0000	1.5	1.5	149.0
f-50-300_T1700	300	1700	1215	0.0000	1.7	0.3	30.7
f-50-300_T1900	300	1900	1562	-754.5	18.4	13.1	1279
f-50-300_T2100	300	2100	1869	0.9672	88.5	69.4	44.5
f-50-300_T2300	300	2300	1889	0.9954	223.5	191.2	18.1
f-50-300_T2500	300	2500	1894	0.9997	189.6	152.0	49.7
f-50-300_T2700	300	2700	1893	0.9995	581.3	378.9	6.8
f-50-300_T2900	300	2900	1895	0.9997	818.4	464.6	2.5
f-50-500_T1500	500	1500	434	0.0000	0.0	0.0	1.6
f-50-500_T2100	500	2100	1886	0.9835	87.6	64.8	116.9
f-50-500_T2300	500	2300	1891	0.9936	318.5	238.9	42.3
f-50-500_T2500	500	2500	1892	0.9996	192.6	151.0	7.5

continued on next page

Table S12 (continued)

Simulation	$n(O_2)$	T (K)	n_{rows}	R^2	RMSE (molecule·ps)	MAE (molecule·ps)	MAPE (%)
f-50-500_T2700	500	2700	1895	0.9994	624.6	403.9	7.6
f-50-500_T2900	500	2900	1893	0.9999	341.9	286.6	3.4
f-50-700_T1700	700	1700	1154	0.0000	1.0	0.3	26.9
f-50-700_T1900	700	1900	1812	0.7444	81.2	57.9	29.0
f-50-700_T2100	700	2100	1874	0.9862	80.7	54.7	79.1
f-50-700_T2500	700	2500	1894	0.9996	224.6	143.7	9.7
f-50-700_T2700	700	2700	1893	0.9996	437.3	365.2	6.5
f-50-700_T2900	700	2900	1894	1.0000	259.9	200.1	4.2
f-50-900_T2100	900	2100	1876	0.9540	184.3	95.2	291.6
f-50-900_T2500	900	2500	1884	0.9997	181.3	135.6	12.6
f-50-900_T2900	900	2900	1893	0.9999	328.8	243.8	6.4
**Repeat: firstpass Case: A Model: RF**							
f-50-1_T1700	1	1700	357	0.0000	1.0	1.0	104.1
f-50-1_T1900	1	1900	1318	0.0000	19.0	10.1	1005
f-50-1_T2100	1	2100	1816	0.9644	61.5	49.4	30.7
f-50-1_T2500	1	2500	1892	0.9983	357.9	273.5	8.0
f-50-1_T2700	1	2700	1893	0.9998	277.0	226.9	3.8
f-50-1_T2900	1	2900	1892	0.9996	798.8	607.4	2.1
f-50-100_T1500	100	1500	175	0.0000	2.5	1.6	159.7
f-50-100_T1700	100	1700	663	0.0000	3.0	2.2	216.7
f-50-100_T1900	100	1900	1458	-0.3455	15.4	6.4	146.4
f-50-100_T2100	100	2100	1827	0.9385	254.0	135.3	61.2
f-50-100_T2500	100	2500	1891	0.9997	171.7	125.7	6.4
f-50-100_T2700	100	2700	1893	0.9999	205.4	181.3	3.9
f-50-100_T2900	100	2900	1893	0.9999	532.4	385.9	2.8
f-50-300_T1500	300	1500	408	0.0000	1.5	1.5	147.9
f-50-300_T1700	300	1700	1215	0.0000	2.0	0.3	25.5
f-50-300_T1900	300	1900	1562	-2019.7	30.1	19.9	1922
f-50-300_T2100	300	2100	1869	0.9777	73.0	57.0	85.0
f-50-300_T2300	300	2300	1889	0.9958	213.9	189.6	17.6
f-50-300_T2500	300	2500	1894	0.9998	165.5	141.7	40.5
f-50-300_T2700	300	2700	1893	0.9997	428.5	294.2	4.6
f-50-300_T2900	300	2900	1895	0.9997	786.1	362.7	2.7
f-50-500_T1500	500	1500	434	0.0000	1.7	1.3	126.4
f-50-500_T2100	500	2100	1886	0.9925	58.9	45.4	103.9
f-50-500_T2300	500	2300	1891	0.9961	249.9	198.6	37.3

continued on next page

Table S12 (continued)

Simulation	$n(O_2)$	T (K)	n_{rows}	R^2	RMSE (molecule·ps)	MAE (molecule·ps)	MAPE (%)
f-50-500_T2500	500	2500	1892	0.9998	157.6	136.0	6.3
f-50-500_T2700	500	2700	1895	0.9996	493.4	330.2	6.8
f-50-500_T2900	500	2900	1893	1.0000	268.8	241.3	3.0
f-50-700_T1700	700	1700	1154	0.0000	3.1	1.5	150.2
f-50-700_T1900	700	1900	1812	0.8849	54.5	39.5	49.0
f-50-700_T2100	700	2100	1874	0.9975	34.0	26.0	65.4
f-50-700_T2500	700	2500	1894	0.9997	194.9	102.1	7.3
f-50-700_T2700	700	2700	1893	0.9998	338.9	288.5	5.4
f-50-700_T2900	700	2900	1894	1.0000	197.9	156.6	3.2
f-50-900_T2100	900	2100	1876	0.9664	157.5	80.5	301.3
f-50-900_T2500	900	2500	1884	0.9999	99.3	76.1	8.8
f-50-900_T2900	900	2900	1893	1.0000	232.0	176.9	5.0
**Repeat: firstpass Case: A Model: XGBoost**							
f-50-1_T1700	1	1700	357	0.0000	81.5	81.5	8153
f-50-1_T1900	1	1900	1318	0.0000	12.8	9.2	920.3
f-50-1_T2100	1	2100	1816	0.8452	128.2	122.5	705.9
f-50-1_T2500	1	2500	1892	0.9988	299.3	231.4	14.5
f-50-1_T2700	1	2700	1893	0.9996	429.9	320.8	5.4
f-50-1_T2900	1	2900	1892	0.9988	1411	842.6	2.1
f-50-100_T1500	100	1500	175	0.0000	28.0	22.4	2242
f-50-100_T1700	100	1700	663	0.0000	7.6	6.1	613.6
f-50-100_T1900	100	1900	1458	-1.4007	20.6	13.2	1020
f-50-100_T2100	100	2100	1827	0.9910	97.4	78.3	64.9
f-50-100_T2500	100	2500	1891	0.9995	248.6	191.1	14.7
f-50-100_T2700	100	2700	1893	0.9997	404.3	322.5	4.2
f-50-100_T2900	100	2900	1893	0.9994	1065	800.5	5.0
f-50-300_T1500	300	1500	408	0.0000	2.3	0.3	33.4
f-50-300_T1700	300	1700	1215	0.0000	3.2	0.6	57.7
f-50-300_T1900	300	1900	1562	-4005.9	42.4	25.0	2370
f-50-300_T2100	300	2100	1869	0.9365	123.1	83.8	372.1
f-50-300_T2300	300	2300	1889	0.9889	347.3	257.3	43.4
f-50-300_T2500	300	2500	1894	0.9998	173.0	139.6	42.5
f-50-300_T2700	300	2700	1893	0.9989	883.0	574.9	8.0
f-50-300_T2900	300	2900	1895	0.9981	2006	1261	2.8
f-50-500_T1500	500	1500	434	0.0000	17.1	10.6	1060
f-50-500_T2100	500	2100	1886	0.9942	51.7	40.6	377.8

continued on next page

Table S12 (continued)

Simulation	$n(O_2)$	T (K)	n_{rows}	R^2	RMSE (molecule·ps)	MAE (molecule·ps)	MAPE (%)
f-50-500_T2300	500	2300	1891	0.9985	155.7	115.8	27.3
f-50-500_T2500	500	2500	1892	0.9997	181.6	154.1	6.3
f-50-500_T2700	500	2700	1895	0.9996	524.3	355.2	5.6
f-50-500_T2900	500	2900	1893	0.9995	923.6	498.2	3.0
f-50-700_T1700	700	1700	1154	0.0000	3.5	2.1	209.5
f-50-700_T1900	700	1900	1812	0.3395	130.5	122.5	1909
f-50-700_T2100	700	2100	1874	0.9933	56.2	39.9	59.9
f-50-700_T2500	700	2500	1894	0.9998	156.7	124.9	4.9
f-50-700_T2700	700	2700	1893	0.9993	570.7	435.7	5.1
f-50-700_T2900	700	2900	1894	0.9997	687.8	435.5	3.3
f-50-900_T2100	900	2100	1876	0.9037	266.5	219.7	376.6
f-50-900_T2500	900	2500	1884	0.9995	223.4	185.9	13.1
f-50-900_T2900	900	2900	1893	0.9995	900.8	710.3	5.6
**Repeat: firstpass Case: A Model: CatBoost**							
f-50-1_T1700	1	1700	357	0.0000	22.1	18.6	1858
f-50-1_T1900	1	1900	1318	0.0000	45.7	38.8	3885
f-50-1_T2100	1	2100	1816	0.9262	88.5	67.2	237.2
f-50-1_T2500	1	2500	1892	0.9987	318.3	255.7	14.5
f-50-1_T2700	1	2700	1893	0.9986	803.6	619.2	5.1
f-50-1_T2900	1	2900	1892	0.9990	1307	822.6	2.9
f-50-100_T1500	100	1500	175	0.0000	30.7	24.4	2439
f-50-100_T1700	100	1700	663	0.0000	35.5	27.2	2721
f-50-100_T1900	100	1900	1458	-8.0616	40.0	31.7	2463
f-50-100_T2100	100	2100	1827	0.9404	250.2	203.0	113.9
f-50-100_T2500	100	2500	1891	0.9994	272.2	204.1	9.0
f-50-100_T2700	100	2700	1893	0.9993	588.3	477.0	5.4
f-50-100_T2900	100	2900	1893	0.9995	985.8	770.5	4.7
f-50-300_T1500	300	1500	408	0.0000	42.0	32.8	3277
f-50-300_T1700	300	1700	1215	0.0000	31.4	24.9	2493
f-50-300_T1900	300	1900	1562	-18,428.4	90.9	63.1	6155
f-50-300_T2100	300	2100	1869	0.8770	171.3	126.3	296.2
f-50-300_T2300	300	2300	1889	0.9923	289.7	235.7	31.6
f-50-300_T2500	300	2500	1894	0.9997	207.8	160.4	27.9
f-50-300_T2700	300	2700	1893	0.9991	806.7	473.1	6.5
f-50-300_T2900	300	2900	1895	0.9987	1634	953.9	2.7
f-50-500_T1500	500	1500	434	0.0000	41.6	33.2	3323

continued on next page

Table S12 (continued)

Simulation	$n(O_2)$	T (K)	n_{rows}	R^2	RMSE (molecule·ps)	MAE (molecule·ps)	MAPE (%)
f-50-500_T2100	500	2100	1886	0.9747	108.6	80.7	338.0
f-50-500_T2300	500	2300	1891	0.9942	304.5	240.9	79.9
f-50-500_T2500	500	2500	1892	0.9986	376.0	303.6	10.0
f-50-500_T2700	500	2700	1895	0.9996	493.6	328.3	5.6
f-50-500_T2900	500	2900	1893	0.9996	846.2	619.7	4.4
f-50-700_T1700	700	1700	1154	0.0000	23.9	19.0	1897
f-50-700_T1900	700	1900	1812	0.8059	70.7	59.5	423.1
f-50-700_T2100	700	2100	1874	0.9830	89.6	69.6	365.5
f-50-700_T2500	700	2500	1894	0.9988	396.9	299.1	8.2
f-50-700_T2700	700	2700	1893	0.9991	649.3	442.5	4.9
f-50-700_T2900	700	2900	1894	0.9995	972.1	806.6	7.7
f-50-900_T2100	900	2100	1876	0.8223	362.1	296.7	2827
f-50-900_T2500	900	2500	1884	0.9944	725.2	585.8	27.7
f-50-900_T2900	900	2900	1893	0.9991	1251	982.3	5.7
**Repeat: firstpass Case: A Model: ANN**							
f-50-1_T1700	1	1700	357	0.0000	35.3	26.8	2681
f-50-1_T1900	1	1900	1318	0.0000	107.3	86.8	8675
f-50-1_T2100	1	2100	1816	0.7419	165.6	125.5	462.7
f-50-1_T2500	1	2500	1892	0.9937	694.0	614.5	46.1
f-50-1_T2700	1	2700	1893	0.9992	630.4	494.8	13.8
f-50-1_T2900	1	2900	1892	0.9999	466.8	360.8	4.7
f-50-100_T1500	100	1500	175	0.0000	48.2	37.6	3760
f-50-100_T1700	100	1700	663	0.0000	65.2	57.0	5703
f-50-100_T1900	100	1900	1458	-1.9908	23.0	14.5	1018
f-50-100_T2100	100	2100	1827	0.4018	792.2	642.1	146.8
f-50-100_T2500	100	2500	1891	0.9992	300.0	257.2	12.6
f-50-100_T2700	100	2700	1893	0.9993	595.4	458.9	5.3
f-50-100_T2900	100	2900	1893	0.9997	756.1	557.8	4.8
f-50-300_T1500	300	1500	408	0.0000	47.2	44.0	4404
f-50-300_T1700	300	1700	1215	0.0000	97.1	93.1	9314
f-50-300_T1900	300	1900	1562	-36,499.1	127.9	107.2	10,396
f-50-300_T2100	300	2100	1869	-0.8068	656.7	407.3	489.2
f-50-300_T2300	300	2300	1889	0.9030	1026	751.2	42.5
f-50-300_T2500	300	2500	1894	0.9969	625.6	492.4	31.5
f-50-300_T2700	300	2700	1893	0.9998	390.4	304.1	14.3
f-50-300_T2900	300	2900	1895	0.9999	427.3	336.2	4.8

continued on next page

Table S12 (continued)

Simulation	$n(O_2)$	T (K)	n_{rows}	R^2	RMSE (molecule·ps)	MAE (molecule·ps)	MAPE (%)
f-50-500_T1500	500	1500	434	0.0000	27.5	24.3	2428
f-50-500_T2100	500	2100	1886	0.7934	310.0	239.0	678.7
f-50-500_T2300	500	2300	1891	0.9673	721.5	589.9	37.2
f-50-500_T2500	500	2500	1892	0.9965	603.4	445.1	10.6
f-50-500_T2700	500	2700	1895	0.9994	617.7	509.5	6.8
f-50-500_T2900	500	2900	1893	0.9999	447.1	345.1	4.1
f-50-700_T1700	700	1700	1154	0.0000	90.9	85.2	8525
f-50-700_T1900	700	1900	1812	-0.0548	164.9	155.2	2186
f-50-700_T2100	700	2100	1874	0.7826	320.0	215.2	1227
f-50-700_T2500	700	2500	1894	0.9961	708.9	577.2	11.9
f-50-700_T2700	700	2700	1893	0.9994	533.0	418.7	7.0
f-50-700_T2900	700	2900	1894	0.9997	796.5	683.5	4.0
f-50-900_T2100	900	2100	1876	0.9043	265.7	234.2	8507
f-50-900_T2500	900	2500	1884	0.9947	706.2	544.7	87.6
f-50-900_T2900	900	2900	1893	0.9991	1220	1021	10.3
**Repeat: firstpass Case: A Model: SVR**							
f-50-1_T1700	1	1700	357	0.0000	1220	1082	108,195
f-50-1_T1900	1	1900	1318	0.0000	1140	1075	107,541
f-50-1_T2100	1	2100	1816	-22.1486	1568	1109	9660
f-50-1_T2500	1	2500	1892	0.8634	3233	2649	170.2
f-50-1_T2700	1	2700	1893	0.7110	11,649	6762	39.1
f-50-1_T2900	1	2900	1892	-0.1879	44,089	31,693	79.0
f-50-100_T1500	100	1500	175	0.0000	644.8	435.0	43,502
f-50-100_T1700	100	1700	663	0.0000	572.4	427.0	42,704
f-50-100_T1900	100	1900	1458	-1527.2	519.2	369.3	21,753
f-50-100_T2100	100	2100	1827	0.4897	731.7	637.7	1348
f-50-100_T2500	100	2500	1891	0.9139	3147	2169	133.3
f-50-100_T2700	100	2700	1893	0.9227	6058	3277	31.8
f-50-100_T2900	100	2900	1893	0.1226	40,932	25,948	33.4
f-50-300_T1500	300	1500	408	0.0000	300.3	233.8	23,377
f-50-300_T1700	300	1700	1215	0.0000	499.6	352.3	35,234
f-50-300_T1900	300	1900	1562	-424,660.4	436.3	391.5	38,043
f-50-300_T2100	300	2100	1869	-15.0207	1956	1382	7051
f-50-300_T2300	300	2300	1889	0.6498	1950	1438	254.7
f-50-300_T2500	300	2500	1894	0.9774	1695	1160	675.9
f-50-300_T2700	300	2700	1893	0.5330	18,072	12,764	109.9

continued on next page

Table S12 (continued)

Simulation	$n(O_2)$	T (K)	n_{rows}	R^2	RMSE (molecule·ps)	MAE (molecule·ps)	MAPE (%)
f-50-300_T2900	300	2900	1895	-0.1819	49,733	35,206	74.7
f-50-500_T1500	500	1500	434	0.0000	549.5	351.3	35,130
f-50-500_T2100	500	2100	1886	-4.9756	1667	1155	5279
f-50-500_T2300	500	2300	1891	0.8147	1717	1449	451.7
f-50-500_T2500	500	2500	1892	0.9544	2176	1590	46.6
f-50-500_T2700	500	2700	1895	0.8037	11,160	6907	40.0
f-50-500_T2900	500	2900	1893	0.2354	37,814	25,064	34.6
f-50-700_T1700	700	1700	1154	0.0000	1776	1666	166,609
f-50-700_T1900	700	1900	1812	-28.9570	878.7	643.7	3472
f-50-700_T2100	700	2100	1874	-5.2039	1710	1167	5175
f-50-700_T2500	700	2500	1894	0.9384	2813	1658	55.1
f-50-700_T2700	700	2700	1893	0.6349	13,361	8439	64.6
f-50-700_T2900	700	2900	1894	0.0746	40,975	27,734	48.3
f-50-900_T2100	900	2100	1876	-13.7583	3300	2996	81,614
f-50-900_T2500	900	2500	1884	0.8703	3494	3078	979.9
f-50-900_T2900	900	2900	1893	-0.0258	41,638	27,544	124.5
**Repeat: firstpass Case: A Model: GPR**							
f-50-1_T1700	1	1700	357	0.0000	13.4	10.4	1040
f-50-1_T1900	1	1900	1318	0.0000	7.8	6.7	671.2
f-50-1_T2100	1	2100	1816	0.9659	60.2	51.8	40.6
f-50-1_T2500	1	2500	1892	0.9994	206.1	160.7	15.0
f-50-1_T2700	1	2700	1893	0.9999	183.8	157.8	4.3
f-50-1_T2900	1	2900	1892	0.9999	484.9	387.0	2.1
f-50-100_T1500	100	1500	175	0.0000	14.2	8.8	880.0
f-50-100_T1700	100	1700	663	0.0000	9.2	8.7	872.2
f-50-100_T1900	100	1900	1458	-0.5935	16.8	15.2	1273
f-50-100_T2100	100	2100	1827	0.9931	85.3	77.4	91.9
f-50-100_T2500	100	2500	1891	0.9996	224.0	176.2	7.5
f-50-100_T2700	100	2700	1893	0.9999	174.5	133.4	2.8
f-50-100_T2900	100	2900	1893	1.0000	255.9	228.6	3.7
f-50-300_T1500	300	1500	408	0.0000	10.0	7.1	708.1
f-50-300_T1700	300	1700	1215	0.0000	10.1	7.9	790.7
f-50-300_T1900	300	1900	1562	-794.7	18.9	16.0	1586
f-50-300_T2100	300	2100	1869	0.9619	95.4	75.2	381.3
f-50-300_T2300	300	2300	1889	0.9981	144.3	123.5	29.1
f-50-300_T2500	300	2500	1894	0.9999	124.9	103.4	29.2

continued on next page

Table S12 (continued)

Simulation	$n(O_2)$	T (K)	n_{rows}	R^2	RMSE (molecule·ps)	MAE (molecule·ps)	MAPE (%)
f-50-300_T2700	300	2700	1893	0.9999	246.9	211.1	5.1
f-50-300_T2900	300	2900	1895	0.9999	351.9	269.3	3.0
f-50-500_T1500	500	1500	434	0.0000	31.8	29.4	2943
f-50-500_T2100	500	2100	1886	0.9773	102.7	77.0	296.6
f-50-500_T2300	500	2300	1891	0.9956	263.1	188.1	28.7
f-50-500_T2500	500	2500	1892	0.9999	120.3	97.7	5.8
f-50-500_T2700	500	2700	1895	0.9999	232.7	198.1	6.5
f-50-500_T2900	500	2900	1893	0.9999	375.8	347.0	3.8
f-50-700_T1700	700	1700	1154	0.0000	103.9	90.1	9007
f-50-700_T1900	700	1900	1812	0.8910	53.0	37.0	824.9
f-50-700_T2100	700	2100	1874	0.9714	116.1	89.1	134.7
f-50-700_T2500	700	2500	1894	0.9998	168.8	147.3	7.9
f-50-700_T2700	700	2700	1893	0.9999	259.6	205.0	4.6
f-50-700_T2900	700	2900	1894	0.9999	379.2	314.4	4.5
f-50-900_T2100	900	2100	1876	0.9447	201.9	147.7	2907
f-50-900_T2500	900	2500	1884	0.9951	679.5	565.7	35.7
f-50-900_T2900	900	2900	1893	0.9995	902.3	799.5	4.3
**Repeat: firstpass Case: A Model: persistence**							
f-50-1_T1700	1	1700	357	1.0000	0.0	0.0	0.0
f-50-1_T1900	1	1900	1318	1.0000	0.0	0.0	0.0
f-50-1_T2100	1	2100	1816	0.9631	62.6	54.9	23.7
f-50-1_T2500	1	2500	1892	0.9646	1647	1468	28.5
f-50-1_T2700	1	2700	1893	0.9644	4090	3642	27.3
f-50-1_T2900	1	2900	1892	0.9666	7390	6812	25.5
f-50-100_T1500	100	1500	175	1.0000	0.0	0.0	0.0
f-50-100_T1700	100	1700	663	1.0000	0.0	0.0	0.0
f-50-100_T1900	100	1900	1458	0.5926	8.5	3.1	11.9
f-50-100_T2100	100	2100	1827	0.9585	208.7	176.9	28.0
f-50-100_T2500	100	2500	1891	0.9636	2046	1794	29.2
f-50-100_T2700	100	2700	1893	0.9633	4174	3673	26.9
f-50-100_T2900	100	2900	1893	0.9656	8105	7371	25.8
f-50-300_T1500	300	1500	408	1.0000	0.0	0.0	0.0
f-50-300_T1700	300	1700	1215	1.0000	0.0	0.0	0.0
f-50-300_T1900	300	1900	1562	0.0667	0.6	0.1	3.4
f-50-300_T2100	300	2100	1869	0.9635	93.4	80.0	24.3
f-50-300_T2300	300	2300	1889	0.9586	670.4	572.4	28.8

continued on next page

Table S12 (continued)

Simulation	$n(O_2)$	T (K)	n_{rows}	R^2	RMSE (molecule·ps)	MAE (molecule·ps)	MAPE (%)
f-50-300_T2500	300	2500	1894	0.9609	2228	1892	31.4
f-50-300_T2700	300	2700	1893	0.9631	5080	4441	28.5
f-50-300_T2900	300	2900	1895	0.9655	8494	7665	26.3
f-50-500_T1500	500	1500	434	1.0000	0.0	0.0	0.0
f-50-500_T2100	500	2100	1886	0.9467	157.5	113.8	27.3
f-50-500_T2300	500	2300	1891	0.9589	808.1	663.3	31.3
f-50-500_T2500	500	2500	1892	0.9581	2085	1751	28.4
f-50-500_T2700	500	2700	1895	0.9622	4897	4216	29.2
f-50-500_T2900	500	2900	1893	0.9655	8037	7244	27.0
f-50-700_T1700	700	1700	1154	1.0000	0.0	0.0	0.0
f-50-700_T1900	700	1900	1812	0.9550	34.0	27.5	21.3
f-50-700_T2100	700	2100	1874	0.9562	143.7	115.1	27.1
f-50-700_T2500	700	2500	1894	0.9619	2213	1916	28.9
f-50-700_T2700	700	2700	1893	0.9621	4303	3713	28.5
f-50-700_T2900	700	2900	1894	0.9644	8040	7131	27.6
f-50-900_T2100	900	2100	1876	0.9632	164.8	133.9	23.0
f-50-900_T2500	900	2500	1884	0.9600	1940	1651	31.2
f-50-900_T2900	900	2900	1893	0.9640	7798	6850	28.8
**Repeat: firstpass Case: A Model: physical_MLR**							
f-50-1_T1700	1	1700	357	0.0000	19,534	15,721	1,572,079
f-50-1_T1900	1	1900	1318	0.0000	15,053	12,848	1,284,761
f-50-1_T2100	1	2100	1816	-1732.2	13,568	11,554	140,773
f-50-1_T2500	1	2500	1892	-1.6586	14,263	12,681	459.2
f-50-1_T2700	1	2700	1893	0.7733	10,315	9389	298.6
f-50-1_T2900	1	2900	1892	0.4257	30,657	23,598	175.4
f-50-100_T1500	100	1500	175	0.0000	29,286	25,089	2,508,930
f-50-100_T1700	100	1700	663	0.0000	17,953	14,504	1,450,368
f-50-100_T1900	100	1900	1458	-1.22×10^6	14,683	12,373	981,577
f-50-100_T2100	100	2100	1827	-209.8	14,874	12,786	33,260
f-50-100_T2500	100	2500	1891	0.2265	9430	8674	310.3
f-50-100_T2700	100	2700	1893	0.7773	10,281	9407	238.8
f-50-100_T2900	100	2900	1893	0.4622	32,047	24,344	173.7
f-50-300_T1500	300	1500	408	0.0000	26,561	22,680	2,268,010
f-50-300_T1700	300	1700	1215	0.0000	19,011	14,993	1,499,316
f-50-300_T1900	300	1900	1562	-4.99×10^8	14,954	12,517	1,224,385
f-50-300_T2100	300	2100	1869	-947.9	15,050	12,922	281,782

continued on next page

Table S12 (continued)

Simulation	$n(O_2)$	T (K)	n_{rows}	R^2	RMSE (molecule·ps)	MAE (molecule·ps)	MAPE (%)
f-50-300_T2300	300	2300	1889	-20.3703	15,234	12,826	4338
f-50-300_T2500	300	2500	1894	-0.0588	11,597	10,544	2114
f-50-300_T2700	300	2700	1893	0.7334	13,654	11,204	302.2
f-50-300_T2900	300	2900	1895	0.3316	37,400	28,168	186.6
f-50-500_T1500	500	1500	434	0.0000	26,460	22,328	2,232,805
f-50-500_T2100	500	2100	1886	-489.5	15,105	13,041	302,682
f-50-500_T2300	500	2300	1891	-13.0690	14,959	12,738	7584
f-50-500_T2500	500	2500	1892	-0.6015	12,892	11,541	340.5
f-50-500_T2700	500	2700	1895	0.7923	11,479	10,169	354.9
f-50-500_T2900	500	2900	1893	0.4968	30,678	23,643	237.5
f-50-700_T1700	700	1700	1154	0.0000	17,465	13,412	1,341,152
f-50-700_T1900	700	1900	1812	-7503.4	13,907	11,733	516,385
f-50-700_T2100	700	2100	1874	-471.1	14,915	12,907	257,677
f-50-700_T2500	700	2500	1894	0.1000	10,751	9731	392.7
f-50-700_T2700	700	2700	1893	0.7532	10,986	10,090	384.2
f-50-700_T2900	700	2900	1894	0.5019	30,063	23,639	314.3
f-50-900_T2100	900	2100	1876	-256.4	13,781	11,906	413,908
f-50-900_T2500	900	2500	1884	-0.6323	12,395	11,206	794.7
f-50-900_T2900	900	2900	1893	0.5451	27,729	21,575	396.3
**Repeat: firstpass Case: A Model: physical_RF**							
f-50-1_T1700	1	1700	357	0.0000	0.0	0.0	0.4
f-50-1_T1900	1	1900	1318	0.0000	13.6	4.7	471.9
f-50-1_T2100	1	2100	1816	-8.8284	1022	747.2	386.4
f-50-1_T2500	1	2500	1892	0.8899	2903	1973	14.6
f-50-1_T2700	1	2700	1893	0.9945	1605	636.5	5.0
f-50-1_T2900	1	2900	1892	0.9885	4346	2537	3.7
f-50-100_T1500	100	1500	175	0.0000	0.1	0.0	1.6
f-50-100_T1700	100	1700	663	0.0000	1.1	0.3	34.3
f-50-100_T1900	100	1900	1458	-0.1169	14.0	4.9	21.4
f-50-100_T2100	100	2100	1827	0.0009	1024	743.7	69.5
f-50-100_T2500	100	2500	1891	0.9594	2160	1551	11.2
f-50-100_T2700	100	2700	1893	0.9959	1399	695.5	6.5
f-50-100_T2900	100	2900	1893	0.9902	4332	2712	4.9
f-50-300_T1500	300	1500	408	0.0000	0.0	0.0	0.2
f-50-300_T1700	300	1700	1215	0.0000	0.1	0.1	5.5
f-50-300_T1900	300	1900	1562	-412.9	13.6	4.8	415.5

continued on next page

Table S12 (continued)

Simulation	$n(O_2)$	T (K)	n_{rows}	R^2	RMSE (molecule·ps)	MAE (molecule·ps)	MAPE (%)
f-50-300_T2100	300	2100	1869	-1.5920	786.6	573.9	493.6
f-50-300_T2300	300	2300	1889	0.9437	781.7	620.2	25.3
f-50-300_T2500	300	2500	1894	0.9933	920.6	564.4	37.8
f-50-300_T2700	300	2700	1893	0.9345	6771	4918	16.3
f-50-300_T2900	300	2900	1895	0.9957	2983	2167	5.5
f-50-500_T1500	500	1500	434	0.0000	0.1	0.0	1.2
f-50-500_T2100	500	2100	1886	0.8755	240.7	169.1	64.7
f-50-500_T2300	500	2300	1891	0.9607	790.8	627.0	45.6
f-50-500_T2500	500	2500	1892	0.9764	1564	1233	16.9
f-50-500_T2700	500	2700	1895	0.9867	2908	2354	13.3
f-50-500_T2900	500	2900	1893	0.9926	3718	3050	11.5
f-50-700_T1700	700	1700	1154	0.0000	276.5	224.2	22,422
f-50-700_T1900	700	1900	1812	-1.1429	235.0	171.8	81.0
f-50-700_T2100	700	2100	1874	0.8547	261.7	194.1	43.2
f-50-700_T2500	700	2500	1894	0.9533	2449	1965	21.5
f-50-700_T2700	700	2700	1893	0.9574	4562	3366	11.3
f-50-700_T2900	700	2900	1894	0.9954	2877	2492	9.9
f-50-900_T2100	900	2100	1876	0.9050	264.7	201.0	498.7
f-50-900_T2500	900	2500	1884	0.9175	2787	2263	45.1
f-50-900_T2900	900	2900	1893	0.9918	3731	3181	17.9
**Repeat: firstpass Case: B Model: MLR**							
f-50-1_T1700	1	1700	357	0.0000	24,365	20,208	2,020,836
f-50-1_T1900	1	1900	1318	0.0000	21,168	19,323	1,932,263
f-50-1_T2100	1	2100	1816	-3262.1	18,616	15,725	182,604
f-50-1_T2500	1	2500	1892	0.9495	1967	1474	647.8
f-50-1_T2700	1	2700	1893	0.5600	14,373	12,877	331.9
f-50-1_T2900	1	2900	1892	0.3069	33,678	25,665	125.9
f-50-100_T1500	100	1500	175	0.0000	15,739	13,514	1,351,368
f-50-100_T1700	100	1700	663	0.0000	16,198	14,295	1,429,456
f-50-100_T1900	100	1900	1458	-2.08×10^6	19,160	17,318	1,317,038
f-50-100_T2100	100	2100	1827	-3.3967	2148	1733	4400
f-50-100_T2500	100	2500	1891	0.8603	4008	3469	357.3
f-50-100_T2700	100	2700	1893	0.9159	6316	5347	164.0
f-50-100_T2900	100	2900	1893	0.4477	32,475	22,236	63.1
f-50-300_T1500	300	1500	408	0.0000	7206	6229	622,853
f-50-300_T1700	300	1700	1215	0.0000	8568	7346	734,640

continued on next page

Table S12 (continued)

Simulation	$n(O_2)$	T (K)	n_{rows}	R^2	RMSE (molecule·ps)	MAE (molecule·ps)	MAPE (%)
f-50-300_T1900	300	1900	1562	-7.14×10^7	5658	5020	489,549
f-50-300_T2100	300	2100	1869	-1787.6	20,662	16,743	60,450
f-50-300_T2300	300	2300	1889	-34.3163	19,583	16,579	2318
f-50-300_T2500	300	2500	1894	-0.7589	14,948	13,851	4383
f-50-300_T2700	300	2700	1893	0.6953	14,598	12,337	727.4
f-50-300_T2900	300	2900	1895	0.5086	32,068	24,638	253.5
f-50-500_T1500	500	1500	434	0.0000	7648	6583	658,339
f-50-500_T2100	500	2100	1886	-1070.5	22,325	18,925	202,879
f-50-500_T2300	500	2300	1891	-14.6073	15,755	13,789	4194
f-50-500_T2500	500	2500	1892	-1.1584	14,967	13,928	797.9
f-50-500_T2700	500	2700	1895	0.6103	15,724	13,834	734.5
f-50-500_T2900	500	2900	1893	0.0968	41,100	30,065	233.3
f-50-700_T1700	700	1700	1154	0.0000	4312	3576	357,617
f-50-700_T1900	700	1900	1812	-9672.6	15,789	14,237	95,640
f-50-700_T2100	700	2100	1874	-897.8	20,579	18,475	265,420
f-50-700_T2500	700	2500	1894	-0.3382	13,109	12,334	1163
f-50-700_T2700	700	2700	1893	0.5344	15,090	13,246	647.9
f-50-700_T2900	700	2900	1894	0.1885	38,371	29,187	301.7
f-50-900_T2100	900	2100	1876	-310.2	15,155	12,782	312,141
f-50-900_T2500	900	2500	1884	-2.2823	17,576	16,425	4095
f-50-900_T2900	900	2900	1893	-1.4172	63,918	49,263	766.3
**Repeat: firstpass Case: B Model: DT**							
f-50-1_T1700	1	1700	357	0.0000	5.8	0.7	69.8
f-50-1_T1900	1	1900	1318	1.0000	0.0	0.0	0.0
f-50-1_T2100	1	2100	1816	-6.9396	918.3	642.7	369.9
f-50-1_T2500	1	2500	1892	-1.9474	15,018	10,415	98.2
f-50-1_T2700	1	2700	1893	0.0983	20,575	13,046	40.7
f-50-1_T2900	1	2900	1892	0.4819	29,117	21,398	37.5
f-50-100_T1500	100	1500	175	0.0000	0.0	0.0	0.2
f-50-100_T1700	100	1700	663	1.0000	0.0	0.0	0.0
f-50-100_T1900	100	1900	1458	-16.2050	55.1	37.7	3301
f-50-100_T2100	100	2100	1827	-129.8	11,717	5576	261.1
f-50-100_T2500	100	2500	1891	-1.2958	16,246	11,151	79.0
f-50-100_T2700	100	2700	1893	0.4801	15,708	10,651	36.7
f-50-100_T2900	100	2900	1893	0.8920	14,360	7200	17.5
f-50-300_T1500	300	1500	408	1.0000	0.0	0.0	0.0

continued on next page

Table S12 (continued)

Simulation	$n(O_2)$	T (K)	n_{rows}	R^2	RMSE (molecule·ps)	MAE (molecule·ps)	MAPE (%)
f-50-300_T1700	300	1700	1215	0.0000	16.2	9.4	939.3
f-50-300_T1900	300	1900	1562	-21,148.2	97.4	56.7	5430
f-50-300_T2100	300	2100	1869	-0.0045	489.7	373.2	119.4
f-50-300_T2300	300	2300	1889	-6.8343	9224	6332	130.9
f-50-300_T2500	300	2500	1894	-0.3167	12,933	7485	96.0
f-50-300_T2700	300	2700	1893	-0.2246	29,265	22,990	72.0
f-50-300_T2900	300	2900	1895	0.9795	6552	5120	26.7
f-50-500_T1500	500	1500	434	1.0000	0.0	0.0	0.0
f-50-500_T2100	500	2100	1886	-33.6872	4017	2336	260.0
f-50-500_T2300	500	2300	1891	-58.1594	30,675	22,345	470.1
f-50-500_T2500	500	2500	1892	-3.4557	21,505	15,851	114.2
f-50-500_T2700	500	2700	1895	0.2707	21,509	14,366	42.9
f-50-500_T2900	500	2900	1893	0.5120	30,211	21,479	40.8
f-50-700_T1700	700	1700	1154	0.0000	14.5	6.3	626.4
f-50-700_T1900	700	1900	1812	-353.3	3022	1237	1818
f-50-700_T2100	700	2100	1874	-9.0792	2179	1059	139.1
f-50-700_T2500	700	2500	1894	-1.2656	17,057	10,498	59.2
f-50-700_T2700	700	2700	1893	0.3676	17,586	10,316	34.8
f-50-700_T2900	700	2900	1894	-0.3395	49,299	35,951	60.3
f-50-900_T2100	900	2100	1876	-92.9029	8324	3036	622.4
f-50-900_T2500	900	2500	1884	-23.6167	48,134	33,858	214.2
f-50-900_T2900	900	2900	1893	-0.2997	46,868	32,278	50.1
**Repeat: firstpass Case: B Model: RF**							
f-50-1_T1700	1	1700	357	0.0000	499.3	166.5	16,650
f-50-1_T1900	1	1900	1318	0.0000	1.5	0.7	68.7
f-50-1_T2100	1	2100	1816	-402.0	6542	5399	1609
f-50-1_T2500	1	2500	1892	0.5912	5593	4538	60.8
f-50-1_T2700	1	2700	1893	0.8247	9073	5693	21.0
f-50-1_T2900	1	2900	1892	0.8761	14,241	10,397	22.2
f-50-100_T1500	100	1500	175	0.0000	3.3	1.1	110.9
f-50-100_T1700	100	1700	663	0.0000	0.0	0.0	0.7
f-50-100_T1900	100	1900	1458	-21,139.2	1931	1286	79,756
f-50-100_T2100	100	2100	1827	-19.0643	4588	2916	191.2
f-50-100_T2500	100	2500	1891	0.7130	5745	4608	51.7
f-50-100_T2700	100	2700	1893	0.6257	13,329	8513	29.2
f-50-100_T2900	100	2900	1893	0.7176	23,222	16,029	25.0

continued on next page

Table S12 (continued)

Simulation	$n(O_2)$	T (K)	n_{rows}	R^2	RMSE (molecule·ps)	MAE (molecule·ps)	MAPE (%)
f-50-300_T1500	300	1500	408	0.0000	783.5	563.4	56,341
f-50-300_T1700	300	1700	1215	0.0000	43.2	3.4	340.7
f-50-300_T1900	300	1900	1562	-543,211.3	493.4	245.1	22,198
f-50-300_T2100	300	2100	1869	-1583.6	19,448	12,079	1353
f-50-300_T2300	300	2300	1889	-6.6450	9111	6511	127.5
f-50-300_T2500	300	2500	1894	-0.1440	12,055	7157	98.9
f-50-300_T2700	300	2700	1893	0.8745	9369	7567	33.5
f-50-300_T2900	300	2900	1895	0.3915	35,684	29,008	57.0
f-50-500_T1500	500	1500	434	0.0000	6730	4882	488,201
f-50-500_T2100	500	2100	1886	-29.8957	3791	2219	258.0
f-50-500_T2300	500	2300	1891	-30.5234	22,392	16,034	355.9
f-50-500_T2500	500	2500	1892	-1.5996	16,426	11,452	76.5
f-50-500_T2700	500	2700	1895	0.9692	4418	3035	20.4
f-50-500_T2900	500	2900	1893	0.4874	30,962	22,998	41.9
f-50-700_T1700	700	1700	1154	0.0000	10,845	8178	817,752
f-50-700_T1900	700	1900	1812	-1259.0	5698	4687	6471
f-50-700_T2100	700	2100	1874	-7.4765	1999	978.8	137.1
f-50-700_T2500	700	2500	1894	-0.2802	12,822	8541	53.9
f-50-700_T2700	700	2700	1893	0.9763	3403	2326	11.7
f-50-700_T2900	700	2900	1894	0.4712	30,976	23,363	42.0
f-50-900_T2100	900	2100	1876	-366.4	16,466	10,025	2091
f-50-900_T2500	900	2500	1884	-3.6940	21,019	16,216	187.2
f-50-900_T2900	900	2900	1893	-0.3023	46,915	32,083	47.4
**Repeat: firstpass Case: B Model: XGBoost**							
f-50-1_T1700	1	1700	357	0.0000	1194	771.8	77,180
f-50-1_T1900	1	1900	1318	0.0000	458.2	293.1	29,311
f-50-1_T2100	1	2100	1816	-92.1510	3145	2549	3144
f-50-1_T2500	1	2500	1892	0.3490	7058	5369	95.9
f-50-1_T2700	1	2700	1893	0.9559	4552	2950	32.9
f-50-1_T2900	1	2900	1892	0.8444	15,959	10,831	24.3
f-50-100_T1500	100	1500	175	0.0000	1309	1080	108,010
f-50-100_T1700	100	1700	663	0.0000	189.1	131.9	13,191
f-50-100_T1900	100	1900	1458	-151,314.1	5166	3081	148,093
f-50-100_T2100	100	2100	1827	-33.3573	6004	4187	4105
f-50-100_T2500	100	2500	1891	0.2773	9115	5986	122.0
f-50-100_T2700	100	2700	1893	0.7994	9756	6472	22.8

continued on next page

Table S12 (continued)

Simulation	$n(O_2)$	T (K)	n_{rows}	R^2	RMSE (molecule·ps)	MAE (molecule·ps)	MAPE (%)
f-50-100_T2900	100	2900	1893	0.8072	19,189	14,960	36.3
f-50-300_T1500	300	1500	408	0.0000	2504	2504	250,379
f-50-300_T1700	300	1700	1215	0.0000	770.0	508.4	50,839
f-50-300_T1900	300	1900	1562	-1.36×10^7	2467	2467	243,565
f-50-300_T2100	300	2100	1869	-860.8	14,343	8761	3074
f-50-300_T2300	300	2300	1889	-5.1479	8171	4950	171.4
f-50-300_T2500	300	2500	1894	0.8453	4433	2700	70.7
f-50-300_T2700	300	2700	1893	0.8669	9649	8012	102.2
f-50-300_T2900	300	2900	1895	0.8142	19,720	17,181	67.3
f-50-500_T1500	500	1500	434	0.0000	2557	2382	238,238
f-50-500_T2100	500	2100	1886	-41.0644	4423	2309	1157
f-50-500_T2300	500	2300	1891	-34.1256	23,636	18,919	2025
f-50-500_T2500	500	2500	1892	-1.2851	15,400	12,605	279.5
f-50-500_T2700	500	2700	1895	0.9086	7616	5594	40.2
f-50-500_T2900	500	2900	1893	0.1705	39,387	27,651	64.6
f-50-700_T1700	700	1700	1154	0.0000	6693	4891	489,145
f-50-700_T1900	700	1900	1812	-603.7	3948	2586	32,405
f-50-700_T2100	700	2100	1874	-15.1889	2762	1512	811.5
f-50-700_T2500	700	2500	1894	-0.2615	12,728	9369	315.8
f-50-700_T2700	700	2700	1893	0.8967	7106	5202	30.1
f-50-700_T2900	700	2900	1894	0.5213	29,471	22,970	44.9
f-50-900_T2100	900	2100	1876	-419.3	17,612	10,006	9362
f-50-900_T2500	900	2500	1884	-7.7812	28,748	20,947	384.0
f-50-900_T2900	900	2900	1893	0.5650	27,114	16,633	25.1
**Repeat: firstpass Case: B Model: CatBoost**							
f-50-1_T1700	1	1700	357	0.0000	560.3	448.0	44,798
f-50-1_T1900	1	1900	1318	0.0000	379.7	343.2	34,321
f-50-1_T2100	1	2100	1816	-38.8340	2057	854.7	918.7
f-50-1_T2500	1	2500	1892	0.7336	4515	2845	55.2
f-50-1_T2700	1	2700	1893	0.9189	6171	4407	25.6
f-50-1_T2900	1	2900	1892	0.9272	10,917	9241	25.6
f-50-100_T1500	100	1500	175	0.0000	973.0	486.7	48,671
f-50-100_T1700	100	1700	663	0.0000	1308	578.8	57,882
f-50-100_T1900	100	1900	1458	-3813.8	820.3	529.5	28,806
f-50-100_T2100	100	2100	1827	-45.4795	6983	4304	271.0
f-50-100_T2500	100	2500	1891	0.4703	7804	5272	61.6

continued on next page

Table S12 (continued)

Simulation	$n(O_2)$	T (K)	n_{rows}	R^2	RMSE (molecule·ps)	MAE (molecule·ps)	MAPE (%)
f-50-100_T2700	100	2700	1893	0.6292	13,265	9326	42.6
f-50-100_T2900	100	2900	1893	0.8483	17,020	13,493	33.0
f-50-300_T1500	300	1500	408	0.0000	381.2	249.2	24,918
f-50-300_T1700	300	1700	1215	0.0000	305.2	195.9	19,588
f-50-300_T1900	300	1900	1562	-766,133.0	586.0	319.3	28,870
f-50-300_T2100	300	2100	1869	-1695.9	20,125	13,460	3572
f-50-300_T2300	300	2300	1889	-7.2631	9473	6191	152.8
f-50-300_T2500	300	2500	1894	0.7356	5796	3773	61.0
f-50-300_T2700	300	2700	1893	0.9570	5483	4410	77.7
f-50-300_T2900	300	2900	1895	0.6646	26,494	21,800	57.9
f-50-500_T1500	500	1500	434	0.0000	3279	2228	222,824
f-50-500_T2100	500	2100	1886	-24.7389	3460	2313	1557
f-50-500_T2300	500	2300	1891	-26.7583	21,012	13,523	283.1
f-50-500_T2500	500	2500	1892	-2.0246	17,718	11,223	77.7
f-50-500_T2700	500	2700	1895	0.9826	3322	2358	27.9
f-50-500_T2900	500	2900	1893	0.6018	27,289	18,493	34.8
f-50-700_T1700	700	1700	1154	0.0000	17,198	12,379	1,237,936
f-50-700_T1900	700	1900	1812	-179.0	2154	1708	18,031
f-50-700_T2100	700	2100	1874	-29.3628	3783	2613	1764
f-50-700_T2500	700	2500	1894	-0.4699	13,739	9353	72.9
f-50-700_T2700	700	2700	1893	0.9048	6823	4691	27.5
f-50-700_T2900	700	2900	1894	0.5812	27,566	21,193	44.4
f-50-900_T2100	900	2100	1876	-695.2	22,665	12,745	3901
f-50-900_T2500	900	2500	1884	-11.6138	34,455	25,653	207.7
f-50-900_T2900	900	2900	1893	0.4669	30,016	19,002	30.1
**Repeat: firstpass Case: B Model: ANN**							
f-50-1_T1700	1	1700	357	0.0000	7914	7444	744,389
f-50-1_T1900	1	1900	1318	0.0000	1271	667.6	66,760
f-50-1_T2100	1	2100	1816	-59.5440	2536	2054	19,789
f-50-1_T2500	1	2500	1892	0.9644	1651	1243	100.3
f-50-1_T2700	1	2700	1893	0.9681	3868	2668	26.2
f-50-1_T2900	1	2900	1892	0.9917	3689	2982	56.7
f-50-100_T1500	100	1500	175	0.0000	558.5	367.4	36,736
f-50-100_T1700	100	1700	663	0.0000	602.7	357.0	35,697
f-50-100_T1900	100	1900	1458	-1986.5	592.1	412.4	23,573
f-50-100_T2100	100	2100	1827	-3.5322	2181	1514	134.5

continued on next page

Table S12 (continued)

Simulation	$n(O_2)$	T (K)	n_{rows}	R^2	RMSE (molecule·ps)	MAE (molecule·ps)	MAPE (%)
f-50-100_T2500	100	2500	1891	0.9487	2429	2044	200.1
f-50-100_T2700	100	2700	1893	0.8667	7953	5056	18.7
f-50-100_T2900	100	2900	1893	0.8327	17,871	11,223	18.3
f-50-300_T1500	300	1500	408	0.0000	392.5	324.6	32,462
f-50-300_T1700	300	1700	1215	0.0000	707.9	195.1	19,513
f-50-300_T1900	300	1900	1562	-1.26×10^6	751.3	451.9	42,623
f-50-300_T2100	300	2100	1869	-54.6493	3645	1708	1140
f-50-300_T2300	300	2300	1889	-4.2430	7546	4864	109.8
f-50-300_T2500	300	2500	1894	0.9106	3370	2097	78.5
f-50-300_T2700	300	2700	1893	0.9192	7516	6587	278.9
f-50-300_T2900	300	2900	1895	0.9639	8690	7139	129.1
f-50-500_T1500	500	1500	434	0.0000	201.7	134.0	13,401
f-50-500_T2100	500	2100	1886	-2.5382	1283	704.1	542.3
f-50-500_T2300	500	2300	1891	-1.5401	6356	3923	202.9
f-50-500_T2500	500	2500	1892	-0.8475	13,847	8822	68.3
f-50-500_T2700	500	2700	1895	0.9796	3598	2610	15.4
f-50-500_T2900	500	2900	1893	0.8806	14,941	8847	17.3
f-50-700_T1700	700	1700	1154	0.0000	888.2	321.5	32,149
f-50-700_T1900	700	1900	1812	-762.8	4437	3744	31,320
f-50-700_T2100	700	2100	1874	0.7033	373.9	240.9	888.7
f-50-700_T2500	700	2500	1894	0.1796	10,265	7093	74.9
f-50-700_T2700	700	2700	1893	0.8686	8016	5696	27.6
f-50-700_T2900	700	2900	1894	0.7072	23,047	18,040	42.3
f-50-900_T2100	900	2100	1876	-469.6	18,635	7830	4918
f-50-900_T2500	900	2500	1884	-19.7985	44,244	29,266	193.8
f-50-900_T2900	900	2900	1893	0.9830	5366	3359	7.8
**Repeat: firstpass Case: B Model: SVR**							
f-50-1_T1700	1	1700	357	0.0000	997.1	929.9	92,991
f-50-1_T1900	1	1900	1318	0.0000	549.3	537.6	53,758
f-50-1_T2100	1	2100	1816	-20.8392	1523	1065	9161
f-50-1_T2500	1	2500	1892	0.8674	3186	2754	124.9
f-50-1_T2700	1	2700	1893	0.4808	15,612	9130	35.2
f-50-1_T2900	1	2900	1892	-0.3495	46,992	34,097	58.4
f-50-100_T1500	100	1500	175	0.0000	243.9	208.9	20,885
f-50-100_T1700	100	1700	663	0.0000	154.3	116.4	11,641
f-50-100_T1900	100	1900	1458	-1768.3	558.6	329.2	14,183

continued on next page

Table S12 (continued)

Simulation	$n(O_2)$	T (K)	n_{rows}	R^2	RMSE (molecule·ps)	MAE (molecule·ps)	MAPE (%)
f-50-100_T2100	100	2100	1827	0.1071	967.9	814.5	761.2
f-50-100_T2500	100	2500	1891	0.8349	4356	2637	84.8
f-50-100_T2700	100	2700	1893	0.3923	16,983	10,562	32.4
f-50-100_T2900	100	2900	1893	-0.3268	50,333	35,299	46.6
f-50-300_T1500	300	1500	408	0.0000	312.3	274.6	27,457
f-50-300_T1700	300	1700	1215	0.0000	249.0	164.7	16,469
f-50-300_T1900	300	1900	1562	-2.10×10^6	970.5	834.6	82,929
f-50-300_T2100	300	2100	1869	-332.6	8923	6759	3840
f-50-300_T2300	300	2300	1889	-4.8794	7990	6593	237.0
f-50-300_T2500	300	2500	1894	0.7539	5592	3773	167.9
f-50-300_T2700	300	2700	1893	-0.2999	30,152	21,739	95.9
f-50-300_T2900	300	2900	1895	-0.7224	60,037	45,244	77.6
f-50-500_T1500	500	1500	434	0.0000	218.8	144.8	14,480
f-50-500_T2100	500	2100	1886	-105.7	7045	5007	5786
f-50-500_T2300	500	2300	1891	-0.0960	4175	3360	256.0
f-50-500_T2500	500	2500	1892	0.8068	4478	3187	43.1
f-50-500_T2700	500	2700	1895	0.1308	23,481	15,086	46.6
f-50-500_T2900	500	2900	1893	-0.6405	55,391	39,246	53.5
f-50-700_T1700	700	1700	1154	0.0000	2042	1877	187,676
f-50-700_T1900	700	1900	1812	-584.1	3883	3545	26,510
f-50-700_T2100	700	2100	1874	-25.3496	3524	2284	4089
f-50-700_T2500	700	2500	1894	0.7413	5764	3851	54.6
f-50-700_T2700	700	2700	1893	0.0452	21,608	13,934	68.6
f-50-700_T2900	700	2900	1894	-0.5996	53,872	38,581	63.6
f-50-900_T2100	900	2100	1876	-59.7400	6695	4606	42,804
f-50-900_T2500	900	2500	1884	0.6524	5720	4684	611.0
f-50-900_T2900	900	2900	1893	-0.3660	48,049	32,921	75.5
**Repeat: firstpass Case: B Model: GPR**							
f-50-1_T1700	1	1700	357	0.0000	725.8	558.9	55,891
f-50-1_T1900	1	1900	1318	0.0000	395.5	257.1	25,714
f-50-1_T2100	1	2100	1816	-31.6050	1861	1434	631.3
f-50-1_T2500	1	2500	1892	-0.1075	9206	6781	67.2
f-50-1_T2700	1	2700	1893	0.9246	5950	3694	13.8
f-50-1_T2900	1	2900	1892	0.9722	6748	5299	12.3
f-50-100_T1500	100	1500	175	0.0000	234.9	164.6	16,458
f-50-100_T1700	100	1700	663	0.0000	593.8	384.0	38,402

continued on next page

Table S12 (continued)

Simulation	$n(O_2)$	T (K)	n_{rows}	R^2	RMSE (molecule·ps)	MAE (molecule·ps)	MAPE (%)
f-50-100_T1900	100	1900	1458	-24,445.8	2076	1167	49,531
f-50-100_T2100	100	2100	1827	-1.0821	1478	1183	267.6
f-50-100_T2500	100	2500	1891	0.9904	1052	804.7	27.4
f-50-100_T2700	100	2700	1893	0.8009	9719	6314	17.1
f-50-100_T2900	100	2900	1893	0.9794	6278	4196	10.4
f-50-300_T1500	300	1500	408	0.0000	557.4	355.3	35,532
f-50-300_T1700	300	1700	1215	0.0000	1595	1054	105,448
f-50-300_T1900	300	1900	1562	-6.75×10^6	1740	1146	108,944
f-50-300_T2100	300	2100	1869	-486.3	10,785	6848	3141
f-50-300_T2300	300	2300	1889	-0.6166	4190	3031	98.4
f-50-300_T2500	300	2500	1894	0.9408	2742	1911	75.5
f-50-300_T2700	300	2700	1893	0.9715	4462	3669	29.2
f-50-300_T2900	300	2900	1895	0.9464	10,594	9372	29.1
f-50-500_T1500	500	1500	434	0.0000	1543	998.0	99,798
f-50-500_T2100	500	2100	1886	-17.6635	2946	2068	1580
f-50-500_T2300	500	2300	1891	-4.0606	8972	5968	134.0
f-50-500_T2500	500	2500	1892	-0.5121	12,528	7675	45.8
f-50-500_T2700	500	2700	1895	0.9732	4121	3112	23.3
f-50-500_T2900	500	2900	1893	0.5171	30,054	21,366	35.5
f-50-700_T1700	700	1700	1154	0.0000	1568	1314	131,385
f-50-700_T1900	700	1900	1812	-1254.1	5687	4214	16,490
f-50-700_T2100	700	2100	1874	-5.0940	1695	1004	723.8
f-50-700_T2500	700	2500	1894	0.4517	8391	6597	58.4
f-50-700_T2700	700	2700	1893	0.4682	16,127	11,475	37.3
f-50-700_T2900	700	2900	1894	-0.1124	44,925	33,061	52.4
f-50-900_T2100	900	2100	1876	-1317.9	31,197	17,923	16,064
f-50-900_T2500	900	2500	1884	-23.1190	47,645	34,484	326.1
f-50-900_T2900	900	2900	1893	0.7615	20,078	15,155	39.8
**Repeat: firstpass Case: B Model: persistence**							
f-50-1_T1700	1	1700	357	1.0000	0.0	0.0	0.0
f-50-1_T1900	1	1900	1318	1.0000	0.0	0.0	0.0
f-50-1_T2100	1	2100	1816	0.9631	62.6	54.9	23.7
f-50-1_T2500	1	2500	1892	0.9646	1647	1468	28.5
f-50-1_T2700	1	2700	1893	0.9644	4090	3642	27.3
f-50-1_T2900	1	2900	1892	0.9666	7390	6812	25.5
f-50-100_T1500	100	1500	175	1.0000	0.0	0.0	0.0

continued on next page

Table S12 (continued)

Simulation	$n(O_2)$	T (K)	n_{rows}	R^2	RMSE (molecule·ps)	MAE (molecule·ps)	MAPE (%)
f-50-100_T1700	100	1700	663	1.0000	0.0	0.0	0.0
f-50-100_T1900	100	1900	1458	0.5926	8.5	3.1	11.9
f-50-100_T2100	100	2100	1827	0.9585	208.7	176.9	28.0
f-50-100_T2500	100	2500	1891	0.9636	2046	1794	29.2
f-50-100_T2700	100	2700	1893	0.9633	4174	3673	26.9
f-50-100_T2900	100	2900	1893	0.9656	8105	7371	25.8
f-50-300_T1500	300	1500	408	1.0000	0.0	0.0	0.0
f-50-300_T1700	300	1700	1215	1.0000	0.0	0.0	0.0
f-50-300_T1900	300	1900	1562	0.0667	0.6	0.1	3.4
f-50-300_T2100	300	2100	1869	0.9635	93.4	80.0	24.3
f-50-300_T2300	300	2300	1889	0.9586	670.4	572.4	28.8
f-50-300_T2500	300	2500	1894	0.9609	2228	1892	31.4
f-50-300_T2700	300	2700	1893	0.9631	5080	4441	28.5
f-50-300_T2900	300	2900	1895	0.9655	8494	7665	26.3
f-50-500_T1500	500	1500	434	1.0000	0.0	0.0	0.0
f-50-500_T2100	500	2100	1886	0.9467	157.5	113.8	27.3
f-50-500_T2300	500	2300	1891	0.9589	808.1	663.3	31.3
f-50-500_T2500	500	2500	1892	0.9581	2085	1751	28.4
f-50-500_T2700	500	2700	1895	0.9622	4897	4216	29.2
f-50-500_T2900	500	2900	1893	0.9655	8037	7244	27.0
f-50-700_T1700	700	1700	1154	1.0000	0.0	0.0	0.0
f-50-700_T1900	700	1900	1812	0.9550	34.0	27.5	21.3
f-50-700_T2100	700	2100	1874	0.9562	143.7	115.1	27.1
f-50-700_T2500	700	2500	1894	0.9619	2213	1916	28.9
f-50-700_T2700	700	2700	1893	0.9621	4303	3713	28.5
f-50-700_T2900	700	2900	1894	0.9644	8040	7131	27.6
f-50-900_T2100	900	2100	1876	0.9632	164.8	133.9	23.0
f-50-900_T2500	900	2500	1884	0.9600	1940	1651	31.2
f-50-900_T2900	900	2900	1893	0.9640	7798	6850	28.8
**Repeat: firstpass Case: B Model: physical_MLR**							
f-50-1_T1700	1	1700	357	0.0000	19,534	15,721	1,572,079
f-50-1_T1900	1	1900	1318	0.0000	15,053	12,848	1,284,761
f-50-1_T2100	1	2100	1816	-1732.2	13,568	11,554	140,773
f-50-1_T2500	1	2500	1892	-1.6586	14,263	12,681	459.2
f-50-1_T2700	1	2700	1893	0.7733	10,315	9389	298.6
f-50-1_T2900	1	2900	1892	0.4257	30,657	23,598	175.4

continued on next page

Table S12 (continued)

Simulation	$n(O_2)$	T (K)	n_{rows}	R^2	RMSE (molecule·ps)	MAE (molecule·ps)	MAPE (%)
f-50-100_T1500	100	1500	175	0.0000	29,286	25,089	2,508,930
f-50-100_T1700	100	1700	663	0.0000	17,953	14,504	1,450,368
f-50-100_T1900	100	1900	1458	-1.22×10^6	14,683	12,373	981,577
f-50-100_T2100	100	2100	1827	-209.8	14,874	12,786	33,260
f-50-100_T2500	100	2500	1891	0.2265	9430	8674	310.3
f-50-100_T2700	100	2700	1893	0.7773	10,281	9407	238.8
f-50-100_T2900	100	2900	1893	0.4622	32,047	24,344	173.7
f-50-300_T1500	300	1500	408	0.0000	26,561	22,680	2,268,010
f-50-300_T1700	300	1700	1215	0.0000	19,011	14,993	1,499,316
f-50-300_T1900	300	1900	1562	-4.99×10^8	14,954	12,517	1,224,385
f-50-300_T2100	300	2100	1869	-947.9	15,050	12,922	281,782
f-50-300_T2300	300	2300	1889	-20.3703	15,234	12,826	4338
f-50-300_T2500	300	2500	1894	-0.0588	11,597	10,544	2114
f-50-300_T2700	300	2700	1893	0.7334	13,654	11,204	302.2
f-50-300_T2900	300	2900	1895	0.3316	37,400	28,168	186.6
f-50-500_T1500	500	1500	434	0.0000	26,460	22,328	2,232,805
f-50-500_T2100	500	2100	1886	-489.5	15,105	13,041	302,682
f-50-500_T2300	500	2300	1891	-13.0690	14,959	12,738	7584
f-50-500_T2500	500	2500	1892	-0.6015	12,892	11,541	340.5
f-50-500_T2700	500	2700	1895	0.7923	11,479	10,169	354.9
f-50-500_T2900	500	2900	1893	0.4968	30,678	23,643	237.5
f-50-700_T1700	700	1700	1154	0.0000	17,465	13,412	1,341,152
f-50-700_T1900	700	1900	1812	-7503.4	13,907	11,733	516,385
f-50-700_T2100	700	2100	1874	-471.1	14,915	12,907	257,677
f-50-700_T2500	700	2500	1894	0.1000	10,751	9731	392.7
f-50-700_T2700	700	2700	1893	0.7532	10,986	10,090	384.2
f-50-700_T2900	700	2900	1894	0.5019	30,063	23,639	314.3
f-50-900_T2100	900	2100	1876	-256.4	13,781	11,906	413,908
f-50-900_T2500	900	2500	1884	-0.6323	12,395	11,206	794.7
f-50-900_T2900	900	2900	1893	0.5451	27,729	21,575	396.3
**Repeat: firstpass Case: B Model: physical_RF**							
f-50-1_T1700	1	1700	357	0.0000	69.8	26.7	2668
f-50-1_T1900	1	1900	1318	0.0000	14.1	4.8	481.9
f-50-1_T2100	1	2100	1816	0.7139	174.3	93.6	89.1
f-50-1_T2500	1	2500	1892	0.5043	6159	5417	335.8
f-50-1_T2700	1	2700	1893	0.7355	11,143	8305	130.5

continued on next page

Table S12 (continued)

Simulation	$n(O_2)$	T (K)	n_{rows}	R^2	RMSE (molecule·ps)	MAE (molecule·ps)	MAPE (%)
f-50-1_T2900	1	2900	1892	0.8022	17,990	14,762	53.3
f-50-100_T1500	100	1500	175	0.0000	120.7	45.8	4584
f-50-100_T1700	100	1700	663	0.0000	0.6	0.2	18.9
f-50-100_T1900	100	1900	1458	-2743.2	695.7	459.4	32,458
f-50-100_T2100	100	2100	1827	-0.5578	1278	946.5	90.9
f-50-100_T2500	100	2500	1891	0.9714	1813	1568	221.0
f-50-100_T2700	100	2700	1893	0.9963	1320	597.3	4.8
f-50-100_T2900	100	2900	1893	0.7931	19,877	15,280	28.7
f-50-300_T1500	300	1500	408	0.0000	61.3	25.8	2578
f-50-300_T1700	300	1700	1215	0.0000	175.4	88.3	8829
f-50-300_T1900	300	1900	1562	-370,864.8	407.7	269.8	26,096
f-50-300_T2100	300	2100	1869	0.8963	157.4	124.4	311.6
f-50-300_T2300	300	2300	1889	0.9040	1021	891.1	188.9
f-50-300_T2500	300	2500	1894	0.9952	783.5	570.5	27.1
f-50-300_T2700	300	2700	1893	0.8096	11,539	8736	40.9
f-50-300_T2900	300	2900	1895	0.8808	15,791	12,060	22.0
f-50-500_T1500	500	1500	434	0.0000	530.8	365.5	36,545
f-50-500_T2100	500	2100	1886	0.8894	226.8	157.0	45.5
f-50-500_T2300	500	2300	1891	0.4757	2888	1952	87.3
f-50-500_T2500	500	2500	1892	0.9630	1961	1470	41.1
f-50-500_T2700	500	2700	1895	0.9617	4931	3175	35.8
f-50-500_T2900	500	2900	1893	0.9552	9154	7108	43.3
f-50-700_T1700	700	1700	1154	0.0000	381.1	283.3	28,331
f-50-700_T1900	700	1900	1812	-6.4001	436.7	274.0	281.2
f-50-700_T2100	700	2100	1874	0.8525	263.7	200.2	38.0
f-50-700_T2500	700	2500	1894	0.9840	1433	1070	101.1
f-50-700_T2700	700	2700	1893	0.9934	1791	1349	58.3
f-50-700_T2900	700	2900	1894	0.9243	11,718	8438	28.1
f-50-900_T2100	900	2100	1876	-1222.1	30,042	23,912	95,386
f-50-900_T2500	900	2500	1884	-5.3416	24,430	19,620	823.8
f-50-900_T2900	900	2900	1893	0.9929	3467	2968	13.9
**Repeat: strat42 Case: A Model: MLR**							
f-50-1_T1700	1	1700	357	0.0000	933.7	757.7	75,772
f-50-1_T1900	1	1900	1318	0.0000	491.6	411.3	41,133
f-50-1_T2100	1	2100	1816	-0.6170	414.4	393.4	4619
f-50-1_T2500	1	2500	1892	0.9949	623.6	575.2	118.6

continued on next page

Table S12 (continued)

Simulation	$n(O_2)$	T (K)	n_{rows}	R^2	RMSE (molecule·ps)	MAE (molecule·ps)	MAPE (%)
f-50-1_T2700	1	2700	1893	0.9963	1317	907.1	10.8
f-50-1_T2900	1	2900	1892	0.9982	1725	1560	14.4
f-50-100_T1500	100	1500	175	0.0000	1329	1128	112,829
f-50-100_T1700	100	1700	663	0.0000	453.9	388.7	38,871
f-50-100_T1900	100	1900	1458	-1466.0	508.7	424.5	33,122
f-50-100_T2100	100	2100	1827	0.9100	307.3	244.4	701.6
f-50-100_T2500	100	2500	1891	0.9980	478.1	382.4	113.5
f-50-100_T2700	100	2700	1893	0.9995	509.4	424.7	25.4
f-50-100_T2900	100	2900	1893	0.9988	1545	1417	12.5
f-50-300_T1500	300	1500	408	0.0000	193.8	160.0	16,004
f-50-300_T1700	300	1700	1215	0.0000	573.5	563.7	56,374
f-50-300_T1900	300	1900	1562	-30,988.9	117.9	96.6	9619
f-50-300_T2100	300	2100	1869	0.5543	326.2	292.5	4889
f-50-300_T2300	300	2300	1889	0.9862	387.7	371.6	240.1
f-50-300_T2500	300	2500	1894	0.9985	431.2	326.0	596.8
f-50-300_T2700	300	2700	1893	0.9995	590.9	532.3	18.7
f-50-300_T2900	300	2900	1895	0.9985	1797	1525	6.9
f-50-500_T1500	500	1500	434	0.0000	1349	1347	134,683
f-50-500_T2100	500	2100	1886	0.7132	365.2	344.2	3790
f-50-500_T2300	500	2300	1891	0.9828	523.2	486.7	255.2
f-50-500_T2500	500	2500	1892	0.9953	698.7	621.9	48.5
f-50-500_T2700	500	2700	1895	0.9995	570.9	454.4	62.5
f-50-500_T2900	500	2900	1893	0.9983	1766	1534	16.2
f-50-700_T1700	700	1700	1154	0.0000	649.6	627.5	62,752
f-50-700_T1900	700	1900	1812	-17.0981	682.9	591.2	24,123
f-50-700_T2100	700	2100	1874	0.8316	281.7	204.4	1469
f-50-700_T2500	700	2500	1894	0.9978	528.9	499.2	80.4
f-50-700_T2700	700	2700	1893	0.9996	465.7	360.7	35.1
f-50-700_T2900	700	2900	1894	0.9995	968.1	856.1	20.4
f-50-900_T2100	900	2100	1876	0.1247	803.7	727.2	31,843
f-50-900_T2500	900	2500	1884	0.9976	476.0	430.0	163.9
f-50-900_T2900	900	2900	1893	0.9983	1681	1471	34.5
**Repeat: strat42 Case: A Model: DT**							
f-50-1_T1700	1	1700	357	0.0000	1.2	1.2	117.1
f-50-1_T1900	1	1900	1318	0.0000	118.2	64.3	6426
f-50-1_T2100	1	2100	1816	0.8422	129.5	92.2	121.8

continued on next page

Table S12 (continued)

Simulation	$n(O_2)$	T (K)	n_{rows}	R^2	RMSE (molecule·ps)	MAE (molecule·ps)	MAPE (%)
f-50-1_T2500	1	2500	1892	0.9981	379.5	289.3	17.5
f-50-1_T2700	1	2700	1893	0.9998	330.1	258.0	5.6
f-50-1_T2900	1	2900	1892	0.9994	970.9	808.2	5.0
f-50-100_T1500	100	1500	175	0.0000	1.2	1.2	117.1
f-50-100_T1700	100	1700	663	0.0000	5.6	3.4	337.8
f-50-100_T1900	100	1900	1458	-0.1202	14.1	5.8	114.5
f-50-100_T2100	100	2100	1827	0.9441	242.1	163.3	54.5
f-50-100_T2500	100	2500	1891	0.9993	289.5	213.8	11.2
f-50-100_T2700	100	2700	1893	0.9997	371.2	287.2	5.5
f-50-100_T2900	100	2900	1893	0.9997	712.3	445.3	4.6
f-50-300_T1500	300	1500	408	0.0000	1.9	1.9	187.1
f-50-300_T1700	300	1700	1215	0.0000	5.4	3.5	346.2
f-50-300_T1900	300	1900	1562	-94.4387	6.5	3.5	351.0
f-50-300_T2100	300	2100	1869	0.9745	78.0	56.8	33.7
f-50-300_T2300	300	2300	1889	0.9946	242.5	203.6	22.6
f-50-300_T2500	300	2500	1894	0.9996	214.8	150.2	29.1
f-50-300_T2700	300	2700	1893	0.9996	497.6	346.8	4.9
f-50-300_T2900	300	2900	1895	0.9997	806.9	452.3	4.2
f-50-500_T1500	500	1500	434	0.0000	3.2	3.2	322.4
f-50-500_T2100	500	2100	1886	0.9874	76.5	53.0	129.9
f-50-500_T2300	500	2300	1891	0.9948	287.9	212.6	20.0
f-50-500_T2500	500	2500	1892	0.9997	188.6	150.5	6.6
f-50-500_T2700	500	2700	1895	0.9996	488.3	333.1	8.5
f-50-500_T2900	500	2900	1893	0.9999	364.0	300.7	3.8
f-50-700_T1700	700	1700	1154	0.0000	1.9	1.9	187.1
f-50-700_T1900	700	1900	1812	0.9075	48.8	36.6	67.2
f-50-700_T2100	700	2100	1874	0.9951	48.2	38.0	85.2
f-50-700_T2500	700	2500	1894	0.9996	215.1	135.2	7.8
f-50-700_T2700	700	2700	1893	0.9995	482.2	369.9	6.4
f-50-700_T2900	700	2900	1894	1.0000	264.3	211.1	3.8
f-50-900_T2100	900	2100	1876	0.9595	172.9	98.0	824.0
f-50-900_T2500	900	2500	1884	0.9994	246.0	155.8	26.3
f-50-900_T2900	900	2900	1893	0.9999	323.5	240.5	5.9
**Repeat: strat42**	**Case: A**	**Model: RF**					
f-50-1_T1700	1	1700	357	0.0000	1.4	1.3	134.0
f-50-1_T1900	1	1900	1318	0.0000	61.8	31.8	3178

continued on next page

Table S12 (continued)

Simulation	$n(O_2)$	T (K)	n_{rows}	R^2	RMSE (molecule·ps)	MAE (molecule·ps)	MAPE (%)
f-50-1_T2100	1	2100	1816	0.9476	74.6	56.2	84.4
f-50-1_T2500	1	2500	1892	0.9983	356.9	274.3	11.6
f-50-1_T2700	1	2700	1893	0.9998	334.4	263.2	3.9
f-50-1_T2900	1	2900	1892	0.9996	837.2	665.3	4.4
f-50-100_T1500	100	1500	175	0.0000	1.3	1.3	132.4
f-50-100_T1700	100	1700	663	0.0000	4.8	2.6	258.6
f-50-100_T1900	100	1900	1458	0.1364	12.3	5.3	125.9
f-50-100_T2100	100	2100	1827	0.9806	142.5	103.2	47.6
f-50-100_T2500	100	2500	1891	0.9998	167.6	116.8	8.2
f-50-100_T2700	100	2700	1893	0.9999	200.2	173.3	4.0
f-50-100_T2900	100	2900	1893	0.9999	485.0	347.0	4.0
f-50-300_T1500	300	1500	408	0.0000	1.7	1.7	171.8
f-50-300_T1700	300	1700	1215	0.0000	4.8	2.9	292.0
f-50-300_T1900	300	1900	1562	-2980.5	36.6	21.7	2073
f-50-300_T2100	300	2100	1869	0.9790	70.9	52.7	33.8
f-50-300_T2300	300	2300	1889	0.9959	209.8	185.4	18.4
f-50-300_T2500	300	2500	1894	0.9998	166.6	139.1	33.7
f-50-300_T2700	300	2700	1893	0.9997	425.0	284.4	4.9
f-50-300_T2900	300	2900	1895	0.9997	818.8	448.2	3.6
f-50-500_T1500	500	1500	434	0.0000	2.8	2.7	272.5
f-50-500_T2100	500	2100	1886	0.9936	54.5	42.0	132.0
f-50-500_T2300	500	2300	1891	0.9954	270.2	219.8	20.4
f-50-500_T2500	500	2500	1892	0.9998	159.3	137.1	5.2
f-50-500_T2700	500	2700	1895	0.9997	418.8	305.4	7.7
f-50-500_T2900	500	2900	1893	1.0000	266.5	239.3	3.0
f-50-700_T1700	700	1700	1154	0.0000	1.7	1.7	171.4
f-50-700_T1900	700	1900	1812	0.9124	47.5	35.5	67.2
f-50-700_T2100	700	2100	1874	0.9973	35.8	27.5	100.2
f-50-700_T2500	700	2500	1894	0.9998	144.4	85.8	5.5
f-50-700_T2700	700	2700	1893	0.9998	343.6	285.4	5.2
f-50-700_T2900	700	2900	1894	1.0000	200.2	155.7	3.2
f-50-900_T2100	900	2100	1876	0.9762	132.6	81.3	699.7
f-50-900_T2500	900	2500	1884	0.9999	106.5	76.3	24.1
f-50-900_T2900	900	2900	1893	1.0000	256.2	207.1	5.1
**Repeat: strat42 Case: A Model: XGBoost**							
f-50-1_T1700	1	1700	357	0.0000	3.3	1.2	121.5

continued on next page

Table S12 (continued)

Simulation	$n(O_2)$	T (K)	n_{rows}	R^2	RMSE (molecule·ps)	MAE (molecule·ps)	MAPE (%)
f-50-1_T1900	1	1900	1318	0.0000	1.6	1.1	107.4
f-50-1_T2100	1	2100	1816	0.8973	104.4	82.6	86.2
f-50-1_T2500	1	2500	1892	0.9987	316.8	272.2	14.4
f-50-1_T2700	1	2700	1893	0.9998	303.2	234.3	3.9
f-50-1_T2900	1	2900	1892	0.9985	1567	801.7	4.1
f-50-100_T1500	100	1500	175	0.0000	2.8	1.0	102.3
f-50-100_T1700	100	1700	663	0.0000	2.9	1.7	171.8
f-50-100_T1900	100	1900	1458	0.7603	6.5	3.9	182.0
f-50-100_T2100	100	2100	1827	0.9952	71.3	50.8	35.0
f-50-100_T2500	100	2500	1891	0.9996	219.0	167.7	7.0
f-50-100_T2700	100	2700	1893	0.9997	406.0	309.1	4.0
f-50-100_T2900	100	2900	1893	0.9991	1311	843.3	4.1
f-50-300_T1500	300	1500	408	0.0000	0.7	0.4	41.2
f-50-300_T1700	300	1700	1215	0.0000	3.5	2.8	282.0
f-50-300_T1900	300	1900	1562	-3740.4	41.0	26.0	2492
f-50-300_T2100	300	2100	1869	0.8718	175.0	120.6	58.8
f-50-300_T2300	300	2300	1889	0.9936	264.0	222.7	21.4
f-50-300_T2500	300	2500	1894	0.9998	145.9	108.5	48.8
f-50-300_T2700	300	2700	1893	0.9997	454.3	350.8	4.0
f-50-300_T2900	300	2900	1895	0.9986	1728	989.7	3.3
f-50-500_T1500	500	1500	434	0.0000	3.1	3.0	297.7
f-50-500_T2100	500	2100	1886	0.9960	42.9	35.2	303.1
f-50-500_T2300	500	2300	1891	0.9981	173.4	127.3	21.7
f-50-500_T2500	500	2500	1892	0.9997	174.4	137.6	4.2
f-50-500_T2700	500	2700	1895	0.9997	416.6	285.6	9.2
f-50-500_T2900	500	2900	1893	0.9996	873.4	512.4	3.0
f-50-700_T1700	700	1700	1154	0.0000	5.7	4.7	469.3
f-50-700_T1900	700	1900	1812	0.9460	37.3	26.2	31.3
f-50-700_T2100	700	2100	1874	0.9970	37.3	26.2	75.0
f-50-700_T2500	700	2500	1894	0.9998	172.9	125.4	3.6
f-50-700_T2700	700	2700	1893	0.9995	507.2	385.1	4.9
f-50-700_T2900	700	2900	1894	0.9998	653.4	411.8	2.8
f-50-900_T2100	900	2100	1876	0.9651	160.4	136.9	564.5
f-50-900_T2500	900	2500	1884	0.9995	205.9	164.1	20.5
f-50-900_T2900	900	2900	1893	0.9992	1134	911.1	6.2
**Repeat: strat42 Case: A Model: CatBoost**							

continued on next page

Table S12 (continued)

Simulation	$n(O_2)$	T (K)	n_{rows}	R^2	RMSE (molecule·ps)	MAE (molecule·ps)	MAPE (%)
f-50-1_T1700	1	1700	357	0.0000	36.3	29.4	2945
f-50-1_T1900	1	1900	1318	0.0000	48.7	41.5	4154
f-50-1_T2100	1	2100	1816	0.7208	172.2	127.6	227.4
f-50-1_T2500	1	2500	1892	0.9981	381.3	292.6	13.0
f-50-1_T2700	1	2700	1893	0.9993	572.7	456.1	5.2
f-50-1_T2900	1	2900	1892	0.9975	2029	1444	6.2
f-50-100_T1500	100	1500	175	0.0000	28.9	23.7	2370
f-50-100_T1700	100	1700	663	0.0000	28.1	21.9	2189
f-50-100_T1900	100	1900	1458	-7.3778	38.4	31.3	2400
f-50-100_T2100	100	2100	1827	0.9369	257.3	207.7	55.8
f-50-100_T2500	100	2500	1891	0.9993	287.5	219.0	8.2
f-50-100_T2700	100	2700	1893	0.9993	595.2	476.0	6.6
f-50-100_T2900	100	2900	1893	0.9989	1459	1159	6.0
f-50-300_T1500	300	1500	408	0.0000	44.9	40.4	4043
f-50-300_T1700	300	1700	1215	0.0000	37.0	30.6	3061
f-50-300_T1900	300	1900	1562	-9441.5	65.1	57.2	5564
f-50-300_T2100	300	2100	1869	0.7949	221.3	135.3	346.8
f-50-300_T2300	300	2300	1889	0.9915	303.1	235.8	20.8
f-50-300_T2500	300	2500	1894	0.9993	298.6	227.9	46.7
f-50-300_T2700	300	2700	1893	0.9997	487.5	368.3	5.4
f-50-300_T2900	300	2900	1895	0.9982	1936	1111	4.8
f-50-500_T1500	500	1500	434	0.0000	144.3	138.9	13,886
f-50-500_T2100	500	2100	1886	0.9493	153.6	119.6	727.7
f-50-500_T2300	500	2300	1891	0.9939	312.5	218.4	20.9
f-50-500_T2500	500	2500	1892	0.9987	372.1	291.5	9.1
f-50-500_T2700	500	2700	1895	0.9996	533.5	372.6	10.9
f-50-500_T2900	500	2900	1893	0.9996	879.3	680.4	4.7
f-50-700_T1700	700	1700	1154	0.0000	101.9	83.0	8296
f-50-700_T1900	700	1900	1812	0.7571	79.1	47.2	635.2
f-50-700_T2100	700	2100	1874	0.9765	105.2	70.6	284.1
f-50-700_T2500	700	2500	1894	0.9989	370.3	276.7	10.3
f-50-700_T2700	700	2700	1893	0.9991	658.6	477.5	5.4
f-50-700_T2900	700	2900	1894	0.9996	802.8	623.5	5.1
f-50-900_T2100	900	2100	1876	0.9316	224.6	184.1	7090
f-50-900_T2500	900	2500	1884	0.9953	664.1	529.6	42.0
f-50-900_T2900	900	2900	1893	0.9992	1177	979.9	5.5

continued on next page

Table S12 (continued)

Simulation	$n(O_2)$	T (K)	n_{rows}	R^2	RMSE (molecule·ps)	MAE (molecule·ps)	MAPE (%)
**Repeat: strat42**	**Case: A**	**Model: ANN**					
f-50-1_T1700	1	1700	357	0.0000	264.3	176.9	17,695
f-50-1_T1900	1	1900	1318	0.0000	67.5	55.0	5502
f-50-1_T2100	1	2100	1816	-0.2589	365.7	225.0	529.4
f-50-1_T2500	1	2500	1892	0.9966	509.7	450.0	46.0
f-50-1_T2700	1	2700	1893	0.9998	300.2	225.9	3.5
f-50-1_T2900	1	2900	1892	0.9993	1045	833.2	12.1
f-50-100_T1500	100	1500	175	0.0000	177.5	131.3	13,127
f-50-100_T1700	100	1700	663	0.0000	80.5	69.6	6962
f-50-100_T1900	100	1900	1458	-37.4371	82.3	72.2	5064
f-50-100_T2100	100	2100	1827	0.4376	768.1	636.0	90.2
f-50-100_T2500	100	2500	1891	0.9974	542.3	444.6	10.0
f-50-100_T2700	100	2700	1893	0.9996	446.8	345.4	4.6
f-50-100_T2900	100	2900	1893	0.9996	886.5	721.8	6.1
f-50-300_T1500	300	1500	408	0.0000	193.5	181.8	18,179
f-50-300_T1700	300	1700	1215	0.0000	175.8	113.2	11,325
f-50-300_T1900	300	1900	1562	-135,581.5	246.5	144.4	13,455
f-50-300_T2100	300	2100	1869	0.8661	178.8	149.8	1043
f-50-300_T2300	300	2300	1889	0.9387	815.7	582.4	34.3
f-50-300_T2500	300	2500	1894	0.9992	327.1	235.7	54.3
f-50-300_T2700	300	2700	1893	0.9992	743.8	508.7	4.1
f-50-300_T2900	300	2900	1895	0.9998	634.8	557.6	5.8
f-50-500_T1500	500	1500	434	0.0000	119.3	113.0	11,296
f-50-500_T2100	500	2100	1886	0.8950	221.0	178.1	1732
f-50-500_T2300	500	2300	1891	0.9847	493.2	339.8	35.4
f-50-500_T2500	500	2500	1892	0.9978	477.9	366.6	8.7
f-50-500_T2700	500	2700	1895	0.9996	519.3	405.4	6.5
f-50-500_T2900	500	2900	1893	0.9998	657.5	506.8	6.0
f-50-700_T1700	700	1700	1154	0.0000	348.5	295.1	29,515
f-50-700_T1900	700	1900	1812	-2.4581	298.5	241.3	11,831
f-50-700_T2100	700	2100	1874	0.9871	78.0	55.9	556.1
f-50-700_T2500	700	2500	1894	0.9953	776.4	639.6	14.5
f-50-700_T2700	700	2700	1893	0.9992	612.6	498.9	8.0
f-50-700_T2900	700	2900	1894	0.9996	807.8	673.8	6.4
f-50-900_T2100	900	2100	1876	0.7658	415.7	361.2	14,135
f-50-900_T2500	900	2500	1884	0.9985	380.8	300.2	46.6

continued on next page

Table S12 (continued)

Simulation	$n(O_2)$	T (K)	n_{rows}	R^2	RMSE (molecule·ps)	MAE (molecule·ps)	MAPE (%)
f-50-900_T2900	900	2900	1893	0.9995	938.7	762.1	8.6
**Repeat: strat42**	**Case: A**	**Model: SVR**					
f-50-1_T1700	1	1700	357	0.0000	1175	1053	105,273
f-50-1_T1900	1	1900	1318	0.0000	1330	1210	121,022
f-50-1_T2100	1	2100	1816	-22.2912	1573	1115	6532
f-50-1_T2500	1	2500	1892	0.8602	3271	2688	178.4
f-50-1_T2700	1	2700	1893	0.7068	11,732	6904	53.2
f-50-1_T2900	1	2900	1892	-0.0516	41,482	28,786	55.3
f-50-100_T1500	100	1500	175	0.0000	867.1	754.5	75,445
f-50-100_T1700	100	1700	663	0.0000	946.4	711.4	71,140
f-50-100_T1900	100	1900	1458	-1087.9	438.2	314.4	18,422
f-50-100_T2100	100	2100	1827	-0.5767	1286	983.9	723.0
f-50-100_T2500	100	2500	1891	0.9732	1755	1120	81.2
f-50-100_T2700	100	2700	1893	0.9294	5788	3068	31.5
f-50-100_T2900	100	2900	1893	-0.0203	44,138	28,927	45.6
f-50-300_T1500	300	1500	408	0.0000	1158	1076	107,607
f-50-300_T1700	300	1700	1215	0.0000	449.9	380.9	38,090
f-50-300_T1900	300	1900	1562	-511,793.0	479.0	330.8	31,253
f-50-300_T2100	300	2100	1869	-15.2638	1970	1364	3429
f-50-300_T2300	300	2300	1889	0.8105	1435	1062	231.3
f-50-300_T2500	300	2500	1894	0.9809	1557	1206	374.9
f-50-300_T2700	300	2700	1893	0.7419	13,437	8416	29.7
f-50-300_T2900	300	2900	1895	0.1082	43,199	29,030	37.2
f-50-500_T1500	500	1500	434	0.0000	469.8	428.4	42,844
f-50-500_T2100	500	2100	1886	-2.7562	1322	930.6	4033
f-50-500_T2300	500	2300	1891	0.7089	2152	1671	350.3
f-50-500_T2500	500	2500	1892	0.9607	2019	1568	47.9
f-50-500_T2700	500	2700	1895	0.8229	10,600	6513	80.3
f-50-500_T2900	500	2900	1893	0.1997	38,687	26,555	56.6
f-50-700_T1700	700	1700	1154	0.0000	3926	3676	367,591
f-50-700_T1900	700	1900	1812	-264.4	2615	2361	28,769
f-50-700_T2100	700	2100	1874	-5.0932	1694	1270	8718
f-50-700_T2500	700	2500	1894	0.8498	4392	2535	107.5
f-50-700_T2700	700	2700	1893	0.7300	11,491	7013	47.8
f-50-700_T2900	700	2900	1894	0.1591	39,059	25,766	41.8
f-50-900_T2100	900	2100	1876	-25.5019	4422	3719	41,575

continued on next page

Table S12 (continued)

Simulation	$n(O_2)$	T (K)	n_{rows}	R^2	RMSE (molecule·ps)	MAE (molecule·ps)	MAPE (%)
f-50-900_T2500	900	2500	1884	0.8987	3088	2352	523.1
f-50-900_T2900	900	2900	1893	-0.0608	42,343	28,303	129.7
**Repeat: strat42 Case: A Model: GPR**							
f-50-1_T1700	1	1700	357	0.0000	23.1	15.5	1552
f-50-1_T1900	1	1900	1318	0.0000	24.8	19.9	1985
f-50-1_T2100	1	2100	1816	0.8585	122.6	103.5	115.2
f-50-1_T2500	1	2500	1892	0.9996	180.6	134.0	17.8
f-50-1_T2700	1	2700	1893	0.9999	177.4	143.9	3.1
f-50-1_T2900	1	2900	1892	0.9999	416.8	326.3	2.9
f-50-100_T1500	100	1500	175	0.0000	30.0	24.4	2439
f-50-100_T1700	100	1700	663	0.0000	6.1	3.9	386.2
f-50-100_T1900	100	1900	1458	-1.3211	20.2	13.9	746.0
f-50-100_T2100	100	2100	1827	0.9774	153.9	129.2	28.8
f-50-100_T2500	100	2500	1891	0.9999	76.5	63.7	5.7
f-50-100_T2700	100	2700	1893	0.9999	163.1	131.9	2.6
f-50-100_T2900	100	2900	1893	0.9999	385.4	332.6	3.1
f-50-300_T1500	300	1500	408	0.0000	12.5	11.4	1142
f-50-300_T1700	300	1700	1215	0.0000	17.0	14.7	1468
f-50-300_T1900	300	1900	1562	-4962.6	47.2	39.1	3899
f-50-300_T2100	300	2100	1869	0.9664	89.5	77.7	412.5
f-50-300_T2300	300	2300	1889	0.9978	154.7	125.7	15.0
f-50-300_T2500	300	2500	1894	0.9998	144.1	121.0	38.6
f-50-300_T2700	300	2700	1893	0.9999	190.0	158.5	4.4
f-50-300_T2900	300	2900	1895	1.0000	199.3	152.2	3.2
f-50-500_T1500	500	1500	434	0.0000	10.9	10.0	998.6
f-50-500_T2100	500	2100	1886	0.9848	84.2	64.8	278.3
f-50-500_T2300	500	2300	1891	0.9935	322.4	237.7	35.2
f-50-500_T2500	500	2500	1892	0.9999	92.0	76.1	6.4
f-50-500_T2700	500	2700	1895	1.0000	177.5	153.2	6.0
f-50-500_T2900	500	2900	1893	0.9999	508.1	455.4	3.7
f-50-700_T1700	700	1700	1154	0.0000	292.0	230.9	23,087
f-50-700_T1900	700	1900	1812	0.1715	146.1	112.4	4924
f-50-700_T2100	700	2100	1874	0.9899	68.9	58.3	243.1
f-50-700_T2500	700	2500	1894	0.9997	183.0	151.1	3.8
f-50-700_T2700	700	2700	1893	0.9999	257.7	196.8	4.3
f-50-700_T2900	700	2900	1894	1.0000	213.2	183.7	2.6

continued on next page

Table S12 (continued)

Simulation	$n(O_2)$	T (K)	n_{rows}	R^2	RMSE (molecule·ps)	MAE (molecule·ps)	MAPE (%)
f-50-900_T2100	900	2100	1876	0.6441	512.4	421.0	20,705
f-50-900_T2500	900	2500	1884	0.9979	441.8	380.5	9.1
f-50-900_T2900	900	2900	1893	0.9995	944.5	819.6	4.6
**Repeat: strat42 Case: A Model: persistence**							
f-50-1_T1700	1	1700	357	1.0000	0.0	0.0	0.0
f-50-1_T1900	1	1900	1318	1.0000	0.0	0.0	0.0
f-50-1_T2100	1	2100	1816	0.9631	62.6	54.9	23.7
f-50-1_T2500	1	2500	1892	0.9646	1647	1468	28.5
f-50-1_T2700	1	2700	1893	0.9644	4090	3642	27.3
f-50-1_T2900	1	2900	1892	0.9666	7390	6812	25.5
f-50-100_T1500	100	1500	175	1.0000	0.0	0.0	0.0
f-50-100_T1700	100	1700	663	1.0000	0.0	0.0	0.0
f-50-100_T1900	100	1900	1458	0.5926	8.5	3.1	11.9
f-50-100_T2100	100	2100	1827	0.9585	208.7	176.9	28.0
f-50-100_T2500	100	2500	1891	0.9636	2046	1794	29.2
f-50-100_T2700	100	2700	1893	0.9633	4174	3673	26.9
f-50-100_T2900	100	2900	1893	0.9656	8105	7371	25.8
f-50-300_T1500	300	1500	408	1.0000	0.0	0.0	0.0
f-50-300_T1700	300	1700	1215	1.0000	0.0	0.0	0.0
f-50-300_T1900	300	1900	1562	0.0667	0.6	0.1	3.4
f-50-300_T2100	300	2100	1869	0.9635	93.4	80.0	24.3
f-50-300_T2300	300	2300	1889	0.9586	670.4	572.4	28.8
f-50-300_T2500	300	2500	1894	0.9609	2228	1892	31.4
f-50-300_T2700	300	2700	1893	0.9631	5080	4441	28.5
f-50-300_T2900	300	2900	1895	0.9655	8494	7665	26.3
f-50-500_T1500	500	1500	434	1.0000	0.0	0.0	0.0
f-50-500_T2100	500	2100	1886	0.9467	157.5	113.8	27.3
f-50-500_T2300	500	2300	1891	0.9589	808.1	663.3	31.3
f-50-500_T2500	500	2500	1892	0.9581	2085	1751	28.4
f-50-500_T2700	500	2700	1895	0.9622	4897	4216	29.2
f-50-500_T2900	500	2900	1893	0.9655	8037	7244	27.0
f-50-700_T1700	700	1700	1154	1.0000	0.0	0.0	0.0
f-50-700_T1900	700	1900	1812	0.9550	34.0	27.5	21.3
f-50-700_T2100	700	2100	1874	0.9562	143.7	115.1	27.1
f-50-700_T2500	700	2500	1894	0.9619	2213	1916	28.9
f-50-700_T2700	700	2700	1893	0.9621	4303	3713	28.5

continued on next page

Table S12 (continued)

Simulation	$n(O_2)$	T (K)	n_{rows}	R^2	RMSE (molecule·ps)	MAE (molecule·ps)	MAPE (%)
f-50-700_T2900	700	2900	1894	0.9644	8040	7131	27.6
f-50-900_T2100	900	2100	1876	0.9632	164.8	133.9	23.0
f-50-900_T2500	900	2500	1884	0.9600	1940	1651	31.2
f-50-900_T2900	900	2900	1893	0.9640	7798	6850	28.8
**Repeat: strat42 Case: A Model: physical_MLR**							
f-50-1_T1700	1	1700	357	0.0000	21,566	17,398	1,739,781
f-50-1_T1900	1	1900	1318	0.0000	14,648	12,497	1,249,742
f-50-1_T2100	1	2100	1816	-1730.0	13,559	11,585	147,699
f-50-1_T2500	1	2500	1892	-1.6175	14,153	12,619	434.6
f-50-1_T2700	1	2700	1893	0.7457	10,926	9932	318.7
f-50-1_T2900	1	2900	1892	0.4111	31,043	23,659	162.3
f-50-100_T1500	100	1500	175	0.0000	29,543	25,311	2,531,088
f-50-100_T1700	100	1700	663	0.0000	17,427	14,085	1,408,508
f-50-100_T1900	100	1900	1458	-1.25×10^6	14,831	12,488	993,083
f-50-100_T2100	100	2100	1827	-156.6	12,860	11,070	29,396
f-50-100_T2500	100	2500	1891	0.2824	9083	8318	340.0
f-50-100_T2700	100	2700	1893	0.7723	10,396	9517	247.5
f-50-100_T2900	100	2900	1893	0.3538	35,125	26,178	161.2
f-50-300_T1500	300	1500	408	0.0000	29,146	25,738	2,573,827
f-50-300_T1700	300	1700	1215	0.0000	19,579	15,437	1,543,682
f-50-300_T1900	300	1900	1562	-4.97×10^8	14,920	12,323	1,208,185
f-50-300_T2100	300	2100	1869	-967.4	15,204	13,050	282,629
f-50-300_T2300	300	2300	1889	-20.2023	15,174	12,765	4177
f-50-300_T2500	300	2500	1894	0.0293	11,105	10,113	2030
f-50-300_T2700	300	2700	1893	0.7839	12,295	10,435	281.8
f-50-300_T2900	300	2900	1895	0.4108	35,114	26,585	183.5
f-50-500_T1500	500	1500	434	0.0000	28,569	24,748	2,474,841
f-50-500_T2100	500	2100	1886	-462.2	14,679	12,705	314,182
f-50-500_T2300	500	2300	1891	-12.6143	14,715	12,531	7344
f-50-500_T2500	500	2500	1892	-0.5766	12,792	11,476	318.1
f-50-500_T2700	500	2700	1895	0.7506	12,579	11,032	436.9
f-50-500_T2900	500	2900	1893	0.4780	31,245	24,115	245.7
f-50-700_T1700	700	1700	1154	0.0000	19,515	15,101	1,510,135
f-50-700_T1900	700	1900	1812	-11,473.6	17,197	14,181	663,354
f-50-700_T2100	700	2100	1874	-451.6	14,604	12,611	241,556
f-50-700_T2500	700	2500	1894	0.3009	9475	8582	421.6

continued on next page

Table S12 (continued)

Simulation	$n(O_2)$	T (K)	n_{rows}	R^2	RMSE (molecule·ps)	MAE (molecule·ps)	MAPE (%)
f-50-700_T2700	700	2700	1893	0.7727	10,542	9654	333.5
f-50-700_T2900	700	2900	1894	0.5215	29,463	22,958	287.8
f-50-900_T2100	900	2100	1876	-277.8	14,344	12,334	517,333
f-50-900_T2500	900	2500	1884	-0.9793	13,649	12,712	674.5
f-50-900_T2900	900	2900	1893	0.5462	27,694	21,848	433.4
**Repeat: strat42 Case: A Model: physical_RF**							
f-50-1_T1700	1	1700	357	0.0000	0.0	0.0	0.1
f-50-1_T1900	1	1900	1318	0.0000	14.2	5.0	501.4
f-50-1_T2100	1	2100	1816	0.5039	229.5	167.4	45.9
f-50-1_T2500	1	2500	1892	0.8922	2872	1931	14.3
f-50-1_T2700	1	2700	1893	0.9954	1473	593.7	4.5
f-50-1_T2900	1	2900	1892	0.9712	6868	4557	8.8
f-50-100_T1500	100	1500	175	0.0000	0.0	0.0	0.4
f-50-100_T1700	100	1700	663	0.0000	1.1	0.3	31.6
f-50-100_T1900	100	1900	1458	-0.1135	14.0	4.9	22.0
f-50-100_T2100	100	2100	1827	0.3861	802.5	594.4	58.9
f-50-100_T2500	100	2500	1891	0.9634	2052	1477	10.6
f-50-100_T2700	100	2700	1893	0.9969	1218	663.6	6.2
f-50-100_T2900	100	2900	1893	0.9967	2523	1985	6.6
f-50-300_T1500	300	1500	408	0.0000	0.0	0.0	1.5
f-50-300_T1700	300	1700	1215	0.0000	0.2	0.0	3.1
f-50-300_T1900	300	1900	1562	-413.5	13.6	4.8	417.0
f-50-300_T2100	300	2100	1869	-1.5989	787.6	575.6	480.4
f-50-300_T2300	300	2300	1889	0.9427	788.5	623.7	25.4
f-50-300_T2500	300	2500	1894	0.9943	848.5	534.1	38.4
f-50-300_T2700	300	2700	1893	0.9349	6746	4900	16.1
f-50-300_T2900	300	2900	1895	0.9947	3330	2471	5.6
f-50-500_T1500	500	1500	434	0.0000	0.2	0.0	3.5
f-50-500_T2100	500	2100	1886	0.8639	251.6	177.0	76.3
f-50-500_T2300	500	2300	1891	0.9606	791.8	627.7	46.1
f-50-500_T2500	500	2500	1892	0.9696	1777	1388	13.6
f-50-500_T2700	500	2700	1895	0.9866	2910	2369	13.4
f-50-500_T2900	500	2900	1893	0.9932	3560	2964	11.4
f-50-700_T1700	700	1700	1154	0.0000	0.0	0.0	2.4
f-50-700_T1900	700	1900	1812	-1.1424	235.0	171.8	80.3
f-50-700_T2100	700	2100	1874	0.9695	119.9	93.6	42.5

continued on next page

Table S12 (continued)

Simulation	$n(O_2)$	T (K)	n_{rows}	R^2	RMSE (molecule·ps)	MAE (molecule·ps)	MAPE (%)
f-50-700_T2500	700	2500	1894	0.9635	2164	1772	15.4
f-50-700_T2700	700	2700	1893	0.9571	4582	3401	12.7
f-50-700_T2900	700	2900	1894	0.9945	3172	2723	11.5
f-50-900_T2100	900	2100	1876	0.8903	284.5	212.0	430.7
f-50-900_T2500	900	2500	1884	0.9172	2792	2262	46.0
f-50-900_T2900	900	2900	1893	0.9916	3758	3198	18.0
**Repeat: strat42**	**Case: B**	**Model: MLR**					
f-50-1_T1700	1	1700	357	0.0000	13,723	13,398	1,339,760
f-50-1_T1900	1	1900	1318	0.0000	22,496	21,050	2,105,050
f-50-1_T2100	1	2100	1816	-2617.2	16,675	16,010	69,207
f-50-1_T2500	1	2500	1892	0.9407	2131	1629	687.1
f-50-1_T2700	1	2700	1893	-0.1342	23,076	20,873	417.2
f-50-1_T2900	1	2900	1892	-0.2891	45,929	36,414	110.1
f-50-100_T1500	100	1500	175	0.0000	25,834	25,738	2,573,783
f-50-100_T1700	100	1700	663	0.0000	17,909	16,269	1,626,937
f-50-100_T1900	100	1900	1458	-234,410.2	6430	5758	442,920
f-50-100_T2100	100	2100	1827	-179.8	13,772	12,855	6876
f-50-100_T2500	100	2500	1891	0.9395	2638	1974	266.9
f-50-100_T2700	100	2700	1893	0.9133	6415	5110	165.3
f-50-100_T2900	100	2900	1893	-0.0852	45,521	32,957	68.1
f-50-300_T1500	300	1500	408	0.0000	14,338	13,208	1,320,770
f-50-300_T1700	300	1700	1215	0.0000	10,843	9064	906,408
f-50-300_T1900	300	1900	1562	-2.32×10^8	10,196	8465	820,065
f-50-300_T2100	300	2100	1869	-218.8	7244	6419	107,572
f-50-300_T2300	300	2300	1889	-34.3962	19,606	16,596	2333
f-50-300_T2500	300	2500	1894	0.4063	8684	7244	837.6
f-50-300_T2700	300	2700	1893	0.7159	14,096	11,799	256.4
f-50-300_T2900	300	2900	1895	0.3403	37,155	26,447	107.5
f-50-500_T1500	500	1500	434	0.0000	7550	6421	642,057
f-50-500_T2100	500	2100	1886	-983.2	21,396	18,193	197,041
f-50-500_T2300	500	2300	1891	-3.1478	8122	6191	6582
f-50-500_T2500	500	2500	1892	-0.1526	10,938	8747	1632
f-50-500_T2700	500	2700	1895	0.6166	15,596	13,071	634.3
f-50-500_T2900	500	2900	1893	-0.0498	44,310	31,861	196.7
f-50-700_T1700	700	1700	1154	0.0000	17,216	16,326	1,632,605
f-50-700_T1900	700	1900	1812	-5400.7	11,799	10,874	124,844

continued on next page

Table S12 (continued)

Simulation	$n(O_2)$	T (K)	n_{rows}	R^2	RMSE (molecule·ps)	MAE (molecule·ps)	MAPE (%)
f-50-700_T2100	700	2100	1874	-882.1	20,399	17,990	272,047
f-50-700_T2500	700	2500	1894	-0.0773	11,762	10,493	1908
f-50-700_T2700	700	2700	1893	0.7731	10,533	7845	983.7
f-50-700_T2900	700	2900	1894	0.7966	19,209	14,698	476.9
f-50-900_T2100	900	2100	1876	-329.8	15,625	11,947	83,327
f-50-900_T2500	900	2500	1884	-3.5670	20,732	19,417	7768
f-50-900_T2900	900	2900	1893	-1.5023	65,033	50,036	722.6
**Repeat: strat42**	**Case: B**	**Model: DT**					
f-50-1_T1700	1	1700	357	1.0000	0.0	0.0	0.0
f-50-1_T1900	1	1900	1318	0.0000	15.9	7.3	728.0
f-50-1_T2100	1	2100	1816	-9226.0	31,305	6647	708.0
f-50-1_T2500	1	2500	1892	0.1712	7964	6397	80.8
f-50-1_T2700	1	2700	1893	0.0185	21,466	16,936	59.8
f-50-1_T2900	1	2900	1892	0.3666	32,194	25,431	46.4
f-50-100_T1500	100	1500	175	1.0000	0.0	0.0	0.0
f-50-100_T1700	100	1700	663	0.0000	1.1	0.1	13.2
f-50-100_T1900	100	1900	1458	-0.0946	13.9	4.8	34.6
f-50-100_T2100	100	2100	1827	-901.1	30,766	7249	290.7
f-50-100_T2500	100	2500	1891	-2.1122	18,916	7352	71.8
f-50-100_T2700	100	2700	1893	0.5660	14,352	9185	38.4
f-50-100_T2900	100	2900	1893	0.3139	36,194	27,618	48.6
f-50-300_T1500	300	1500	408	1.0000	0.0	0.0	0.0
f-50-300_T1700	300	1700	1215	0.0000	0.7	0.3	25.8
f-50-300_T1900	300	1900	1562	-100,502.7	212.2	135.3	13,030
f-50-300_T2100	300	2100	1869	0.4792	352.6	233.9	489.0
f-50-300_T2300	300	2300	1889	-0.2321	3658	2093	45.8
f-50-300_T2500	300	2500	1894	0.0361	11,066	4442	93.0
f-50-300_T2700	300	2700	1893	0.1924	23,766	17,801	51.1
f-50-300_T2900	300	2900	1895	-0.5015	56,054	43,271	67.2
f-50-500_T1500	500	1500	434	1.0000	0.0	0.0	0.0
f-50-500_T2100	500	2100	1886	-5.1911	1697	1024	171.3
f-50-500_T2300	500	2300	1891	-4.1214	9025	4626	90.7
f-50-500_T2500	500	2500	1892	0.9042	3153	2164	22.2
f-50-500_T2700	500	2700	1895	0.8440	9949	7388	38.4
f-50-500_T2900	500	2900	1893	0.5532	28,908	21,246	40.7
f-50-700_T1700	700	1700	1154	1.0000	0.0	0.0	0.0

continued on next page

Table S12 (continued)

Simulation	$n(O_2)$	T (K)	n_{rows}	R^2	RMSE (molecule·ps)	MAE (molecule·ps)	MAPE (%)
f-50-700_T1900	700	1900	1812	-2.1958	287.0	208.8	914.4
f-50-700_T2100	700	2100	1874	-4.9594	1676	629.6	84.7
f-50-700_T2500	700	2500	1894	0.8993	3597	2533	24.8
f-50-700_T2700	700	2700	1893	-0.0861	23,047	15,498	45.3
f-50-700_T2900	700	2900	1894	0.9872	4817	3162	13.7
f-50-900_T2100	900	2100	1876	-61.9189	6814	3912	88,914
f-50-900_T2500	900	2500	1884	-3.6912	21,012	11,091	78.8
f-50-900_T2900	900	2900	1893	-0.3116	47,083	32,090	49.7
**Repeat: strat42**	**Case: B**	**Model: RF**					
f-50-1_T1700	1	1700	357	0.0000	488.8	102.8	10,283
f-50-1_T1900	1	1900	1318	0.0000	274.6	167.4	16,740
f-50-1_T2100	1	2100	1816	-549.1	7643	3800	588.1
f-50-1_T2500	1	2500	1892	0.6365	5274	4272	53.9
f-50-1_T2700	1	2700	1893	0.9342	5556	3477	14.7
f-50-1_T2900	1	2900	1892	0.4550	29,865	21,477	33.0
f-50-100_T1500	100	1500	175	0.0000	12.7	9.7	974.8
f-50-100_T1700	100	1700	663	0.0000	383.1	254.7	25,469
f-50-100_T1900	100	1900	1458	-482.4	292.0	205.6	13,888
f-50-100_T2100	100	2100	1827	-21.2047	4827	2697	176.3
f-50-100_T2500	100	2500	1891	0.7289	5583	4562	55.2
f-50-100_T2700	100	2700	1893	0.8883	7280	4617	15.1
f-50-100_T2900	100	2900	1893	0.2010	39,059	28,611	43.1
f-50-300_T1500	300	1500	408	0.0000	0.4	0.0	3.7
f-50-300_T1700	300	1700	1215	0.0000	227.0	74.7	7468
f-50-300_T1900	300	1900	1562	-1.46×10^8	8085	5826	561,829
f-50-300_T2100	300	2100	1869	-46.7611	3376	2144	469.9
f-50-300_T2300	300	2300	1889	-4.2910	7580	5445	109.1
f-50-300_T2500	300	2500	1894	0.7857	5218	3679	195.9
f-50-300_T2700	300	2700	1893	-0.2289	29,318	20,261	49.1
f-50-300_T2900	300	2900	1895	-0.7591	60,672	45,552	66.9
f-50-500_T1500	500	1500	434	0.0000	3784	2800	279,984
f-50-500_T2100	500	2100	1886	-40.2446	4380	3026	903.6
f-50-500_T2300	500	2300	1891	-1.8251	6703	3400	73.6
f-50-500_T2500	500	2500	1892	0.2765	8665	6374	43.9
f-50-500_T2700	500	2700	1895	0.9642	4767	3020	19.2
f-50-500_T2900	500	2900	1893	0.2659	37,053	26,637	46.5

continued on next page

Table S12 (continued)

Simulation	$n(O_2)$	T (K)	n_{rows}	R^2	RMSE (molecule·ps)	MAE (molecule·ps)	MAPE (%)
f-50-700_T1700	700	1700	1154	0.0000	2438	1801	180,099
f-50-700_T1900	700	1900	1812	-139.6	1903	1354	1699
f-50-700_T2100	700	2100	1874	-7.2338	1970	1136	352.2
f-50-700_T2500	700	2500	1894	0.8076	4970	3197	22.4
f-50-700_T2700	700	2700	1893	0.9270	5974	3822	14.2
f-50-700_T2900	700	2900	1894	0.9428	10,188	7300	17.3
f-50-900_T2100	900	2100	1876	-32.3920	4964	4179	28,411
f-50-900_T2500	900	2500	1884	-4.3651	22,471	14,938	96.4
f-50-900_T2900	900	2900	1893	0.1592	37,696	23,604	31.3
**Repeat: strat42**	**Case: B**	**Model: XGBoost**					
f-50-1_T1700	1	1700	357	0.0000	361.2	256.3	25,630
f-50-1_T1900	1	1900	1318	0.0000	1760	1261	126,089
f-50-1_T2100	1	2100	1816	-645.2	8284	3670	1722
f-50-1_T2500	1	2500	1892	0.6163	5419	4171	76.4
f-50-1_T2700	1	2700	1893	0.9503	4830	3521	39.2
f-50-1_T2900	1	2900	1892	0.5670	26,618	19,662	45.3
f-50-100_T1500	100	1500	175	0.0000	178.8	151.5	15,153
f-50-100_T1700	100	1700	663	0.0000	6255	3570	356,962
f-50-100_T1900	100	1900	1458	-1441.8	504.5	434.0	32,978
f-50-100_T2100	100	2100	1827	-14.7637	4067	2140	356.4
f-50-100_T2500	100	2500	1891	0.8204	4544	2873	89.0
f-50-100_T2700	100	2700	1893	0.8216	9202	6283	36.1
f-50-100_T2900	100	2900	1893	0.6578	25,562	20,933	56.7
f-50-300_T1500	300	1500	408	0.0000	815.6	737.6	73,757
f-50-300_T1700	300	1700	1215	0.0000	5351	3577	357,671
f-50-300_T1900	300	1900	1562	-2.32×10^7	3221	2306	228,101
f-50-300_T2100	300	2100	1869	-10.7878	1677	1263	775.1
f-50-300_T2300	300	2300	1889	-5.0757	8123	5467	241.7
f-50-300_T2500	300	2500	1894	-1.2141	16,771	10,303	397.3
f-50-300_T2700	300	2700	1893	-0.2424	29,477	20,963	76.3
f-50-300_T2900	300	2900	1895	-0.6817	59,322	44,598	74.3
f-50-500_T1500	500	1500	434	0.0000	4217	2929	292,900
f-50-500_T2100	500	2100	1886	-16.5367	2856	1896	5280
f-50-500_T2300	500	2300	1891	-1.7003	6553	3129	114.3
f-50-500_T2500	500	2500	1892	0.6649	5898	3870	30.2
f-50-500_T2700	500	2700	1895	0.7895	11,556	6988	44.0

continued on next page

Table S12 (continued)

Simulation	$n(O_2)$	T (K)	n_{rows}	R^2	RMSE (molecule·ps)	MAE (molecule·ps)	MAPE (%)
f-50-500_T2900	500	2900	1893	-0.1913	47,201	34,069	55.9
f-50-700_T1700	700	1700	1154	0.0000	1530	1221	122,109
f-50-700_T1900	700	1900	1812	-91.1756	1541	1114	7931
f-50-700_T2100	700	2100	1874	-8.4294	2108	1367	1996
f-50-700_T2500	700	2500	1894	0.8409	4520	3106	47.0
f-50-700_T2700	700	2700	1893	0.8563	8383	5502	21.8
f-50-700_T2900	700	2900	1894	0.9052	13,115	9022	19.5
f-50-900_T2100	900	2100	1876	-23.0501	4213	3216	92,079
f-50-900_T2500	900	2500	1884	-3.3482	20,230	11,236	108.0
f-50-900_T2900	900	2900	1893	0.7123	22,050	14,539	34.6
**Repeat: strat42**	**Case: B**	**Model: CatBoost**					
f-50-1_T1700	1	1700	357	0.0000	222.1	163.2	16,316
f-50-1_T1900	1	1900	1318	0.0000	568.2	428.1	42,810
f-50-1_T2100	1	2100	1816	-541.8	7593	5027	1311
f-50-1_T2500	1	2500	1892	0.0715	8429	5769	53.0
f-50-1_T2700	1	2700	1893	0.9679	3880	3301	29.4
f-50-1_T2900	1	2900	1892	0.4435	30,176	23,327	44.9
f-50-100_T1500	100	1500	175	0.0000	602.7	412.2	41,223
f-50-100_T1700	100	1700	663	0.0000	1160	729.8	72,976
f-50-100_T1900	100	1900	1458	-1302.5	479.5	363.3	28,595
f-50-100_T2100	100	2100	1827	-13.3300	3878	2430	557.5
f-50-100_T2500	100	2500	1891	-0.6002	13,563	8350	121.5
f-50-100_T2700	100	2700	1893	0.8164	9334	7083	32.2
f-50-100_T2900	100	2900	1893	0.5458	29,449	23,226	41.7
f-50-300_T1500	300	1500	408	0.0000	447.7	319.1	31,910
f-50-300_T1700	300	1700	1215	0.0000	1615	1104	110,358
f-50-300_T1900	300	1900	1562	-2.84×10^7	3568	2280	227,552
f-50-300_T2100	300	2100	1869	-21.5986	2323	1722	2484
f-50-300_T2300	300	2300	1889	-4.7873	7928	4757	157.5
f-50-300_T2500	300	2500	1894	-0.9299	15,658	8349	447.6
f-50-300_T2700	300	2700	1893	-0.1590	28,471	20,309	62.5
f-50-300_T2900	300	2900	1895	-0.8892	62,876	47,172	70.3
f-50-500_T1500	500	1500	434	0.0000	1858	1228	122,755
f-50-500_T2100	500	2100	1886	-22.1037	3278	2323	2748
f-50-500_T2300	500	2300	1891	-2.2327	7170	3279	121.8
f-50-500_T2500	500	2500	1892	0.4675	7434	4793	45.7

continued on next page

Table S12 (continued)

Simulation	$n(O_2)$	T (K)	n_{rows}	R^2	RMSE (molecule·ps)	MAE (molecule·ps)	MAPE (%)
f-50-500_T2700	500	2700	1895	0.9612	4961	3618	28.0
f-50-500_T2900	500	2900	1893	0.0567	42,003	28,405	47.4
f-50-700_T1700	700	1700	1154	0.0000	3230	2040	203,951
f-50-700_T1900	700	1900	1812	-90.4009	1535	988.8	7238
f-50-700_T2100	700	2100	1874	-1.5169	1089	743.6	2031
f-50-700_T2500	700	2500	1894	0.9224	3158	1907	26.1
f-50-700_T2700	700	2700	1893	0.9430	5278	3176	16.4
f-50-700_T2900	700	2900	1894	0.9486	9654	7328	18.1
f-50-900_T2100	900	2100	1876	-11.8854	3084	2066	28,880
f-50-900_T2500	900	2500	1884	-5.0529	23,868	14,882	98.1
f-50-900_T2900	900	2900	1893	0.8821	14,114	9023	19.9
**Repeat: strat42 Case: B Model: ANN**							
f-50-1_T1700	1	1700	357	0.0000	2754	1849	184,905
f-50-1_T1900	1	1900	1318	0.0000	2064	1402	140,160
f-50-1_T2100	1	2100	1816	-8.7268	1016	734.3	1644
f-50-1_T2500	1	2500	1892	0.9552	1851	1432	66.7
f-50-1_T2700	1	2700	1893	0.9538	4658	3154	34.3
f-50-1_T2900	1	2900	1892	0.9150	11,791	7492	17.1
f-50-100_T1500	100	1500	175	0.0000	2848	1740	174,033
f-50-100_T1700	100	1700	663	0.0000	755.6	665.4	66,538
f-50-100_T1900	100	1900	1458	-58.1654	102.2	97.2	7865
f-50-100_T2100	100	2100	1827	-1.2112	1523	1212	654.7
f-50-100_T2500	100	2500	1891	0.9606	2128	1589	64.2
f-50-100_T2700	100	2700	1893	0.9565	4544	2917	18.9
f-50-100_T2900	100	2900	1893	0.9606	8678	6527	24.1
f-50-300_T1500	300	1500	408	0.0000	1183	1027	102,746
f-50-300_T1700	300	1700	1215	0.0000	433.2	410.7	41,069
f-50-300_T1900	300	1900	1562	-124,922.3	236.6	186.7	18,208
f-50-300_T2100	300	2100	1869	-4.9098	1188	600.8	1460
f-50-300_T2300	300	2300	1889	-1.2301	4921	3003	81.3
f-50-300_T2500	300	2500	1894	0.9192	3204	2321	313.3
f-50-300_T2700	300	2700	1893	0.8471	10,340	6286	22.1
f-50-300_T2900	300	2900	1895	0.9090	13,797	9004	17.3
f-50-500_T1500	500	1500	434	0.0000	1704	1441	144,092
f-50-500_T2100	500	2100	1886	-0.6291	870.5	475.0	7885
f-50-500_T2300	500	2300	1891	0.6509	2356	1358	72.2

continued on next page

Table S12 (continued)

Simulation	$n(O_2)$	T (K)	n_{rows}	R^2	RMSE (molecule·ps)	MAE (molecule·ps)	MAPE (%)
f-50-500_T2500	500	2500	1892	0.9555	2149	1456	25.2
f-50-500_T2700	500	2700	1895	0.9843	3161	2421	25.3
f-50-500_T2900	500	2900	1893	0.6175	26,745	18,186	50.3
f-50-700_T1700	700	1700	1154	0.0000	7619	6407	640,745
f-50-700_T1900	700	1900	1812	-764.8	4442	3289	22,892
f-50-700_T2100	700	2100	1874	0.9154	199.7	150.5	141.1
f-50-700_T2500	700	2500	1894	0.9393	2792	1803	25.4
f-50-700_T2700	700	2700	1893	0.9871	2515	1611	13.3
f-50-700_T2900	700	2900	1894	0.9894	4390	3283	13.5
f-50-900_T2100	900	2100	1876	-2.4569	1597	1316	16,691
f-50-900_T2500	900	2500	1884	0.9201	2742	1709	58.0
f-50-900_T2900	900	2900	1893	0.9598	8244	6068	19.2
**Repeat: strat42 Case: B Model: SVR**							
f-50-1_T1700	1	1700	357	0.0000	535.5	483.8	48,376
f-50-1_T1900	1	1900	1318	0.0000	609.9	591.0	59,101
f-50-1_T2100	1	2100	1816	-119.9	3583	2395	4041
f-50-1_T2500	1	2500	1892	0.8554	3327	2896	139.5
f-50-1_T2700	1	2700	1893	0.4522	16,036	9398	43.3
f-50-1_T2900	1	2900	1892	-0.4951	49,464	36,601	57.5
f-50-100_T1500	100	1500	175	0.0000	544.7	516.8	51,684
f-50-100_T1700	100	1700	663	0.0000	249.0	142.1	14,207
f-50-100_T1900	100	1900	1458	-520.3	303.2	227.5	14,279
f-50-100_T2100	100	2100	1827	-20.3746	4736	3311	520.6
f-50-100_T2500	100	2500	1891	0.8455	4215	2685	42.0
f-50-100_T2700	100	2700	1893	0.4211	16,574	10,425	31.0
f-50-100_T2900	100	2900	1893	-0.5333	54,109	39,033	55.1
f-50-300_T1500	300	1500	408	0.0000	3024	2658	265,793
f-50-300_T1700	300	1700	1215	0.0000	1174	846.8	84,683
f-50-300_T1900	300	1900	1562	-7.06×10^6	1779	1066	99,994
f-50-300_T2100	300	2100	1869	-246.2	7681	5314	1874
f-50-300_T2300	300	2300	1889	-2.2667	5956	4876	173.7
f-50-300_T2500	300	2500	1894	0.8780	3936	3046	125.3
f-50-300_T2700	300	2700	1893	-0.0900	27,611	18,460	46.9
f-50-300_T2900	300	2900	1895	-0.6469	58,706	42,536	57.9
f-50-500_T1500	500	1500	434	0.0000	632.6	438.5	43,850
f-50-500_T2100	500	2100	1886	-107.8	7115	5079	5183

continued on next page

Table S12 (continued)

Simulation	$n(O_2)$	T (K)	n_{rows}	R^2	RMSE (molecule·ps)	MAE (molecule·ps)	MAPE (%)
f-50-500_T2300	500	2300	1891	-1.3614	6128	4632	302.3
f-50-500_T2500	500	2500	1892	0.8400	4076	3234	62.9
f-50-500_T2700	500	2700	1895	0.0693	24,298	15,713	45.8
f-50-500_T2900	500	2900	1893	-0.7265	56,824	41,467	66.2
f-50-700_T1700	700	1700	1154	0.0000	6455	6093	609,309
f-50-700_T1900	700	1900	1812	-356.9	3037	2886	42,531
f-50-700_T2100	700	2100	1874	-15.9037	2822	1844	3522
f-50-700_T2500	700	2500	1894	0.8392	4545	2976	39.6
f-50-700_T2700	700	2700	1893	0.4554	16,320	9814	45.6
f-50-700_T2900	700	2900	1894	-0.1680	46,033	31,345	47.6
f-50-900_T2100	900	2100	1876	-13.2070	3238	2728	50,481
f-50-900_T2500	900	2500	1884	0.8489	3771	2842	158.6
f-50-900_T2900	900	2900	1893	-0.3733	48,177	32,782	71.3
**Repeat: strat42 Case: B Model: GPR**							
f-50-1_T1700	1	1700	357	0.0000	622.3	550.8	55,079
f-50-1_T1900	1	1900	1318	0.0000	387.0	262.2	26,216
f-50-1_T2100	1	2100	1816	-91.3016	3131	1876	722.5
f-50-1_T2500	1	2500	1892	-0.0227	8847	6459	68.5
f-50-1_T2700	1	2700	1893	0.9231	6007	3772	14.1
f-50-1_T2900	1	2900	1892	0.6809	22,850	17,914	34.9
f-50-100_T1500	100	1500	175	0.0000	1891	1560	156,019
f-50-100_T1700	100	1700	663	0.0000	533.1	367.1	36,709
f-50-100_T1900	100	1900	1458	-7161.6	1124	651.7	28,650
f-50-100_T2100	100	2100	1827	-10.7553	3512	2504	234.6
f-50-100_T2500	100	2500	1891	0.7582	5272	3529	27.7
f-50-100_T2700	100	2700	1893	0.7533	10,821	7220	19.6
f-50-100_T2900	100	2900	1893	0.9392	10,772	8960	19.5
f-50-300_T1500	300	1500	408	0.0000	1410	1078	107,766
f-50-300_T1700	300	1700	1215	0.0000	3620	2488	248,798
f-50-300_T1900	300	1900	1562	-5.97×10^7	5175	4015	392,650
f-50-300_T2100	300	2100	1869	-78.0796	4345	3004	2476
f-50-300_T2300	300	2300	1889	-1.3430	5044	3438	85.0
f-50-300_T2500	300	2500	1894	0.8918	3708	2825	155.6
f-50-300_T2700	300	2700	1893	0.9191	7521	4746	14.2
f-50-300_T2900	300	2900	1895	0.8370	18,470	12,393	19.4
f-50-500_T1500	500	1500	434	0.0000	590.0	451.4	45,143

continued on next page

Table S12 (continued)

Simulation	$n(O_2)$	T (K)	n_{rows}	R^2	RMSE (molecule·ps)	MAE (molecule·ps)	MAPE (%)
f-50-500_T2100	500	2100	1886	-19.7233	3105	2179	2195
f-50-500_T2300	500	2300	1891	0.5584	2650	2018	119.8
f-50-500_T2500	500	2500	1892	0.9281	2732	1970	38.1
f-50-500_T2700	500	2700	1895	0.9689	4440	3324	22.4
f-50-500_T2900	500	2900	1893	0.4426	32,286	23,304	43.1
f-50-700_T1700	700	1700	1154	0.0000	3531	3247	324,741
f-50-700_T1900	700	1900	1812	-124.9	1801	1606	29,392
f-50-700_T2100	700	2100	1874	-2.6314	1308	885.0	608.0
f-50-700_T2500	700	2500	1894	0.8923	3719	2399	17.2
f-50-700_T2700	700	2700	1893	0.9919	1988	1288	10.0
f-50-700_T2900	700	2900	1894	0.9799	6035	4591	10.4
f-50-900_T2100	900	2100	1876	-1.1887	1271	1074	45,366
f-50-900_T2500	900	2500	1884	-0.7348	12,778	7544	60.8
f-50-900_T2900	900	2900	1893	0.6734	23,495	16,971	41.4
**Repeat: strat42**	**Case: B**	**Model: persistence**					
f-50-1_T1700	1	1700	357	1.0000	0.0	0.0	0.0
f-50-1_T1900	1	1900	1318	1.0000	0.0	0.0	0.0
f-50-1_T2100	1	2100	1816	0.9631	62.6	54.9	23.7
f-50-1_T2500	1	2500	1892	0.9646	1647	1468	28.5
f-50-1_T2700	1	2700	1893	0.9644	4090	3642	27.3
f-50-1_T2900	1	2900	1892	0.9666	7390	6812	25.5
f-50-100_T1500	100	1500	175	1.0000	0.0	0.0	0.0
f-50-100_T1700	100	1700	663	1.0000	0.0	0.0	0.0
f-50-100_T1900	100	1900	1458	0.5926	8.5	3.1	11.9
f-50-100_T2100	100	2100	1827	0.9585	208.7	176.9	28.0
f-50-100_T2500	100	2500	1891	0.9636	2046	1794	29.2
f-50-100_T2700	100	2700	1893	0.9633	4174	3673	26.9
f-50-100_T2900	100	2900	1893	0.9656	8105	7371	25.8
f-50-300_T1500	300	1500	408	1.0000	0.0	0.0	0.0
f-50-300_T1700	300	1700	1215	1.0000	0.0	0.0	0.0
f-50-300_T1900	300	1900	1562	0.0667	0.6	0.1	3.4
f-50-300_T2100	300	2100	1869	0.9635	93.4	80.0	24.3
f-50-300_T2300	300	2300	1889	0.9586	670.4	572.4	28.8
f-50-300_T2500	300	2500	1894	0.9609	2228	1892	31.4
f-50-300_T2700	300	2700	1893	0.9631	5080	4441	28.5
f-50-300_T2900	300	2900	1895	0.9655	8494	7665	26.3

continued on next page

Table S12 (continued)

Simulation	$n(O_2)$	T (K)	n_{rows}	R^2	RMSE (molecule·ps)	MAE (molecule·ps)	MAPE (%)
f-50-500_T1500	500	1500	434	1.0000	0.0	0.0	0.0
f-50-500_T2100	500	2100	1886	0.9467	157.5	113.8	27.3
f-50-500_T2300	500	2300	1891	0.9589	808.1	663.3	31.3
f-50-500_T2500	500	2500	1892	0.9581	2085	1751	28.4
f-50-500_T2700	500	2700	1895	0.9622	4897	4216	29.2
f-50-500_T2900	500	2900	1893	0.9655	8037	7244	27.0
f-50-700_T1700	700	1700	1154	1.0000	0.0	0.0	0.0
f-50-700_T1900	700	1900	1812	0.9550	34.0	27.5	21.3
f-50-700_T2100	700	2100	1874	0.9562	143.7	115.1	27.1
f-50-700_T2500	700	2500	1894	0.9619	2213	1916	28.9
f-50-700_T2700	700	2700	1893	0.9621	4303	3713	28.5
f-50-700_T2900	700	2900	1894	0.9644	8040	7131	27.6
f-50-900_T2100	900	2100	1876	0.9632	164.8	133.9	23.0
f-50-900_T2500	900	2500	1884	0.9600	1940	1651	31.2
f-50-900_T2900	900	2900	1893	0.9640	7798	6850	28.8
**Repeat: strat42 Case: B Model: physical_MLR**							
f-50-1_T1700	1	1700	357	0.0000	21,566	17,398	1,739,781
f-50-1_T1900	1	1900	1318	0.0000	14,648	12,497	1,249,742
f-50-1_T2100	1	2100	1816	-1730.0	13,559	11,585	147,699
f-50-1_T2500	1	2500	1892	-1.6175	14,153	12,619	434.6
f-50-1_T2700	1	2700	1893	0.7457	10,926	9932	318.7
f-50-1_T2900	1	2900	1892	0.4111	31,043	23,659	162.3
f-50-100_T1500	100	1500	175	0.0000	29,543	25,311	2,531,088
f-50-100_T1700	100	1700	663	0.0000	17,427	14,085	1,408,508
f-50-100_T1900	100	1900	1458	-1.25×10^6	14,831	12,488	993,083
f-50-100_T2100	100	2100	1827	-156.6	12,860	11,070	29,396
f-50-100_T2500	100	2500	1891	0.2824	9083	8318	340.0
f-50-100_T2700	100	2700	1893	0.7723	10,396	9517	247.5
f-50-100_T2900	100	2900	1893	0.3538	35,125	26,178	161.2
f-50-300_T1500	300	1500	408	0.0000	29,146	25,738	2,573,827
f-50-300_T1700	300	1700	1215	0.0000	19,579	15,437	1,543,682
f-50-300_T1900	300	1900	1562	-4.97×10^8	14,920	12,323	1,208,185
f-50-300_T2100	300	2100	1869	-967.4	15,204	13,050	282,629
f-50-300_T2300	300	2300	1889	-20.2023	15,174	12,765	4177
f-50-300_T2500	300	2500	1894	0.0293	11,105	10,113	2030
f-50-300_T2700	300	2700	1893	0.7839	12,295	10,435	281.8

continued on next page

Table S12 (continued)

Simulation	$n(O_2)$	T (K)	n_{rows}	R^2	RMSE (molecule·ps)	MAE (molecule·ps)	MAPE (%)
f-50-300_T2900	300	2900	1895	0.4108	35,114	26,585	183.5
f-50-500_T1500	500	1500	434	0.0000	28,569	24,748	2,474,841
f-50-500_T2100	500	2100	1886	-462.2	14,679	12,705	314,182
f-50-500_T2300	500	2300	1891	-12.6143	14,715	12,531	7344
f-50-500_T2500	500	2500	1892	-0.5766	12,792	11,476	318.1
f-50-500_T2700	500	2700	1895	0.7506	12,579	11,032	436.9
f-50-500_T2900	500	2900	1893	0.4780	31,245	24,115	245.7
f-50-700_T1700	700	1700	1154	0.0000	19,515	15,101	1,510,135
f-50-700_T1900	700	1900	1812	-11,473.6	17,197	14,181	663,354
f-50-700_T2100	700	2100	1874	-451.6	14,604	12,611	241,556
f-50-700_T2500	700	2500	1894	0.3009	9475	8582	421.6
f-50-700_T2700	700	2700	1893	0.7727	10,542	9654	333.5
f-50-700_T2900	700	2900	1894	0.5215	29,463	22,958	287.8
f-50-900_T2100	900	2100	1876	-277.8	14,344	12,334	517,333
f-50-900_T2500	900	2500	1884	-0.9793	13,649	12,712	674.5
f-50-900_T2900	900	2900	1893	0.5462	27,694	21,848	433.4
**Repeat: strat42**	**Case: B**	**Model: physical_RF**					
f-50-1_T1700	1	1700	357	0.0000	43.2	18.4	1838
f-50-1_T1900	1	1900	1318	0.0000	353.1	248.4	24,844
f-50-1_T2100	1	2100	1816	-0.0541	334.6	254.9	79.8
f-50-1_T2500	1	2500	1892	0.4551	6457	5092	239.7
f-50-1_T2700	1	2700	1893	0.8463	8495	5734	72.6
f-50-1_T2900	1	2900	1892	0.7757	19,158	15,709	46.8
f-50-100_T1500	100	1500	175	0.0000	94.0	51.0	5101
f-50-100_T1700	100	1700	663	0.0000	67.5	30.0	3002
f-50-100_T1900	100	1900	1458	-2212.1	624.8	424.0	29,933
f-50-100_T2100	100	2100	1827	-0.6605	1320	990.5	90.1
f-50-100_T2500	100	2500	1891	0.9636	2046	1726	340.9
f-50-100_T2700	100	2700	1893	0.9452	5100	3344	15.1
f-50-100_T2900	100	2900	1893	0.7085	23,591	18,962	38.5
f-50-300_T1500	300	1500	408	0.0000	15.7	6.2	615.8
f-50-300_T1700	300	1700	1215	0.0000	102.4	64.9	6489
f-50-300_T1900	300	1900	1562	-577,119.0	508.6	407.1	39,438
f-50-300_T2100	300	2100	1869	-7.4054	1416	966.7	2735
f-50-300_T2300	300	2300	1889	0.0740	3171	2303	219.8
f-50-300_T2500	300	2500	1894	0.9346	2882	1939	452.8

continued on next page

Table S12 (continued)

Simulation	$n(O_2)$	T (K)	n_{rows}	R^2	RMSE (molecule·ps)	MAE (molecule·ps)	MAPE (%)
f-50-300_T2700	300	2700	1893	0.8217	11,168	8796	31.5
f-50-300_T2900	300	2900	1895	0.8869	15,387	12,014	21.6
f-50-500_T1500	500	1500	434	0.0000	3058	2341	234,073
f-50-500_T2100	500	2100	1886	-10.7203	2335	1784	1784
f-50-500_T2300	500	2300	1891	0.4680	2909	2079	73.0
f-50-500_T2500	500	2500	1892	0.8462	3996	2968	41.2
f-50-500_T2700	500	2700	1895	0.9303	6648	5344	106.1
f-50-500_T2900	500	2900	1893	0.9429	10,331	7851	25.3
f-50-700_T1700	700	1700	1154	0.0000	1084	868.0	86,803
f-50-700_T1900	700	1900	1812	-19.3893	724.9	515.3	726.8
f-50-700_T2100	700	2100	1874	0.7071	371.5	269.6	92.2
f-50-700_T2500	700	2500	1894	0.9760	1754	1342	167.0
f-50-700_T2700	700	2700	1893	0.9759	3435	2146	29.0
f-50-700_T2900	700	2900	1894	0.9233	11,800	8938	20.4
f-50-900_T2100	900	2100	1876	-72.9389	7386	5618	16,043
f-50-900_T2500	900	2500	1884	-0.5641	12,133	8483	244.3
f-50-900_T2900	900	2900	1893	0.6956	22,683	17,207	42.0
**Repeat: strat43 Case: A Model: MLR**							
f-50-1_T1700	1	1700	357	0.0000	607.7	505.4	50,543
f-50-1_T1900	1	1900	1318	0.0000	93.9	78.5	7850
f-50-1_T2100	1	2100	1816	-0.5141	401.0	383.7	4202
f-50-1_T2500	1	2500	1892	0.9961	546.2	490.3	130.7
f-50-1_T2700	1	2700	1893	0.9963	1317	907.1	10.8
f-50-1_T2900	1	2900	1892	0.9984	1605	1443	14.0
f-50-100_T1500	100	1500	175	0.0000	550.6	466.8	46,681
f-50-100_T1700	100	1700	663	0.0000	457.1	454.4	45,444
f-50-100_T1900	100	1900	1458	-1331.2	484.7	407.2	32,290
f-50-100_T2100	100	2100	1827	0.9122	303.5	252.9	625.2
f-50-100_T2500	100	2500	1891	0.9980	476.0	387.5	111.1
f-50-100_T2700	100	2700	1893	0.9994	532.0	447.1	26.9
f-50-100_T2900	100	2900	1893	0.9989	1417	1291	11.8
f-50-300_T1500	300	1500	408	0.0000	193.8	160.0	16,004
f-50-300_T1700	300	1700	1215	0.0000	542.7	535.9	53,590
f-50-300_T1900	300	1900	1562	-19,133.3	92.6	75.1	7462
f-50-300_T2100	300	2100	1869	0.3536	392.8	359.6	10,201
f-50-300_T2300	300	2300	1889	0.9549	699.6	690.3	307.9

continued on next page

Table S12 (continued)

Simulation	$n(O_2)$	T (K)	n_{rows}	R^2	RMSE (molecule·ps)	MAE (molecule·ps)	MAPE (%)
f-50-300_T2500	300	2500	1894	0.9985	431.2	326.0	596.8
f-50-300_T2700	300	2700	1893	0.9995	581.8	454.8	48.8
f-50-300_T2900	300	2900	1895	0.9993	1251	1108	13.0
f-50-500_T1500	500	1500	434	0.0000	1298	1296	129,629
f-50-500_T2100	500	2100	1886	0.7983	306.3	286.5	4116
f-50-500_T2300	500	2300	1891	0.9714	674.8	660.5	305.0
f-50-500_T2500	500	2500	1892	0.9946	747.6	731.5	79.2
f-50-500_T2700	500	2700	1895	0.9994	595.0	480.1	65.1
f-50-500_T2900	500	2900	1893	0.9983	1766	1534	16.2
f-50-700_T1700	700	1700	1154	0.0000	649.6	627.5	62,752
f-50-700_T1900	700	1900	1812	-17.0981	682.9	591.2	24,123
f-50-700_T2100	700	2100	1874	0.9260	186.8	145.6	952.9
f-50-700_T2500	700	2500	1894	0.9965	665.8	636.5	80.7
f-50-700_T2700	700	2700	1893	0.9996	468.4	398.8	8.8
f-50-700_T2900	700	2900	1894	0.9989	1417	1169	5.8
f-50-900_T2100	900	2100	1876	0.1247	803.7	727.2	31,843
f-50-900_T2500	900	2500	1884	0.9991	283.7	224.2	174.9
f-50-900_T2900	900	2900	1893	0.9982	1745	1515	32.3
**Repeat: strat43**	**Case: A**	**Model: DT**					
f-50-1_T1700	1	1700	357	0.0000	6.2	1.6	157.6
f-50-1_T1900	1	1900	1318	0.0000	3.3	1.8	177.7
f-50-1_T2100	1	2100	1816	0.9247	89.4	67.0	110.0
f-50-1_T2500	1	2500	1892	0.9982	371.3	283.3	7.3
f-50-1_T2700	1	2700	1893	0.9998	330.1	258.0	5.6
f-50-1_T2900	1	2900	1892	0.9996	808.5	631.6	3.0
f-50-100_T1500	100	1500	175	0.0000	1.1	0.9	91.4
f-50-100_T1700	100	1700	663	0.0000	5.0	2.0	204.5
f-50-100_T1900	100	1900	1458	0.1346	12.4	5.0	100.8
f-50-100_T2100	100	2100	1827	0.9825	135.3	97.0	55.6
f-50-100_T2500	100	2500	1891	0.9993	288.4	220.6	13.2
f-50-100_T2700	100	2700	1893	0.9997	407.4	305.9	4.5
f-50-100_T2900	100	2900	1893	0.9998	609.2	447.0	2.8
f-50-300_T1500	300	1500	408	0.0000	1.9	1.9	187.1
f-50-300_T1700	300	1700	1215	0.0000	5.6	3.5	347.4
f-50-300_T1900	300	1900	1562	-101.2	6.8	3.6	353.0
f-50-300_T2100	300	2100	1869	0.9719	81.9	56.8	59.3

continued on next page

Table S12 (continued)

Simulation	$n(O_2)$	T (K)	n_{rows}	R^2	RMSE (molecule·ps)	MAE (molecule·ps)	MAPE (%)
f-50-300_T2300	300	2300	1889	0.9943	248.9	206.1	20.2
f-50-300_T2500	300	2500	1894	0.9996	214.8	150.2	29.1
f-50-300_T2700	300	2700	1893	0.9996	502.3	346.8	6.4
f-50-300_T2900	300	2900	1895	0.9997	801.7	446.8	3.7
f-50-500_T1500	500	1500	434	0.0000	3.2	3.2	322.4
f-50-500_T2100	500	2100	1886	0.9930	57.3	43.4	131.9
f-50-500_T2300	500	2300	1891	0.9926	343.6	266.3	43.6
f-50-500_T2500	500	2500	1892	0.9996	195.1	155.7	8.3
f-50-500_T2700	500	2700	1895	0.9997	459.9	321.3	9.6
f-50-500_T2900	500	2900	1893	0.9999	364.0	300.7	3.8
f-50-700_T1700	700	1700	1154	0.0000	1.9	1.9	187.1
f-50-700_T1900	700	1900	1812	0.9075	48.8	36.6	67.2
f-50-700_T2100	700	2100	1874	0.9949	49.2	38.7	127.6
f-50-700_T2500	700	2500	1894	0.9996	233.5	153.1	7.7
f-50-700_T2700	700	2700	1893	0.9996	463.2	349.6	6.1
f-50-700_T2900	700	2900	1894	1.0000	269.9	218.2	4.1
f-50-900_T2100	900	2100	1876	0.9595	172.9	98.0	824.0
f-50-900_T2500	900	2500	1884	0.9993	253.1	172.0	28.8
f-50-900_T2900	900	2900	1893	0.9999	327.4	237.7	6.4
**Repeat: strat43 Case: A Model: RF**							
f-50-1_T1700	1	1700	357	0.0000	11.5	2.7	271.3
f-50-1_T1900	1	1900	1318	0.0000	9.2	6.1	606.8
f-50-1_T2100	1	2100	1816	0.9429	77.9	59.1	99.6
f-50-1_T2500	1	2500	1892	0.9984	351.5	272.7	8.1
f-50-1_T2700	1	2700	1893	0.9998	334.4	263.2	3.9
f-50-1_T2900	1	2900	1892	0.9996	808.2	637.0	2.4
f-50-100_T1500	100	1500	175	0.0000	2.0	1.5	145.8
f-50-100_T1700	100	1700	663	0.0000	4.1	2.7	265.4
f-50-100_T1900	100	1900	1458	0.0085	13.2	5.6	141.3
f-50-100_T2100	100	2100	1827	0.9834	131.9	97.3	56.1
f-50-100_T2500	100	2500	1891	0.9997	172.6	122.4	9.9
f-50-100_T2700	100	2700	1893	0.9999	229.3	187.1	4.0
f-50-100_T2900	100	2900	1893	0.9999	453.7	355.8	2.6
f-50-300_T1500	300	1500	408	0.0000	1.7	1.7	171.8
f-50-300_T1700	300	1700	1215	0.0000	4.9	3.0	301.5
f-50-300_T1900	300	1900	1562	-3710.7	40.8	23.9	2267

continued on next page

Table S12 (continued)

Simulation	$n(O_2)$	T (K)	n_{rows}	R^2	RMSE (molecule·ps)	MAE (molecule·ps)	MAPE (%)
f-50-300_T2100	300	2100	1869	0.9796	69.8	51.1	71.8
f-50-300_T2300	300	2300	1889	0.9952	227.7	206.2	19.5
f-50-300_T2500	300	2500	1894	0.9998	166.6	139.1	33.7
f-50-300_T2700	300	2700	1893	0.9998	416.7	288.4	5.5
f-50-300_T2900	300	2900	1895	0.9997	813.7	444.1	3.5
f-50-500_T1500	500	1500	434	0.0000	2.8	2.8	280.4
f-50-500_T2100	500	2100	1886	0.9935	54.9	43.3	127.5
f-50-500_T2300	500	2300	1891	0.9947	290.6	240.9	39.3
f-50-500_T2500	500	2500	1892	0.9997	161.8	140.7	6.8
f-50-500_T2700	500	2700	1895	0.9998	316.4	262.9	8.7
f-50-500_T2900	500	2900	1893	1.0000	266.5	239.3	3.0
f-50-700_T1700	700	1700	1154	0.0000	1.7	1.7	171.4
f-50-700_T1900	700	1900	1812	0.9124	47.5	35.5	67.2
f-50-700_T2100	700	2100	1874	0.9975	34.2	26.7	83.5
f-50-700_T2500	700	2500	1894	0.9999	133.6	88.4	5.7
f-50-700_T2700	700	2700	1893	0.9998	331.0	279.0	5.2
f-50-700_T2900	700	2900	1894	1.0000	211.6	166.4	3.5
f-50-900_T2100	900	2100	1876	0.9762	132.6	81.3	699.7
f-50-900_T2500	900	2500	1884	0.9999	110.8	81.8	21.8
f-50-900_T2900	900	2900	1893	1.0000	269.8	208.8	5.7
**Repeat: strat43**	**Case: A**	**Model: XGBoost**					
f-50-1_T1700	1	1700	357	0.0000	11.3	2.4	240.1
f-50-1_T1900	1	1900	1318	0.0000	7.3	3.5	352.1
f-50-1_T2100	1	2100	1816	0.7128	174.7	127.4	125.6
f-50-1_T2500	1	2500	1892	0.9985	339.4	296.4	14.3
f-50-1_T2700	1	2700	1893	0.9998	303.2	234.3	3.9
f-50-1_T2900	1	2900	1892	0.9996	797.9	530.3	2.3
f-50-100_T1500	100	1500	175	0.0000	4.1	1.3	130.7
f-50-100_T1700	100	1700	663	0.0000	1.5	1.0	103.9
f-50-100_T1900	100	1900	1458	0.6366	8.0	4.2	199.7
f-50-100_T2100	100	2100	1827	0.9873	115.3	81.8	72.8
f-50-100_T2500	100	2500	1891	0.9996	213.9	171.0	10.3
f-50-100_T2700	100	2700	1893	0.9995	466.2	351.8	4.0
f-50-100_T2900	100	2900	1893	0.9995	987.9	617.3	2.6
f-50-300_T1500	300	1500	408	0.0000	0.7	0.4	41.2
f-50-300_T1700	300	1700	1215	0.0000	2.7	2.0	197.4

continued on next page

Table S12 (continued)

Simulation	$n(O_2)$	T (K)	n_{rows}	R^2	RMSE (molecule·ps)	MAE (molecule·ps)	MAPE (%)
f-50-300_T1900	300	1900	1562	-2081.1	30.5	18.6	1780
f-50-300_T2100	300	2100	1869	0.9442	115.4	82.0	77.3
f-50-300_T2300	300	2300	1889	0.9942	250.7	216.3	13.5
f-50-300_T2500	300	2500	1894	0.9998	145.9	108.5	48.8
f-50-300_T2700	300	2700	1893	0.9996	507.7	387.3	6.6
f-50-300_T2900	300	2900	1895	0.9986	1737	966.7	3.5
f-50-500_T1500	500	1500	434	0.0000	1.6	1.4	143.2
f-50-500_T2100	500	2100	1886	0.9944	50.9	38.9	135.3
f-50-500_T2300	500	2300	1891	0.9981	172.6	117.2	26.0
f-50-500_T2500	500	2500	1892	0.9997	187.8	157.4	6.0
f-50-500_T2700	500	2700	1895	0.9997	412.3	290.7	8.7
f-50-500_T2900	500	2900	1893	0.9996	873.4	512.4	3.0
f-50-700_T1700	700	1700	1154	0.0000	5.7	4.7	469.3
f-50-700_T1900	700	1900	1812	0.9460	37.3	26.2	31.3
f-50-700_T2100	700	2100	1874	0.9978	32.3	23.4	69.4
f-50-700_T2500	700	2500	1894	0.9998	167.7	127.4	4.9
f-50-700_T2700	700	2700	1893	0.9995	486.3	380.9	5.2
f-50-700_T2900	700	2900	1894	0.9998	663.0	413.0	2.9
f-50-900_T2100	900	2100	1876	0.9651	160.4	136.9	564.5
f-50-900_T2500	900	2500	1884	0.9996	184.6	150.3	22.9
f-50-900_T2900	900	2900	1893	0.9993	1091	869.1	6.2
**Repeat: strat43 Case: A Model: CatBoost**							
f-50-1_T1700	1	1700	357	0.0000	30.9	22.3	2229
f-50-1_T1900	1	1900	1318	0.0000	51.7	44.6	4461
f-50-1_T2100	1	2100	1816	0.7589	160.0	123.8	503.5
f-50-1_T2500	1	2500	1892	0.9964	527.1	411.2	12.5
f-50-1_T2700	1	2700	1893	0.9993	572.7	456.1	5.2
f-50-1_T2900	1	2900	1892	0.9995	889.9	618.6	3.2
f-50-100_T1500	100	1500	175	0.0000	20.3	15.0	1502
f-50-100_T1700	100	1700	663	0.0000	52.4	47.6	4757
f-50-100_T1900	100	1900	1458	-2.6744	25.5	21.1	1640
f-50-100_T2100	100	2100	1827	0.8767	359.7	295.4	62.8
f-50-100_T2500	100	2500	1891	0.9993	280.2	205.5	7.2
f-50-100_T2700	100	2700	1893	0.9995	476.8	371.7	5.0
f-50-100_T2900	100	2900	1893	0.9993	1149	820.6	4.8
f-50-300_T1500	300	1500	408	0.0000	44.9	40.4	4043

continued on next page

Table S12 (continued)

Simulation	$n(O_2)$	T (K)	n_{rows}	R^2	RMSE (molecule·ps)	MAE (molecule·ps)	MAPE (%)
f-50-300_T1700	300	1700	1215	0.0000	51.4	35.0	3499
f-50-300_T1900	300	1900	1562	-29,369.1	114.7	96.4	9374
f-50-300_T2100	300	2100	1869	0.9699	84.8	70.1	216.5
f-50-300_T2300	300	2300	1889	0.9888	349.2	278.6	23.9
f-50-300_T2500	300	2500	1894	0.9993	298.6	227.9	46.7
f-50-300_T2700	300	2700	1893	0.9986	978.6	527.3	7.2
f-50-300_T2900	300	2900	1895	0.9986	1718	1096	4.6
f-50-500_T1500	500	1500	434	0.0000	23.9	17.3	1731
f-50-500_T2100	500	2100	1886	0.9479	155.7	125.3	691.5
f-50-500_T2300	500	2300	1891	0.9960	250.9	194.6	77.8
f-50-500_T2500	500	2500	1892	0.9989	335.7	261.1	8.3
f-50-500_T2700	500	2700	1895	0.9995	573.4	376.9	11.2
f-50-500_T2900	500	2900	1893	0.9996	879.3	680.4	4.7
f-50-700_T1700	700	1700	1154	0.0000	101.9	83.0	8296
f-50-700_T1900	700	1900	1812	0.7571	79.1	47.2	635.2
f-50-700_T2100	700	2100	1874	0.9697	119.5	93.8	486.6
f-50-700_T2500	700	2500	1894	0.9988	390.6	277.8	17.5
f-50-700_T2700	700	2700	1893	0.9993	566.9	405.7	5.5
f-50-700_T2900	700	2900	1894	0.9996	878.2	695.5	6.0
f-50-900_T2100	900	2100	1876	0.9316	224.6	184.1	7090
f-50-900_T2500	900	2500	1884	0.9980	428.5	316.7	48.4
f-50-900_T2900	900	2900	1893	0.9811	5647	4203	10.4
**Repeat: strat43**	**Case: A**	**Model: ANN**					
f-50-1_T1700	1	1700	357	0.0000	81.3	61.7	6166
f-50-1_T1900	1	1900	1318	0.0000	114.5	91.6	9161
f-50-1_T2100	1	2100	1816	-4.3404	753.1	420.3	1253
f-50-1_T2500	1	2500	1892	0.9942	663.4	601.4	55.6
f-50-1_T2700	1	2700	1893	0.9998	300.2	225.9	3.5
f-50-1_T2900	1	2900	1892	0.9995	858.9	660.7	10.5
f-50-100_T1500	100	1500	175	0.0000	37.5	24.8	2484
f-50-100_T1700	100	1700	663	0.0000	59.3	54.5	5450
f-50-100_T1900	100	1900	1458	-6.1756	35.6	23.6	1362
f-50-100_T2100	100	2100	1827	0.6948	565.9	445.6	144.9
f-50-100_T2500	100	2500	1891	0.9922	946.3	748.9	16.5
f-50-100_T2700	100	2700	1893	0.9988	769.2	564.1	6.2
f-50-100_T2900	100	2900	1893	0.9997	693.0	552.5	4.8

continued on next page

Table S12 (continued)

Simulation	$n(O_2)$	T (K)	n_{rows}	R^2	RMSE (molecule·ps)	MAE (molecule·ps)	MAPE (%)
f-50-300_T1500	300	1500	408	0.0000	193.5	181.8	18,179
f-50-300_T1700	300	1700	1215	0.0000	100.0	81.4	8141
f-50-300_T1900	300	1900	1562	-5010.7	47.4	42.1	4133
f-50-300_T2100	300	2100	1869	0.0846	467.4	315.1	382.1
f-50-300_T2300	300	2300	1889	0.8171	1409	1101	48.9
f-50-300_T2500	300	2500	1894	0.9992	327.1	235.7	54.3
f-50-300_T2700	300	2700	1893	0.9995	612.5	464.4	5.1
f-50-300_T2900	300	2900	1895	0.9998	629.7	500.9	4.1
f-50-500_T1500	500	1500	434	0.0000	60.0	44.1	4407
f-50-500_T2100	500	2100	1886	0.8951	220.9	174.4	203.6
f-50-500_T2300	500	2300	1891	0.9891	416.8	302.3	36.5
f-50-500_T2500	500	2500	1892	0.9982	436.4	334.8	8.0
f-50-500_T2700	500	2700	1895	0.9996	533.1	400.2	11.3
f-50-500_T2900	500	2900	1893	0.9998	657.5	506.8	6.0
f-50-700_T1700	700	1700	1154	0.0000	348.5	295.1	29,515
f-50-700_T1900	700	1900	1812	-2.4581	298.5	241.3	11,831
f-50-700_T2100	700	2100	1874	0.8372	277.0	190.6	963.8
f-50-700_T2500	700	2500	1894	0.9966	663.7	535.0	15.2
f-50-700_T2700	700	2700	1893	0.9995	477.1	377.1	6.8
f-50-700_T2900	700	2900	1894	0.9998	632.4	556.3	5.1
f-50-900_T2100	900	2100	1876	0.7658	415.7	361.2	14,135
f-50-900_T2500	900	2500	1884	0.9913	905.4	722.3	53.2
f-50-900_T2900	900	2900	1893	0.9981	1793	1470	9.4
**Repeat: strat43 Case: A Model: SVR**							
f-50-1_T1700	1	1700	357	0.0000	1155	998.7	99,873
f-50-1_T1900	1	1900	1318	0.0000	1163	1100	110,009
f-50-1_T2100	1	2100	1816	-21.0350	1530	1087	6960
f-50-1_T2500	1	2500	1892	0.8900	2901	2372	162.3
f-50-1_T2700	1	2700	1893	0.7068	11,732	6904	53.2
f-50-1_T2900	1	2900	1892	0.1706	36,842	24,596	46.1
f-50-100_T1500	100	1500	175	0.0000	647.9	431.0	43,096
f-50-100_T1700	100	1700	663	0.0000	623.5	495.2	49,516
f-50-100_T1900	100	1900	1458	-1610.7	533.2	372.1	21,345
f-50-100_T2100	100	2100	1827	-0.4997	1254	962.7	762.7
f-50-100_T2500	100	2500	1891	0.9866	1241	801.6	84.7
f-50-100_T2700	100	2700	1893	0.8890	7258	4161	33.7

continued on next page

Table S12 (continued)

Simulation	$n(O_2)$	T (K)	n_{rows}	R^2	RMSE (molecule·ps)	MAE (molecule·ps)	MAPE (%)
f-50-100_T2900	100	2900	1893	0.1306	40,744	25,977	43.0
f-50-300_T1500	300	1500	408	0.0000	1158	1076	107,607
f-50-300_T1700	300	1700	1215	0.0000	415.6	336.9	33,694
f-50-300_T1900	300	1900	1562	-474,068.5	461.0	309.3	29,190
f-50-300_T2100	300	2100	1869	-15.5900	1990	1399	5871
f-50-300_T2300	300	2300	1889	0.7417	1675	1443	269.6
f-50-300_T2500	300	2500	1894	0.9809	1557	1206	374.9
f-50-300_T2700	300	2700	1893	0.8102	11,521	6842	70.1
f-50-300_T2900	300	2900	1895	0.2044	40,803	25,804	45.5
f-50-500_T1500	500	1500	434	0.0000	402.0	362.4	36,243
f-50-500_T2100	500	2100	1886	-2.1299	1207	861.3	4133
f-50-500_T2300	500	2300	1891	0.7575	1964	1625	428.6
f-50-500_T2500	500	2500	1892	0.9499	2280	1399	68.3
f-50-500_T2700	500	2700	1895	0.8056	11,106	6893	85.8
f-50-500_T2900	500	2900	1893	0.1997	38,687	26,555	56.6
f-50-700_T1700	700	1700	1154	0.0000	3926	3676	367,591
f-50-700_T1900	700	1900	1812	-264.4	2615	2361	28,769
f-50-700_T2100	700	2100	1874	-2.3348	1254	880.6	4457
f-50-700_T2500	700	2500	1894	0.9144	3315	1946	85.9
f-50-700_T2700	700	2700	1893	0.6962	12,188	7616	38.4
f-50-700_T2900	700	2900	1894	0.1473	39,332	26,531	40.2
f-50-900_T2100	900	2100	1876	-25.5019	4422	3719	41,575
f-50-900_T2500	900	2500	1884	0.8277	4027	2884	633.3
f-50-900_T2900	900	2900	1893	-0.1023	43,164	28,712	134.2
**Repeat: strat43**	**Case: A**	**Model: GPR**					
f-50-1_T1700	1	1700	357	0.0000	21.8	14.4	1441
f-50-1_T1900	1	1900	1318	0.0000	8.1	7.1	712.1
f-50-1_T2100	1	2100	1816	0.9498	73.0	61.8	110.7
f-50-1_T2500	1	2500	1892	0.9995	198.5	149.7	16.0
f-50-1_T2700	1	2700	1893	0.9999	177.4	143.9	3.1
f-50-1_T2900	1	2900	1892	0.9999	400.8	363.0	2.6
f-50-100_T1500	100	1500	175	0.0000	16.0	8.4	844.4
f-50-100_T1700	100	1700	663	0.0000	10.1	9.6	959.6
f-50-100_T1900	100	1900	1458	-1.5348	21.1	17.3	1248
f-50-100_T2100	100	2100	1827	0.9878	113.2	95.5	28.2
f-50-100_T2500	100	2500	1891	0.9999	92.2	80.4	4.9

continued on next page

Table S12 (continued)

Simulation	$n(O_2)$	T (K)	n_{rows}	R^2	RMSE (molecule·ps)	MAE (molecule·ps)	MAPE (%)
f-50-100_T2700	100	2700	1893	0.9999	171.6	130.2	2.9
f-50-100_T2900	100	2900	1893	1.0000	281.6	250.7	2.1
f-50-300_T1500	300	1500	408	0.0000	12.5	11.4	1142
f-50-300_T1700	300	1700	1215	0.0000	14.7	12.3	1232
f-50-300_T1900	300	1900	1562	-3461.5	39.4	33.5	3337
f-50-300_T2100	300	2100	1869	0.9765	74.9	61.8	296.0
f-50-300_T2300	300	2300	1889	0.9987	118.6	80.5	8.9
f-50-300_T2500	300	2500	1894	0.9998	144.1	121.0	38.6
f-50-300_T2700	300	2700	1893	0.9999	250.0	211.8	5.9
f-50-300_T2900	300	2900	1895	1.0000	193.4	140.7	2.0
f-50-500_T1500	500	1500	434	0.0000	9.5	6.9	690.8
f-50-500_T2100	500	2100	1886	0.9849	83.8	61.8	199.9
f-50-500_T2300	500	2300	1891	0.9946	292.8	204.9	46.2
f-50-500_T2500	500	2500	1892	0.9999	117.9	86.7	6.3
f-50-500_T2700	500	2700	1895	1.0000	165.5	143.2	5.8
f-50-500_T2900	500	2900	1893	0.9999	508.1	455.4	3.7
f-50-700_T1700	700	1700	1154	0.0000	292.0	230.9	23,087
f-50-700_T1900	700	1900	1812	0.1715	146.1	112.4	4924
f-50-700_T2100	700	2100	1874	0.9930	57.6	48.1	91.3
f-50-700_T2500	700	2500	1894	0.9998	173.8	142.5	6.7
f-50-700_T2700	700	2700	1893	0.9999	252.5	188.5	4.6
f-50-700_T2900	700	2900	1894	1.0000	213.6	181.4	3.3
f-50-900_T2100	900	2100	1876	0.6441	512.4	421.0	20,705
f-50-900_T2500	900	2500	1884	0.9996	202.3	143.9	17.8
f-50-900_T2900	900	2900	1893	0.9994	1036	897.9	5.8
**Repeat: strat43 Case: A Model: persistence**							
f-50-1_T1700	1	1700	357	1.0000	0.0	0.0	0.0
f-50-1_T1900	1	1900	1318	1.0000	0.0	0.0	0.0
f-50-1_T2100	1	2100	1816	0.9631	62.6	54.9	23.7
f-50-1_T2500	1	2500	1892	0.9646	1647	1468	28.5
f-50-1_T2700	1	2700	1893	0.9644	4090	3642	27.3
f-50-1_T2900	1	2900	1892	0.9666	7390	6812	25.5
f-50-100_T1500	100	1500	175	1.0000	0.0	0.0	0.0
f-50-100_T1700	100	1700	663	1.0000	0.0	0.0	0.0
f-50-100_T1900	100	1900	1458	0.5926	8.5	3.1	11.9
f-50-100_T2100	100	2100	1827	0.9585	208.7	176.9	28.0

continued on next page

Table S12 (continued)

Simulation	$n(O_2)$	T (K)	n_{rows}	R^2	RMSE (molecule·ps)	MAE (molecule·ps)	MAPE (%)
f-50-100_T2500	100	2500	1891	0.9636	2046	1794	29.2
f-50-100_T2700	100	2700	1893	0.9633	4174	3673	26.9
f-50-100_T2900	100	2900	1893	0.9656	8105	7371	25.8
f-50-300_T1500	300	1500	408	1.0000	0.0	0.0	0.0
f-50-300_T1700	300	1700	1215	1.0000	0.0	0.0	0.0
f-50-300_T1900	300	1900	1562	0.0667	0.6	0.1	3.4
f-50-300_T2100	300	2100	1869	0.9635	93.4	80.0	24.3
f-50-300_T2300	300	2300	1889	0.9586	670.4	572.4	28.8
f-50-300_T2500	300	2500	1894	0.9609	2228	1892	31.4
f-50-300_T2700	300	2700	1893	0.9631	5080	4441	28.5
f-50-300_T2900	300	2900	1895	0.9655	8494	7665	26.3
f-50-500_T1500	500	1500	434	1.0000	0.0	0.0	0.0
f-50-500_T2100	500	2100	1886	0.9467	157.5	113.8	27.3
f-50-500_T2300	500	2300	1891	0.9589	808.1	663.3	31.3
f-50-500_T2500	500	2500	1892	0.9581	2085	1751	28.4
f-50-500_T2700	500	2700	1895	0.9622	4897	4216	29.2
f-50-500_T2900	500	2900	1893	0.9655	8037	7244	27.0
f-50-700_T1700	700	1700	1154	1.0000	0.0	0.0	0.0
f-50-700_T1900	700	1900	1812	0.9550	34.0	27.5	21.3
f-50-700_T2100	700	2100	1874	0.9562	143.7	115.1	27.1
f-50-700_T2500	700	2500	1894	0.9619	2213	1916	28.9
f-50-700_T2700	700	2700	1893	0.9621	4303	3713	28.5
f-50-700_T2900	700	2900	1894	0.9644	8040	7131	27.6
f-50-900_T2100	900	2100	1876	0.9632	164.8	133.9	23.0
f-50-900_T2500	900	2500	1884	0.9600	1940	1651	31.2
f-50-900_T2900	900	2900	1893	0.9640	7798	6850	28.8
**Repeat: strat43 Case: A Model: physical_MLR**							
f-50-1_T1700	1	1700	357	0.0000	20,821	16,876	1,687,579
f-50-1_T1900	1	1900	1318	0.0000	14,870	12,624	1,262,415
f-50-1_T2100	1	2100	1816	-1939.6	14,357	12,122	131,275
f-50-1_T2500	1	2500	1892	-1.9443	15,010	13,381	434.5
f-50-1_T2700	1	2700	1893	0.7457	10,926	9932	318.7
f-50-1_T2900	1	2900	1892	0.4685	29,491	23,085	191.9
f-50-100_T1500	100	1500	175	0.0000	28,730	24,485	2,448,501
f-50-100_T1700	100	1700	663	0.0000	17,889	14,406	1,440,605
f-50-100_T1900	100	1900	1458	-1.23×10^6	14,742	12,479	977,990

continued on next page

Table S12 (continued)

Simulation	$n(O_2)$	T (K)	n_{rows}	R^2	RMSE (molecule·ps)	MAE (molecule·ps)	MAPE (%)
f-50-100_T2100	100	2100	1827	-171.4	13,448	11,486	27,105
f-50-100_T2500	100	2500	1891	-0.0737	11,111	10,305	309.8
f-50-100_T2700	100	2700	1893	0.8021	9692	8868	216.7
f-50-100_T2900	100	2900	1893	0.4302	32,985	24,805	165.3
f-50-300_T1500	300	1500	408	0.0000	29,146	25,738	2,573,827
f-50-300_T1700	300	1700	1215	0.0000	19,241	15,171	1,517,095
f-50-300_T1900	300	1900	1562	-4.80×10^8	14,667	12,096	1,186,258
f-50-300_T2100	300	2100	1869	-982.9	15,325	13,138	278,504
f-50-300_T2300	300	2300	1889	-20.3681	15,233	12,821	4285
f-50-300_T2500	300	2500	1894	0.0293	11,105	10,113	2030
f-50-300_T2700	300	2700	1893	0.7793	12,424	10,417	272.0
f-50-300_T2900	300	2900	1895	0.4166	34,941	26,452	182.4
f-50-500_T1500	500	1500	434	0.0000	27,270	23,414	2,341,387
f-50-500_T2100	500	2100	1886	-447.2	14,438	12,483	299,089
f-50-500_T2300	500	2300	1891	-12.3838	14,590	12,438	7545
f-50-500_T2500	500	2500	1892	-0.4927	12,447	11,175	314.8
f-50-500_T2700	500	2700	1895	0.7528	12,524	11,032	440.5
f-50-500_T2900	500	2900	1893	0.4780	31,245	24,115	245.7
f-50-700_T1700	700	1700	1154	0.0000	19,515	15,101	1,510,135
f-50-700_T1900	700	1900	1812	-11,473.6	17,197	14,181	663,354
f-50-700_T2100	700	2100	1874	-467.5	14,859	12,802	236,696
f-50-700_T2500	700	2500	1894	0.2203	10,007	9101	340.4
f-50-700_T2700	700	2700	1893	0.7959	9990	9130	290.4
f-50-700_T2900	700	2900	1894	0.5122	29,749	22,929	270.8
f-50-900_T2100	900	2100	1876	-277.8	14,344	12,334	517,333
f-50-900_T2500	900	2500	1884	-0.4604	11,724	10,696	642.1
f-50-900_T2900	900	2900	1893	0.4937	29,253	22,991	462.7
**Repeat: strat43**	**Case: A**	**Model: physical_RF**					
f-50-1_T1700	1	1700	357	0.0000	0.0	0.0	0.1
f-50-1_T1900	1	1900	1318	0.0000	14.2	5.0	499.0
f-50-1_T2100	1	2100	1816	0.5046	229.4	166.8	45.2
f-50-1_T2500	1	2500	1892	0.8896	2907	1971	14.6
f-50-1_T2700	1	2700	1893	0.9954	1473	593.7	4.5
f-50-1_T2900	1	2900	1892	0.9873	4564	2837	4.1
f-50-100_T1500	100	1500	175	0.0000	0.0	0.0	0.4
f-50-100_T1700	100	1700	663	0.0000	1.1	0.3	32.6

continued on next page

Table S12 (continued)

Simulation	$n(O_2)$	T (K)	n_{rows}	R^2	RMSE (molecule·ps)	MAE (molecule·ps)	MAPE (%)
f-50-100_T1900	100	1900	1458	-0.1162	14.0	4.9	22.2
f-50-100_T2100	100	2100	1827	0.3857	802.9	594.9	58.8
f-50-100_T2500	100	2500	1891	0.9612	2112	1523	10.7
f-50-100_T2700	100	2700	1893	0.9969	1222	673.1	6.4
f-50-100_T2900	100	2900	1893	0.9901	4339	2713	4.8
f-50-300_T1500	300	1500	408	0.0000	0.0	0.0	1.5
f-50-300_T1700	300	1700	1215	0.0000	0.2	0.1	5.4
f-50-300_T1900	300	1900	1562	-408.7	13.6	4.8	414.6
f-50-300_T2100	300	2100	1869	-1.5981	787.5	575.7	481.3
f-50-300_T2300	300	2300	1889	0.9418	795.1	625.4	25.2
f-50-300_T2500	300	2500	1894	0.9943	848.5	534.1	38.4
f-50-300_T2700	300	2700	1893	0.9354	6721	4881	16.6
f-50-300_T2900	300	2900	1895	0.9947	3338	2474	5.6
f-50-500_T1500	500	1500	434	0.0000	0.3	0.1	6.2
f-50-500_T2100	500	2100	1886	0.8605	254.8	180.9	87.2
f-50-500_T2300	500	2300	1891	0.9604	793.9	629.9	45.9
f-50-500_T2500	500	2500	1892	0.9693	1785	1395	13.6
f-50-500_T2700	500	2700	1895	0.9861	2971	2364	13.5
f-50-500_T2900	500	2900	1893	0.9932	3560	2964	11.4
f-50-700_T1700	700	1700	1154	0.0000	0.0	0.0	2.4
f-50-700_T1900	700	1900	1812	-1.1424	235.0	171.8	80.3
f-50-700_T2100	700	2100	1874	0.9666	125.4	96.9	38.7
f-50-700_T2500	700	2500	1894	0.9622	2204	1805	15.8
f-50-700_T2700	700	2700	1893	0.9572	4575	3393	12.6
f-50-700_T2900	700	2900	1894	0.9945	3166	2721	11.5
f-50-900_T2100	900	2100	1876	0.8903	284.5	212.0	430.7
f-50-900_T2500	900	2500	1884	0.9172	2791	2262	46.0
f-50-900_T2900	900	2900	1893	0.9922	3622	3098	17.7
**Repeat: strat43 Case: B Model: MLR**							
f-50-1_T1700	1	1700	357	0.0000	19,841	18,266	1,826,566
f-50-1_T1900	1	1900	1318	0.0000	22,501	20,894	2,089,442
f-50-1_T2100	1	2100	1816	-1662.6	13,293	13,061	70,171
f-50-1_T2500	1	2500	1892	0.7349	4504	4132	694.8
f-50-1_T2700	1	2700	1893	-0.1342	23,076	20,873	417.2
f-50-1_T2900	1	2900	1892	0.5211	27,993	22,501	168.4
f-50-100_T1500	100	1500	175	0.0000	14,864	12,915	1,291,527

continued on next page

Table S12 (continued)

Simulation	$n(O_2)$	T (K)	n_{rows}	R^2	RMSE (molecule·ps)	MAE (molecule·ps)	MAPE (%)
f-50-100_T1700	100	1700	663	0.0000	17,945	16,122	1,612,179
f-50-100_T1900	100	1900	1458	-2.00×10^6	18,776	17,093	1,306,173
f-50-100_T2100	100	2100	1827	-126.8	11,582	11,093	7156
f-50-100_T2500	100	2500	1891	0.6858	6010	5595	452.4
f-50-100_T2700	100	2700	1893	0.8831	7448	5107	87.7
f-50-100_T2900	100	2900	1893	0.3758	34,525	23,743	67.3
f-50-300_T1500	300	1500	408	0.0000	14,338	13,208	1,320,770
f-50-300_T1700	300	1700	1215	0.0000	12,014	10,061	1,006,150
f-50-300_T1900	300	1900	1562	-3.24×10^8	12,058	9997	969,416
f-50-300_T2100	300	2100	1869	-1816.0	20,825	16,888	50,813
f-50-300_T2300	300	2300	1889	-37.0436	20,325	17,215	2654
f-50-300_T2500	300	2500	1894	0.4063	8684	7244	837.6
f-50-300_T2700	300	2700	1893	0.7099	14,243	11,612	288.0
f-50-300_T2900	300	2900	1895	0.3679	36,369	26,012	114.1
f-50-500_T1500	500	1500	434	0.0000	7887	6782	678,240
f-50-500_T2100	500	2100	1886	-985.9	21,425	17,976	205,004
f-50-500_T2300	500	2300	1891	-29.4350	22,002	19,669	8862
f-50-500_T2500	500	2500	1892	-2.4453	18,910	17,844	1226
f-50-500_T2700	500	2700	1895	0.5289	17,287	15,010	748.3
f-50-500_T2900	500	2900	1893	-0.0498	44,310	31,861	196.7
f-50-700_T1700	700	1700	1154	0.0000	17,216	16,326	1,632,605
f-50-700_T1900	700	1900	1812	-5400.7	11,799	10,874	124,844
f-50-700_T2100	700	2100	1874	-972.9	21,422	18,868	282,456
f-50-700_T2500	700	2500	1894	-0.9924	15,996	14,946	2005
f-50-700_T2700	700	2700	1893	0.4317	16,671	14,074	903.0
f-50-700_T2900	700	2900	1894	-0.1599	45,875	34,371	356.8
f-50-900_T2100	900	2100	1876	-329.8	15,625	11,947	83,327
f-50-900_T2500	900	2500	1884	-0.7623	12,879	9923	7548
f-50-900_T2900	900	2900	1893	-1.3637	63,205	48,222	743.6
**Repeat: strat43**	**Case: B**	**Model: DT**					
f-50-1_T1700	1	1700	357	1.0000	0.0	0.0	0.0
f-50-1_T1900	1	1900	1318	0.0000	0.6	0.0	3.2
f-50-1_T2100	1	2100	1816	-8394.0	29,860	17,786	2690
f-50-1_T2500	1	2500	1892	-0.0501	8964	7065	82.3
f-50-1_T2700	1	2700	1893	0.0185	21,466	16,936	59.8
f-50-1_T2900	1	2900	1892	0.4575	29,796	22,538	47.4

continued on next page

Table S12 (continued)

Simulation	$n(O_2)$	T (K)	n_{rows}	R^2	RMSE (molecule·ps)	MAE (molecule·ps)	MAPE (%)
f-50-100_T1500	100	1500	175	0.0000	11.3	0.9	85.7
f-50-100_T1700	100	1700	663	1.0000	0.0	0.0	0.0
f-50-100_T1900	100	1900	1458	-34.6753	79.3	21.7	51.3
f-50-100_T2100	100	2100	1827	-108.9	10,736	4054	224.5
f-50-100_T2500	100	2500	1891	0.6042	6746	5271	77.4
f-50-100_T2700	100	2700	1893	0.4416	16,279	11,294	37.1
f-50-100_T2900	100	2900	1893	0.5992	27,664	19,793	31.8
f-50-300_T1500	300	1500	408	1.0000	0.0	0.0	0.0
f-50-300_T1700	300	1700	1215	0.0000	552.2	158.9	15,888
f-50-300_T1900	300	1900	1562	-3.09×10^6	1178	759.3	73,446
f-50-300_T2100	300	2100	1869	-1340.8	17,896	5667	1073
f-50-300_T2300	300	2300	1889	-0.5822	4145	2047	49.0
f-50-300_T2500	300	2500	1894	0.0361	11,066	4442	93.0
f-50-300_T2700	300	2700	1893	0.1836	23,895	16,245	46.0
f-50-300_T2900	300	2900	1895	0.5255	31,512	23,621	38.6
f-50-500_T1500	500	1500	434	1.0000	0.0	0.0	0.0
f-50-500_T2100	500	2100	1886	-32.8988	3971	2489	290.4
f-50-500_T2300	500	2300	1891	-2.3802	7332	4638	114.6
f-50-500_T2500	500	2500	1892	0.3556	8178	5988	49.2
f-50-500_T2700	500	2700	1895	0.4535	18,619	11,653	41.2
f-50-500_T2900	500	2900	1893	0.5532	28,908	21,246	40.7
f-50-700_T1700	700	1700	1154	1.0000	0.0	0.0	0.0
f-50-700_T1900	700	1900	1812	-2.1958	287.0	208.8	914.4
f-50-700_T2100	700	2100	1874	0.5760	447.0	131.8	62.6
f-50-700_T2500	700	2500	1894	0.5067	7959	4585	36.6
f-50-700_T2700	700	2700	1893	0.5340	15,095	9267	25.5
f-50-700_T2900	700	2900	1894	0.5567	28,359	20,488	35.2
f-50-900_T2100	900	2100	1876	-61.9189	6814	3912	88,914
f-50-900_T2500	900	2500	1884	0.0569	9421	5565	56.9
f-50-900_T2900	900	2900	1893	0.7841	19,103	12,743	41.3
**Repeat: strat43 Case: B Model: RF**							
f-50-1_T1700	1	1700	357	0.0000	1343	789.5	78,951
f-50-1_T1900	1	1900	1318	0.0000	551.6	323.4	32,336
f-50-1_T2100	1	2100	1816	-437.3	6823	4223	767.7
f-50-1_T2500	1	2500	1892	0.5030	6167	4914	57.0
f-50-1_T2700	1	2700	1893	0.9342	5556	3477	14.7

continued on next page

Table S12 (continued)

Simulation	$n(O_2)$	T (K)	n_{rows}	R^2	RMSE (molecule·ps)	MAE (molecule·ps)	MAPE (%)
f-50-1_T2900	1	2900	1892	0.7139	21,639	14,572	26.6
f-50-100_T1500	100	1500	175	0.0000	4.5	2.0	201.1
f-50-100_T1700	100	1700	663	0.0000	804.0	553.9	55,395
f-50-100_T1900	100	1900	1458	-47,603.8	2898	1971	119,974
f-50-100_T2100	100	2100	1827	-10.6931	3503	1983	154.0
f-50-100_T2500	100	2500	1891	-0.4388	12,861	9577	79.8
f-50-100_T2700	100	2700	1893	0.7616	10,637	6870	19.5
f-50-100_T2900	100	2900	1893	0.7031	23,812	16,463	26.0
f-50-300_T1500	300	1500	408	0.0000	0.4	0.0	3.7
f-50-300_T1700	300	1700	1215	0.0000	382.7	138.7	13,865
f-50-300_T1900	300	1900	1562	-6.85×10^6	1752	1138	105,769
f-50-300_T2100	300	2100	1869	-2055.4	22,155	14,401	1797
f-50-300_T2300	300	2300	1889	-6.2664	8883	6256	126.0
f-50-300_T2500	300	2500	1894	0.7857	5218	3679	195.9
f-50-300_T2700	300	2700	1893	0.5375	17,986	10,813	28.6
f-50-300_T2900	300	2900	1895	0.0670	44,187	30,575	40.5
f-50-500_T1500	500	1500	434	0.0000	3728	3016	301,613
f-50-500_T2100	500	2100	1886	-16.9754	2892	1988	685.3
f-50-500_T2300	500	2300	1891	-0.8182	5378	3169	97.1
f-50-500_T2500	500	2500	1892	0.7478	5117	3733	34.7
f-50-500_T2700	500	2700	1895	0.9570	5225	3471	22.8
f-50-500_T2900	500	2900	1893	0.2659	37,053	26,637	46.5
f-50-700_T1700	700	1700	1154	0.0000	2438	1801	180,099
f-50-700_T1900	700	1900	1812	-139.6	1903	1354	1699
f-50-700_T2100	700	2100	1874	-5.6225	1767	873.1	284.1
f-50-700_T2500	700	2500	1894	0.7099	6104	4090	26.1
f-50-700_T2700	700	2700	1893	0.9190	6293	4253	15.0
f-50-700_T2900	700	2900	1894	0.8211	18,018	13,698	27.5
f-50-900_T2100	900	2100	1876	-32.3920	4964	4179	28,411
f-50-900_T2500	900	2500	1884	-0.5802	12,195	8501	55.9
f-50-900_T2900	900	2900	1893	0.7817	19,210	11,044	18.7
**Repeat: strat43**	**Case: B**	**Model: XGBoost**					
f-50-1_T1700	1	1700	357	0.0000	1027	717.5	71,752
f-50-1_T1900	1	1900	1318	0.0000	2319	1572	157,166
f-50-1_T2100	1	2100	1816	-726.2	8789	4593	2197
f-50-1_T2500	1	2500	1892	0.2008	7821	6865	195.6

continued on next page

Table S12 (continued)

Simulation	$n(O_2)$	T (K)	n_{rows}	R^2	RMSE (molecule·ps)	MAE (molecule·ps)	MAPE (%)
f-50-1_T2700	1	2700	1893	0.9503	4830	3521	39.2
f-50-1_T2900	1	2900	1892	0.7757	19,158	13,920	33.8
f-50-100_T1500	100	1500	175	0.0000	895.7	644.6	64,463
f-50-100_T1700	100	1700	663	0.0000	1310	926.1	92,605
f-50-100_T1900	100	1900	1458	-893,175.4	12,551	8217	476,082
f-50-100_T2100	100	2100	1827	-8.4003	3141	2217	349.4
f-50-100_T2500	100	2500	1891	0.5059	7537	5685	139.4
f-50-100_T2700	100	2700	1893	0.4669	15,906	10,937	39.7
f-50-100_T2900	100	2900	1893	0.8456	17,172	13,473	40.0
f-50-300_T1500	300	1500	408	0.0000	815.6	737.6	73,757
f-50-300_T1700	300	1700	1215	0.0000	6120	3739	373,928
f-50-300_T1900	300	1900	1562	-2.73×10^7	3501	2152	200,909
f-50-300_T2100	300	2100	1869	-974.9	15,262	8086	3190
f-50-300_T2300	300	2300	1889	-4.6621	7841	5346	163.7
f-50-300_T2500	300	2500	1894	-1.2141	16,771	10,303	397.3
f-50-300_T2700	300	2700	1893	0.2848	22,365	14,965	45.4
f-50-300_T2900	300	2900	1895	0.2663	39,185	25,094	36.4
f-50-500_T1500	500	1500	434	0.0000	1473	898.9	89,890
f-50-500_T2100	500	2100	1886	-13.7370	2618	1722	5117
f-50-500_T2300	500	2300	1891	0.2534	3446	2085	210.9
f-50-500_T2500	500	2500	1892	0.8160	4370	3025	43.6
f-50-500_T2700	500	2700	1895	0.9202	7115	4636	37.1
f-50-500_T2900	500	2900	1893	-0.1913	47,201	34,069	55.9
f-50-700_T1700	700	1700	1154	0.0000	1530	1221	122,109
f-50-700_T1900	700	1900	1812	-91.1756	1541	1114	7931
f-50-700_T2100	700	2100	1874	-16.9932	2912	1794	5013
f-50-700_T2500	700	2500	1894	0.8724	4049	2613	32.5
f-50-700_T2700	700	2700	1893	0.9731	3626	2449	17.6
f-50-700_T2900	700	2900	1894	0.5644	28,113	17,367	26.0
f-50-900_T2100	900	2100	1876	-23.0501	4213	3216	92,079
f-50-900_T2500	900	2500	1884	-0.4080	11,512	6924	68.7
f-50-900_T2900	900	2900	1893	0.8298	16,958	12,886	40.1
**Repeat: strat43 Case: B Model: CatBoost**							
f-50-1_T1700	1	1700	357	0.0000	1779	1081	108,086
f-50-1_T1900	1	1900	1318	0.0000	1303	890.5	89,050
f-50-1_T2100	1	2100	1816	-365.6	6240	2926	1472

continued on next page

Table S12 (continued)

Simulation	$n(O_2)$	T (K)	n_{rows}	R^2	RMSE (molecule·ps)	MAE (molecule·ps)	MAPE (%)
f-50-1_T2500	1	2500	1892	0.7784	4118	3279	81.2
f-50-1_T2700	1	2700	1893	0.9679	3880	3301	29.4
f-50-1_T2900	1	2900	1892	0.7719	19,321	15,348	33.0
f-50-100_T1500	100	1500	175	0.0000	1704	933.8	93,379
f-50-100_T1700	100	1700	663	0.0000	1122	777.7	77,770
f-50-100_T1900	100	1900	1458	-262,857.7	6809	3942	168,076
f-50-100_T2100	100	2100	1827	-4.8130	2470	1627	378.1
f-50-100_T2500	100	2500	1891	-0.9392	14,931	10,451	85.5
f-50-100_T2700	100	2700	1893	0.6054	13,685	9880	37.8
f-50-100_T2900	100	2900	1893	0.9655	8116	6537	22.6
f-50-300_T1500	300	1500	408	0.0000	447.7	319.1	31,910
f-50-300_T1700	300	1700	1215	0.0000	2802	1274	127,424
f-50-300_T1900	300	1900	1562	-9.88×10^6	2104	1458	138,749
f-50-300_T2100	300	2100	1869	-1561.6	19,313	12,142	3379
f-50-300_T2300	300	2300	1889	-5.3058	8275	5573	137.6
f-50-300_T2500	300	2500	1894	-0.9299	15,658	8349	447.6
f-50-300_T2700	300	2700	1893	0.8454	10,398	5870	32.2
f-50-300_T2900	300	2900	1895	0.2244	40,288	26,705	39.0
f-50-500_T1500	500	1500	434	0.0000	3669	2595	259,464
f-50-500_T2100	500	2100	1886	-8.4879	2101	1620	10,719
f-50-500_T2300	500	2300	1891	-0.1781	4329	2393	129.9
f-50-500_T2500	500	2500	1892	0.8445	4018	2836	42.9
f-50-500_T2700	500	2700	1895	0.8669	9188	5791	28.1
f-50-500_T2900	500	2900	1893	0.0567	42,003	28,405	47.4
f-50-700_T1700	700	1700	1154	0.0000	3230	2040	203,951
f-50-700_T1900	700	1900	1812	-90.4009	1535	988.8	7238
f-50-700_T2100	700	2100	1874	0.0296	676.2	508.5	1202
f-50-700_T2500	700	2500	1894	0.6081	7094	4298	34.2
f-50-700_T2700	700	2700	1893	0.9771	3345	2232	19.8
f-50-700_T2900	700	2900	1894	0.5956	27,086	19,125	33.4
f-50-900_T2100	900	2100	1876	-11.8854	3084	2066	28,880
f-50-900_T2500	900	2500	1884	-0.6288	12,381	7940	63.4
f-50-900_T2900	900	2900	1893	0.8260	17,147	10,837	26.0
**Repeat: strat43**	**Case: B**	**Model: ANN**					
f-50-1_T1700	1	1700	357	0.0000	302.6	250.7	25,068
f-50-1_T1900	1	1900	1318	0.0000	1155	774.1	77,406

continued on next page

Table S12 (continued)

Simulation	$n(O_2)$	T (K)	n_{rows}	R^2	RMSE (molecule·ps)	MAE (molecule·ps)	MAPE (%)
f-50-1_T2100	1	2100	1816	-13.5000	1241	720.9	1860
f-50-1_T2500	1	2500	1892	0.7851	4055	3513	75.0
f-50-1_T2700	1	2700	1893	0.9538	4658	3154	34.3
f-50-1_T2900	1	2900	1892	0.9553	8548	5073	11.3
f-50-100_T1500	100	1500	175	0.0000	298.6	250.9	25,091
f-50-100_T1700	100	1700	663	0.0000	967.6	895.8	89,581
f-50-100_T1900	100	1900	1458	-31.0283	75.2	57.8	4367
f-50-100_T2100	100	2100	1827	0.3204	844.4	735.8	750.0
f-50-100_T2500	100	2500	1891	0.9610	2118	1541	43.4
f-50-100_T2700	100	2700	1893	0.9569	4522	2981	16.6
f-50-100_T2900	100	2900	1893	0.9138	12,826	8516	23.5
f-50-300_T1500	300	1500	408	0.0000	1183	1027	102,746
f-50-300_T1700	300	1700	1215	0.0000	1458	632.9	63,287
f-50-300_T1900	300	1900	1562	-1.72×10^7	2778	982.6	83,485
f-50-300_T2100	300	2100	1869	-547.4	11,441	6631	3274
f-50-300_T2300	300	2300	1889	-0.6819	4274	2520	121.2
f-50-300_T2500	300	2500	1894	0.9192	3204	2321	313.3
f-50-300_T2700	300	2700	1893	0.9462	6134	4100	19.3
f-50-300_T2900	300	2900	1895	0.9619	8928	6383	15.2
f-50-500_T1500	500	1500	434	0.0000	295.3	165.1	16,510
f-50-500_T2100	500	2100	1886	0.6797	386.0	283.1	394.9
f-50-500_T2300	500	2300	1891	0.1199	3741	1786	46.7
f-50-500_T2500	500	2500	1892	0.8603	3807	2210	25.5
f-50-500_T2700	500	2700	1895	0.9785	3693	2821	23.3
f-50-500_T2900	500	2900	1893	0.6175	26,745	18,186	50.3
f-50-700_T1700	700	1700	1154	0.0000	7619	6407	640,745
f-50-700_T1900	700	1900	1812	-764.8	4442	3289	22,892
f-50-700_T2100	700	2100	1874	0.5959	436.4	281.0	526.0
f-50-700_T2500	700	2500	1894	0.9415	2742	1782	22.0
f-50-700_T2700	700	2700	1893	0.9790	3208	2212	15.4
f-50-700_T2900	700	2900	1894	0.9758	6626	4981	15.7
f-50-900_T2100	900	2100	1876	-2.4569	1597	1316	16,691
f-50-900_T2500	900	2500	1884	0.7492	4859	2768	28.9
f-50-900_T2900	900	2900	1893	0.9247	11,280	8749	22.1
**Repeat: strat43**	**Case: B**	**Model: SVR**					
f-50-1_T1700	1	1700	357	0.0000	457.2	343.7	34,374

continued on next page

Table S12 (continued)

Simulation	$n(O_2)$	T (K)	n_{rows}	R^2	RMSE (molecule·ps)	MAE (molecule·ps)	MAPE (%)
f-50-1_T1900	1	1900	1318	0.0000	579.4	563.7	56,369
f-50-1_T2100	1	2100	1816	-162.2	4163	2838	4408
f-50-1_T2500	1	2500	1892	0.8719	3130	2728	144.8
f-50-1_T2700	1	2700	1893	0.4522	16,036	9398	43.3
f-50-1_T2900	1	2900	1892	-0.2218	44,715	32,453	49.5
f-50-100_T1500	100	1500	175	0.0000	299.3	264.3	26,432
f-50-100_T1700	100	1700	663	0.0000	193.1	117.0	11,703
f-50-100_T1900	100	1900	1458	-2605.8	678.1	400.1	17,341
f-50-100_T2100	100	2100	1827	-33.4010	6008	4264	589.9
f-50-100_T2500	100	2500	1891	0.9066	3277	2277	41.3
f-50-100_T2700	100	2700	1893	0.3644	17,368	10,890	33.0
f-50-100_T2900	100	2900	1893	-0.5057	53,620	38,451	52.7
f-50-300_T1500	300	1500	408	0.0000	3024	2658	265,793
f-50-300_T1700	300	1700	1215	0.0000	1190	905.4	90,542
f-50-300_T1900	300	1900	1562	-5.19×10^6	1525	918.9	86,333
f-50-300_T2100	300	2100	1869	-441.0	10,271	7748	3946
f-50-300_T2300	300	2300	1889	-3.0255	6612	5395	192.7
f-50-300_T2500	300	2500	1894	0.8780	3936	3046	125.3
f-50-300_T2700	300	2700	1893	-0.0957	27,683	18,636	44.3
f-50-300_T2900	300	2900	1895	-0.5866	57,620	41,347	55.7
f-50-500_T1500	500	1500	434	0.0000	679.4	537.2	53,719
f-50-500_T2100	500	2100	1886	-76.3677	5999	4263	4802
f-50-500_T2300	500	2300	1891	-1.1641	5867	4677	356.0
f-50-500_T2500	500	2500	1892	0.8154	4377	2996	50.8
f-50-500_T2700	500	2700	1895	0.0940	23,974	15,437	42.4
f-50-500_T2900	500	2900	1893	-0.7265	56,824	41,467	66.2
f-50-700_T1700	700	1700	1154	0.0000	6455	6093	609,309
f-50-700_T1900	700	1900	1812	-356.9	3037	2886	42,531
f-50-700_T2100	700	2100	1874	-17.0588	2917	1959	4122
f-50-700_T2500	700	2500	1894	0.8514	4369	3024	38.2
f-50-700_T2700	700	2700	1893	0.2794	18,772	11,509	36.9
f-50-700_T2900	700	2900	1894	-0.3874	50,172	34,844	49.2
f-50-900_T2100	900	2100	1876	-13.2070	3238	2728	50,481
f-50-900_T2500	900	2500	1884	0.8273	4032	2691	216.8
f-50-900_T2900	900	2900	1893	-0.3912	48,490	32,847	76.2
**Repeat: strat43 Case: B Model: GPR**							

continued on next page

Table S12 (continued)

Simulation	$n(O_2)$	T (K)	n_{rows}	R^2	RMSE (molecule·ps)	MAE (molecule·ps)	MAPE (%)
f-50-1_T1700	1	1700	357	0.0000	388.9	260.5	26,049
f-50-1_T1900	1	1900	1318	0.0000	348.0	227.3	22,728
f-50-1_T2100	1	2100	1816	-57.1189	2484	1407	772.6
f-50-1_T2500	1	2500	1892	0.3204	7212	5241	54.7
f-50-1_T2700	1	2700	1893	0.9231	6007	3772	14.1
f-50-1_T2900	1	2900	1892	0.8815	13,926	10,457	19.8
f-50-100_T1500	100	1500	175	0.0000	169.8	119.2	11,921
f-50-100_T1700	100	1700	663	0.0000	533.7	343.1	34,313
f-50-100_T1900	100	1900	1458	-26,526.6	2163	1296	61,376
f-50-100_T2100	100	2100	1827	-4.3378	2367	1699	267.3
f-50-100_T2500	100	2500	1891	0.9090	3234	1940	23.9
f-50-100_T2700	100	2700	1893	0.7915	9947	6509	17.7
f-50-100_T2900	100	2900	1893	0.9887	4637	3555	10.8
f-50-300_T1500	300	1500	408	0.0000	1410	1078	107,766
f-50-300_T1700	300	1700	1215	0.0000	3498	2389	238,851
f-50-300_T1900	300	1900	1562	-7.26×10^7	5706	4308	418,957
f-50-300_T2100	300	2100	1869	-405.6	9851	6457	2925
f-50-300_T2300	300	2300	1889	0.5051	2318	1735	69.3
f-50-300_T2500	300	2500	1894	0.8918	3708	2825	155.6
f-50-300_T2700	300	2700	1893	0.9472	6079	4115	13.9
f-50-300_T2900	300	2900	1895	0.9173	13,156	10,242	19.7
f-50-500_T1500	500	1500	434	0.0000	373.8	269.8	26,982
f-50-500_T2100	500	2100	1886	-12.6533	2520	1747	1717
f-50-500_T2300	500	2300	1891	0.9179	1142	764.3	78.7
f-50-500_T2500	500	2500	1892	0.9366	2565	1518	19.2
f-50-500_T2700	500	2700	1895	0.9712	4275	3186	21.9
f-50-500_T2900	500	2900	1893	0.4426	32,286	23,304	43.1
f-50-700_T1700	700	1700	1154	0.0000	3531	3247	324,741
f-50-700_T1900	700	1900	1812	-124.9	1801	1606	29,392
f-50-700_T2100	700	2100	1874	-3.1040	1391	929.9	680.9
f-50-700_T2500	700	2500	1894	0.9076	3445	2225	17.9
f-50-700_T2700	700	2700	1893	0.9753	3475	2384	11.6
f-50-700_T2900	700	2900	1894	0.9150	12,420	9524	18.4
f-50-900_T2100	900	2100	1876	-1.1887	1271	1074	45,366
f-50-900_T2500	900	2500	1884	-0.2607	10,893	8707	94.2
f-50-900_T2900	900	2900	1893	0.4081	31,629	25,830	73.7

continued on next page

Table S12 (continued)

Simulation	$n(O_2)$	T (K)	n_{rows}	R^2	RMSE (molecule·ps)	MAE (molecule·ps)	MAPE (%)
**Repeat: strat43**	**Case: B**	**Model: persistence**					
f-50-1_T1700	1	1700	357	1.0000	0.0	0.0	0.0
f-50-1_T1900	1	1900	1318	1.0000	0.0	0.0	0.0
f-50-1_T2100	1	2100	1816	0.9631	62.6	54.9	23.7
f-50-1_T2500	1	2500	1892	0.9646	1647	1468	28.5
f-50-1_T2700	1	2700	1893	0.9644	4090	3642	27.3
f-50-1_T2900	1	2900	1892	0.9666	7390	6812	25.5
f-50-100_T1500	100	1500	175	1.0000	0.0	0.0	0.0
f-50-100_T1700	100	1700	663	1.0000	0.0	0.0	0.0
f-50-100_T1900	100	1900	1458	0.5926	8.5	3.1	11.9
f-50-100_T2100	100	2100	1827	0.9585	208.7	176.9	28.0
f-50-100_T2500	100	2500	1891	0.9636	2046	1794	29.2
f-50-100_T2700	100	2700	1893	0.9633	4174	3673	26.9
f-50-100_T2900	100	2900	1893	0.9656	8105	7371	25.8
f-50-300_T1500	300	1500	408	1.0000	0.0	0.0	0.0
f-50-300_T1700	300	1700	1215	1.0000	0.0	0.0	0.0
f-50-300_T1900	300	1900	1562	0.0667	0.6	0.1	3.4
f-50-300_T2100	300	2100	1869	0.9635	93.4	80.0	24.3
f-50-300_T2300	300	2300	1889	0.9586	670.4	572.4	28.8
f-50-300_T2500	300	2500	1894	0.9609	2228	1892	31.4
f-50-300_T2700	300	2700	1893	0.9631	5080	4441	28.5
f-50-300_T2900	300	2900	1895	0.9655	8494	7665	26.3
f-50-500_T1500	500	1500	434	1.0000	0.0	0.0	0.0
f-50-500_T2100	500	2100	1886	0.9467	157.5	113.8	27.3
f-50-500_T2300	500	2300	1891	0.9589	808.1	663.3	31.3
f-50-500_T2500	500	2500	1892	0.9581	2085	1751	28.4
f-50-500_T2700	500	2700	1895	0.9622	4897	4216	29.2
f-50-500_T2900	500	2900	1893	0.9655	8037	7244	27.0
f-50-700_T1700	700	1700	1154	1.0000	0.0	0.0	0.0
f-50-700_T1900	700	1900	1812	0.9550	34.0	27.5	21.3
f-50-700_T2100	700	2100	1874	0.9562	143.7	115.1	27.1
f-50-700_T2500	700	2500	1894	0.9619	2213	1916	28.9
f-50-700_T2700	700	2700	1893	0.9621	4303	3713	28.5
f-50-700_T2900	700	2900	1894	0.9644	8040	7131	27.6
f-50-900_T2100	900	2100	1876	0.9632	164.8	133.9	23.0
f-50-900_T2500	900	2500	1884	0.9600	1940	1651	31.2

continued on next page

Table S12 (continued)

Simulation	$n(O_2)$	T (K)	n_{rows}	R^2	RMSE (molecule·ps)	MAE (molecule·ps)	MAPE (%)
f-50-900_T2900	900	2900	1893	0.9640	7798	6850	28.8
**Repeat: strat43**	**Case: B**	**Model: physical_MLR**					
f-50-1_T1700	1	1700	357	0.0000	20,821	16,876	1,687,579
f-50-1_T1900	1	1900	1318	0.0000	14,870	12,624	1,262,415
f-50-1_T2100	1	2100	1816	-1939.6	14,357	12,122	131,275
f-50-1_T2500	1	2500	1892	-1.9443	15,010	13,381	434.5
f-50-1_T2700	1	2700	1893	0.7457	10,926	9932	318.7
f-50-1_T2900	1	2900	1892	0.4685	29,491	23,085	191.9
f-50-100_T1500	100	1500	175	0.0000	28,730	24,485	2,448,501
f-50-100_T1700	100	1700	663	0.0000	17,889	14,406	1,440,605
f-50-100_T1900	100	1900	1458	-1.23×10^6	14,742	12,479	977,990
f-50-100_T2100	100	2100	1827	-171.4	13,448	11,486	27,105
f-50-100_T2500	100	2500	1891	-0.0737	11,111	10,305	309.8
f-50-100_T2700	100	2700	1893	0.8021	9692	8868	216.7
f-50-100_T2900	100	2900	1893	0.4302	32,985	24,805	165.3
f-50-300_T1500	300	1500	408	0.0000	29,146	25,738	2,573,827
f-50-300_T1700	300	1700	1215	0.0000	19,241	15,171	1,517,095
f-50-300_T1900	300	1900	1562	-4.80×10^8	14,667	12,096	1,186,258
f-50-300_T2100	300	2100	1869	-982.9	15,325	13,138	278,504
f-50-300_T2300	300	2300	1889	-20.3681	15,233	12,821	4285
f-50-300_T2500	300	2500	1894	0.0293	11,105	10,113	2030
f-50-300_T2700	300	2700	1893	0.7793	12,424	10,417	272.0
f-50-300_T2900	300	2900	1895	0.4166	34,941	26,452	182.4
f-50-500_T1500	500	1500	434	0.0000	27,270	23,414	2,341,387
f-50-500_T2100	500	2100	1886	-447.2	14,438	12,483	299,089
f-50-500_T2300	500	2300	1891	-12.3838	14,590	12,438	7545
f-50-500_T2500	500	2500	1892	-0.4927	12,447	11,175	314.8
f-50-500_T2700	500	2700	1895	0.7528	12,524	11,032	440.5
f-50-500_T2900	500	2900	1893	0.4780	31,245	24,115	245.7
f-50-700_T1700	700	1700	1154	0.0000	19,515	15,101	1,510,135
f-50-700_T1900	700	1900	1812	-11,473.6	17,197	14,181	663,354
f-50-700_T2100	700	2100	1874	-467.5	14,859	12,802	236,696
f-50-700_T2500	700	2500	1894	0.2203	10,007	9101	340.4
f-50-700_T2700	700	2700	1893	0.7959	9990	9130	290.4
f-50-700_T2900	700	2900	1894	0.5122	29,749	22,929	270.8
f-50-900_T2100	900	2100	1876	-277.8	14,344	12,334	517,333

continued on next page

Table S12 (continued)

Simulation	$n(O_2)$	T (K)	n_{rows}	R^2	RMSE (molecule·ps)	MAE (molecule·ps)	MAPE (%)
f-50-900_T2500	900	2500	1884	-0.4604	11,724	10,696	642.1
f-50-900_T2900	900	2900	1893	0.4937	29,253	22,991	462.7
**Repeat: strat43**	**Case: B**	**Model: physical_RF**					
f-50-1_T1700	1	1700	357	0.0000	66.4	16.1	1607
f-50-1_T1900	1	1900	1318	0.0000	300.5	218.4	21,838
f-50-1_T2100	1	2100	1816	0.1114	307.2	233.3	77.2
f-50-1_T2500	1	2500	1892	0.8657	3205	2973	385.2
f-50-1_T2700	1	2700	1893	0.8463	8495	5734	72.6
f-50-1_T2900	1	2900	1892	0.7606	19,793	15,478	44.2
f-50-100_T1500	100	1500	175	0.0000	129.0	53.9	5392
f-50-100_T1700	100	1700	663	0.0000	80.9	31.4	3144
f-50-100_T1900	100	1900	1458	-3055.1	734.2	486.9	33,662
f-50-100_T2100	100	2100	1827	-0.5875	1291	964.6	87.5
f-50-100_T2500	100	2500	1891	0.9773	1614	1485	176.5
f-50-100_T2700	100	2700	1893	0.9613	4287	2756	16.3
f-50-100_T2900	100	2900	1893	0.8164	18,724	14,066	26.0
f-50-300_T1500	300	1500	408	0.0000	15.7	6.2	615.8
f-50-300_T1700	300	1700	1215	0.0000	143.7	81.3	8135
f-50-300_T1900	300	1900	1562	-905,124.8	636.9	500.9	48,282
f-50-300_T2100	300	2100	1869	-6.5928	1346	894.8	2258
f-50-300_T2300	300	2300	1889	-0.0579	3389	2443	216.1
f-50-300_T2500	300	2500	1894	0.9346	2882	1939	452.8
f-50-300_T2700	300	2700	1893	0.9705	4539	3450	26.4
f-50-300_T2900	300	2900	1895	0.8853	15,491	11,739	26.6
f-50-500_T1500	500	1500	434	0.0000	3962	3032	303,201
f-50-500_T2100	500	2100	1886	-18.9824	3049	2319	3006
f-50-500_T2300	500	2300	1891	0.7833	1856	1293	150.0
f-50-500_T2500	500	2500	1892	0.8985	3245	2243	30.2
f-50-500_T2700	500	2700	1895	0.9071	7677	6279	122.5
f-50-500_T2900	500	2900	1893	0.9429	10,331	7851	25.3
f-50-700_T1700	700	1700	1154	0.0000	1084	868.0	86,803
f-50-700_T1900	700	1900	1812	-19.3893	724.9	515.3	726.8
f-50-700_T2100	700	2100	1874	0.6631	398.5	286.5	102.5
f-50-700_T2500	700	2500	1894	0.9862	1332	919.2	118.4
f-50-700_T2700	700	2700	1893	0.9672	4004	2331	32.6
f-50-700_T2900	700	2900	1894	0.8948	13,815	10,206	23.0

continued on next page

Table S12 (continued)

Simulation	$n(O_2)$	T (K)	n_{rows}	R^2	RMSE (molecule·ps)	MAE (molecule·ps)	MAPE (%)
f-50-900_T2100	900	2100	1876	-72.9389	7386	5618	16,043
f-50-900_T2500	900	2500	1884	0.1471	8960	6569	65.9
f-50-900_T2900	900	2900	1893	0.4621	30,152	22,551	50.1
**Repeat: strat44 Case: A Model: MLR**							
f-50-1_T1700	1	1700	357	0.0000	442.9	431.5	43,147
f-50-1_T1900	1	1900	1318	0.0000	501.3	418.6	41,865
f-50-1_T2100	1	2100	1816	-0.6972	424.6	408.3	4540
f-50-1_T2500	1	2500	1892	0.9952	605.1	556.9	121.0
f-50-1_T2700	1	2700	1893	0.9989	732.5	638.9	32.5
f-50-1_T2900	1	2900	1892	0.9984	1626	1467	13.6
f-50-100_T1500	100	1500	175	0.0000	993.1	989.8	98,980
f-50-100_T1700	100	1700	663	0.0000	464.5	397.9	39,788
f-50-100_T1900	100	1900	1458	-66.6130	109.2	92.1	6985
f-50-100_T2100	100	2100	1827	0.9073	311.9	258.0	690.6
f-50-100_T2500	100	2500	1891	0.9982	448.7	346.1	115.4
f-50-100_T2700	100	2700	1893	0.9994	524.7	433.5	27.5
f-50-100_T2900	100	2900	1893	0.9989	1436	1312	11.8
f-50-300_T1500	300	1500	408	0.0000	623.2	614.7	61,466
f-50-300_T1700	300	1700	1215	0.0000	529.7	523.4	52,339
f-50-300_T1900	300	1900	1562	-17,981.8	89.8	74.8	7411
f-50-300_T2100	300	2100	1869	-0.0960	511.5	501.8	7005
f-50-300_T2300	300	2300	1889	0.9839	418.7	405.7	244.0
f-50-300_T2500	300	2500	1894	0.9978	533.9	511.9	339.1
f-50-300_T2700	300	2700	1893	0.9997	445.2	370.6	27.4
f-50-300_T2900	300	2900	1895	0.9986	1725	1463	6.6
f-50-500_T1500	500	1500	434	0.0000	1292	1290	129,019
f-50-500_T2100	500	2100	1886	0.7842	316.8	294.3	4091
f-50-500_T2300	500	2300	1891	0.9683	709.9	688.4	382.9
f-50-500_T2500	500	2500	1892	0.9940	791.9	775.6	84.8
f-50-500_T2700	500	2700	1895	0.9994	599.9	475.9	64.6
f-50-500_T2900	500	2900	1893	0.9993	1163	1001	18.4
f-50-700_T1700	700	1700	1154	0.0000	1150	1139	113,871
f-50-700_T1900	700	1900	1812	-30.5343	901.5	888.7	16,212
f-50-700_T2100	700	2100	1874	0.7973	309.1	226.4	1266
f-50-700_T2500	700	2500	1894	0.9965	675.2	648.3	78.1
f-50-700_T2700	700	2700	1893	0.9996	449.5	370.9	8.0

continued on next page

Table S12 (continued)

Simulation	$n(O_2)$	T (K)	n_{rows}	R^2	RMSE (molecule·ps)	MAE (molecule·ps)	MAPE (%)
f-50-700_T2900	700	2900	1894	0.9993	1108	982.0	24.0
f-50-900_T2100	900	2100	1876	-0.9344	1195	1169	22,341
f-50-900_T2500	900	2500	1884	0.9983	401.1	359.0	170.9
f-50-900_T2900	900	2900	1893	0.9993	1069	937.9	9.9
**Repeat: strat44 Case: A Model: DT**							
f-50-1_T1700	1	1700	357	0.0000	7.2	3.0	299.3
f-50-1_T1900	1	1900	1318	0.0000	2.9	1.7	171.8
f-50-1_T2100	1	2100	1816	0.9432	77.6	56.5	86.8
f-50-1_T2500	1	2500	1892	0.9982	369.2	282.4	7.8
f-50-1_T2700	1	2700	1893	0.9998	320.7	251.4	6.1
f-50-1_T2900	1	2900	1892	0.9995	859.8	678.8	3.3
f-50-100_T1500	100	1500	175	0.0000	4.6	2.3	225.6
f-50-100_T1700	100	1700	663	0.0000	5.0	2.0	199.7
f-50-100_T1900	100	1900	1458	-0.2079	14.6	6.4	177.0
f-50-100_T2100	100	2100	1827	0.9582	209.5	134.6	70.9
f-50-100_T2500	100	2500	1891	0.9993	292.8	216.1	10.5
f-50-100_T2700	100	2700	1893	0.9997	389.0	294.4	6.4
f-50-100_T2900	100	2900	1893	0.9997	722.8	475.2	2.9
f-50-300_T1500	300	1500	408	0.0000	1.0	1.0	102.5
f-50-300_T1700	300	1700	1215	0.0000	5.4	3.3	334.6
f-50-300_T1900	300	1900	1562	-93.5232	6.5	3.4	339.7
f-50-300_T2100	300	2100	1869	0.9680	87.4	65.3	116.7
f-50-300_T2300	300	2300	1889	0.9940	255.5	210.2	20.1
f-50-300_T2500	300	2500	1894	0.9990	354.8	200.3	35.7
f-50-300_T2700	300	2700	1893	0.9997	494.4	345.0	6.2
f-50-300_T2900	300	2900	1895	0.9997	808.8	457.0	4.4
f-50-500_T1500	500	1500	434	0.0000	3.1	3.1	310.6
f-50-500_T2100	500	2100	1886	0.9907	65.7	48.1	128.1
f-50-500_T2300	500	2300	1891	0.9931	330.4	238.1	34.6
f-50-500_T2500	500	2500	1892	0.9996	191.5	151.3	6.5
f-50-500_T2700	500	2700	1895	0.9997	418.2	310.7	7.6
f-50-500_T2900	500	2900	1893	0.9999	328.2	279.9	3.4
f-50-700_T1700	700	1700	1154	0.0000	1.0	1.0	102.5
f-50-700_T1900	700	1900	1812	0.8679	58.4	42.3	50.7
f-50-700_T2100	700	2100	1874	0.9949	49.1	38.8	120.7
f-50-700_T2500	700	2500	1894	0.9996	217.5	133.8	8.0

continued on next page

Table S12 (continued)

Simulation	$n(O_2)$	T (K)	n_{rows}	R^2	RMSE (molecule·ps)	MAE (molecule·ps)	MAPE (%)
f-50-700_T2700	700	2700	1893	0.9996	433.2	357.1	7.6
f-50-700_T2900	700	2900	1894	1.0000	266.6	212.5	3.3
f-50-900_T2100	900	2100	1876	0.9652	160.3	90.8	688.5
f-50-900_T2500	900	2500	1884	0.9996	202.2	139.2	24.1
f-50-900_T2900	900	2900	1893	0.9999	341.4	254.7	6.5
**Repeat: strat44**	**Case: A**	**Model: RF**					
f-50-1_T1700	1	1700	357	0.0000	6.2	2.7	267.8
f-50-1_T1900	1	1900	1318	0.0000	57.9	30.5	3045
f-50-1_T2100	1	2100	1816	0.9533	70.4	53.3	95.9
f-50-1_T2500	1	2500	1892	0.9984	346.1	269.0	11.5
f-50-1_T2700	1	2700	1893	0.9998	325.4	252.1	3.1
f-50-1_T2900	1	2900	1892	0.9995	861.2	672.4	2.7
f-50-100_T1500	100	1500	175	0.0000	2.6	2.0	203.9
f-50-100_T1700	100	1700	663	0.0000	4.5	2.4	235.6
f-50-100_T1900	100	1900	1458	0.0156	13.2	6.0	181.2
f-50-100_T2100	100	2100	1827	0.9819	137.9	100.8	54.0
f-50-100_T2500	100	2500	1891	0.9998	166.9	116.3	9.2
f-50-100_T2700	100	2700	1893	0.9999	222.8	186.0	4.4
f-50-100_T2900	100	2900	1893	0.9999	497.3	372.6	2.6
f-50-300_T1500	300	1500	408	0.0000	1.0	1.0	103.7
f-50-300_T1700	300	1700	1215	0.0000	4.7	3.0	298.2
f-50-300_T1900	300	1900	1562	-4178.1	43.3	25.3	2393
f-50-300_T2100	300	2100	1869	0.9759	75.8	57.5	81.7
f-50-300_T2300	300	2300	1889	0.9954	222.9	202.6	19.1
f-50-300_T2500	300	2500	1894	0.9997	191.3	148.7	29.1
f-50-300_T2700	300	2700	1893	0.9997	418.2	288.2	6.0
f-50-300_T2900	300	2900	1895	0.9997	817.6	448.9	3.9
f-50-500_T1500	500	1500	434	0.0000	2.8	2.8	277.3
f-50-500_T2100	500	2100	1886	0.9935	55.0	43.1	116.9
f-50-500_T2300	500	2300	1891	0.9956	264.2	207.5	35.0
f-50-500_T2500	500	2500	1892	0.9997	162.0	141.1	5.6
f-50-500_T2700	500	2700	1895	0.9998	315.2	262.2	6.8
f-50-500_T2900	500	2900	1893	1.0000	261.0	235.0	2.8
f-50-700_T1700	700	1700	1154	0.0000	1.0	1.0	103.7
f-50-700_T1900	700	1900	1812	0.9071	48.9	36.9	49.0
f-50-700_T2100	700	2100	1874	0.9976	33.9	25.8	86.9

continued on next page

Table S12 (continued)

Simulation	$n(O_2)$	T (K)	n_{rows}	R^2	RMSE (molecule·ps)	MAE (molecule·ps)	MAPE (%)
f-50-700_T2500	700	2500	1894	0.9999	112.9	80.8	5.1
f-50-700_T2700	700	2700	1893	0.9998	343.6	291.7	6.2
f-50-700_T2900	700	2900	1894	1.0000	194.8	153.0	2.6
f-50-900_T2100	900	2100	1876	0.9741	138.1	79.9	626.9
f-50-900_T2500	900	2500	1884	0.9999	104.8	76.7	20.9
f-50-900_T2900	900	2900	1893	1.0000	275.5	219.9	5.2
**Repeat: strat44 Case: A Model: XGBoost**							
f-50-1_T1700	1	1700	357	0.0000	3.3	1.9	187.7
f-50-1_T1900	1	1900	1318	0.0000	10.7	2.3	233.3
f-50-1_T2100	1	2100	1816	0.7921	148.6	124.5	117.5
f-50-1_T2500	1	2500	1892	0.9985	337.7	286.9	14.9
f-50-1_T2700	1	2700	1893	0.9997	348.4	263.9	3.6
f-50-1_T2900	1	2900	1892	0.9996	844.5	600.7	2.7
f-50-100_T1500	100	1500	175	0.0000	2.3	1.9	193.0
f-50-100_T1700	100	1700	663	0.0000	2.0	1.0	104.9
f-50-100_T1900	100	1900	1458	0.8553	5.1	3.1	184.3
f-50-100_T2100	100	2100	1827	0.9963	62.2	47.3	43.3
f-50-100_T2500	100	2500	1891	0.9997	178.1	140.1	8.6
f-50-100_T2700	100	2700	1893	0.9998	326.6	259.3	3.7
f-50-100_T2900	100	2900	1893	0.9992	1206	738.1	2.8
f-50-300_T1500	300	1500	408	0.0000	0.8	0.6	64.4
f-50-300_T1700	300	1700	1215	0.0000	3.5	2.9	292.9
f-50-300_T1900	300	1900	1562	-2085.5	30.6	18.0	1721
f-50-300_T2100	300	2100	1869	0.9620	95.3	66.2	57.1
f-50-300_T2300	300	2300	1889	0.9939	258.3	216.8	20.4
f-50-300_T2500	300	2500	1894	0.9998	159.1	124.7	70.9
f-50-300_T2700	300	2700	1893	0.9997	449.3	358.0	10.7
f-50-300_T2900	300	2900	1895	0.9985	1756	995.0	3.4
f-50-500_T1500	500	1500	434	0.0000	1.9	1.4	135.1
f-50-500_T2100	500	2100	1886	0.9949	48.6	39.6	257.3
f-50-500_T2300	500	2300	1891	0.9981	174.5	125.7	21.8
f-50-500_T2500	500	2500	1892	0.9997	164.0	135.8	5.2
f-50-500_T2700	500	2700	1895	0.9997	408.0	277.6	7.6
f-50-500_T2900	500	2900	1893	0.9994	1014	565.2	2.2
f-50-700_T1700	700	1700	1154	0.0000	10.3	9.6	955.5
f-50-700_T1900	700	1900	1812	0.9661	29.6	21.1	232.4

continued on next page

Table S12 (continued)

Simulation	$n(O_2)$	T (K)	n_{rows}	R^2	RMSE (molecule·ps)	MAE (molecule·ps)	MAPE (%)
f-50-700_T2100	700	2100	1874	0.9942	52.1	35.1	65.6
f-50-700_T2500	700	2500	1894	0.9998	168.3	128.1	3.7
f-50-700_T2700	700	2700	1893	0.9995	508.3	393.9	4.6
f-50-700_T2900	700	2900	1894	0.9998	647.7	410.4	2.2
f-50-900_T2100	900	2100	1876	0.9873	96.7	81.1	700.2
f-50-900_T2500	900	2500	1884	0.9995	220.9	173.2	16.9
f-50-900_T2900	900	2900	1893	0.9991	1213	965.3	6.3
**Repeat: strat44**		**Case: A**		**Model: CatBoost**			
f-50-1_T1700	1	1700	357	0.0000	32.1	24.3	2431
f-50-1_T1900	1	1900	1318	0.0000	30.4	24.6	2459
f-50-1_T2100	1	2100	1816	0.8765	114.5	88.9	193.5
f-50-1_T2500	1	2500	1892	0.9989	289.0	222.6	11.7
f-50-1_T2700	1	2700	1893	0.9990	687.6	535.2	4.6
f-50-1_T2900	1	2900	1892	0.9987	1446	968.8	4.1
f-50-100_T1500	100	1500	175	0.0000	47.5	39.2	3919
f-50-100_T1700	100	1700	663	0.0000	31.1	27.3	2727
f-50-100_T1900	100	1900	1458	-7.9648	39.8	32.6	2941
f-50-100_T2100	100	2100	1827	0.8851	347.2	288.0	49.6
f-50-100_T2500	100	2500	1891	0.9993	284.9	209.3	7.1
f-50-100_T2700	100	2700	1893	0.9990	694.2	564.9	6.0
f-50-100_T2900	100	2900	1893	0.9992	1254	894.0	4.5
f-50-300_T1500	300	1500	408	0.0000	70.8	60.3	6027
f-50-300_T1700	300	1700	1215	0.0000	39.7	30.3	3031
f-50-300_T1900	300	1900	1562	-22,618.1	100.7	82.3	7974
f-50-300_T2100	300	2100	1869	0.9520	107.0	77.5	144.0
f-50-300_T2300	300	2300	1889	0.9927	281.9	230.9	22.0
f-50-300_T2500	300	2500	1894	0.9994	273.4	214.6	53.9
f-50-300_T2700	300	2700	1893	0.9995	572.7	420.0	9.3
f-50-300_T2900	300	2900	1895	0.9973	2375	1464	5.6
f-50-500_T1500	500	1500	434	0.0000	78.2	63.7	6374
f-50-500_T2100	500	2100	1886	0.9557	143.6	120.6	1310
f-50-500_T2300	500	2300	1891	0.9917	363.0	215.4	27.3
f-50-500_T2500	500	2500	1892	0.9991	308.4	228.4	7.0
f-50-500_T2700	500	2700	1895	0.9995	544.5	405.9	9.8
f-50-500_T2900	500	2900	1893	0.9995	997.4	808.0	4.0
f-50-700_T1700	700	1700	1154	0.0000	74.5	63.8	6385

continued on next page

Table S12 (continued)

Simulation	$n(O_2)$	T (K)	n_{rows}	R^2	RMSE (molecule·ps)	MAE (molecule·ps)	MAPE (%)
f-50-700_T1900	700	1900	1812	0.8127	69.5	44.3	635.8
f-50-700_T2100	700	2100	1874	0.9777	102.5	74.0	175.7
f-50-700_T2500	700	2500	1894	0.9987	403.6	296.8	9.2
f-50-700_T2700	700	2700	1893	0.9990	703.0	533.9	5.7
f-50-700_T2900	700	2900	1894	0.9993	1099	867.3	4.6
f-50-900_T2100	900	2100	1876	0.9857	102.7	82.0	1921
f-50-900_T2500	900	2500	1884	0.9951	677.8	540.8	47.8
f-50-900_T2900	900	2900	1893	0.9994	968.5	756.2	5.0
**Repeat: strat44**	**Case: A**	**Model: ANN**					
f-50-1_T1700	1	1700	357	0.0000	128.7	105.7	10,566
f-50-1_T1900	1	1900	1318	0.0000	29.6	18.9	1886
f-50-1_T2100	1	2100	1816	-2.1982	582.8	301.5	413.9
f-50-1_T2500	1	2500	1892	0.9963	532.5	461.6	42.0
f-50-1_T2700	1	2700	1893	0.9996	414.0	340.0	8.6
f-50-1_T2900	1	2900	1892	0.9997	730.5	550.9	9.3
f-50-100_T1500	100	1500	175	0.0000	163.5	130.5	13,050
f-50-100_T1700	100	1700	663	0.0000	106.9	87.3	8729
f-50-100_T1900	100	1900	1458	-33.8908	78.4	64.1	5659
f-50-100_T2100	100	2100	1827	0.5039	721.5	595.4	96.6
f-50-100_T2500	100	2500	1891	0.9967	617.5	501.9	10.3
f-50-100_T2700	100	2700	1893	0.9997	344.7	262.2	4.9
f-50-100_T2900	100	2900	1893	0.9997	709.5	563.0	4.7
f-50-300_T1500	300	1500	408	0.0000	121.1	87.7	8773
f-50-300_T1700	300	1700	1215	0.0000	156.1	117.8	11,777
f-50-300_T1900	300	1900	1562	-13,236.2	77.0	66.1	6572
f-50-300_T2100	300	2100	1869	0.3773	385.5	271.0	290.1
f-50-300_T2300	300	2300	1889	0.9352	839.1	584.0	27.0
f-50-300_T2500	300	2500	1894	0.9900	1125	826.3	28.2
f-50-300_T2700	300	2700	1893	0.9992	762.7	598.5	5.4
f-50-300_T2900	300	2900	1895	0.9998	588.8	521.0	6.0
f-50-500_T1500	500	1500	434	0.0000	107.1	72.5	7250
f-50-500_T2100	500	2100	1886	0.9039	211.4	164.4	453.3
f-50-500_T2300	500	2300	1891	0.9884	429.1	353.5	58.4
f-50-500_T2500	500	2500	1892	0.9981	443.0	323.4	15.8
f-50-500_T2700	500	2700	1895	0.9995	548.1	406.4	5.4
f-50-500_T2900	500	2900	1893	0.9996	905.7	667.3	4.1

continued on next page

Table S12 (continued)

Simulation	$n(O_2)$	T (K)	n_{rows}	R^2	RMSE (molecule·ps)	MAE (molecule·ps)	MAPE (%)
f-50-700_T1700	700	1700	1154	0.0000	126.4	106.7	10,668
f-50-700_T1900	700	1900	1812	-1.4434	250.9	189.4	807.7
f-50-700_T2100	700	2100	1874	0.9755	107.5	80.6	408.0
f-50-700_T2500	700	2500	1894	0.9966	658.3	531.3	13.5
f-50-700_T2700	700	2700	1893	0.9990	689.6	534.9	7.3
f-50-700_T2900	700	2900	1894	0.9994	1065	840.1	6.3
f-50-900_T2100	900	2100	1876	0.8512	331.4	196.0	1502
f-50-900_T2500	900	2500	1884	0.9979	447.1	327.5	21.5
f-50-900_T2900	900	2900	1893	0.9994	985.3	767.7	15.9
**Repeat: strat44**		**Case: A**		**Model: SVR**			
f-50-1_T1700	1	1700	357	0.0000	1275	1173	117,269
f-50-1_T1900	1	1900	1318	0.0000	1343	1219	121,884
f-50-1_T2100	1	2100	1816	-22.5037	1580	1120	6856
f-50-1_T2500	1	2500	1892	0.8716	3135	2587	179.0
f-50-1_T2700	1	2700	1893	0.7277	11,307	7545	67.5
f-50-1_T2900	1	2900	1892	0.1575	37,130	24,706	46.0
f-50-100_T1500	100	1500	175	0.0000	943.5	765.8	76,584
f-50-100_T1700	100	1700	663	0.0000	931.4	690.2	69,017
f-50-100_T1900	100	1900	1458	-1538.0	521.0	368.1	21,383
f-50-100_T2100	100	2100	1827	-0.7104	1340	1017	772.4
f-50-100_T2500	100	2500	1891	0.9862	1261	782.9	82.9
f-50-100_T2700	100	2700	1893	0.9262	5918	3171	30.5
f-50-100_T2900	100	2900	1893	0.1334	40,678	26,193	45.7
f-50-300_T1500	300	1500	408	0.0000	393.6	279.6	27,960
f-50-300_T1700	300	1700	1215	0.0000	467.5	381.7	38,172
f-50-300_T1900	300	1900	1562	-567,524.0	504.4	342.7	32,396
f-50-300_T2100	300	2100	1869	-13.5144	1861	1349	4222
f-50-300_T2300	300	2300	1889	0.7183	1749	1286	239.3
f-50-300_T2500	300	2500	1894	0.9836	1445	1108	582.3
f-50-300_T2700	300	2700	1893	0.8800	9160	5044	53.6
f-50-300_T2900	300	2900	1895	0.1669	41,753	27,437	34.1
f-50-500_T1500	500	1500	434	0.0000	436.0	395.9	39,593
f-50-500_T2100	500	2100	1886	-2.3620	1251	897.7	3930
f-50-500_T2300	500	2300	1891	0.9499	892.8	771.1	359.7
f-50-500_T2500	500	2500	1892	0.9438	2415	1487	71.5
f-50-500_T2700	500	2700	1895	0.7525	12,529	8034	91.5

continued on next page

Table S12 (continued)

Simulation	$n(O_2)$	T (K)	n_{rows}	R^2	RMSE (molecule·ps)	MAE (molecule·ps)	MAPE (%)
f-50-500_T2900	500	2900	1893	0.2016	38,642	25,171	58.9
f-50-700_T1700	700	1700	1154	0.0000	1518	1318	131,839
f-50-700_T1900	700	1900	1812	-21.7231	765.3	601.1	13,192
f-50-700_T2100	700	2100	1874	-4.5431	1616	1234	9395
f-50-700_T2500	700	2500	1894	0.9169	3266	1910	87.4
f-50-700_T2700	700	2700	1893	0.7644	10,733	6637	40.3
f-50-700_T2900	700	2900	1894	0.1939	38,242	24,975	62.8
f-50-900_T2100	900	2100	1876	-7.0826	2442	1984	42,518
f-50-900_T2500	900	2500	1884	0.8914	3198	2517	545.7
f-50-900_T2900	900	2900	1893	0.0639	39,776	25,709	136.6
**Repeat: strat44 Case: A Model: GPR**							
f-50-1_T1700	1	1700	357	0.0000	24.9	17.9	1795
f-50-1_T1900	1	1900	1318	0.0000	23.7	18.9	1887
f-50-1_T2100	1	2100	1816	0.9509	72.2	61.4	116.2
f-50-1_T2500	1	2500	1892	0.9996	179.1	133.8	17.8
f-50-1_T2700	1	2700	1893	1.0000	145.5	119.4	3.3
f-50-1_T2900	1	2900	1892	0.9999	422.3	381.1	2.6
f-50-100_T1500	100	1500	175	0.0000	17.8	15.4	1541
f-50-100_T1700	100	1700	663	0.0000	6.6	4.1	411.0
f-50-100_T1900	100	1900	1458	-0.2239	14.7	9.4	461.1
f-50-100_T2100	100	2100	1827	0.9864	119.3	99.4	27.5
f-50-100_T2500	100	2500	1891	0.9999	95.2	82.7	5.1
f-50-100_T2700	100	2700	1893	0.9999	169.3	135.3	2.8
f-50-100_T2900	100	2900	1893	1.0000	272.4	244.9	1.9
f-50-300_T1500	300	1500	408	0.0000	7.0	5.9	588.7
f-50-300_T1700	300	1700	1215	0.0000	17.3	16.0	1596
f-50-300_T1900	300	1900	1562	-4720.4	46.0	39.1	3897
f-50-300_T2100	300	2100	1869	0.9735	79.5	70.3	527.9
f-50-300_T2300	300	2300	1889	0.9984	133.4	100.4	11.7
f-50-300_T2500	300	2500	1894	0.9999	107.2	95.8	51.8
f-50-300_T2700	300	2700	1893	0.9999	215.3	178.9	7.5
f-50-300_T2900	300	2900	1895	1.0000	291.6	253.6	3.7
f-50-500_T1500	500	1500	434	0.0000	11.5	10.7	1065
f-50-500_T2100	500	2100	1886	0.9829	89.1	66.9	279.6
f-50-500_T2300	500	2300	1891	0.9984	159.2	119.0	34.7
f-50-500_T2500	500	2500	1892	0.9998	143.1	112.8	10.1

continued on next page

Table S12 (continued)

Simulation	$n(O_2)$	T (K)	n_{rows}	R^2	RMSE (molecule·ps)	MAE (molecule·ps)	MAPE (%)
f-50-500_T2700	500	2700	1895	1.0000	141.9	110.5	6.8
f-50-500_T2900	500	2900	1893	0.9999	390.7	350.5	2.2
f-50-700_T1700	700	1700	1154	0.0000	49.5	41.4	4145
f-50-700_T1900	700	1900	1812	0.8993	51.0	41.7	522.9
f-50-700_T2100	700	2100	1874	0.9948	49.5	39.7	224.5
f-50-700_T2500	700	2500	1894	0.9998	164.2	134.6	4.3
f-50-700_T2700	700	2700	1893	0.9999	259.4	195.1	3.0
f-50-700_T2900	700	2900	1894	0.9999	379.0	319.5	2.6
f-50-900_T2100	900	2100	1876	0.9829	112.3	89.7	756.7
f-50-900_T2500	900	2500	1884	0.9975	486.2	419.3	10.7
f-50-900_T2900	900	2900	1893	0.9997	702.8	637.0	5.5
**Repeat: strat44**	**Case: A**	**Model: persistence**					
f-50-1_T1700	1	1700	357	1.0000	0.0	0.0	0.0
f-50-1_T1900	1	1900	1318	1.0000	0.0	0.0	0.0
f-50-1_T2100	1	2100	1816	0.9631	62.6	54.9	23.7
f-50-1_T2500	1	2500	1892	0.9646	1647	1468	28.5
f-50-1_T2700	1	2700	1893	0.9644	4090	3642	27.3
f-50-1_T2900	1	2900	1892	0.9666	7390	6812	25.5
f-50-100_T1500	100	1500	175	1.0000	0.0	0.0	0.0
f-50-100_T1700	100	1700	663	1.0000	0.0	0.0	0.0
f-50-100_T1900	100	1900	1458	0.5926	8.5	3.1	11.9
f-50-100_T2100	100	2100	1827	0.9585	208.7	176.9	28.0
f-50-100_T2500	100	2500	1891	0.9636	2046	1794	29.2
f-50-100_T2700	100	2700	1893	0.9633	4174	3673	26.9
f-50-100_T2900	100	2900	1893	0.9656	8105	7371	25.8
f-50-300_T1500	300	1500	408	1.0000	0.0	0.0	0.0
f-50-300_T1700	300	1700	1215	1.0000	0.0	0.0	0.0
f-50-300_T1900	300	1900	1562	0.0667	0.6	0.1	3.4
f-50-300_T2100	300	2100	1869	0.9635	93.4	80.0	24.3
f-50-300_T2300	300	2300	1889	0.9586	670.4	572.4	28.8
f-50-300_T2500	300	2500	1894	0.9609	2228	1892	31.4
f-50-300_T2700	300	2700	1893	0.9631	5080	4441	28.5
f-50-300_T2900	300	2900	1895	0.9655	8494	7665	26.3
f-50-500_T1500	500	1500	434	1.0000	0.0	0.0	0.0
f-50-500_T2100	500	2100	1886	0.9467	157.5	113.8	27.3
f-50-500_T2300	500	2300	1891	0.9589	808.1	663.3	31.3

continued on next page

Table S12 (continued)

Simulation	$n(O_2)$	T (K)	n_{rows}	R^2	RMSE (molecule·ps)	MAE (molecule·ps)	MAPE (%)
f-50-500_T2500	500	2500	1892	0.9581	2085	1751	28.4
f-50-500_T2700	500	2700	1895	0.9622	4897	4216	29.2
f-50-500_T2900	500	2900	1893	0.9655	8037	7244	27.0
f-50-700_T1700	700	1700	1154	1.0000	0.0	0.0	0.0
f-50-700_T1900	700	1900	1812	0.9550	34.0	27.5	21.3
f-50-700_T2100	700	2100	1874	0.9562	143.7	115.1	27.1
f-50-700_T2500	700	2500	1894	0.9619	2213	1916	28.9
f-50-700_T2700	700	2700	1893	0.9621	4303	3713	28.5
f-50-700_T2900	700	2900	1894	0.9644	8040	7131	27.6
f-50-900_T2100	900	2100	1876	0.9632	164.8	133.9	23.0
f-50-900_T2500	900	2500	1884	0.9600	1940	1651	31.2
f-50-900_T2900	900	2900	1893	0.9640	7798	6850	28.8
**Repeat: strat44 Case: A Model: physical_MLR**							
f-50-1_T1700	1	1700	357	0.0000	21,123	17,083	1,708,337
f-50-1_T1900	1	1900	1318	0.0000	14,850	12,648	1,264,836
f-50-1_T2100	1	2100	1816	-1844.0	13,998	11,869	135,795
f-50-1_T2500	1	2500	1892	-1.6220	14,165	12,587	462.6
f-50-1_T2700	1	2700	1893	0.8207	9174	8316	230.6
f-50-1_T2900	1	2900	1892	0.4452	30,131	23,328	180.9
f-50-100_T1500	100	1500	175	0.0000	29,077	24,841	2,484,092
f-50-100_T1700	100	1700	663	0.0000	17,722	14,308	1,430,836
f-50-100_T1900	100	1900	1458	-1.23×10^6	14,758	12,461	982,915
f-50-100_T2100	100	2100	1827	-164.3	13,170	11,279	27,685
f-50-100_T2500	100	2500	1891	0.0758	10,308	9517	301.8
f-50-100_T2700	100	2700	1893	0.7825	10,160	9301	235.2
f-50-100_T2900	100	2900	1893	0.4454	32,542	24,569	168.5
f-50-300_T1500	300	1500	408	0.0000	29,153	25,784	2,578,429
f-50-300_T1700	300	1700	1215	0.0000	19,389	15,287	1,528,699
f-50-300_T1900	300	1900	1562	-4.90×10^8	14,819	12,232	1,199,387
f-50-300_T2100	300	2100	1869	-973.1	15,249	13,081	280,249
f-50-300_T2300	300	2300	1889	-20.7210	15,358	12,919	4256
f-50-300_T2500	300	2500	1894	0.0550	10,957	9981	2023
f-50-300_T2700	300	2700	1893	0.7815	12,361	10,634	305.1
f-50-300_T2900	300	2900	1895	0.4206	34,821	26,394	183.7
f-50-500_T1500	500	1500	434	0.0000	27,614	23,711	2,371,108
f-50-500_T2100	500	2100	1886	-458.0	14,611	12,635	304,190

continued on next page

Table S12 (continued)

Simulation	$n(O_2)$	T (K)	n_{rows}	R^2	RMSE (molecule·ps)	MAE (molecule·ps)	MAPE (%)
f-50-500_T2300	500	2300	1891	-12.4118	14,605	12,447	7464
f-50-500_T2500	500	2500	1892	-0.5331	12,614	11,310	326.2
f-50-500_T2700	500	2700	1895	0.7511	12,565	11,016	435.0
f-50-500_T2900	500	2900	1893	0.4150	33,077	25,445	256.3
f-50-700_T1700	700	1700	1154	0.0000	19,666	15,251	1,525,142
f-50-700_T1900	700	1900	1812	-11,580.8	17,277	14,220	667,233
f-50-700_T2100	700	2100	1874	-466.2	14,837	12,798	241,117
f-50-700_T2500	700	2500	1894	0.2145	10,044	9118	362.1
f-50-700_T2700	700	2700	1893	0.7894	10,149	9290	306.9
f-50-700_T2900	700	2900	1894	0.5152	29,658	22,955	277.7
f-50-900_T2100	900	2100	1876	-276.8	14,317	12,289	524,470
f-50-900_T2500	900	2500	1884	-0.5807	12,197	11,200	586.3
f-50-900_T2900	900	2900	1893	0.5174	28,560	21,937	380.0
**Repeat: strat44 Case: A Model: physical_RF**							
f-50-1_T1700	1	1700	357	0.0000	0.0	0.0	0.1
f-50-1_T1900	1	1900	1318	0.0000	14.4	5.0	502.7
f-50-1_T2100	1	2100	1816	0.5045	229.4	167.4	45.6
f-50-1_T2500	1	2500	1892	0.8906	2893	1965	14.5
f-50-1_T2700	1	2700	1893	0.9953	1487	590.6	4.5
f-50-1_T2900	1	2900	1892	0.9868	4653	2804	4.4
f-50-100_T1500	100	1500	175	0.0000	0.0	0.0	0.4
f-50-100_T1700	100	1700	663	0.0000	1.1	0.3	33.6
f-50-100_T1900	100	1900	1458	-0.1175	14.0	4.9	21.5
f-50-100_T2100	100	2100	1827	0.3863	802.5	594.3	58.7
f-50-100_T2500	100	2500	1891	0.9610	2118	1531	10.9
f-50-100_T2700	100	2700	1893	0.9968	1224	673.6	6.3
f-50-100_T2900	100	2900	1893	0.9902	4318	2693	4.9
f-50-300_T1500	300	1500	408	0.0000	0.0	0.0	1.3
f-50-300_T1700	300	1700	1215	0.0000	0.2	0.0	3.5
f-50-300_T1900	300	1900	1562	-414.1	13.6	4.8	416.6
f-50-300_T2100	300	2100	1869	-1.5956	787.1	575.0	481.8
f-50-300_T2300	300	2300	1889	0.9436	782.9	620.9	25.0
f-50-300_T2500	300	2500	1894	0.9912	1058	704.1	43.4
f-50-300_T2700	300	2700	1893	0.9355	6716	4870	16.3
f-50-300_T2900	300	2900	1895	0.9946	3357	2466	5.4
f-50-500_T1500	500	1500	434	0.0000	0.2	0.0	4.2

continued on next page

Table S12 (continued)

Simulation	$n(O_2)$	T (K)	n_{rows}	R^2	RMSE (molecule·ps)	MAE (molecule·ps)	MAPE (%)
f-50-500_T2100	500	2100	1886	0.8696	246.2	173.4	74.5
f-50-500_T2300	500	2300	1891	0.9607	790.4	626.7	46.0
f-50-500_T2500	500	2500	1892	0.9699	1767	1383	13.6
f-50-500_T2700	500	2700	1895	0.9876	2805	2339	13.2
f-50-500_T2900	500	2900	1893	0.9905	4222	3361	11.5
f-50-700_T1700	700	1700	1154	0.0000	0.0	0.0	1.9
f-50-700_T1900	700	1900	1812	-1.1415	234.9	171.8	80.2
f-50-700_T2100	700	2100	1874	0.9664	125.8	97.3	38.0
f-50-700_T2500	700	2500	1894	0.9634	2168	1775	15.4
f-50-700_T2700	700	2700	1893	0.9570	4588	3401	12.6
f-50-700_T2900	700	2900	1894	0.9944	3192	2716	11.8
f-50-900_T2100	900	2100	1876	0.8555	326.6	242.2	559.9
f-50-900_T2500	900	2500	1884	0.9175	2787	2258	46.3
f-50-900_T2900	900	2900	1893	0.9920	3667	3098	15.6
**Repeat: strat44 Case: B Model: MLR**							
f-50-1_T1700	1	1700	357	0.0000	20,070	18,239	1,823,904
f-50-1_T1900	1	1900	1318	0.0000	22,555	21,114	2,111,450
f-50-1_T2100	1	2100	1816	-2381.4	15,907	15,426	72,915
f-50-1_T2500	1	2500	1892	0.9286	2337	1891	685.0
f-50-1_T2700	1	2700	1893	0.3464	17,518	15,025	194.3
f-50-1_T2900	1	2900	1892	0.1123	38,113	30,237	121.6
f-50-100_T1500	100	1500	175	0.0000	15,088	12,831	1,283,110
f-50-100_T1700	100	1700	663	0.0000	17,889	16,254	1,625,437
f-50-100_T1900	100	1900	1458	-2.06×10^6	19,080	17,156	1,299,688
f-50-100_T2100	100	2100	1827	-171.0	13,433	12,673	7425
f-50-100_T2500	100	2500	1891	0.9567	2232	1719	299.0
f-50-100_T2700	100	2700	1893	0.9150	6351	4793	117.3
f-50-100_T2900	100	2900	1893	0.3671	34,762	24,375	81.0
f-50-300_T1500	300	1500	408	0.0000	5015	4947	494,713
f-50-300_T1700	300	1700	1215	0.0000	12,273	10,278	1,027,849
f-50-300_T1900	300	1900	1562	-3.17×10^8	11,920	9906	960,848
f-50-300_T2100	300	2100	1869	-1966.7	21,672	17,563	64,316
f-50-300_T2300	300	2300	1889	-34.2506	19,565	16,542	2358
f-50-300_T2500	300	2500	1894	-0.4539	13,591	12,625	8591
f-50-300_T2700	300	2700	1893	0.5430	17,879	15,919	663.6
f-50-300_T2900	300	2900	1895	0.3542	36,763	26,225	109.9

continued on next page

Table S12 (continued)

Simulation	$n(O_2)$	T (K)	n_{rows}	R^2	RMSE (molecule·ps)	MAE (molecule·ps)	MAPE (%)
f-50-500_T1500	500	1500	434	0.0000	7726	6655	665,451
f-50-500_T2100	500	2100	1886	-975.6	21,314	17,802	195,310
f-50-500_T2300	500	2300	1891	-33.1701	23,313	20,795	9469
f-50-500_T2500	500	2500	1892	-2.4340	18,879	17,865	1206
f-50-500_T2700	500	2700	1895	0.5833	16,259	14,046	682.3
f-50-500_T2900	500	2900	1893	0.1477	39,924	29,076	208.1
f-50-700_T1700	700	1700	1154	0.0000	15,685	15,649	1,564,940
f-50-700_T1900	700	1900	1812	-8106.7	14,455	12,548	476,216
f-50-700_T2100	700	2100	1874	-890.1	20,491	18,096	275,505
f-50-700_T2500	700	2500	1894	-1.0719	16,312	15,265	1985
f-50-700_T2700	700	2700	1893	0.5197	15,326	13,318	939.7
f-50-700_T2900	700	2900	1894	-0.0902	44,475	33,399	365.0
f-50-900_T2100	900	2100	1876	-393.1	17,053	12,627	755,597
f-50-900_T2500	900	2500	1884	-1.6294	15,731	14,118	7869
f-50-900_T2900	900	2900	1893	-0.1357	43,812	32,110	1017
**Repeat: strat44 Case: B Model: DT**							
f-50-1_T1700	1	1700	357	0.0000	1.5	0.1	11.1
f-50-1_T1900	1	1900	1318	0.0000	17.5	7.7	769.2
f-50-1_T2100	1	2100	1816	-821.5	9347	1382	215.1
f-50-1_T2500	1	2500	1892	0.2032	7808	6363	83.6
f-50-1_T2700	1	2700	1893	0.2783	18,407	13,604	42.7
f-50-1_T2900	1	2900	1892	0.4509	29,975	22,630	47.6
f-50-100_T1500	100	1500	175	0.0000	11.3	0.9	85.1
f-50-100_T1700	100	1700	663	0.0000	1.1	0.1	13.2
f-50-100_T1900	100	1900	1458	-0.0270	13.5	4.7	17.5
f-50-100_T2100	100	2100	1827	0.0102	1019	739.6	104.6
f-50-100_T2500	100	2500	1891	0.6135	6666	5166	79.2
f-50-100_T2700	100	2700	1893	0.6167	13,487	8490	31.5
f-50-100_T2900	100	2900	1893	0.6089	27,329	20,159	36.5
f-50-300_T1500	300	1500	408	1.0000	0.0	0.0	0.0
f-50-300_T1700	300	1700	1215	0.0000	7.6	0.3	31.0
f-50-300_T1900	300	1900	1562	-9.16×10^8	20,263	5009	394,484
f-50-300_T2100	300	2100	1869	-8112.5	44,007	14,396	1526
f-50-300_T2300	300	2300	1889	-0.6956	4291	2481	54.3
f-50-300_T2500	300	2500	1894	0.0719	10,858	7343	80.5
f-50-300_T2700	300	2700	1893	0.1274	24,704	16,544	54.9

continued on next page

Table S12 (continued)

Simulation	$n(O_2)$	T (K)	n_{rows}	R^2	RMSE (molecule·ps)	MAE (molecule·ps)	MAPE (%)
f-50-300_T2900	300	2900	1895	0.5527	30,595	21,663	34.2
f-50-500_T1500	500	1500	434	1.0000	0.0	0.0	0.0
f-50-500_T2100	500	2100	1886	-428.7	14,138	4801	751.7
f-50-500_T2300	500	2300	1891	-0.6528	5127	3783	107.6
f-50-500_T2500	500	2500	1892	0.8152	4379	3193	40.7
f-50-500_T2700	500	2700	1895	0.2855	21,290	14,883	46.7
f-50-500_T2900	500	2900	1893	0.1940	38,826	26,238	49.5
f-50-700_T1700	700	1700	1154	1.0000	0.0	0.0	0.0
f-50-700_T1900	700	1900	1812	0.3309	131.3	95.7	55.9
f-50-700_T2100	700	2100	1874	-9.0185	2173	1079	113.5
f-50-700_T2500	700	2500	1894	-1.1512	16,621	9112	46.5
f-50-700_T2700	700	2700	1893	-0.5436	27,475	15,502	41.4
f-50-700_T2900	700	2900	1894	0.8194	18,102	10,421	21.3
f-50-900_T2100	900	2100	1876	-1.0273	1223	580.8	622.4
f-50-900_T2500	900	2500	1884	-0.1724	10,504	6051	59.6
f-50-900_T2900	900	2900	1893	0.5986	26,047	17,333	44.0
**Repeat: strat44**	**Case: B**	**Model: RF**					
f-50-1_T1700	1	1700	357	0.0000	731.7	331.5	33,154
f-50-1_T1900	1	1900	1318	0.0000	946.2	557.6	55,763
f-50-1_T2100	1	2100	1816	-805.7	9256	5353	829.5
f-50-1_T2500	1	2500	1892	0.4947	6218	5012	57.6
f-50-1_T2700	1	2700	1893	0.7160	11,547	8338	28.4
f-50-1_T2900	1	2900	1892	0.7170	21,519	14,441	25.3
f-50-100_T1500	100	1500	175	0.0000	3.4	1.7	169.8
f-50-100_T1700	100	1700	663	0.0000	48.2	4.0	395.6
f-50-100_T1900	100	1900	1458	-65,064.8	3388	2278	138,883
f-50-100_T2100	100	2100	1827	-13.3857	3885	1763	136.8
f-50-100_T2500	100	2500	1891	-0.1142	11,318	8403	73.7
f-50-100_T2700	100	2700	1893	0.8287	9017	5788	17.2
f-50-100_T2900	100	2900	1893	0.6855	24,507	17,158	27.7
f-50-300_T1500	300	1500	408	0.0000	9.5	7.1	714.1
f-50-300_T1700	300	1700	1215	0.0000	1534	612.5	61,252
f-50-300_T1900	300	1900	1562	-3.24×10^7	3811	2484	232,190
f-50-300_T2100	300	2100	1869	-3091.1	27,167	16,714	1943
f-50-300_T2300	300	2300	1889	-6.0996	8780	6161	121.1
f-50-300_T2500	300	2500	1894	0.7297	5860	3806	99.3

continued on next page

Table S12 (continued)

Simulation	$n(O_2)$	T (K)	n_{rows}	R^2	RMSE (molecule·ps)	MAE (molecule·ps)	MAPE (%)
f-50-300_T2700	300	2700	1893	0.9239	7295	5743	37.5
f-50-300_T2900	300	2900	1895	0.1453	42,293	29,521	40.4
f-50-500_T1500	500	1500	434	0.0000	4470	3148	314,756
f-50-500_T2100	500	2100	1886	-20.4029	3155	2251	758.0
f-50-500_T2300	500	2300	1891	-0.2455	4451	2475	97.3
f-50-500_T2500	500	2500	1892	-0.0625	10,501	7669	57.8
f-50-500_T2700	500	2700	1895	0.3211	20,752	14,414	43.1
f-50-500_T2900	500	2900	1893	-0.0572	44,466	33,365	53.8
f-50-700_T1700	700	1700	1154	0.0000	1146	904.5	90,450
f-50-700_T1900	700	1900	1812	-16.1603	665.0	485.6	466.3
f-50-700_T2100	700	2100	1874	-2.9933	1372	778.7	237.9
f-50-700_T2500	700	2500	1894	0.7197	5999	3985	27.2
f-50-700_T2700	700	2700	1893	0.7103	11,902	7288	22.2
f-50-700_T2900	700	2900	1894	0.7155	22,720	15,717	26.9
f-50-900_T2100	900	2100	1876	-8.0896	2590	1140	5266
f-50-900_T2500	900	2500	1884	-1.8091	16,260	10,978	79.2
f-50-900_T2900	900	2900	1893	0.8846	13,968	10,173	22.0
**Repeat: strat44 Case: B Model: XGBoost**							
f-50-1_T1700	1	1700	357	0.0000	2208	2021	202,140
f-50-1_T1900	1	1900	1318	0.0000	1751	1434	143,417
f-50-1_T2100	1	2100	1816	-1003.0	10,326	5303	1251
f-50-1_T2500	1	2500	1892	0.3681	6954	5681	123.3
f-50-1_T2700	1	2700	1893	0.5656	14,280	10,391	49.3
f-50-1_T2900	1	2900	1892	0.7951	18,312	13,649	35.2
f-50-100_T1500	100	1500	175	0.0000	2520	1656	165,591
f-50-100_T1700	100	1700	663	0.0000	3195	1738	173,827
f-50-100_T1900	100	1900	1458	-658,062.4	10,773	7116	407,040
f-50-100_T2100	100	2100	1827	-9.9902	3396	1691	159.6
f-50-100_T2500	100	2500	1891	0.2873	9052	6666	63.3
f-50-100_T2700	100	2700	1893	0.8661	7970	5888	65.8
f-50-100_T2900	100	2900	1893	0.7581	21,491	16,728	47.9
f-50-300_T1500	300	1500	408	0.0000	339.3	242.3	24,230
f-50-300_T1700	300	1700	1215	0.0000	7966	5040	503,977
f-50-300_T1900	300	1900	1562	-2.51×10^7	3353	2068	201,824
f-50-300_T2100	300	2100	1869	-1024.4	15,644	8094	4592
f-50-300_T2300	300	2300	1889	-4.3386	7614	5193	210.9

continued on next page

Table S12 (continued)

Simulation	$n(O_2)$	T (K)	n_{rows}	R^2	RMSE (molecule·ps)	MAE (molecule·ps)	MAPE (%)
f-50-300_T2500	300	2500	1894	0.8730	4016	2665	188.2
f-50-300_T2700	300	2700	1893	0.9543	5651	3982	34.0
f-50-300_T2900	300	2900	1895	0.4009	35,409	23,883	36.4
f-50-500_T1500	500	1500	434	0.0000	4283	2761	276,066
f-50-500_T2100	500	2100	1886	-30.4960	3828	2102	8413
f-50-500_T2300	500	2300	1891	0.2040	3558	1984	273.2
f-50-500_T2500	500	2500	1892	0.9051	3139	2295	59.3
f-50-500_T2700	500	2700	1895	0.1644	23,023	15,282	46.3
f-50-500_T2900	500	2900	1893	-0.3179	49,646	34,518	53.2
f-50-700_T1700	700	1700	1154	0.0000	1250	760.1	76,008
f-50-700_T1900	700	1900	1812	-62.9692	1284	817.9	1705
f-50-700_T2100	700	2100	1874	-14.6465	2715	1730	3638
f-50-700_T2500	700	2500	1894	0.7564	5593	3945	46.0
f-50-700_T2700	700	2700	1893	0.9302	5843	3652	30.0
f-50-700_T2900	700	2900	1894	0.6564	24,966	15,099	24.1
f-50-900_T2100	900	2100	1876	-4.5381	2022	1079	4283
f-50-900_T2500	900	2500	1884	-1.0595	13,922	8085	93.2
f-50-900_T2900	900	2900	1893	0.8345	16,726	9920	22.6
**Repeat: strat44**	**Case: B**	**Model: CatBoost**					
f-50-1_T1700	1	1700	357	0.0000	615.2	431.7	43,169
f-50-1_T1900	1	1900	1318	0.0000	2243	1214	121,388
f-50-1_T2100	1	2100	1816	-297.4	5629	3333	1235
f-50-1_T2500	1	2500	1892	-0.0139	8809	5886	79.1
f-50-1_T2700	1	2700	1893	0.9473	4973	3757	25.4
f-50-1_T2900	1	2900	1892	0.6887	22,571	18,558	43.7
f-50-100_T1500	100	1500	175	0.0000	1051	850.5	85,053
f-50-100_T1700	100	1700	663	0.0000	684.3	460.0	46,000
f-50-100_T1900	100	1900	1458	-383,870.0	8228	5199	283,618
f-50-100_T2100	100	2100	1827	-8.8647	3217	2363	251.2
f-50-100_T2500	100	2500	1891	-0.3819	12,605	8860	73.4
f-50-100_T2700	100	2700	1893	0.7702	10,444	8000	39.8
f-50-100_T2900	100	2900	1893	0.7885	20,097	16,812	42.0
f-50-300_T1500	300	1500	408	0.0000	2222	2148	214,775
f-50-300_T1700	300	1700	1215	0.0000	2693	1756	175,632
f-50-300_T1900	300	1900	1562	-9.94×10^6	2111	1347	130,428
f-50-300_T2100	300	2100	1869	-1567.8	19,351	11,836	3651

continued on next page

Table S12 (continued)

Simulation	$n(O_2)$	T (K)	n_{rows}	R^2	RMSE (molecule·ps)	MAE (molecule·ps)	MAPE (%)
f-50-300_T2300	300	2300	1889	-6.4705	9007	5772	142.5
f-50-300_T2500	300	2500	1894	0.9346	2883	1939	127.2
f-50-300_T2700	300	2700	1893	0.9745	4226	3048	38.7
f-50-300_T2900	300	2900	1895	0.1921	41,117	27,873	44.1
f-50-500_T1500	500	1500	434	0.0000	3790	2602	260,232
f-50-500_T2100	500	2100	1886	-14.5411	2689	1792	8476
f-50-500_T2300	500	2300	1891	0.0202	3948	2880	173.8
f-50-500_T2500	500	2500	1892	0.7735	4849	3683	57.7
f-50-500_T2700	500	2700	1895	0.3648	20,073	13,851	45.8
f-50-500_T2900	500	2900	1893	-0.3654	50,533	36,841	57.7
f-50-700_T1700	700	1700	1154	0.0000	1180	810.7	81,073
f-50-700_T1900	700	1900	1812	-56.8107	1221	759.1	3540
f-50-700_T2100	700	2100	1874	-2.5995	1302	862.4	1262
f-50-700_T2500	700	2500	1894	0.3757	8954	5413	44.3
f-50-700_T2700	700	2700	1893	0.7601	10,831	6292	23.1
f-50-700_T2900	700	2900	1894	0.6501	25,195	16,910	27.6
f-50-900_T2100	900	2100	1876	-0.6387	1100	668.4	8445
f-50-900_T2500	900	2500	1884	-4.6826	23,126	13,496	90.7
f-50-900_T2900	900	2900	1893	0.6541	24,181	18,105	36.3
**Repeat: strat44**	**Case: B**	**Model: ANN**					
f-50-1_T1700	1	1700	357	0.0000	1233	638.1	63,812
f-50-1_T1900	1	1900	1318	0.0000	465.1	328.1	32,808
f-50-1_T2100	1	2100	1816	-1.6034	525.8	464.0	1706
f-50-1_T2500	1	2500	1892	0.6318	5308	3515	47.9
f-50-1_T2700	1	2700	1893	0.9558	4556	3344	19.2
f-50-1_T2900	1	2900	1892	0.9726	6693	4362	12.9
f-50-100_T1500	100	1500	175	0.0000	235.5	218.4	21,844
f-50-100_T1700	100	1700	663	0.0000	231.3	203.9	20,393
f-50-100_T1900	100	1900	1458	-153.6	165.1	103.2	6333
f-50-100_T2100	100	2100	1827	-0.4413	1230	1101	680.5
f-50-100_T2500	100	2500	1891	0.9709	1828	1254	53.4
f-50-100_T2700	100	2700	1893	0.8797	7555	4358	15.7
f-50-100_T2900	100	2900	1893	0.9030	13,608	9570	24.7
f-50-300_T1500	300	1500	408	0.0000	299.7	274.5	27,446
f-50-300_T1700	300	1700	1215	0.0000	1415	593.9	59,387
f-50-300_T1900	300	1900	1562	-1.46×10^7	2561	838.9	67,909

continued on next page

Table S12 (continued)

Simulation	$n(O_2)$	T (K)	n_{rows}	R^2	RMSE (molecule·ps)	MAE (molecule·ps)	MAPE (%)
f-50-300_T2100	300	2100	1869	-570.4	11,679	6080	962.5
f-50-300_T2300	300	2300	1889	-1.8123	5526	3624	105.9
f-50-300_T2500	300	2500	1894	0.8813	3883	2573	105.1
f-50-300_T2700	300	2700	1893	0.9493	5955	3646	13.1
f-50-300_T2900	300	2900	1895	0.9731	7496	4810	11.6
f-50-500_T1500	500	1500	434	0.0000	648.0	316.6	31,664
f-50-500_T2100	500	2100	1886	0.3453	551.8	429.6	1053
f-50-500_T2300	500	2300	1891	-0.3599	4651	2301	249.7
f-50-500_T2500	500	2500	1892	0.7677	4910	2670	41.5
f-50-500_T2700	500	2700	1895	0.9751	3974	3088	27.0
f-50-500_T2900	500	2900	1893	0.9846	5372	3927	11.7
f-50-700_T1700	700	1700	1154	0.0000	966.9	650.8	65,076
f-50-700_T1900	700	1900	1812	-42.7708	1062	677.1	4265
f-50-700_T2100	700	2100	1874	0.8696	247.9	178.9	165.1
f-50-700_T2500	700	2500	1894	0.9452	2653	1721	24.6
f-50-700_T2700	700	2700	1893	0.9612	4358	2435	18.2
f-50-700_T2900	700	2900	1894	0.9717	7170	5114	16.1
f-50-900_T2100	900	2100	1876	0.0484	838.0	642.3	4590
f-50-900_T2500	900	2500	1884	0.8772	3400	1789	37.2
f-50-900_T2900	900	2900	1893	0.9907	3970	2986	9.7
**Repeat: strat44 Case: B Model: SVR**							
f-50-1_T1700	1	1700	357	0.0000	405.6	305.3	30,530
f-50-1_T1900	1	1900	1318	0.0000	599.8	578.4	57,840
f-50-1_T2100	1	2100	1816	-178.8	4370	3013	5097
f-50-1_T2500	1	2500	1892	0.8646	3219	2805	141.9
f-50-1_T2700	1	2700	1893	0.2021	19,355	13,261	50.3
f-50-1_T2900	1	2900	1892	-0.2213	44,705	32,455	49.9
f-50-100_T1500	100	1500	175	0.0000	281.8	248.0	24,804
f-50-100_T1700	100	1700	663	0.0000	205.1	125.6	12,563
f-50-100_T1900	100	1900	1458	-2039.8	599.9	351.4	15,038
f-50-100_T2100	100	2100	1827	-34.1032	6069	4310	687.9
f-50-100_T2500	100	2500	1891	0.9052	3301	2349	42.3
f-50-100_T2700	100	2700	1893	0.4634	15,957	9950	30.8
f-50-100_T2900	100	2900	1893	-0.4448	52,524	37,707	51.8
f-50-300_T1500	300	1500	408	0.0000	921.4	905.6	90,559
f-50-300_T1700	300	1700	1215	0.0000	1423	1082	108,216

continued on next page

Table S12 (continued)

Simulation	$n(O_2)$	T (K)	n_{rows}	R^2	RMSE (molecule·ps)	MAE (molecule·ps)	MAPE (%)
f-50-300_T1900	300	1900	1562	-7.55×10^6	1839	1149	108,471
f-50-300_T2100	300	2100	1869	-396.6	9742	7355	3953
f-50-300_T2300	300	2300	1889	-3.8735	7275	5931	201.7
f-50-300_T2500	300	2500	1894	0.8134	4868	3608	184.0
f-50-300_T2700	300	2700	1893	0.2994	22,136	13,855	43.0
f-50-300_T2900	300	2900	1895	-0.5211	56,419	40,393	53.8
f-50-500_T1500	500	1500	434	0.0000	804.7	633.9	63,392
f-50-500_T2100	500	2100	1886	-102.5	6937	4974	4912
f-50-500_T2300	500	2300	1891	-1.8339	6714	5362	376.6
f-50-500_T2500	500	2500	1892	0.8349	4139	3207	58.1
f-50-500_T2700	500	2700	1895	-0.0773	26,142	17,243	46.1
f-50-500_T2900	500	2900	1893	-0.6453	55,473	40,183	57.5
f-50-700_T1700	700	1700	1154	0.0000	486.8	388.3	38,834
f-50-700_T1900	700	1900	1812	-33.7749	946.7	844.9	16,827
f-50-700_T2100	700	2100	1874	-14.2414	2680	1811	4037
f-50-700_T2500	700	2500	1894	0.8555	4307	3015	39.3
f-50-700_T2700	700	2700	1893	0.3782	17,438	10,499	37.3
f-50-700_T2900	700	2900	1894	-0.3013	48,590	33,405	46.7
f-50-900_T2100	900	2100	1876	-5.2880	2154	1913	64,389
f-50-900_T2500	900	2500	1884	0.8235	4075	3096	207.2
f-50-900_T2900	900	2900	1893	-0.2233	45,470	30,427	109.0
**Repeat: strat44**	**Case: B**	**Model: GPR**					
f-50-1_T1700	1	1700	357	0.0000	631.5	420.3	42,030
f-50-1_T1900	1	1900	1318	0.0000	386.1	259.0	25,900
f-50-1_T2100	1	2100	1816	-78.6483	2908	1724	869.1
f-50-1_T2500	1	2500	1892	-0.0581	8998	6551	68.0
f-50-1_T2700	1	2700	1893	0.9738	3504	2257	11.0
f-50-1_T2900	1	2900	1892	0.8832	13,823	10,292	19.5
f-50-100_T1500	100	1500	175	0.0000	173.5	119.3	11,928
f-50-100_T1700	100	1700	663	0.0000	562.7	383.7	38,370
f-50-100_T1900	100	1900	1458	-36,759.7	2546	1564	76,626
f-50-100_T2100	100	2100	1827	-7.0930	2914	2120	291.8
f-50-100_T2500	100	2500	1891	0.9080	3252	1952	23.8
f-50-100_T2700	100	2700	1893	0.8161	9342	6381	19.0
f-50-100_T2900	100	2900	1893	0.9867	5034	3568	10.1
f-50-300_T1500	300	1500	408	0.0000	978.3	900.2	90,022

continued on next page

Table S12 (continued)

Simulation	$n(O_2)$	T (K)	n_{rows}	R^2	RMSE (molecule·ps)	MAE (molecule·ps)	MAPE (%)
f-50-300_T1700	300	1700	1215	0.0000	3649	2491	249,100
f-50-300_T1900	300	1900	1562	-6.18×10^7	5264	4074	397,948
f-50-300_T2100	300	2100	1869	-370.5	9417	6318	2826
f-50-300_T2300	300	2300	1889	0.3844	2585	1943	72.8
f-50-300_T2500	300	2500	1894	0.9216	3157	2006	105.1
f-50-300_T2700	300	2700	1893	0.9793	3801	2799	23.5
f-50-300_T2900	300	2900	1895	0.9227	12,723	9698	18.7
f-50-500_T1500	500	1500	434	0.0000	458.2	344.7	34,468
f-50-500_T2100	500	2100	1886	-16.8339	2880	2035	2054
f-50-500_T2300	500	2300	1891	0.2691	3410	2697	150.2
f-50-500_T2500	500	2500	1892	0.8232	4284	3129	46.3
f-50-500_T2700	500	2700	1895	0.9497	5651	3356	19.3
f-50-500_T2900	500	2900	1893	0.8659	15,838	9508	13.9
f-50-700_T1700	700	1700	1154	0.0000	1354	1025	102,459
f-50-700_T1900	700	1900	1812	-33.7000	945.7	669.4	827.4
f-50-700_T2100	700	2100	1874	-6.0695	1825	1114	653.3
f-50-700_T2500	700	2500	1894	0.8647	4169	2625	19.2
f-50-700_T2700	700	2700	1893	0.9250	6057	3440	11.0
f-50-700_T2900	700	2900	1894	0.9250	11,667	8508	14.3
f-50-900_T2100	900	2100	1876	-1.4031	1332	856.1	3684
f-50-900_T2500	900	2500	1884	-0.6284	12,380	7314	52.9
f-50-900_T2900	900	2900	1893	0.9488	9298	5376	16.6
**Repeat: strat44**	**Case: B**	**Model: persistence**					
f-50-1_T1700	1	1700	357	1.0000	0.0	0.0	0.0
f-50-1_T1900	1	1900	1318	1.0000	0.0	0.0	0.0
f-50-1_T2100	1	2100	1816	0.9631	62.6	54.9	23.7
f-50-1_T2500	1	2500	1892	0.9646	1647	1468	28.5
f-50-1_T2700	1	2700	1893	0.9644	4090	3642	27.3
f-50-1_T2900	1	2900	1892	0.9666	7390	6812	25.5
f-50-100_T1500	100	1500	175	1.0000	0.0	0.0	0.0
f-50-100_T1700	100	1700	663	1.0000	0.0	0.0	0.0
f-50-100_T1900	100	1900	1458	0.5926	8.5	3.1	11.9
f-50-100_T2100	100	2100	1827	0.9585	208.7	176.9	28.0
f-50-100_T2500	100	2500	1891	0.9636	2046	1794	29.2
f-50-100_T2700	100	2700	1893	0.9633	4174	3673	26.9
f-50-100_T2900	100	2900	1893	0.9656	8105	7371	25.8

continued on next page

Table S12 (continued)

Simulation	$n(O_2)$	T (K)	n_{rows}	R^2	RMSE (molecule·ps)	MAE (molecule·ps)	MAPE (%)
f-50-300_T1500	300	1500	408	1.0000	0.0	0.0	0.0
f-50-300_T1700	300	1700	1215	1.0000	0.0	0.0	0.0
f-50-300_T1900	300	1900	1562	0.0667	0.6	0.1	3.4
f-50-300_T2100	300	2100	1869	0.9635	93.4	80.0	24.3
f-50-300_T2300	300	2300	1889	0.9586	670.4	572.4	28.8
f-50-300_T2500	300	2500	1894	0.9609	2228	1892	31.4
f-50-300_T2700	300	2700	1893	0.9631	5080	4441	28.5
f-50-300_T2900	300	2900	1895	0.9655	8494	7665	26.3
f-50-500_T1500	500	1500	434	1.0000	0.0	0.0	0.0
f-50-500_T2100	500	2100	1886	0.9467	157.5	113.8	27.3
f-50-500_T2300	500	2300	1891	0.9589	808.1	663.3	31.3
f-50-500_T2500	500	2500	1892	0.9581	2085	1751	28.4
f-50-500_T2700	500	2700	1895	0.9622	4897	4216	29.2
f-50-500_T2900	500	2900	1893	0.9655	8037	7244	27.0
f-50-700_T1700	700	1700	1154	1.0000	0.0	0.0	0.0
f-50-700_T1900	700	1900	1812	0.9550	34.0	27.5	21.3
f-50-700_T2100	700	2100	1874	0.9562	143.7	115.1	27.1
f-50-700_T2500	700	2500	1894	0.9619	2213	1916	28.9
f-50-700_T2700	700	2700	1893	0.9621	4303	3713	28.5
f-50-700_T2900	700	2900	1894	0.9644	8040	7131	27.6
f-50-900_T2100	900	2100	1876	0.9632	164.8	133.9	23.0
f-50-900_T2500	900	2500	1884	0.9600	1940	1651	31.2
f-50-900_T2900	900	2900	1893	0.9640	7798	6850	28.8
**Repeat: strat44 Case: B Model: physical_MLR**							
f-50-1_T1700	1	1700	357	0.0000	21,123	17,083	1,708,337
f-50-1_T1900	1	1900	1318	0.0000	14,850	12,648	1,264,836
f-50-1_T2100	1	2100	1816	-1844.0	13,998	11,869	135,795
f-50-1_T2500	1	2500	1892	-1.6220	14,165	12,587	462.6
f-50-1_T2700	1	2700	1893	0.8207	9174	8316	230.6
f-50-1_T2900	1	2900	1892	0.4452	30,131	23,328	180.9
f-50-100_T1500	100	1500	175	0.0000	29,077	24,841	2,484,092
f-50-100_T1700	100	1700	663	0.0000	17,722	14,308	1,430,836
f-50-100_T1900	100	1900	1458	-1.23×10^6	14,758	12,461	982,915
f-50-100_T2100	100	2100	1827	-164.3	13,170	11,279	27,685
f-50-100_T2500	100	2500	1891	0.0758	10,308	9517	301.8
f-50-100_T2700	100	2700	1893	0.7825	10,160	9301	235.2

continued on next page

Table S12 (continued)

Simulation	$n(O_2)$	T (K)	n_{rows}	R^2	RMSE (molecule·ps)	MAE (molecule·ps)	MAPE (%)
f-50-100_T2900	100	2900	1893	0.4454	32,542	24,569	168.5
f-50-300_T1500	300	1500	408	0.0000	29,153	25,784	2,578,429
f-50-300_T1700	300	1700	1215	0.0000	19,389	15,287	1,528,699
f-50-300_T1900	300	1900	1562	-4.90×10^8	14,819	12,232	1,199,387
f-50-300_T2100	300	2100	1869	-973.1	15,249	13,081	280,249
f-50-300_T2300	300	2300	1889	-20.7210	15,358	12,919	4256
f-50-300_T2500	300	2500	1894	0.0550	10,957	9981	2023
f-50-300_T2700	300	2700	1893	0.7815	12,361	10,634	305.1
f-50-300_T2900	300	2900	1895	0.4206	34,821	26,394	183.7
f-50-500_T1500	500	1500	434	0.0000	27,614	23,711	2,371,108
f-50-500_T2100	500	2100	1886	-458.0	14,611	12,635	304,190
f-50-500_T2300	500	2300	1891	-12.4118	14,605	12,447	7464
f-50-500_T2500	500	2500	1892	-0.5331	12,614	11,310	326.2
f-50-500_T2700	500	2700	1895	0.7511	12,565	11,016	435.0
f-50-500_T2900	500	2900	1893	0.4150	33,077	25,445	256.3
f-50-700_T1700	700	1700	1154	0.0000	19,666	15,251	1,525,142
f-50-700_T1900	700	1900	1812	-11,580.8	17,277	14,220	667,233
f-50-700_T2100	700	2100	1874	-466.2	14,837	12,798	241,117
f-50-700_T2500	700	2500	1894	0.2145	10,044	9118	362.1
f-50-700_T2700	700	2700	1893	0.7894	10,149	9290	306.9
f-50-700_T2900	700	2900	1894	0.5152	29,658	22,955	277.7
f-50-900_T2100	900	2100	1876	-276.8	14,317	12,289	524,470
f-50-900_T2500	900	2500	1884	-0.5807	12,197	11,200	586.3
f-50-900_T2900	900	2900	1893	0.5174	28,560	21,937	380.0
**Repeat: strat44 Case: B Model: physical_RF**							
f-50-1_T1700	1	1700	357	0.0000	43.1	16.5	1650
f-50-1_T1900	1	1900	1318	0.0000	371.2	258.6	25,861
f-50-1_T2100	1	2100	1816	-0.0842	339.3	256.1	80.1
f-50-1_T2500	1	2500	1892	-0.0046	8768	6992	334.1
f-50-1_T2700	1	2700	1893	0.6478	12,858	8816	85.9
f-50-1_T2900	1	2900	1892	0.7994	18,116	14,562	44.8
f-50-100_T1500	100	1500	175	0.0000	65.2	33.9	3389
f-50-100_T1700	100	1700	663	0.0000	67.9	30.9	3086
f-50-100_T1900	100	1900	1458	-2169.4	618.7	407.9	28,259
f-50-100_T2100	100	2100	1827	-0.6567	1318	985.1	88.6
f-50-100_T2500	100	2500	1891	0.9713	1816	1563	252.8

continued on next page

Table S12 (continued)

Simulation	$n(O_2)$	T (K)	n_{rows}	R^2	RMSE (molecule·ps)	MAE (molecule·ps)	MAPE (%)
f-50-100_T2700	100	2700	1893	0.9687	3853	2814	15.1
f-50-100_T2900	100	2900	1893	0.7680	21,049	15,991	29.0
f-50-300_T1500	300	1500	408	0.0000	39.0	8.4	836.2
f-50-300_T1700	300	1700	1215	0.0000	110.5	68.4	6836
f-50-300_T1900	300	1900	1562	-783,706.1	592.7	472.6	45,723
f-50-300_T2100	300	2100	1869	-5.8965	1283	867.6	2203
f-50-300_T2300	300	2300	1889	0.2234	2904	2073	184.1
f-50-300_T2500	300	2500	1894	0.6730	6445	4564	166.8
f-50-300_T2700	300	2700	1893	0.9842	3322	2390	31.3
f-50-300_T2900	300	2900	1895	0.9150	13,333	10,186	20.4
f-50-500_T1500	500	1500	434	0.0000	3441	2601	260,091
f-50-500_T2100	500	2100	1886	-13.7021	2615	1970	1506
f-50-500_T2300	500	2300	1891	0.4769	2885	2051	85.5
f-50-500_T2500	500	2500	1892	0.8804	3523	2655	47.0
f-50-500_T2700	500	2700	1895	0.9956	1680	925.5	19.6
f-50-500_T2900	500	2900	1893	0.9945	3200	2213	12.7
f-50-700_T1700	700	1700	1154	0.0000	1003	803.1	80,313
f-50-700_T1900	700	1900	1812	-18.2832	705.0	498.6	680.8
f-50-700_T2100	700	2100	1874	0.7205	362.9	264.4	137.6
f-50-700_T2500	700	2500	1894	0.9746	1807	1418	151.0
f-50-700_T2700	700	2700	1893	0.2297	19,409	15,100	146.8
f-50-700_T2900	700	2900	1894	0.8672	15,525	11,711	30.4
f-50-900_T2100	900	2100	1876	-98.8832	8585	6558	15,395
f-50-900_T2500	900	2500	1884	-0.5094	11,919	8202	236.4
f-50-900_T2900	900	2900	1893	0.7724	19,612	14,997	42.1
**Repeat: strat45 Case: A Model: MLR**							
f-50-1_T1700	1	1700	357	0.0000	598.7	497.3	49,734
f-50-1_T1900	1	1900	1318	0.0000	77.9	64.3	6434
f-50-1_T2100	1	2100	1816	-0.6170	414.4	393.4	4619
f-50-1_T2500	1	2500	1892	0.9959	561.9	508.3	129.2
f-50-1_T2700	1	2700	1893	0.9964	1307	895.2	11.4
f-50-1_T2900	1	2900	1892	0.9982	1725	1560	14.4
f-50-100_T1500	100	1500	175	0.0000	542.2	459.3	45,932
f-50-100_T1700	100	1700	663	0.0000	500.6	497.2	49,716
f-50-100_T1900	100	1900	1458	-1294.2	477.9	401.2	31,867
f-50-100_T2100	100	2100	1827	0.9100	307.3	244.4	701.6

continued on next page

Table S12 (continued)

Simulation	$n(O_2)$	T (K)	n_{rows}	R^2	RMSE (molecule·ps)	MAE (molecule·ps)	MAPE (%)
f-50-100_T2500	100	2500	1891	0.9980	478.1	382.4	113.5
f-50-100_T2700	100	2700	1893	0.9994	537.7	447.4	27.8
f-50-100_T2900	100	2900	1893	0.9988	1545	1417	12.5
f-50-300_T1500	300	1500	408	0.0000	138.3	113.9	11,388
f-50-300_T1700	300	1700	1215	0.0000	564.1	558.6	55,863
f-50-300_T1900	300	1900	1562	-22,033.8	99.4	83.7	8296
f-50-300_T2100	300	2100	1869	0.3899	381.6	344.7	10,098
f-50-300_T2300	300	2300	1889	0.9528	715.6	706.1	311.3
f-50-300_T2500	300	2500	1894	0.9969	626.7	561.9	650.7
f-50-300_T2700	300	2700	1893	0.9997	464.0	421.0	19.6
f-50-300_T2900	300	2900	1895	0.9993	1230	1091	13.4
f-50-500_T1500	500	1500	434	0.0000	1337	1335	133,545
f-50-500_T2100	500	2100	1886	0.8205	289.0	273.2	4154
f-50-500_T2300	500	2300	1891	0.9740	642.8	631.0	304.0
f-50-500_T2500	500	2500	1892	0.9941	783.9	767.9	82.9
f-50-500_T2700	500	2700	1895	0.9995	570.9	454.4	62.5
f-50-500_T2900	500	2900	1893	0.9993	1116	968.4	20.2
f-50-700_T1700	700	1700	1154	0.0000	796.0	785.2	78,517
f-50-700_T1900	700	1900	1812	-24.7390	814.5	738.1	27,403
f-50-700_T2100	700	2100	1874	0.9082	208.0	168.7	1281
f-50-700_T2500	700	2500	1894	0.9966	665.5	454.0	147.8
f-50-700_T2700	700	2700	1893	0.9991	668.0	536.8	51.7
f-50-700_T2900	700	2900	1894	0.9989	1432	1192	6.0
f-50-900_T2100	900	2100	1876	-0.1819	933.9	835.5	37,678
f-50-900_T2500	900	2500	1884	0.9976	476.0	430.0	163.9
f-50-900_T2900	900	2900	1893	0.9991	1212	1056	48.8
**Repeat: strat45 Case: A Model: DT**							
f-50-1_T1700	1	1700	357	0.0000	5.9	1.6	156.8
f-50-1_T1900	1	1900	1318	0.0000	31.0	14.6	1455
f-50-1_T2100	1	2100	1816	0.8422	129.5	92.2	121.8
f-50-1_T2500	1	2500	1892	0.9982	375.7	282.9	6.6
f-50-1_T2700	1	2700	1893	0.9998	312.5	243.4	5.9
f-50-1_T2900	1	2900	1892	0.9994	970.9	808.2	5.0
f-50-100_T1500	100	1500	175	0.0000	1.2	0.9	93.7
f-50-100_T1700	100	1700	663	0.0000	4.6	2.8	281.4
f-50-100_T1900	100	1900	1458	-0.0620	13.7	5.4	102.1

continued on next page

Table S12 (continued)

Simulation	$n(O_2)$	T (K)	n_{rows}	R^2	RMSE (molecule·ps)	MAE (molecule·ps)	MAPE (%)
f-50-100_T2100	100	2100	1827	0.9441	242.1	163.3	54.5
f-50-100_T2500	100	2500	1891	0.9993	289.5	213.8	11.2
f-50-100_T2700	100	2700	1893	0.9997	374.1	279.6	5.3
f-50-100_T2900	100	2900	1893	0.9997	712.3	445.3	4.6
f-50-300_T1500	300	1500	408	0.0000	1.5	1.5	149.1
f-50-300_T1700	300	1700	1215	0.0000	5.1	3.0	302.5
f-50-300_T1900	300	1900	1562	-84.6766	6.2	3.1	307.6
f-50-300_T2100	300	2100	1869	0.9714	82.7	58.5	60.6
f-50-300_T2300	300	2300	1889	0.9940	255.0	213.8	22.3
f-50-300_T2500	300	2500	1894	0.9997	196.9	142.7	35.6
f-50-300_T2700	300	2700	1893	0.9996	527.5	365.7	9.1
f-50-300_T2900	300	2900	1895	0.9997	802.9	446.5	3.9
f-50-500_T1500	500	1500	434	0.0000	2.8	2.8	279.2
f-50-500_T2100	500	2100	1886	0.9899	68.4	49.8	122.4
f-50-500_T2300	500	2300	1891	0.9917	362.6	278.9	44.2
f-50-500_T2500	500	2500	1892	0.9997	187.1	148.5	7.0
f-50-500_T2700	500	2700	1895	0.9996	488.3	333.1	8.5
f-50-500_T2900	500	2900	1893	0.9999	335.4	281.0	3.3
f-50-700_T1700	700	1700	1154	0.0000	1.5	1.5	149.1
f-50-700_T1900	700	1900	1812	0.8994	50.9	36.8	60.5
f-50-700_T2100	700	2100	1874	0.9949	49.0	38.1	74.1
f-50-700_T2500	700	2500	1894	0.9997	201.3	137.1	8.4
f-50-700_T2700	700	2700	1893	0.9997	408.6	336.6	7.1
f-50-700_T2900	700	2900	1894	1.0000	258.5	207.0	3.4
f-50-900_T2100	900	2100	1876	0.9657	159.1	89.8	738.8
f-50-900_T2500	900	2500	1884	0.9994	246.0	155.8	26.3
f-50-900_T2900	900	2900	1893	0.9999	331.7	246.9	5.8
**Repeat: strat45 Case: A Model: RF**							
f-50-1_T1700	1	1700	357	0.0000	12.4	2.8	283.5
f-50-1_T1900	1	1900	1318	0.0000	17.8	9.6	960.4
f-50-1_T2100	1	2100	1816	0.9476	74.6	56.2	84.4
f-50-1_T2500	1	2500	1892	0.9983	360.1	278.7	8.0
f-50-1_T2700	1	2700	1893	0.9998	325.4	252.6	3.6
f-50-1_T2900	1	2900	1892	0.9996	837.2	665.3	4.4
f-50-100_T1500	100	1500	175	0.0000	2.4	1.5	149.0
f-50-100_T1700	100	1700	663	0.0000	3.9	2.4	235.7

continued on next page

Table S12 (continued)

Simulation	$n(O_2)$	T (K)	n_{rows}	R^2	RMSE (molecule·ps)	MAE (molecule·ps)	MAPE (%)
f-50-100_T1900	100	1900	1458	-0.0829	13.8	5.7	137.6
f-50-100_T2100	100	2100	1827	0.9806	142.5	103.2	47.6
f-50-100_T2500	100	2500	1891	0.9998	167.6	116.8	8.2
f-50-100_T2700	100	2700	1893	0.9999	224.4	187.2	4.6
f-50-100_T2900	100	2900	1893	0.9999	485.0	347.0	4.0
f-50-300_T1500	300	1500	408	0.0000	1.7	1.7	171.9
f-50-300_T1700	300	1700	1215	0.0000	4.8	2.9	288.7
f-50-300_T1900	300	1900	1562	-2355.2	32.5	18.7	1785
f-50-300_T2100	300	2100	1869	0.9789	71.0	52.1	78.0
f-50-300_T2300	300	2300	1889	0.9952	228.1	206.9	20.0
f-50-300_T2500	300	2500	1894	0.9998	171.4	141.3	34.6
f-50-300_T2700	300	2700	1893	0.9997	437.1	295.9	6.6
f-50-300_T2900	300	2900	1895	0.9997	816.5	442.7	3.5
f-50-500_T1500	500	1500	434	0.0000	2.7	2.7	266.6
f-50-500_T2100	500	2100	1886	0.9933	56.0	43.4	121.2
f-50-500_T2300	500	2300	1891	0.9946	292.2	239.7	40.2
f-50-500_T2500	500	2500	1892	0.9998	156.6	135.3	6.4
f-50-500_T2700	500	2700	1895	0.9997	418.8	305.4	7.7
f-50-500_T2900	500	2900	1893	1.0000	263.6	236.3	2.9
f-50-700_T1700	700	1700	1154	0.0000	1.7	1.7	171.8
f-50-700_T1900	700	1900	1812	0.9183	45.9	32.8	72.1
f-50-700_T2100	700	2100	1874	0.9976	33.6	26.1	94.5
f-50-700_T2500	700	2500	1894	0.9999	138.1	87.7	7.0
f-50-700_T2700	700	2700	1893	0.9998	333.0	281.0	5.6
f-50-700_T2900	700	2900	1894	1.0000	211.3	165.8	3.2
f-50-900_T2100	900	2100	1876	0.9760	133.0	79.5	743.8
f-50-900_T2500	900	2500	1884	0.9999	106.5	76.3	24.1
f-50-900_T2900	900	2900	1893	1.0000	278.1	217.6	5.0
**Repeat: strat45 Case: A Model: XGBoost**							
f-50-1_T1700	1	1700	357	0.0000	11.9	2.7	273.9
f-50-1_T1900	1	1900	1318	0.0000	5.5	2.6	264.2
f-50-1_T2100	1	2100	1816	0.8973	104.4	82.6	86.2
f-50-1_T2500	1	2500	1892	0.9984	349.9	306.8	15.0
f-50-1_T2700	1	2700	1893	0.9997	365.8	272.4	4.6
f-50-1_T2900	1	2900	1892	0.9985	1567	801.7	4.1
f-50-100_T1500	100	1500	175	0.0000	3.7	1.6	156.2

continued on next page

Table S12 (continued)

Simulation	$n(O_2)$	T (K)	n_{rows}	R^2	RMSE (molecule·ps)	MAE (molecule·ps)	MAPE (%)
f-50-100_T1700	100	1700	663	0.0000	1.5	1.3	129.9
f-50-100_T1900	100	1900	1458	0.7703	6.4	3.7	236.2
f-50-100_T2100	100	2100	1827	0.9952	71.3	50.8	35.0
f-50-100_T2500	100	2500	1891	0.9996	219.0	167.7	7.0
f-50-100_T2700	100	2700	1893	0.9997	378.5	292.3	3.7
f-50-100_T2900	100	2900	1893	0.9991	1311	843.3	4.1
f-50-300_T1500	300	1500	408	0.0000	0.8	0.7	73.8
f-50-300_T1700	300	1700	1215	0.0000	2.2	1.4	139.7
f-50-300_T1900	300	1900	1562	-3782.6	41.2	25.7	2441
f-50-300_T2100	300	2100	1869	0.9384	121.2	83.1	73.4
f-50-300_T2300	300	2300	1889	0.9948	237.6	205.0	13.6
f-50-300_T2500	300	2500	1894	0.9998	157.0	114.1	39.5
f-50-300_T2700	300	2700	1893	0.9997	466.9	361.7	5.3
f-50-300_T2900	300	2900	1895	0.9985	1768	979.9	3.5
f-50-500_T1500	500	1500	434	0.0000	1.4	0.9	91.7
f-50-500_T2100	500	2100	1886	0.9962	42.1	33.3	255.3
f-50-500_T2300	500	2300	1891	0.9981	174.9	127.7	28.3
f-50-500_T2500	500	2500	1892	0.9997	176.4	147.6	5.7
f-50-500_T2700	500	2700	1895	0.9997	416.6	285.6	9.2
f-50-500_T2900	500	2900	1893	0.9994	1030	540.5	2.7
f-50-700_T1700	700	1700	1154	0.0000	11.8	8.7	868.2
f-50-700_T1900	700	1900	1812	0.9674	29.0	22.2	467.5
f-50-700_T2100	700	2100	1874	0.9977	32.6	23.0	72.2
f-50-700_T2500	700	2500	1894	0.9998	165.0	128.3	5.0
f-50-700_T2700	700	2700	1893	0.9996	466.5	368.6	4.8
f-50-700_T2900	700	2900	1894	0.9998	667.2	414.2	2.6
f-50-900_T2100	900	2100	1876	0.9552	181.9	158.8	1147
f-50-900_T2500	900	2500	1884	0.9995	205.9	164.1	20.5
f-50-900_T2900	900	2900	1893	0.9994	1029	821.7	6.1
**Repeat: strat45**	**Case: A**	**Model: CatBoost**					
f-50-1_T1700	1	1700	357	0.0000	34.7	28.1	2808
f-50-1_T1900	1	1900	1318	0.0000	54.6	48.5	4847
f-50-1_T2100	1	2100	1816	0.7208	172.2	127.6	227.4
f-50-1_T2500	1	2500	1892	0.9983	364.7	263.4	10.7
f-50-1_T2700	1	2700	1893	0.9988	738.1	586.5	5.4
f-50-1_T2900	1	2900	1892	0.9975	2029	1444	6.2

continued on next page

Table S12 (continued)

Simulation	$n(O_2)$	T (K)	n_{rows}	R^2	RMSE (molecule·ps)	MAE (molecule·ps)	MAPE (%)
f-50-100_T1500	100	1500	175	0.0000	29.3	25.2	2516
f-50-100_T1700	100	1700	663	0.0000	30.1	25.0	2501
f-50-100_T1900	100	1900	1458	-7.2843	38.2	32.7	2712
f-50-100_T2100	100	2100	1827	0.9369	257.3	207.7	55.8
f-50-100_T2500	100	2500	1891	0.9993	287.5	219.0	8.2
f-50-100_T2700	100	2700	1893	0.9990	691.6	573.5	6.9
f-50-100_T2900	100	2900	1893	0.9989	1459	1159	6.0
f-50-300_T1500	300	1500	408	0.0000	21.3	17.5	1752
f-50-300_T1700	300	1700	1215	0.0000	30.0	24.1	2406
f-50-300_T1900	300	1900	1562	-10,680.2	69.2	55.0	5336
f-50-300_T2100	300	2100	1869	0.9496	109.6	85.3	209.0
f-50-300_T2300	300	2300	1889	0.9892	342.6	278.4	17.7
f-50-300_T2500	300	2500	1894	0.9995	258.0	192.8	18.4
f-50-300_T2700	300	2700	1893	0.9990	833.6	551.4	6.2
f-50-300_T2900	300	2900	1895	0.9978	2166	1290	4.7
f-50-500_T1500	500	1500	434	0.0000	89.4	81.1	8114
f-50-500_T2100	500	2100	1886	0.9788	99.3	80.8	406.5
f-50-500_T2300	500	2300	1891	0.9946	294.0	232.5	65.5
f-50-500_T2500	500	2500	1892	0.9990	317.0	243.5	6.5
f-50-500_T2700	500	2700	1895	0.9996	533.5	372.6	10.9
f-50-500_T2900	500	2900	1893	0.9996	904.6	706.3	3.7
f-50-700_T1700	700	1700	1154	0.0000	93.0	88.2	8820
f-50-700_T1900	700	1900	1812	0.6862	89.9	67.1	850.2
f-50-700_T2100	700	2100	1874	0.9719	115.2	79.7	433.4
f-50-700_T2500	700	2500	1894	0.9989	379.3	286.6	14.3
f-50-700_T2700	700	2700	1893	0.9993	566.0	416.8	5.7
f-50-700_T2900	700	2900	1894	0.9996	851.4	682.8	5.9
f-50-900_T2100	900	2100	1876	0.9351	218.9	177.7	7150
f-50-900_T2500	900	2500	1884	0.9953	664.1	529.6	42.0
f-50-900_T2900	900	2900	1893	0.9992	1159	934.8	6.1
**Repeat: strat45 Case: A Model: ANN**							
f-50-1_T1700	1	1700	357	0.0000	115.9	95.3	9528
f-50-1_T1900	1	1900	1318	0.0000	87.4	67.5	6746
f-50-1_T2100	1	2100	1816	-0.2589	365.7	225.0	529.4
f-50-1_T2500	1	2500	1892	0.9954	594.8	541.5	52.3
f-50-1_T2700	1	2700	1893	0.9999	234.2	192.4	3.4

continued on next page

Table S12 (continued)

Simulation	$n(O_2)$	T (K)	n_{rows}	R^2	RMSE (molecule·ps)	MAE (molecule·ps)	MAPE (%)
f-50-1_T2900	1	2900	1892	0.9993	1045	833.2	12.1
f-50-100_T1500	100	1500	175	0.0000	53.0	38.8	3879
f-50-100_T1700	100	1700	663	0.0000	195.0	150.7	15,066
f-50-100_T1900	100	1900	1458	-12.2780	48.4	34.9	2108
f-50-100_T2100	100	2100	1827	0.4376	768.1	636.0	90.2
f-50-100_T2500	100	2500	1891	0.9974	542.3	444.6	10.0
f-50-100_T2700	100	2700	1893	0.9992	622.5	470.5	4.7
f-50-100_T2900	100	2900	1893	0.9996	886.5	721.8	6.1
f-50-300_T1500	300	1500	408	0.0000	157.5	147.6	14,759
f-50-300_T1700	300	1700	1215	0.0000	80.3	62.2	6215
f-50-300_T1900	300	1900	1562	-12,298.4	74.2	59.1	5561
f-50-300_T2100	300	2100	1869	0.2148	432.9	301.3	524.0
f-50-300_T2300	300	2300	1889	0.8697	1190	886.8	28.4
f-50-300_T2500	300	2500	1894	0.9963	685.7	523.1	32.6
f-50-300_T2700	300	2700	1893	0.9996	553.8	408.2	4.3
f-50-300_T2900	300	2900	1895	0.9998	620.7	494.2	4.8
f-50-500_T1500	500	1500	434	0.0000	58.3	41.8	4181
f-50-500_T2100	500	2100	1886	0.7986	306.1	232.6	907.0
f-50-500_T2300	500	2300	1891	0.9897	403.8	298.4	51.6
f-50-500_T2500	500	2500	1892	0.9980	450.4	322.1	14.4
f-50-500_T2700	500	2700	1895	0.9996	519.3	405.4	6.5
f-50-500_T2900	500	2900	1893	0.9996	911.1	703.9	4.8
f-50-700_T1700	700	1700	1154	0.0000	464.9	417.5	41,751
f-50-700_T1900	700	1900	1812	-3.3776	335.9	308.6	7346
f-50-700_T2100	700	2100	1874	0.9174	197.3	118.6	545.7
f-50-700_T2500	700	2500	1894	0.9996	228.7	192.2	20.9
f-50-700_T2700	700	2700	1893	0.9977	1062	764.1	11.8
f-50-700_T2900	700	2900	1894	0.9997	733.5	619.5	5.1
f-50-900_T2100	900	2100	1876	0.9051	264.6	209.6	3390
f-50-900_T2500	900	2500	1884	0.9985	380.8	300.2	46.6
f-50-900_T2900	900	2900	1893	0.9998	643.6	570.2	5.9
**Repeat: strat45**	**Case: A**	**Model: SVR**					
f-50-1_T1700	1	1700	357	0.0000	1188	1026	102,608
f-50-1_T1900	1	1900	1318	0.0000	1183	1117	111,688
f-50-1_T2100	1	2100	1816	-22.2912	1573	1115	6532
f-50-1_T2500	1	2500	1892	0.8731	3117	2562	167.5

continued on next page

Table S12 (continued)

Simulation	$n(O_2)$	T (K)	n_{rows}	R^2	RMSE (molecule·ps)	MAE (molecule·ps)	MAPE (%)
f-50-1_T2700	1	2700	1893	0.6943	11,980	7050	56.1
f-50-1_T2900	1	2900	1892	-0.0516	41,482	28,786	55.3
f-50-100_T1500	100	1500	175	0.0000	686.6	459.0	45,895
f-50-100_T1700	100	1700	663	0.0000	610.0	470.9	47,092
f-50-100_T1900	100	1900	1458	-1703.9	548.4	383.7	22,098
f-50-100_T2100	100	2100	1827	-0.5767	1286	983.9	723.0
f-50-100_T2500	100	2500	1891	0.9732	1755	1120	81.2
f-50-100_T2700	100	2700	1893	0.8999	6891	3936	35.5
f-50-100_T2900	100	2900	1893	-0.0203	44,138	28,927	45.6
f-50-300_T1500	300	1500	408	0.0000	858.4	707.3	70,731
f-50-300_T1700	300	1700	1215	0.0000	435.5	350.9	35,091
f-50-300_T1900	300	1900	1562	-342,448.1	391.8	267.7	25,206
f-50-300_T2100	300	2100	1869	-14.7275	1938	1352	6013
f-50-300_T2300	300	2300	1889	0.7391	1683	1447	270.1
f-50-300_T2500	300	2500	1894	0.9611	2223	1436	649.0
f-50-300_T2700	300	2700	1893	0.8004	11,816	6933	25.4
f-50-300_T2900	300	2900	1895	0.2384	39,921	24,847	43.7
f-50-500_T1500	500	1500	434	0.0000	413.0	367.1	36,711
f-50-500_T2100	500	2100	1886	-2.7553	1322	929.8	3853
f-50-500_T2300	500	2300	1891	0.7474	2004	1621	435.2
f-50-500_T2500	500	2500	1892	0.9246	2797	1710	75.3
f-50-500_T2700	500	2700	1895	0.8229	10,600	6513	80.3
f-50-500_T2900	500	2900	1893	0.2762	36,792	23,915	56.0
f-50-700_T1700	700	1700	1154	0.0000	4428	4075	407,546
f-50-700_T1900	700	1900	1812	-319.9	2876	2600	30,048
f-50-700_T2100	700	2100	1874	-2.0670	1202	838.0	5243
f-50-700_T2500	700	2500	1894	0.8169	4849	3535	163.8
f-50-700_T2700	700	2700	1893	0.7287	11,519	7210	85.8
f-50-700_T2900	700	2900	1894	0.1898	38,339	25,614	39.3
f-50-900_T2100	900	2100	1876	-21.7311	4096	3558	50,567
f-50-900_T2500	900	2500	1884	0.8987	3088	2352	523.1
f-50-900_T2900	900	2900	1893	-0.0272	41,666	28,006	149.3
**Repeat: strat45 Case: A Model: GPR**							
f-50-1_T1700	1	1700	357	0.0000	20.1	13.4	1337
f-50-1_T1900	1	1900	1318	0.0000	7.7	6.8	675.3
f-50-1_T2100	1	2100	1816	0.8585	122.6	103.5	115.2

continued on next page

Table S12 (continued)

Simulation	$n(O_2)$	T (K)	n_{rows}	R^2	RMSE (molecule·ps)	MAE (molecule·ps)	MAPE (%)
f-50-1_T2500	1	2500	1892	0.9995	190.8	143.4	15.8
f-50-1_T2700	1	2700	1893	0.9999	169.0	138.8	3.1
f-50-1_T2900	1	2900	1892	0.9999	416.8	326.3	2.9
f-50-100_T1500	100	1500	175	0.0000	12.4	6.9	686.8
f-50-100_T1700	100	1700	663	0.0000	8.7	8.3	825.1
f-50-100_T1900	100	1900	1458	-1.2880	20.1	17.3	1325
f-50-100_T2100	100	2100	1827	0.9774	153.9	129.2	28.8
f-50-100_T2500	100	2500	1891	0.9999	76.5	63.7	5.7
f-50-100_T2700	100	2700	1893	0.9999	167.9	133.9	2.7
f-50-100_T2900	100	2900	1893	0.9999	385.4	332.6	3.1
f-50-300_T1500	300	1500	408	0.0000	7.1	5.1	510.3
f-50-300_T1700	300	1700	1215	0.0000	15.6	13.9	1389
f-50-300_T1900	300	1900	1562	-3700.0	40.7	34.4	3428
f-50-300_T2100	300	2100	1869	0.9761	75.6	61.8	245.5
f-50-300_T2300	300	2300	1889	0.9986	121.5	88.0	13.8
f-50-300_T2500	300	2500	1894	0.9998	141.9	120.0	55.5
f-50-300_T2700	300	2700	1893	0.9999	237.8	201.9	4.5
f-50-300_T2900	300	2900	1895	1.0000	201.2	147.9	1.8
f-50-500_T1500	500	1500	434	0.0000	18.6	15.1	1513
f-50-500_T2100	500	2100	1886	0.9889	71.8	53.9	168.7
f-50-500_T2300	500	2300	1891	0.9947	291.2	207.9	38.8
f-50-500_T2500	500	2500	1892	0.9998	141.9	106.7	6.8
f-50-500_T2700	500	2700	1895	1.0000	177.5	153.2	6.0
f-50-500_T2900	500	2900	1893	0.9999	362.4	334.1	2.3
f-50-700_T1700	700	1700	1154	0.0000	245.0	219.8	21,978
f-50-700_T1900	700	1900	1812	0.4620	117.7	103.3	2752
f-50-700_T2100	700	2100	1874	0.9941	52.6	35.3	97.7
f-50-700_T2500	700	2500	1894	0.9994	267.9	233.1	17.5
f-50-700_T2700	700	2700	1893	0.9998	284.2	214.8	3.5
f-50-700_T2900	700	2900	1894	1.0000	297.5	250.5	3.2
f-50-900_T2100	900	2100	1876	0.7264	449.3	315.5	21,016
f-50-900_T2500	900	2500	1884	0.9979	441.8	380.5	9.1
f-50-900_T2900	900	2900	1893	0.9995	932.9	849.6	9.2
**Repeat: strat45**		**Case: A**		**Model: persistence**			
f-50-1_T1700	1	1700	357	1.0000	0.0	0.0	0.0
f-50-1_T1900	1	1900	1318	1.0000	0.0	0.0	0.0

continued on next page

Table S12 (continued)

Simulation	$n(O_2)$	T (K)	n_{rows}	R^2	RMSE (molecule·ps)	MAE (molecule·ps)	MAPE (%)
f-50-1_T2100	1	2100	1816	0.9631	62.6	54.9	23.7
f-50-1_T2500	1	2500	1892	0.9646	1647	1468	28.5
f-50-1_T2700	1	2700	1893	0.9644	4090	3642	27.3
f-50-1_T2900	1	2900	1892	0.9666	7390	6812	25.5
f-50-100_T1500	100	1500	175	1.0000	0.0	0.0	0.0
f-50-100_T1700	100	1700	663	1.0000	0.0	0.0	0.0
f-50-100_T1900	100	1900	1458	0.5926	8.5	3.1	11.9
f-50-100_T2100	100	2100	1827	0.9585	208.7	176.9	28.0
f-50-100_T2500	100	2500	1891	0.9636	2046	1794	29.2
f-50-100_T2700	100	2700	1893	0.9633	4174	3673	26.9
f-50-100_T2900	100	2900	1893	0.9656	8105	7371	25.8
f-50-300_T1500	300	1500	408	1.0000	0.0	0.0	0.0
f-50-300_T1700	300	1700	1215	1.0000	0.0	0.0	0.0
f-50-300_T1900	300	1900	1562	0.0667	0.6	0.1	3.4
f-50-300_T2100	300	2100	1869	0.9635	93.4	80.0	24.3
f-50-300_T2300	300	2300	1889	0.9586	670.4	572.4	28.8
f-50-300_T2500	300	2500	1894	0.9609	2228	1892	31.4
f-50-300_T2700	300	2700	1893	0.9631	5080	4441	28.5
f-50-300_T2900	300	2900	1895	0.9655	8494	7665	26.3
f-50-500_T1500	500	1500	434	1.0000	0.0	0.0	0.0
f-50-500_T2100	500	2100	1886	0.9467	157.5	113.8	27.3
f-50-500_T2300	500	2300	1891	0.9589	808.1	663.3	31.3
f-50-500_T2500	500	2500	1892	0.9581	2085	1751	28.4
f-50-500_T2700	500	2700	1895	0.9622	4897	4216	29.2
f-50-500_T2900	500	2900	1893	0.9655	8037	7244	27.0
f-50-700_T1700	700	1700	1154	1.0000	0.0	0.0	0.0
f-50-700_T1900	700	1900	1812	0.9550	34.0	27.5	21.3
f-50-700_T2100	700	2100	1874	0.9562	143.7	115.1	27.1
f-50-700_T2500	700	2500	1894	0.9619	2213	1916	28.9
f-50-700_T2700	700	2700	1893	0.9621	4303	3713	28.5
f-50-700_T2900	700	2900	1894	0.9644	8040	7131	27.6
f-50-900_T2100	900	2100	1876	0.9632	164.8	133.9	23.0
f-50-900_T2500	900	2500	1884	0.9600	1940	1651	31.2
f-50-900_T2900	900	2900	1893	0.9640	7798	6850	28.8
**Repeat: strat45 Case: A Model: physical_MLR**							
f-50-1_T1700	1	1700	357	0.0000	20,398	16,553	1,655,299

continued on next page

Table S12 (continued)

Simulation	$n(O_2)$	T (K)	n_{rows}	R^2	RMSE (molecule·ps)	MAE (molecule·ps)	MAPE (%)
f-50-1_T1900	1	1900	1318	0.0000	14,958	12,642	1,264,193
f-50-1_T2100	1	2100	1816	-1730.0	13,559	11,585	147,699
f-50-1_T2500	1	2500	1892	-1.9571	15,043	13,462	409.4
f-50-1_T2700	1	2700	1893	0.7393	11,064	10,052	322.7
f-50-1_T2900	1	2900	1892	0.4111	31,043	23,659	162.3
f-50-100_T1500	100	1500	175	0.0000	28,246	24,053	2,405,268
f-50-100_T1700	100	1700	663	0.0000	18,148	14,572	1,457,222
f-50-100_T1900	100	1900	1458	-1.20×10^6	14,545	12,328	962,754
f-50-100_T2100	100	2100	1827	-156.6	12,860	11,070	29,396
f-50-100_T2500	100	2500	1891	0.2824	9083	8318	340.0
f-50-100_T2700	100	2700	1893	0.7750	10,333	9464	246.3
f-50-100_T2900	100	2900	1893	0.3538	35,125	26,178	161.2
f-50-300_T1500	300	1500	408	0.0000	29,489	26,079	2,607,913
f-50-300_T1700	300	1700	1215	0.0000	19,321	15,232	1,523,152
f-50-300_T1900	300	1900	1562	-4.88×10^8	14,784	12,225	1,198,313
f-50-300_T2100	300	2100	1869	-961.7	15,159	12,992	273,892
f-50-300_T2300	300	2300	1889	-20.1593	15,158	12,764	4316
f-50-300_T2500	300	2500	1894	0.0438	11,022	10,036	2052
f-50-300_T2700	300	2700	1893	0.7878	12,181	10,473	293.3
f-50-300_T2900	300	2900	1895	0.4150	34,987	26,472	181.7
f-50-500_T1500	500	1500	434	0.0000	28,218	24,396	2,439,626
f-50-500_T2100	500	2100	1886	-456.4	14,586	12,624	311,160
f-50-500_T2300	500	2300	1891	-11.8902	14,318	12,216	7491
f-50-500_T2500	500	2500	1892	-0.5361	12,627	11,345	306.5
f-50-500_T2700	500	2700	1895	0.7506	12,579	11,032	436.9
f-50-500_T2900	500	2900	1893	0.4792	31,209	24,118	247.7
f-50-700_T1700	700	1700	1154	0.0000	19,887	15,421	1,542,095
f-50-700_T1900	700	1900	1812	-11,862.1	17,485	14,392	675,277
f-50-700_T2100	700	2100	1874	-474.5	14,968	12,868	230,196
f-50-700_T2500	700	2500	1894	0.4638	8298	7512	524.6
f-50-700_T2700	700	2700	1893	0.7682	10,646	9761	346.2
f-50-700_T2900	700	2900	1894	0.4993	30,140	23,103	265.1
f-50-900_T2100	900	2100	1876	-283.9	14,499	12,445	531,010
f-50-900_T2500	900	2500	1884	-0.9793	13,649	12,712	674.5
f-50-900_T2900	900	2900	1893	0.5269	28,276	21,783	381.9
**Repeat: strat45 Case: A Model: physical_RF**							

continued on next page

Table S12 (continued)

Simulation	$n(O_2)$	T (K)	n_{rows}	R^2	RMSE (molecule·ps)	MAE (molecule·ps)	MAPE (%)
f-50-1_T1700	1	1700	357	0.0000	0.0	0.0	0.8
f-50-1_T1900	1	1900	1318	0.0000	14.3	5.0	502.5
f-50-1_T2100	1	2100	1816	0.5039	229.5	167.4	45.9
f-50-1_T2500	1	2500	1892	0.8974	2802	1812	14.1
f-50-1_T2700	1	2700	1893	0.9947	1579	602.6	4.6
f-50-1_T2900	1	2900	1892	0.9712	6868	4557	8.8
f-50-100_T1500	100	1500	175	0.0000	0.0	0.0	0.9
f-50-100_T1700	100	1700	663	0.0000	1.0	0.3	29.3
f-50-100_T1900	100	1900	1458	-0.1131	14.0	4.9	22.2
f-50-100_T2100	100	2100	1827	0.3861	802.5	594.4	58.9
f-50-100_T2500	100	2500	1891	0.9634	2052	1477	10.6
f-50-100_T2700	100	2700	1893	0.9968	1238	676.9	6.4
f-50-100_T2900	100	2900	1893	0.9967	2523	1985	6.6
f-50-300_T1500	300	1500	408	0.0000	0.0	0.0	1.5
f-50-300_T1700	300	1700	1215	0.0000	0.2	0.0	2.7
f-50-300_T1900	300	1900	1562	-414.7	13.7	4.8	417.3
f-50-300_T2100	300	2100	1869	-1.5541	780.8	566.2	569.8
f-50-300_T2300	300	2300	1889	0.9413	798.1	627.4	25.5
f-50-300_T2500	300	2500	1894	0.9943	853.4	528.0	37.2
f-50-300_T2700	300	2700	1893	0.9352	6731	4882	16.2
f-50-300_T2900	300	2900	1895	0.9946	3363	2478	5.5
f-50-500_T1500	500	1500	434	0.0000	0.2	0.0	3.2
f-50-500_T2100	500	2100	1886	0.8718	244.2	173.4	75.5
f-50-500_T2300	500	2300	1891	0.9604	793.8	630.4	49.4
f-50-500_T2500	500	2500	1892	0.9689	1798	1403	13.6
f-50-500_T2700	500	2700	1895	0.9866	2910	2369	13.4
f-50-500_T2900	500	2900	1893	0.9925	3745	3060	11.5
f-50-700_T1700	700	1700	1154	0.0000	0.0	0.0	2.5
f-50-700_T1900	700	1900	1812	-1.1425	235.0	171.8	80.3
f-50-700_T2100	700	2100	1874	0.9676	123.5	95.1	40.4
f-50-700_T2500	700	2500	1894	0.9634	2168	1773	15.4
f-50-700_T2700	700	2700	1893	0.9569	4590	3403	12.6
f-50-700_T2900	700	2900	1894	0.9951	2995	2572	11.2
f-50-900_T2100	900	2100	1876	0.8898	285.1	212.1	427.2
f-50-900_T2500	900	2500	1884	0.9172	2792	2262	46.0
f-50-900_T2900	900	2900	1893	0.9918	3723	3186	18.2

continued on next page

Table S12 (continued)

Simulation	$n(O_2)$	T (K)	n_{rows}	R^2	RMSE (molecule·ps)	MAE (molecule·ps)	MAPE (%)
**Repeat: strat45**	**Case: B**	**Model: MLR**					
f-50-1_T1700	1	1700	357	0.0000	20,017	18,447	1,844,668
f-50-1_T1900	1	1900	1318	0.0000	22,341	20,805	2,080,479
f-50-1_T2100	1	2100	1816	-2617.2	16,675	16,010	69,207
f-50-1_T2500	1	2500	1892	0.7824	4081	3718	711.0
f-50-1_T2700	1	2700	1893	-0.0353	22,046	20,094	453.2
f-50-1_T2900	1	2900	1892	-0.2891	45,929	36,414	110.1
f-50-100_T1500	100	1500	175	0.0000	15,020	13,081	1,308,122
f-50-100_T1700	100	1700	663	0.0000	17,623	15,876	1,587,554
f-50-100_T1900	100	1900	1458	-2.03×10^6	18,924	17,248	1,318,993
f-50-100_T2100	100	2100	1827	-179.8	13,772	12,855	6876
f-50-100_T2500	100	2500	1891	0.9395	2638	1974	266.9
f-50-100_T2700	100	2700	1893	0.8856	7367	5133	101.3
f-50-100_T2900	100	2900	1893	-0.0852	45,521	32,957	68.1
f-50-300_T1500	300	1500	408	0.0000	15,956	14,699	1,469,916
f-50-300_T1700	300	1700	1215	0.0000	11,861	9926	992,598
f-50-300_T1900	300	1900	1562	-3.11×10^8	11,809	9790	949,068
f-50-300_T2100	300	2100	1869	-1850.0	21,019	17,057	49,492
f-50-300_T2300	300	2300	1889	-37.7974	20,526	17,402	2631
f-50-300_T2500	300	2500	1894	-0.4517	13,580	12,530	4477
f-50-300_T2700	300	2700	1893	0.7096	14,252	12,289	256.4
f-50-300_T2900	300	2900	1895	0.3824	35,951	25,908	115.4
f-50-500_T1500	500	1500	434	0.0000	7913	6793	679,322
f-50-500_T2100	500	2100	1886	-1013.7	21,725	18,315	205,366
f-50-500_T2300	500	2300	1891	-30.2352	22,289	19,900	8816
f-50-500_T2500	500	2500	1892	-2.5930	19,311	18,227	1238
f-50-500_T2700	500	2700	1895	0.6166	15,596	13,071	634.3
f-50-500_T2900	500	2900	1893	0.2022	38,628	28,259	228.3
f-50-700_T1700	700	1700	1154	0.0000	21,054	20,151	2,015,135
f-50-700_T1900	700	1900	1812	-9755.4	15,857	14,903	192,414
f-50-700_T2100	700	2100	1874	-983.0	21,533	19,001	286,287
f-50-700_T2500	700	2500	1894	0.6267	6924	6480	390.6
f-50-700_T2700	700	2700	1893	0.5096	15,486	13,588	972.1
f-50-700_T2900	700	2900	1894	-0.1378	45,436	34,049	354.9
f-50-900_T2100	900	2100	1876	-441.2	18,063	15,715	188,587
f-50-900_T2500	900	2500	1884	-3.5670	20,732	19,417	7768

continued on next page

Table S12 (continued)

Simulation	$n(O_2)$	T (K)	n_{rows}	R^2	RMSE (molecule·ps)	MAE (molecule·ps)	MAPE (%)
f-50-900_T2900	900	2900	1893	0.3042	34,294	24,241	223.6
**Repeat: strat45**	**Case: B**	**Model: DT**					
f-50-1_T1700	1	1700	357	1.0000	0.0	0.0	0.0
f-50-1_T1900	1	1900	1318	0.0000	17.5	7.7	769.3
f-50-1_T2100	1	2100	1816	-9226.0	31,305	6647	708.0
f-50-1_T2500	1	2500	1892	-0.5044	10,730	8689	101.1
f-50-1_T2700	1	2700	1893	-0.8591	29,543	19,980	52.9
f-50-1_T2900	1	2900	1892	0.3666	32,194	25,431	46.4
f-50-100_T1500	100	1500	175	0.0000	0.1	0.0	1.4
f-50-100_T1700	100	1700	663	1.0000	0.0	0.0	0.0
f-50-100_T1900	100	1900	1458	-76.1555	116.7	31.3	88.8
f-50-100_T2100	100	2100	1827	-901.1	30,766	7249	290.7
f-50-100_T2500	100	2500	1891	-2.1122	18,916	7352	71.8
f-50-100_T2700	100	2700	1893	0.4060	16,790	12,391	45.6
f-50-100_T2900	100	2900	1893	0.3139	36,194	27,618	48.6
f-50-300_T1500	300	1500	408	1.0000	0.0	0.0	0.0
f-50-300_T1700	300	1700	1215	0.0000	0.8	0.3	25.8
f-50-300_T1900	300	1900	1562	-101,540.9	213.3	136.7	13,178
f-50-300_T2100	300	2100	1869	-1509.6	18,989	6724	1065
f-50-300_T2300	300	2300	1889	-2.8548	6470	4220	84.9
f-50-300_T2500	300	2500	1894	-0.5037	13,821	8215	100.4
f-50-300_T2700	300	2700	1893	0.7378	13,543	9479	35.9
f-50-300_T2900	300	2900	1895	0.4233	34,739	25,424	42.6
f-50-500_T1500	500	1500	434	1.0000	0.0	0.0	0.0
f-50-500_T2100	500	2100	1886	-28.4762	3703	2163	253.3
f-50-500_T2300	500	2300	1891	-0.0822	4149	2704	93.0
f-50-500_T2500	500	2500	1892	0.5993	6449	4342	36.7
f-50-500_T2700	500	2700	1895	0.8440	9949	7388	38.4
f-50-500_T2900	500	2900	1893	0.5936	27,570	19,492	36.4
f-50-700_T1700	700	1700	1154	1.0000	0.0	0.0	0.0
f-50-700_T1900	700	1900	1812	-1.2888	242.9	184.4	907.9
f-50-700_T2100	700	2100	1874	-5.7590	1785	608.3	64.3
f-50-700_T2500	700	2500	1894	-0.0603	11,669	6387	73.2
f-50-700_T2700	700	2700	1893	-0.5389	27,433	15,146	37.6
f-50-700_T2900	700	2900	1894	0.4679	31,070	21,934	36.4
f-50-900_T2100	900	2100	1876	-18.3170	3775	3119	85,375

continued on next page

Table S12 (continued)

Simulation	$n(O_2)$	T (K)	n_{rows}	R^2	RMSE (molecule·ps)	MAE (molecule·ps)	MAPE (%)
f-50-900_T2500	900	2500	1884	-3.6912	21,012	11,091	78.8
f-50-900_T2900	900	2900	1893	-0.0293	41,710	28,341	64.8
**Repeat: strat45**	**Case: B**	**Model: RF**					
f-50-1_T1700	1	1700	357	0.0000	2374	1586	158,614
f-50-1_T1900	1	1900	1318	0.0000	1241	783.1	78,310
f-50-1_T2100	1	2100	1816	-549.1	7643	3800	588.1
f-50-1_T2500	1	2500	1892	0.4672	6385	5098	59.4
f-50-1_T2700	1	2700	1893	0.9425	5194	3272	14.9
f-50-1_T2900	1	2900	1892	0.4550	29,865	21,477	33.0
f-50-100_T1500	100	1500	175	0.0000	4.2	2.0	200.3
f-50-100_T1700	100	1700	663	0.0000	464.1	296.8	29,682
f-50-100_T1900	100	1900	1458	-206,313.1	6032	4115	264,595
f-50-100_T2100	100	2100	1827	-21.2047	4827	2697	176.3
f-50-100_T2500	100	2500	1891	0.7289	5583	4562	55.2
f-50-100_T2700	100	2700	1893	0.9028	6791	4369	14.5
f-50-100_T2900	100	2900	1893	0.2010	39,059	28,611	43.1
f-50-300_T1500	300	1500	408	0.0000	0.1	0.0	1.1
f-50-300_T1700	300	1700	1215	0.0000	450.6	188.8	18,879
f-50-300_T1900	300	1900	1562	-5.10×10^7	4781	3129	295,298
f-50-300_T2100	300	2100	1869	-1517.7	19,040	13,007	1761
f-50-300_T2300	300	2300	1889	-5.9914	8713	6142	122.7
f-50-300_T2500	300	2500	1894	0.3768	8898	5658	105.1
f-50-300_T2700	300	2700	1893	0.6978	14,539	9291	25.9
f-50-300_T2900	300	2900	1895	0.1499	42,179	29,209	39.2
f-50-500_T1500	500	1500	434	0.0000	7521	5353	535,286
f-50-500_T2100	500	2100	1886	-36.7973	4193	2935	1060
f-50-500_T2300	500	2300	1891	-0.6179	5073	3185	102.6
f-50-500_T2500	500	2500	1892	0.7375	5220	3796	34.5
f-50-500_T2700	500	2700	1895	0.9642	4767	3020	19.2
f-50-500_T2900	500	2900	1893	0.5545	28,864	20,383	33.5
f-50-700_T1700	700	1700	1154	0.0000	4288	2992	299,175
f-50-700_T1900	700	1900	1812	-125.8	1808	1420	1905
f-50-700_T2100	700	2100	1874	-2.7837	1335	759.7	255.4
f-50-700_T2500	700	2500	1894	0.8691	4100	2623	52.8
f-50-700_T2700	700	2700	1893	0.7474	11,115	6843	21.3
f-50-700_T2900	700	2900	1894	0.7562	21,030	13,902	24.7

continued on next page

Table S12 (continued)

Simulation	$n(O_2)$	T (K)	n_{rows}	R^2	RMSE (molecule·ps)	MAE (molecule·ps)	MAPE (%)
f-50-900_T2100	900	2100	1876	-21.3655	4062	3252	28,888
f-50-900_T2500	900	2500	1884	-4.3651	22,471	14,938	96.4
f-50-900_T2900	900	2900	1893	0.4535	30,391	17,720	37.8
**Repeat: strat45**	**Case: B**	**Model: XGBoost**					
f-50-1_T1700	1	1700	357	0.0000	971.6	582.4	58,236
f-50-1_T1900	1	1900	1318	0.0000	3601	2956	295,604
f-50-1_T2100	1	2100	1816	-645.2	8284	3670	1722
f-50-1_T2500	1	2500	1892	0.3665	6963	6121	154.3
f-50-1_T2700	1	2700	1893	0.9332	5600	3941	35.5
f-50-1_T2900	1	2900	1892	0.5670	26,618	19,662	45.3
f-50-100_T1500	100	1500	175	0.0000	1000	609.2	60,916
f-50-100_T1700	100	1700	663	0.0000	4959	2977	297,682
f-50-100_T1900	100	1900	1458	-368,753.9	8065	4430	184,596
f-50-100_T2100	100	2100	1827	-14.7637	4067	2140	356.4
f-50-100_T2500	100	2500	1891	0.8204	4544	2873	89.0
f-50-100_T2700	100	2700	1893	0.7600	10,671	7992	39.0
f-50-100_T2900	100	2900	1893	0.6578	25,562	20,933	56.7
f-50-300_T1500	300	1500	408	0.0000	618.6	310.4	31,038
f-50-300_T1700	300	1700	1215	0.0000	3121	1810	181,048
f-50-300_T1900	300	1900	1562	-2.25×10^6	1004	703.2	65,940
f-50-300_T2100	300	2100	1869	-574.0	11,715	5770	2608
f-50-300_T2300	300	2300	1889	-6.2950	8900	6522	306.8
f-50-300_T2500	300	2500	1894	0.6087	7051	4711	137.2
f-50-300_T2700	300	2700	1893	0.5558	17,626	12,544	51.4
f-50-300_T2900	300	2900	1895	0.1945	41,057	27,327	38.5
f-50-500_T1500	500	1500	434	0.0000	3148	2446	244,590
f-50-500_T2100	500	2100	1886	-10.4186	2305	1438	18,779
f-50-500_T2300	500	2300	1891	-0.1821	4336	2448	277.3
f-50-500_T2500	500	2500	1892	0.7331	5263	3791	46.2
f-50-500_T2700	500	2700	1895	0.7895	11,556	6988	44.0
f-50-500_T2900	500	2900	1893	0.1799	39,164	25,335	37.8
f-50-700_T1700	700	1700	1154	0.0000	1952	1422	142,203
f-50-700_T1900	700	1900	1812	-79.0128	1436	1139	14,901
f-50-700_T2100	700	2100	1874	-15.2618	2768	1935	4051
f-50-700_T2500	700	2500	1894	0.8251	4740	3312	109.4
f-50-700_T2700	700	2700	1893	0.7842	10,273	6319	33.2

continued on next page

Table S12 (continued)

Simulation	$n(O_2)$	T (K)	n_{rows}	R^2	RMSE (molecule·ps)	MAE (molecule·ps)	MAPE (%)
f-50-700_T2900	700	2900	1894	0.6449	25,384	16,122	28.8
f-50-900_T2100	900	2100	1876	-19.2857	3869	3235	21,438
f-50-900_T2500	900	2500	1884	-3.3482	20,230	11,236	108.0
f-50-900_T2900	900	2900	1893	0.3163	33,993	21,178	51.9
**Repeat: strat45**	**Case: B**		**Model: CatBoost**				
f-50-1_T1700	1	1700	357	0.0000	1388	888.0	88,796
f-50-1_T1900	1	1900	1318	0.0000	760.6	577.0	57,698
f-50-1_T2100	1	2100	1816	-541.8	7593	5027	1311
f-50-1_T2500	1	2500	1892	0.9639	1662	1246	60.1
f-50-1_T2700	1	2700	1893	0.9478	4949	3833	26.1
f-50-1_T2900	1	2900	1892	0.4435	30,176	23,327	44.9
f-50-100_T1500	100	1500	175	0.0000	813.3	620.1	62,011
f-50-100_T1700	100	1700	663	0.0000	1587	1047	104,671
f-50-100_T1900	100	1900	1458	-123,051.0	4659	2953	158,767
f-50-100_T2100	100	2100	1827	-13.3300	3878	2430	557.5
f-50-100_T2500	100	2500	1891	-0.6002	13,563	8350	121.5
f-50-100_T2700	100	2700	1893	0.8090	9520	7301	34.1
f-50-100_T2900	100	2900	1893	0.5458	29,449	23,226	41.7
f-50-300_T1500	300	1500	408	0.0000	1904	1397	139,686
f-50-300_T1700	300	1700	1215	0.0000	1282	826.4	82,638
f-50-300_T1900	300	1900	1562	-6.70×10^6	1733	1130	108,454
f-50-300_T2100	300	2100	1869	-1403.8	18,311	12,218	3453
f-50-300_T2300	300	2300	1889	-1.3694	5073	3288	81.5
f-50-300_T2500	300	2500	1894	0.8061	4963	3005	180.5
f-50-300_T2700	300	2700	1893	0.8340	10,774	7771	38.6
f-50-300_T2900	300	2900	1895	0.3245	37,598	25,340	38.1
f-50-500_T1500	500	1500	434	0.0000	2520	1456	145,607
f-50-500_T2100	500	2100	1886	-34.7927	4080	2813	7537
f-50-500_T2300	500	2300	1891	0.5547	2661	1509	193.4
f-50-500_T2500	500	2500	1892	0.8690	3688	2538	46.7
f-50-500_T2700	500	2700	1895	0.9612	4961	3618	28.0
f-50-500_T2900	500	2900	1893	0.6819	24,391	16,142	30.6
f-50-700_T1700	700	1700	1154	0.0000	1730	1467	146,678
f-50-700_T1900	700	1900	1812	-110.0	1692	1439	19,059
f-50-700_T2100	700	2100	1874	-4.6922	1638	1081	1088
f-50-700_T2500	700	2500	1894	0.7918	5171	3535	71.6

continued on next page

Table S12 (continued)

Simulation	$n(O_2)$	T (K)	n_{rows}	R^2	RMSE (molecule·ps)	MAE (molecule·ps)	MAPE (%)
f-50-700_T2700	700	2700	1893	0.5890	14,177	8370	28.3
f-50-700_T2900	700	2900	1894	0.6270	26,014	16,520	23.8
f-50-900_T2100	900	2100	1876	-6.0451	2280	1954	46,435
f-50-900_T2500	900	2500	1884	-5.0529	23,868	14,882	98.1
f-50-900_T2900	900	2900	1893	0.5287	28,225	17,292	35.8
**Repeat: strat45**		**Case: B**		**Model: ANN**			
f-50-1_T1700	1	1700	357	0.0000	596.0	392.1	39,206
f-50-1_T1900	1	1900	1318	0.0000	2505	1338	133,800
f-50-1_T2100	1	2100	1816	-8.7268	1016	734.3	1644
f-50-1_T2500	1	2500	1892	0.9334	2257	1661	25.6
f-50-1_T2700	1	2700	1893	0.9753	3407	2494	29.9
f-50-1_T2900	1	2900	1892	0.9150	11,791	7492	17.1
f-50-100_T1500	100	1500	175	0.0000	238.2	231.4	23,140
f-50-100_T1700	100	1700	663	0.0000	235.7	155.2	15,516
f-50-100_T1900	100	1900	1458	-221.3	198.0	163.7	10,963
f-50-100_T2100	100	2100	1827	-1.2112	1523	1212	654.7
f-50-100_T2500	100	2500	1891	0.9606	2128	1589	64.2
f-50-100_T2700	100	2700	1893	0.9338	5605	3413	19.8
f-50-100_T2900	100	2900	1893	0.9606	8678	6527	24.1
f-50-300_T1500	300	1500	408	0.0000	834.3	683.3	68,327
f-50-300_T1700	300	1700	1215	0.0000	422.0	404.9	40,492
f-50-300_T1900	300	1900	1562	-272,204.2	349.3	326.8	32,246
f-50-300_T2100	300	2100	1869	-474.1	10,649	5149	1176
f-50-300_T2300	300	2300	1889	-1.5533	5266	2930	173.3
f-50-300_T2500	300	2500	1894	0.8856	3813	2600	147.6
f-50-300_T2700	300	2700	1893	0.9753	4153	2904	13.5
f-50-300_T2900	300	2900	1895	0.9610	9031	6310	14.9
f-50-500_T1500	500	1500	434	0.0000	118.6	99.4	9937
f-50-500_T2100	500	2100	1886	0.6652	394.6	323.0	2514
f-50-500_T2300	500	2300	1891	0.2527	3448	1719	157.0
f-50-500_T2500	500	2500	1892	0.8356	4130	1965	30.5
f-50-500_T2700	500	2700	1895	0.9843	3161	2421	25.3
f-50-500_T2900	500	2900	1893	0.9880	4746	3276	10.2
f-50-700_T1700	700	1700	1154	0.0000	2611	1942	194,186
f-50-700_T1900	700	1900	1812	-71.9755	1371	1025	5330
f-50-700_T2100	700	2100	1874	0.7325	355.0	257.8	515.2

continued on next page

Table S12 (continued)

Simulation	$n(O_2)$	T (K)	n_{rows}	R^2	RMSE (molecule·ps)	MAE (molecule·ps)	MAPE (%)
f-50-700_T2500	700	2500	1894	0.9491	2558	1846	62.1
f-50-700_T2700	700	2700	1893	0.9686	3920	2199	14.4
f-50-700_T2900	700	2900	1894	0.9817	5766	4096	14.1
f-50-900_T2100	900	2100	1876	-0.0728	889.7	736.2	25,004
f-50-900_T2500	900	2500	1884	0.9201	2742	1709	58.0
f-50-900_T2900	900	2900	1893	0.9718	6902	5412	35.4
**Repeat: strat45 Case: B Model: SVR**							
f-50-1_T1700	1	1700	357	0.0000	439.5	338.8	33,883
f-50-1_T1900	1	1900	1318	0.0000	612.9	594.0	59,403
f-50-1_T2100	1	2100	1816	-119.9	3583	2395	4041
f-50-1_T2500	1	2500	1892	0.8641	3224	2809	140.6
f-50-1_T2700	1	2700	1893	0.4266	16,408	9700	42.6
f-50-1_T2900	1	2900	1892	-0.4951	49,464	36,601	57.5
f-50-100_T1500	100	1500	175	0.0000	264.7	227.2	22,721
f-50-100_T1700	100	1700	663	0.0000	181.2	120.1	12,012
f-50-100_T1900	100	1900	1458	-1640.6	538.1	317.4	13,696
f-50-100_T2100	100	2100	1827	-20.3746	4736	3311	520.6
f-50-100_T2500	100	2500	1891	0.8455	4215	2685	42.0
f-50-100_T2700	100	2700	1893	0.3304	17,827	11,231	33.4
f-50-100_T2900	100	2900	1893	-0.5333	54,109	39,033	55.1
f-50-300_T1500	300	1500	408	0.0000	2989	2582	258,189
f-50-300_T1700	300	1700	1215	0.0000	1061	790.4	79,037
f-50-300_T1900	300	1900	1562	-3.80×10^6	1304	770.8	72,126
f-50-300_T2100	300	2100	1869	-322.4	8786	6596	3451
f-50-300_T2300	300	2300	1889	-4.5520	7765	6375	220.5
f-50-300_T2500	300	2500	1894	0.6632	6541	4091	187.6
f-50-300_T2700	300	2700	1893	0.1328	24,628	15,877	40.6
f-50-300_T2900	300	2900	1895	-0.5254	56,499	40,296	54.0
f-50-500_T1500	500	1500	434	0.0000	666.1	492.5	49,248
f-50-500_T2100	500	2100	1886	-66.0491	5585	4001	5041
f-50-500_T2300	500	2300	1891	-0.8536	5430	4247	323.4
f-50-500_T2500	500	2500	1892	0.7777	4803	3263	55.4
f-50-500_T2700	500	2700	1895	0.0693	24,298	15,713	45.8
f-50-500_T2900	500	2900	1893	-0.5542	53,914	38,775	55.8
f-50-700_T1700	700	1700	1154	0.0000	6342	5997	599,700
f-50-700_T1900	700	1900	1812	-306.8	2817	2721	46,144

continued on next page

Table S12 (continued)

Simulation	$n(O_2)$	T (K)	n_{rows}	R^2	RMSE (molecule·ps)	MAE (molecule·ps)	MAPE (%)
f-50-700_T2100	700	2100	1874	-12.8978	2559	1763	4406
f-50-700_T2500	700	2500	1894	0.6979	6229	4369	118.8
f-50-700_T2700	700	2700	1893	0.3537	17,779	10,764	38.8
f-50-700_T2900	700	2900	1894	-0.3052	48,663	33,562	46.9
f-50-900_T2100	900	2100	1876	-8.2069	2606	2302	55,439
f-50-900_T2500	900	2500	1884	0.8489	3771	2842	158.6
f-50-900_T2900	900	2900	1893	-0.3781	48,262	33,105	106.3
**Repeat: strat45**	**Case: B**	**Model: GPR**					
f-50-1_T1700	1	1700	357	0.0000	309.4	204.1	20,406
f-50-1_T1900	1	1900	1318	0.0000	436.4	292.6	29,259
f-50-1_T2100	1	2100	1816	-91.3016	3131	1876	722.5
f-50-1_T2500	1	2500	1892	0.3108	7262	5288	54.9
f-50-1_T2700	1	2700	1893	0.9155	6300	3977	14.6
f-50-1_T2900	1	2900	1892	0.6809	22,850	17,914	34.9
f-50-100_T1500	100	1500	175	0.0000	178.0	122.2	12,215
f-50-100_T1700	100	1700	663	0.0000	611.5	421.9	42,192
f-50-100_T1900	100	1900	1458	-23,756.0	2047	1201	54,915
f-50-100_T2100	100	2100	1827	-10.7553	3512	2504	234.6
f-50-100_T2500	100	2500	1891	0.7582	5272	3529	27.7
f-50-100_T2700	100	2700	1893	0.7841	10,123	6740	18.7
f-50-100_T2900	100	2900	1893	0.9392	10,772	8960	19.5
f-50-300_T1500	300	1500	408	0.0000	2152	1494	149,437
f-50-300_T1700	300	1700	1215	0.0000	3265	2250	225,002
f-50-300_T1900	300	1900	1562	-7.65×10^7	5855	4423	430,562
f-50-300_T2100	300	2100	1869	-630.7	12,280	8037	2981
f-50-300_T2300	300	2300	1889	0.6522	1943	1465	59.7
f-50-300_T2500	300	2500	1894	0.9256	3075	2076	85.3
f-50-300_T2700	300	2700	1893	0.9233	7325	4961	16.1
f-50-300_T2900	300	2900	1895	0.9285	12,230	9058	16.7
f-50-500_T1500	500	1500	434	0.0000	399.4	288.4	28,840
f-50-500_T2100	500	2100	1886	-9.2734	2186	1603	1744
f-50-500_T2300	500	2300	1891	0.9486	903.8	629.1	64.7
f-50-500_T2500	500	2500	1892	0.9401	2493	1620	21.4
f-50-500_T2700	500	2700	1895	0.9689	4440	3324	22.4
f-50-500_T2900	500	2900	1893	0.9374	10,824	7985	18.1
f-50-700_T1700	700	1700	1154	0.0000	6438	5684	568,371

continued on next page

Table S12 (continued)

Simulation	$n(O_2)$	T (K)	n_{rows}	R^2	RMSE (molecule·ps)	MAE (molecule·ps)	MAPE (%)
f-50-700_T1900	700	1900	1812	-177.6	2145	1794	27,143
f-50-700_T2100	700	2100	1874	-7.6030	2013	1341	674.8
f-50-700_T2500	700	2500	1894	0.9205	3195	2241	56.4
f-50-700_T2700	700	2700	1893	0.9239	6099	3481	11.3
f-50-700_T2900	700	2900	1894	0.9264	11,558	8447	15.0
f-50-900_T2100	900	2100	1876	-12.7619	3187	2920	39,848
f-50-900_T2500	900	2500	1884	-0.7348	12,778	7544	60.8
f-50-900_T2900	900	2900	1893	0.7105	22,120	15,501	42.6
**Repeat: strat45 Case: B Model: persistence**							
f-50-1_T1700	1	1700	357	1.0000	0.0	0.0	0.0
f-50-1_T1900	1	1900	1318	1.0000	0.0	0.0	0.0
f-50-1_T2100	1	2100	1816	0.9631	62.6	54.9	23.7
f-50-1_T2500	1	2500	1892	0.9646	1647	1468	28.5
f-50-1_T2700	1	2700	1893	0.9644	4090	3642	27.3
f-50-1_T2900	1	2900	1892	0.9666	7390	6812	25.5
f-50-100_T1500	100	1500	175	1.0000	0.0	0.0	0.0
f-50-100_T1700	100	1700	663	1.0000	0.0	0.0	0.0
f-50-100_T1900	100	1900	1458	0.5926	8.5	3.1	11.9
f-50-100_T2100	100	2100	1827	0.9585	208.7	176.9	28.0
f-50-100_T2500	100	2500	1891	0.9636	2046	1794	29.2
f-50-100_T2700	100	2700	1893	0.9633	4174	3673	26.9
f-50-100_T2900	100	2900	1893	0.9656	8105	7371	25.8
f-50-300_T1500	300	1500	408	1.0000	0.0	0.0	0.0
f-50-300_T1700	300	1700	1215	1.0000	0.0	0.0	0.0
f-50-300_T1900	300	1900	1562	0.0667	0.6	0.1	3.4
f-50-300_T2100	300	2100	1869	0.9635	93.4	80.0	24.3
f-50-300_T2300	300	2300	1889	0.9586	670.4	572.4	28.8
f-50-300_T2500	300	2500	1894	0.9609	2228	1892	31.4
f-50-300_T2700	300	2700	1893	0.9631	5080	4441	28.5
f-50-300_T2900	300	2900	1895	0.9655	8494	7665	26.3
f-50-500_T1500	500	1500	434	1.0000	0.0	0.0	0.0
f-50-500_T2100	500	2100	1886	0.9467	157.5	113.8	27.3
f-50-500_T2300	500	2300	1891	0.9589	808.1	663.3	31.3
f-50-500_T2500	500	2500	1892	0.9581	2085	1751	28.4
f-50-500_T2700	500	2700	1895	0.9622	4897	4216	29.2
f-50-500_T2900	500	2900	1893	0.9655	8037	7244	27.0

continued on next page

Table S12 (continued)

Simulation	$n(O_2)$	T (K)	n_{rows}	R^2	RMSE (molecule·ps)	MAE (molecule·ps)	MAPE (%)
f-50-700_T1700	700	1700	1154	1.0000	0.0	0.0	0.0
f-50-700_T1900	700	1900	1812	0.9550	34.0	27.5	21.3
f-50-700_T2100	700	2100	1874	0.9562	143.7	115.1	27.1
f-50-700_T2500	700	2500	1894	0.9619	2213	1916	28.9
f-50-700_T2700	700	2700	1893	0.9621	4303	3713	28.5
f-50-700_T2900	700	2900	1894	0.9644	8040	7131	27.6
f-50-900_T2100	900	2100	1876	0.9632	164.8	133.9	23.0
f-50-900_T2500	900	2500	1884	0.9600	1940	1651	31.2
f-50-900_T2900	900	2900	1893	0.9640	7798	6850	28.8
**Repeat: strat45**	**Case: B**	**Model: physical_MLR**					
f-50-1_T1700	1	1700	357	0.0000	20,398	16,553	1,655,299
f-50-1_T1900	1	1900	1318	0.0000	14,958	12,642	1,264,193
f-50-1_T2100	1	2100	1816	-1730.0	13,559	11,585	147,699
f-50-1_T2500	1	2500	1892	-1.9571	15,043	13,462	409.4
f-50-1_T2700	1	2700	1893	0.7393	11,064	10,052	322.7
f-50-1_T2900	1	2900	1892	0.4111	31,043	23,659	162.3
f-50-100_T1500	100	1500	175	0.0000	28,246	24,053	2,405,268
f-50-100_T1700	100	1700	663	0.0000	18,148	14,572	1,457,222
f-50-100_T1900	100	1900	1458	-1.20×10^6	14,545	12,328	962,754
f-50-100_T2100	100	2100	1827	-156.6	12,860	11,070	29,396
f-50-100_T2500	100	2500	1891	0.2824	9083	8318	340.0
f-50-100_T2700	100	2700	1893	0.7750	10,333	9464	246.3
f-50-100_T2900	100	2900	1893	0.3538	35,125	26,178	161.2
f-50-300_T1500	300	1500	408	0.0000	29,489	26,079	2,607,913
f-50-300_T1700	300	1700	1215	0.0000	19,321	15,232	1,523,152
f-50-300_T1900	300	1900	1562	-4.88×10^8	14,784	12,225	1,198,313
f-50-300_T2100	300	2100	1869	-961.7	15,159	12,992	273,892
f-50-300_T2300	300	2300	1889	-20.1593	15,158	12,764	4316
f-50-300_T2500	300	2500	1894	0.0438	11,022	10,036	2052
f-50-300_T2700	300	2700	1893	0.7878	12,181	10,473	293.3
f-50-300_T2900	300	2900	1895	0.4150	34,987	26,472	181.7
f-50-500_T1500	500	1500	434	0.0000	28,218	24,396	2,439,626
f-50-500_T2100	500	2100	1886	-456.4	14,586	12,624	311,160
f-50-500_T2300	500	2300	1891	-11.8902	14,318	12,216	7491
f-50-500_T2500	500	2500	1892	-0.5361	12,627	11,345	306.5
f-50-500_T2700	500	2700	1895	0.7506	12,579	11,032	436.9

continued on next page

Table S12 (continued)

Simulation	$n(O_2)$	T (K)	n_{rows}	R^2	RMSE (molecule·ps)	MAE (molecule·ps)	MAPE (%)
f-50-500_T2900	500	2900	1893	0.4792	31,209	24,118	247.7
f-50-700_T1700	700	1700	1154	0.0000	19,887	15,421	1,542,095
f-50-700_T1900	700	1900	1812	-11,862.1	17,485	14,392	675,277
f-50-700_T2100	700	2100	1874	-474.5	14,968	12,868	230,196
f-50-700_T2500	700	2500	1894	0.4638	8298	7512	524.6
f-50-700_T2700	700	2700	1893	0.7682	10,646	9761	346.2
f-50-700_T2900	700	2900	1894	0.4993	30,140	23,103	265.1
f-50-900_T2100	900	2100	1876	-283.9	14,499	12,445	531,010
f-50-900_T2500	900	2500	1884	-0.9793	13,649	12,712	674.5
f-50-900_T2900	900	2900	1893	0.5269	28,276	21,783	381.9
**Repeat: strat45 Case: B Model: physical_RF**							
f-50-1_T1700	1	1700	357	0.0000	47.0	13.0	1304
f-50-1_T1900	1	1900	1318	0.0000	342.5	243.1	24,315
f-50-1_T2100	1	2100	1816	-0.0541	334.6	254.9	79.8
f-50-1_T2500	1	2500	1892	0.7844	4061	3195	155.4
f-50-1_T2700	1	2700	1893	0.6357	13,077	9535	89.5
f-50-1_T2900	1	2900	1892	0.7757	19,158	15,709	46.8
f-50-100_T1500	100	1500	175	0.0000	156.8	84.5	8451
f-50-100_T1700	100	1700	663	0.0000	83.3	37.7	3771
f-50-100_T1900	100	1900	1458	-4103.4	850.8	572.3	40,145
f-50-100_T2100	100	2100	1827	-0.6605	1320	990.5	90.1
f-50-100_T2500	100	2500	1891	0.9636	2046	1726	340.9
f-50-100_T2700	100	2700	1893	0.9746	3469	2118	14.2
f-50-100_T2900	100	2900	1893	0.7085	23,591	18,962	38.5
f-50-300_T1500	300	1500	408	0.0000	28.9	11.7	1175
f-50-300_T1700	300	1700	1215	0.0000	85.0	42.9	4290
f-50-300_T1900	300	1900	1562	-734,544.4	573.8	455.3	44,096
f-50-300_T2100	300	2100	1869	-9.6506	1594	1085	3108
f-50-300_T2300	300	2300	1889	0.3230	2711	1980	217.5
f-50-300_T2500	300	2500	1894	0.8793	3916	2557	597.5
f-50-300_T2700	300	2700	1893	0.9342	6785	4836	33.1
f-50-300_T2900	300	2900	1895	0.8817	15,737	12,085	27.5
f-50-500_T1500	500	1500	434	0.0000	3880	2952	295,250
f-50-500_T2100	500	2100	1886	-18.6466	3023	2272	2160
f-50-500_T2300	500	2300	1891	0.6814	2251	1645	188.3
f-50-500_T2500	500	2500	1892	0.9365	2567	1842	22.7

continued on next page

Table S12 (continued)

Simulation	$n(O_2)$	T (K)	n_{rows}	R^2	RMSE (molecule·ps)	MAE (molecule·ps)	MAPE (%)
f-50-500_T2700	500	2700	1895	0.9303	6648	5344	106.1
f-50-500_T2900	500	2900	1893	0.9327	11,221	9133	29.6
f-50-700_T1700	700	1700	1154	0.0000	944.9	767.9	76,794
f-50-700_T1900	700	1900	1812	-14.4803	631.6	459.8	1205
f-50-700_T2100	700	2100	1874	0.6954	378.8	269.8	167.3
f-50-700_T2500	700	2500	1894	0.9595	2282	1979	163.9
f-50-700_T2700	700	2700	1893	0.2753	18,826	15,445	222.8
f-50-700_T2900	700	2900	1894	0.9082	12,907	9857	30.8
f-50-900_T2100	900	2100	1876	-107.9	8965	6764	12,787
f-50-900_T2500	900	2500	1884	-0.5641	12,133	8483	244.3
f-50-900_T2900	900	2900	1893	0.8956	13,283	9874	31.8
**Repeat: strat46**	**Case: A**	**Model: MLR**					
f-50-1_T1700	1	1700	357	0.0000	607.7	505.4	50,543
f-50-1_T1900	1	1900	1318	0.0000	495.0	414.2	41,420
f-50-1_T2100	1	2100	1816	-0.6972	424.6	408.3	4540
f-50-1_T2500	1	2500	1892	0.9961	546.2	490.3	130.7
f-50-1_T2700	1	2700	1893	0.9987	782.0	560.0	18.4
f-50-1_T2900	1	2900	1892	0.9984	1626	1467	13.6
f-50-100_T1500	100	1500	175	0.0000	550.6	466.8	46,681
f-50-100_T1700	100	1700	663	0.0000	461.3	395.2	39,524
f-50-100_T1900	100	1900	1458	-1331.2	484.7	407.2	32,290
f-50-100_T2100	100	2100	1827	0.9073	311.9	258.0	690.6
f-50-100_T2500	100	2500	1891	0.9982	448.7	346.1	115.4
f-50-100_T2700	100	2700	1893	0.9992	628.5	557.6	16.2
f-50-100_T2900	100	2900	1893	0.9989	1417	1291	11.8
f-50-300_T1500	300	1500	408	0.0000	138.2	114.6	11,464
f-50-300_T1700	300	1700	1215	0.0000	542.7	535.9	53,590
f-50-300_T1900	300	1900	1562	-19,133.3	92.6	75.1	7462
f-50-300_T2100	300	2100	1869	0.3536	392.8	359.6	10,201
f-50-300_T2300	300	2300	1889	0.9853	399.6	384.9	239.5
f-50-300_T2500	300	2500	1894	0.9980	507.1	386.1	491.8
f-50-300_T2700	300	2700	1893	0.9995	581.8	454.8	48.8
f-50-300_T2900	300	2900	1895	0.9986	1682	1432	6.7
f-50-500_T1500	500	1500	434	0.0000	1298	1296	129,629
f-50-500_T2100	500	2100	1886	0.7983	306.3	286.5	4116
f-50-500_T2300	500	2300	1891	0.9714	674.8	660.5	305.0

continued on next page

Table S12 (continued)

Simulation	$n(O_2)$	T (K)	n_{rows}	R^2	RMSE (molecule·ps)	MAE (molecule·ps)	MAPE (%)
f-50-500_T2500	500	2500	1892	0.9962	631.4	576.5	32.3
f-50-500_T2700	500	2700	1895	0.9994	599.9	475.9	64.6
f-50-500_T2900	500	2900	1893	0.9993	1163	1001	18.4
f-50-700_T1700	700	1700	1154	0.0000	681.2	668.7	66,867
f-50-700_T1900	700	1900	1812	-23.5354	795.2	763.4	24,013
f-50-700_T2100	700	2100	1874	0.7631	334.1	247.0	1396
f-50-700_T2500	700	2500	1894	0.9965	665.8	636.5	80.7
f-50-700_T2700	700	2700	1893	0.9996	468.4	398.8	8.8
f-50-700_T2900	700	2900	1894	0.9989	1417	1169	5.8
f-50-900_T2100	900	2100	1876	-0.2472	959.3	872.0	35,350
f-50-900_T2500	900	2500	1884	0.9983	401.1	359.0	170.9
f-50-900_T2900	900	2900	1893	0.9981	1776	1595	50.0
**Repeat: strat46 Case: A Model: DT**							
f-50-1_T1700	1	1700	357	0.0000	6.2	1.6	157.6
f-50-1_T1900	1	1900	1318	0.0000	3.1	1.8	175.6
f-50-1_T2100	1	2100	1816	0.9432	77.6	56.5	86.8
f-50-1_T2500	1	2500	1892	0.9982	371.3	283.3	7.3
f-50-1_T2700	1	2700	1893	0.9997	360.3	282.8	6.9
f-50-1_T2900	1	2900	1892	0.9995	859.8	678.8	3.3
f-50-100_T1500	100	1500	175	0.0000	1.1	0.9	91.4
f-50-100_T1700	100	1700	663	0.0000	5.0	2.0	203.0
f-50-100_T1900	100	1900	1458	0.1346	12.4	5.0	100.8
f-50-100_T2100	100	2100	1827	0.9582	209.5	134.6	70.9
f-50-100_T2500	100	2500	1891	0.9993	292.8	216.1	10.5
f-50-100_T2700	100	2700	1893	0.9997	377.6	289.4	5.7
f-50-100_T2900	100	2900	1893	0.9998	609.2	447.0	2.8
f-50-300_T1500	300	1500	408	0.0000	1.4	1.4	141.8
f-50-300_T1700	300	1700	1215	0.0000	5.6	3.5	347.4
f-50-300_T1900	300	1900	1562	-101.2	6.8	3.6	353.0
f-50-300_T2100	300	2100	1869	0.9719	81.9	56.8	59.3
f-50-300_T2300	300	2300	1889	0.9946	241.8	200.8	20.2
f-50-300_T2500	300	2500	1894	0.9996	214.9	165.0	25.2
f-50-300_T2700	300	2700	1893	0.9996	502.3	346.8	6.4
f-50-300_T2900	300	2900	1895	0.9997	805.6	454.1	4.2
f-50-500_T1500	500	1500	434	0.0000	3.2	3.2	322.4
f-50-500_T2100	500	2100	1886	0.9930	57.3	43.4	131.9

continued on next page

Table S12 (continued)

Simulation	$n(O_2)$	T (K)	n_{rows}	R^2	RMSE (molecule·ps)	MAE (molecule·ps)	MAPE (%)
f-50-500_T2300	500	2300	1891	0.9926	343.6	266.3	43.6
f-50-500_T2500	500	2500	1892	0.9996	194.1	154.1	8.3
f-50-500_T2700	500	2700	1895	0.9997	418.2	310.7	7.6
f-50-500_T2900	500	2900	1893	0.9999	328.2	279.9	3.4
f-50-700_T1700	700	1700	1154	0.0000	1.4	1.4	141.8
f-50-700_T1900	700	1900	1812	0.8903	53.2	39.4	59.4
f-50-700_T2100	700	2100	1874	0.9920	61.6	44.2	126.0
f-50-700_T2500	700	2500	1894	0.9996	233.5	153.1	7.7
f-50-700_T2700	700	2700	1893	0.9996	463.2	349.6	6.1
f-50-700_T2900	700	2900	1894	1.0000	269.9	218.2	4.1
f-50-900_T2100	900	2100	1876	0.9669	156.3	83.1	49.8
f-50-900_T2500	900	2500	1884	0.9996	202.2	139.2	24.1
f-50-900_T2900	900	2900	1893	0.9999	318.8	239.7	6.2
**Repeat: strat46**	**Case: A**	**Model: RF**					
f-50-1_T1700	1	1700	357	0.0000	11.5	2.7	271.3
f-50-1_T1900	1	1900	1318	0.0000	2.6	1.7	170.2
f-50-1_T2100	1	2100	1816	0.9533	70.4	53.3	95.9
f-50-1_T2500	1	2500	1892	0.9984	351.5	272.7	8.1
f-50-1_T2700	1	2700	1893	0.9997	346.7	279.2	6.1
f-50-1_T2900	1	2900	1892	0.9995	861.2	672.4	2.7
f-50-100_T1500	100	1500	175	0.0000	2.0	1.5	145.8
f-50-100_T1700	100	1700	663	0.0000	4.5	1.9	190.5
f-50-100_T1900	100	1900	1458	0.0085	13.2	5.6	141.3
f-50-100_T2100	100	2100	1827	0.9819	137.9	100.8	54.0
f-50-100_T2500	100	2500	1891	0.9998	166.9	116.3	9.2
f-50-100_T2700	100	2700	1893	0.9999	203.5	171.5	4.1
f-50-100_T2900	100	2900	1893	0.9999	453.7	355.8	2.6
f-50-300_T1500	300	1500	408	0.0000	1.4	1.4	142.7
f-50-300_T1700	300	1700	1215	0.0000	4.9	3.0	301.5
f-50-300_T1900	300	1900	1562	-3710.7	40.8	23.9	2267
f-50-300_T2100	300	2100	1869	0.9796	69.8	51.1	71.8
f-50-300_T2300	300	2300	1889	0.9957	215.8	192.5	18.0
f-50-300_T2500	300	2500	1894	0.9998	169.3	141.3	24.6
f-50-300_T2700	300	2700	1893	0.9998	416.7	288.4	5.5
f-50-300_T2900	300	2900	1895	0.9997	821.3	452.7	3.9
f-50-500_T1500	500	1500	434	0.0000	2.8	2.8	280.4

continued on next page

Table S12 (continued)

Simulation	$n(O_2)$	T (K)	n_{rows}	R^2	RMSE (molecule·ps)	MAE (molecule·ps)	MAPE (%)
f-50-500_T2100	500	2100	1886	0.9935	54.9	43.3	127.5
f-50-500_T2300	500	2300	1891	0.9947	290.6	240.9	39.3
f-50-500_T2500	500	2500	1892	0.9997	165.0	143.1	7.2
f-50-500_T2700	500	2700	1895	0.9998	315.2	262.2	6.8
f-50-500_T2900	500	2900	1893	1.0000	261.0	235.0	2.8
f-50-700_T1700	700	1700	1154	0.0000	1.4	1.4	142.7
f-50-700_T1900	700	1900	1812	0.9074	48.9	37.3	58.1
f-50-700_T2100	700	2100	1874	0.9971	37.0	28.3	109.1
f-50-700_T2500	700	2500	1894	0.9999	133.6	88.4	5.7
f-50-700_T2700	700	2700	1893	0.9998	331.0	279.0	5.2
f-50-700_T2900	700	2900	1894	1.0000	211.6	166.4	3.5
f-50-900_T2100	900	2100	1876	0.9747	136.8	74.7	79.8
f-50-900_T2500	900	2500	1884	0.9999	104.8	76.7	20.9
f-50-900_T2900	900	2900	1893	1.0000	256.6	210.7	5.8
**Repeat: strat46 Case: A Model: XGBoost**							
f-50-1_T1700	1	1700	357	0.0000	11.3	2.4	240.1
f-50-1_T1900	1	1900	1318	0.0000	2.5	2.1	205.3
f-50-1_T2100	1	2100	1816	0.7921	148.6	124.5	117.5
f-50-1_T2500	1	2500	1892	0.9985	339.4	296.4	14.3
f-50-1_T2700	1	2700	1893	0.9998	332.4	253.3	5.3
f-50-1_T2900	1	2900	1892	0.9996	844.5	600.7	2.7
f-50-100_T1500	100	1500	175	0.0000	4.1	1.3	130.7
f-50-100_T1700	100	1700	663	0.0000	2.6	1.5	147.0
f-50-100_T1900	100	1900	1458	0.6366	8.0	4.2	199.7
f-50-100_T2100	100	2100	1827	0.9963	62.2	47.3	43.3
f-50-100_T2500	100	2500	1891	0.9997	178.1	140.1	8.6
f-50-100_T2700	100	2700	1893	0.9995	463.8	359.9	4.2
f-50-100_T2900	100	2900	1893	0.9995	987.9	617.3	2.6
f-50-300_T1500	300	1500	408	0.0000	0.6	0.4	39.2
f-50-300_T1700	300	1700	1215	0.0000	2.7	2.0	197.4
f-50-300_T1900	300	1900	1562	-2081.1	30.5	18.6	1780
f-50-300_T2100	300	2100	1869	0.9442	115.4	82.0	77.3
f-50-300_T2300	300	2300	1889	0.9966	193.2	162.7	18.7
f-50-300_T2500	300	2500	1894	0.9999	136.7	102.0	20.3
f-50-300_T2700	300	2700	1893	0.9996	507.7	387.3	6.6
f-50-300_T2900	300	2900	1895	0.9986	1735	982.1	3.6

continued on next page

Table S12 (continued)

Simulation	$n(O_2)$	T (K)	n_{rows}	R^2	RMSE (molecule·ps)	MAE (molecule·ps)	MAPE (%)
f-50-500_T1500	500	1500	434	0.0000	1.6	1.4	143.2
f-50-500_T2100	500	2100	1886	0.9944	50.9	38.9	135.3
f-50-500_T2300	500	2300	1891	0.9981	172.6	117.2	26.0
f-50-500_T2500	500	2500	1892	0.9996	194.9	162.1	5.2
f-50-500_T2700	500	2700	1895	0.9997	408.0	277.6	7.6
f-50-500_T2900	500	2900	1893	0.9994	1014	565.2	2.2
f-50-700_T1700	700	1700	1154	0.0000	7.4	6.4	643.5
f-50-700_T1900	700	1900	1812	0.9654	29.9	21.1	196.0
f-50-700_T2100	700	2100	1874	0.9971	36.7	25.9	61.3
f-50-700_T2500	700	2500	1894	0.9998	167.7	127.4	4.9
f-50-700_T2700	700	2700	1893	0.9995	486.3	380.9	5.2
f-50-700_T2900	700	2900	1894	0.9998	663.0	413.0	2.9
f-50-900_T2100	900	2100	1876	0.9823	114.4	102.1	1683
f-50-900_T2500	900	2500	1884	0.9995	220.9	173.2	16.9
f-50-900_T2900	900	2900	1893	0.9993	1082	864.0	6.8
**Repeat: strat46**	**Case: A**	**Model: CatBoost**					
f-50-1_T1700	1	1700	357	0.0000	30.9	22.3	2229
f-50-1_T1900	1	1900	1318	0.0000	43.1	33.3	3327
f-50-1_T2100	1	2100	1816	0.8765	114.5	88.9	193.5
f-50-1_T2500	1	2500	1892	0.9964	527.1	411.2	12.5
f-50-1_T2700	1	2700	1893	0.9991	642.3	486.7	4.2
f-50-1_T2900	1	2900	1892	0.9987	1446	968.8	4.1
f-50-100_T1500	100	1500	175	0.0000	20.3	15.0	1502
f-50-100_T1700	100	1700	663	0.0000	34.8	28.4	2844
f-50-100_T1900	100	1900	1458	-2.6744	25.5	21.1	1640
f-50-100_T2100	100	2100	1827	0.8851	347.2	288.0	49.6
f-50-100_T2500	100	2500	1891	0.9993	284.9	209.3	7.1
f-50-100_T2700	100	2700	1893	0.9994	512.4	391.3	4.4
f-50-100_T2900	100	2900	1893	0.9993	1149	820.6	4.8
f-50-300_T1500	300	1500	408	0.0000	63.1	53.9	5388
f-50-300_T1700	300	1700	1215	0.0000	51.4	35.0	3499
f-50-300_T1900	300	1900	1562	-29,369.1	114.7	96.4	9374
f-50-300_T2100	300	2100	1869	0.9699	84.8	70.1	216.5
f-50-300_T2300	300	2300	1889	0.9930	276.5	214.6	16.7
f-50-300_T2500	300	2500	1894	0.9995	263.9	199.3	17.1
f-50-300_T2700	300	2700	1893	0.9986	978.6	527.3	7.2

continued on next page

Table S12 (continued)

Simulation	$n(O_2)$	T (K)	n_{rows}	R^2	RMSE (molecule·ps)	MAE (molecule·ps)	MAPE (%)
f-50-300_T2900	300	2900	1895	0.9968	2598	1687	5.9
f-50-500_T1500	500	1500	434	0.0000	23.9	17.3	1731
f-50-500_T2100	500	2100	1886	0.9479	155.7	125.3	691.5
f-50-500_T2300	500	2300	1891	0.9960	250.9	194.6	77.8
f-50-500_T2500	500	2500	1892	0.9985	388.0	297.5	7.9
f-50-500_T2700	500	2700	1895	0.9995	544.5	405.9	9.8
f-50-500_T2900	500	2900	1893	0.9995	997.4	808.0	4.0
f-50-700_T1700	700	1700	1154	0.0000	124.5	109.9	10,991
f-50-700_T1900	700	1900	1812	0.6977	88.3	76.9	1831
f-50-700_T2100	700	2100	1874	0.9721	114.7	84.7	320.9
f-50-700_T2500	700	2500	1894	0.9988	390.6	277.8	17.5
f-50-700_T2700	700	2700	1893	0.9993	566.9	405.7	5.5
f-50-700_T2900	700	2900	1894	0.9996	878.2	695.5	6.0
f-50-900_T2100	900	2100	1876	0.9702	148.2	115.3	2052
f-50-900_T2500	900	2500	1884	0.9951	677.8	540.8	47.8
f-50-900_T2900	900	2900	1893	0.9985	1603	1155	9.6
**Repeat: strat46**	**Case: A**	**Model: ANN**					
f-50-1_T1700	1	1700	357	0.0000	81.3	61.7	6166
f-50-1_T1900	1	1900	1318	0.0000	89.3	77.7	7767
f-50-1_T2100	1	2100	1816	-2.1982	582.8	301.5	413.9
f-50-1_T2500	1	2500	1892	0.9942	663.4	601.4	55.6
f-50-1_T2700	1	2700	1893	0.9995	474.6	368.7	8.6
f-50-1_T2900	1	2900	1892	0.9997	730.5	550.9	9.3
f-50-100_T1500	100	1500	175	0.0000	37.5	24.8	2484
f-50-100_T1700	100	1700	663	0.0000	100.6	84.8	8483
f-50-100_T1900	100	1900	1458	-6.1756	35.6	23.6	1362
f-50-100_T2100	100	2100	1827	0.5039	721.5	595.4	96.6
f-50-100_T2500	100	2500	1891	0.9967	617.5	501.9	10.3
f-50-100_T2700	100	2700	1893	0.9997	364.0	307.2	7.2
f-50-100_T2900	100	2900	1893	0.9997	693.0	552.5	4.8
f-50-300_T1500	300	1500	408	0.0000	130.8	97.0	9697
f-50-300_T1700	300	1700	1215	0.0000	100.0	81.4	8141
f-50-300_T1900	300	1900	1562	-5010.7	47.4	42.1	4133
f-50-300_T2100	300	2100	1869	0.0846	467.4	315.1	382.1
f-50-300_T2300	300	2300	1889	0.9178	944.8	724.7	43.2
f-50-300_T2500	300	2500	1894	0.9996	237.2	180.6	128.1

continued on next page

Table S12 (continued)

Simulation	$n(O_2)$	T (K)	n_{rows}	R^2	RMSE (molecule·ps)	MAE (molecule·ps)	MAPE (%)
f-50-300_T2700	300	2700	1893	0.9995	612.5	464.4	5.1
f-50-300_T2900	300	2900	1895	0.9998	583.4	524.7	5.6
f-50-500_T1500	500	1500	434	0.0000	60.0	44.1	4407
f-50-500_T2100	500	2100	1886	0.8951	220.9	174.4	203.6
f-50-500_T2300	500	2300	1891	0.9891	416.8	302.3	36.5
f-50-500_T2500	500	2500	1892	0.9995	225.0	173.6	8.3
f-50-500_T2700	500	2700	1895	0.9995	548.1	406.4	5.4
f-50-500_T2900	500	2900	1893	0.9996	905.7	667.3	4.1
f-50-700_T1700	700	1700	1154	0.0000	936.9	805.5	80,554
f-50-700_T1900	700	1900	1812	-19.0513	718.9	635.1	5456
f-50-700_T2100	700	2100	1874	0.9805	95.7	74.4	648.7
f-50-700_T2500	700	2500	1894	0.9966	663.7	535.0	15.2
f-50-700_T2700	700	2700	1893	0.9995	477.1	377.1	6.8
f-50-700_T2900	700	2900	1894	0.9998	632.4	556.3	5.1
f-50-900_T2100	900	2100	1876	0.9646	161.6	126.5	2939
f-50-900_T2500	900	2500	1884	0.9979	447.1	327.5	21.5
f-50-900_T2900	900	2900	1893	0.9996	769.6	626.7	12.9
**Repeat: strat46**	**Case: A**	**Model: SVR**					
f-50-1_T1700	1	1700	357	0.0000	1155	998.7	99,873
f-50-1_T1900	1	1900	1318	0.0000	1306	1182	118,246
f-50-1_T2100	1	2100	1816	-22.5037	1580	1120	6856
f-50-1_T2500	1	2500	1892	0.8900	2901	2372	162.3
f-50-1_T2700	1	2700	1893	0.7077	11,715	6859	44.7
f-50-1_T2900	1	2900	1892	0.1575	37,130	24,706	46.0
f-50-100_T1500	100	1500	175	0.0000	647.9	431.0	43,096
f-50-100_T1700	100	1700	663	0.0000	924.0	693.3	69,326
f-50-100_T1900	100	1900	1458	-1610.7	533.2	372.1	21,345
f-50-100_T2100	100	2100	1827	-0.7104	1340	1017	772.4
f-50-100_T2500	100	2500	1891	0.9862	1261	782.9	82.9
f-50-100_T2700	100	2700	1893	0.8315	8942	5887	77.4
f-50-100_T2900	100	2900	1893	0.1306	40,744	25,977	43.0
f-50-300_T1500	300	1500	408	0.0000	949.0	753.3	75,330
f-50-300_T1700	300	1700	1215	0.0000	415.6	336.9	33,694
f-50-300_T1900	300	1900	1562	-474,068.5	461.0	309.3	29,190
f-50-300_T2100	300	2100	1869	-15.5900	1990	1399	5871
f-50-300_T2300	300	2300	1889	0.7274	1721	1272	222.1

continued on next page

Table S12 (continued)

Simulation	$n(O_2)$	T (K)	n_{rows}	R^2	RMSE (molecule·ps)	MAE (molecule·ps)	MAPE (%)
f-50-300_T2500	300	2500	1894	0.9146	3295	2467	1029
f-50-300_T2700	300	2700	1893	0.8102	11,521	6842	70.1
f-50-300_T2900	300	2900	1895	0.1600	41,925	27,437	33.9
f-50-500_T1500	500	1500	434	0.0000	402.0	362.4	36,243
f-50-500_T2100	500	2100	1886	-2.1299	1207	861.3	4133
f-50-500_T2300	500	2300	1891	0.7575	1964	1625	428.6
f-50-500_T2500	500	2500	1892	0.9670	1851	1402	48.4
f-50-500_T2700	500	2700	1895	0.7525	12,529	8034	91.5
f-50-500_T2900	500	2900	1893	0.2016	38,642	25,171	58.9
f-50-700_T1700	700	1700	1154	0.0000	4920	4345	434,535
f-50-700_T1900	700	1900	1812	-487.3	3548	2961	15,784
f-50-700_T2100	700	2100	1874	-4.3994	1595	1213	8599
f-50-700_T2500	700	2500	1894	0.9144	3315	1946	85.9
f-50-700_T2700	700	2700	1893	0.6962	12,188	7616	38.4
f-50-700_T2900	700	2900	1894	0.1473	39,332	26,531	40.2
f-50-900_T2100	900	2100	1876	-29.3610	4733	3916	35,315
f-50-900_T2500	900	2500	1884	0.8914	3198	2517	545.7
f-50-900_T2900	900	2900	1893	-0.0769	42,663	28,911	191.6
**Repeat: strat46**	**Case: A**	**Model: GPR**					
f-50-1_T1700	1	1700	357	0.0000	21.8	14.4	1441
f-50-1_T1900	1	1900	1318	0.0000	19.9	15.5	1553
f-50-1_T2100	1	2100	1816	0.9509	72.2	61.4	116.2
f-50-1_T2500	1	2500	1892	0.9995	198.5	149.7	16.0
f-50-1_T2700	1	2700	1893	0.9999	200.7	168.5	3.7
f-50-1_T2900	1	2900	1892	0.9999	422.3	381.1	2.6
f-50-100_T1500	100	1500	175	0.0000	16.0	8.4	844.4
f-50-100_T1700	100	1700	663	0.0000	6.6	4.2	420.2
f-50-100_T1900	100	1900	1458	-1.5348	21.1	17.3	1248
f-50-100_T2100	100	2100	1827	0.9864	119.3	99.4	27.5
f-50-100_T2500	100	2500	1891	0.9999	95.2	82.7	5.1
f-50-100_T2700	100	2700	1893	0.9999	210.6	165.1	3.0
f-50-100_T2900	100	2900	1893	1.0000	281.6	250.7	2.1
f-50-300_T1500	300	1500	408	0.0000	7.2	6.5	654.0
f-50-300_T1700	300	1700	1215	0.0000	14.7	12.3	1232
f-50-300_T1900	300	1900	1562	-3461.5	39.4	33.5	3337
f-50-300_T2100	300	2100	1869	0.9765	74.9	61.8	296.0

continued on next page

Table S12 (continued)

Simulation	$n(O_2)$	T (K)	n_{rows}	R^2	RMSE (molecule·ps)	MAE (molecule·ps)	MAPE (%)
f-50-300_T2300	300	2300	1889	0.9981	144.6	106.7	11.7
f-50-300_T2500	300	2500	1894	0.9998	150.0	115.2	23.9
f-50-300_T2700	300	2700	1893	0.9999	250.0	211.8	5.9
f-50-300_T2900	300	2900	1895	1.0000	286.8	246.2	3.8
f-50-500_T1500	500	1500	434	0.0000	9.5	6.9	690.8
f-50-500_T2100	500	2100	1886	0.9849	83.8	61.8	199.9
f-50-500_T2300	500	2300	1891	0.9946	292.8	204.9	46.2
f-50-500_T2500	500	2500	1892	0.9998	126.8	91.4	3.3
f-50-500_T2700	500	2700	1895	1.0000	141.9	110.5	6.8
f-50-500_T2900	500	2900	1893	0.9999	390.7	350.5	2.2
f-50-700_T1700	700	1700	1154	0.0000	170.9	147.7	14,769
f-50-700_T1900	700	1900	1812	0.4402	120.1	100.9	4133
f-50-700_T2100	700	2100	1874	0.9914	63.5	53.7	258.3
f-50-700_T2500	700	2500	1894	0.9998	173.8	142.5	6.7
f-50-700_T2700	700	2700	1893	0.9999	252.5	188.5	4.6
f-50-700_T2900	700	2900	1894	1.0000	213.6	181.4	3.3
f-50-900_T2100	900	2100	1876	0.9650	160.7	134.9	5798
f-50-900_T2500	900	2500	1884	0.9975	486.2	419.3	10.7
f-50-900_T2900	900	2900	1893	0.9997	653.4	604.7	8.8
**Repeat: strat46 Case: A Model: persistence**							
f-50-1_T1700	1	1700	357	1.0000	0.0	0.0	0.0
f-50-1_T1900	1	1900	1318	1.0000	0.0	0.0	0.0
f-50-1_T2100	1	2100	1816	0.9631	62.6	54.9	23.7
f-50-1_T2500	1	2500	1892	0.9646	1647	1468	28.5
f-50-1_T2700	1	2700	1893	0.9644	4090	3642	27.3
f-50-1_T2900	1	2900	1892	0.9666	7390	6812	25.5
f-50-100_T1500	100	1500	175	1.0000	0.0	0.0	0.0
f-50-100_T1700	100	1700	663	1.0000	0.0	0.0	0.0
f-50-100_T1900	100	1900	1458	0.5926	8.5	3.1	11.9
f-50-100_T2100	100	2100	1827	0.9585	208.7	176.9	28.0
f-50-100_T2500	100	2500	1891	0.9636	2046	1794	29.2
f-50-100_T2700	100	2700	1893	0.9633	4174	3673	26.9
f-50-100_T2900	100	2900	1893	0.9656	8105	7371	25.8
f-50-300_T1500	300	1500	408	1.0000	0.0	0.0	0.0
f-50-300_T1700	300	1700	1215	1.0000	0.0	0.0	0.0
f-50-300_T1900	300	1900	1562	0.0667	0.6	0.1	3.4

continued on next page

Table S12 (continued)

Simulation	$n(O_2)$	T (K)	n_{rows}	R^2	RMSE (molecule·ps)	MAE (molecule·ps)	MAPE (%)
f-50-300_T2100	300	2100	1869	0.9635	93.4	80.0	24.3
f-50-300_T2300	300	2300	1889	0.9586	670.4	572.4	28.8
f-50-300_T2500	300	2500	1894	0.9609	2228	1892	31.4
f-50-300_T2700	300	2700	1893	0.9631	5080	4441	28.5
f-50-300_T2900	300	2900	1895	0.9655	8494	7665	26.3
f-50-500_T1500	500	1500	434	1.0000	0.0	0.0	0.0
f-50-500_T2100	500	2100	1886	0.9467	157.5	113.8	27.3
f-50-500_T2300	500	2300	1891	0.9589	808.1	663.3	31.3
f-50-500_T2500	500	2500	1892	0.9581	2085	1751	28.4
f-50-500_T2700	500	2700	1895	0.9622	4897	4216	29.2
f-50-500_T2900	500	2900	1893	0.9655	8037	7244	27.0
f-50-700_T1700	700	1700	1154	1.0000	0.0	0.0	0.0
f-50-700_T1900	700	1900	1812	0.9550	34.0	27.5	21.3
f-50-700_T2100	700	2100	1874	0.9562	143.7	115.1	27.1
f-50-700_T2500	700	2500	1894	0.9619	2213	1916	28.9
f-50-700_T2700	700	2700	1893	0.9621	4303	3713	28.5
f-50-700_T2900	700	2900	1894	0.9644	8040	7131	27.6
f-50-900_T2100	900	2100	1876	0.9632	164.8	133.9	23.0
f-50-900_T2500	900	2500	1884	0.9600	1940	1651	31.2
f-50-900_T2900	900	2900	1893	0.9640	7798	6850	28.8
**Repeat: strat46 Case: A Model: physical_MLR**							
f-50-1_T1700	1	1700	357	0.0000	20,821	16,876	1,687,579
f-50-1_T1900	1	1900	1318	0.0000	14,858	12,621	1,262,053
f-50-1_T2100	1	2100	1816	-1844.0	13,998	11,869	135,795
f-50-1_T2500	1	2500	1892	-1.9443	15,010	13,381	434.5
f-50-1_T2700	1	2700	1893	0.8125	9383	8531	242.4
f-50-1_T2900	1	2900	1892	0.4452	30,131	23,328	180.9
f-50-100_T1500	100	1500	175	0.0000	28,730	24,485	2,448,501
f-50-100_T1700	100	1700	663	0.0000	17,855	14,384	1,438,423
f-50-100_T1900	100	1900	1458	-1.23×10^6	14,742	12,479	977,990
f-50-100_T2100	100	2100	1827	-164.3	13,170	11,279	27,685
f-50-100_T2500	100	2500	1891	0.0758	10,308	9517	301.8
f-50-100_T2700	100	2700	1893	0.7308	11,302	10,322	283.9
f-50-100_T2900	100	2900	1893	0.4302	32,985	24,805	165.3
f-50-300_T1500	300	1500	408	0.0000	29,488	26,077	2,607,651
f-50-300_T1700	300	1700	1215	0.0000	19,241	15,171	1,517,095

continued on next page

Table S12 (continued)

Simulation	$n(O_2)$	T (K)	n_{rows}	R^2	RMSE (molecule·ps)	MAE (molecule·ps)	MAPE (%)
f-50-300_T1900	300	1900	1562	-4.80×10^8	14,667	12,096	1,186,258
f-50-300_T2100	300	2100	1869	-982.9	15,325	13,138	278,504
f-50-300_T2300	300	2300	1889	-20.3800	15,237	12,824	4280
f-50-300_T2500	300	2500	1894	-0.0113	11,335	10,320	2013
f-50-300_T2700	300	2700	1893	0.7793	12,424	10,417	272.0
f-50-300_T2900	300	2900	1895	0.4166	34,941	26,454	182.5
f-50-500_T1500	500	1500	434	0.0000	27,270	23,414	2,341,387
f-50-500_T2100	500	2100	1886	-447.2	14,438	12,483	299,089
f-50-500_T2300	500	2300	1891	-12.3838	14,590	12,438	7545
f-50-500_T2500	500	2500	1892	-0.4857	12,418	11,148	315.8
f-50-500_T2700	500	2700	1895	0.7511	12,565	11,016	435.0
f-50-500_T2900	500	2900	1893	0.4150	33,077	25,445	256.3
f-50-700_T1700	700	1700	1154	0.0000	20,178	15,687	1,568,698
f-50-700_T1900	700	1900	1812	-12,140.6	17,689	14,532	683,827
f-50-700_T2100	700	2100	1874	-466.5	14,842	12,791	237,503
f-50-700_T2500	700	2500	1894	0.2203	10,007	9101	340.4
f-50-700_T2700	700	2700	1893	0.7959	9990	9130	290.4
f-50-700_T2900	700	2900	1894	0.5122	29,749	22,929	270.8
f-50-900_T2100	900	2100	1876	-287.8	14,598	12,499	545,043
f-50-900_T2500	900	2500	1884	-0.5807	12,197	11,200	586.3
f-50-900_T2900	900	2900	1893	0.5221	28,419	21,779	370.5
**Repeat: strat46 Case: A Model: physical_RF**							
f-50-1_T1700	1	1700	357	0.0000	0.0	0.0	0.1
f-50-1_T1900	1	1900	1318	0.0000	14.2	5.0	500.4
f-50-1_T2100	1	2100	1816	0.5045	229.4	167.4	45.6
f-50-1_T2500	1	2500	1892	0.8896	2907	1971	14.6
f-50-1_T2700	1	2700	1893	0.9955	1447	589.9	4.7
f-50-1_T2900	1	2900	1892	0.9868	4653	2804	4.4
f-50-100_T1500	100	1500	175	0.0000	0.0	0.0	0.4
f-50-100_T1700	100	1700	663	0.0000	1.1	0.3	33.1
f-50-100_T1900	100	1900	1458	-0.1162	14.0	4.9	22.2
f-50-100_T2100	100	2100	1827	0.3863	802.5	594.3	58.7
f-50-100_T2500	100	2500	1891	0.9610	2118	1531	10.9
f-50-100_T2700	100	2700	1893	0.9968	1231	667.0	6.2
f-50-100_T2900	100	2900	1893	0.9901	4339	2713	4.8
f-50-300_T1500	300	1500	408	0.0000	0.0	0.0	1.4

continued on next page

Table S12 (continued)

Simulation	$n(O_2)$	T (K)	n_{rows}	R^2	RMSE (molecule·ps)	MAE (molecule·ps)	MAPE (%)
f-50-300_T1700	300	1700	1215	0.0000	0.2	0.1	5.4
f-50-300_T1900	300	1900	1562	-408.7	13.6	4.8	414.6
f-50-300_T2100	300	2100	1869	-1.5981	787.5	575.7	481.3
f-50-300_T2300	300	2300	1889	0.9426	789.7	623.8	25.3
f-50-300_T2500	300	2500	1894	0.9942	861.5	537.8	38.0
f-50-300_T2700	300	2700	1893	0.9354	6721	4881	16.6
f-50-300_T2900	300	2900	1895	0.9948	3314	2477	5.6
f-50-500_T1500	500	1500	434	0.0000	0.3	0.1	6.2
f-50-500_T2100	500	2100	1886	0.8605	254.8	180.9	87.2
f-50-500_T2300	500	2300	1891	0.9604	793.9	629.9	45.9
f-50-500_T2500	500	2500	1892	0.9692	1789	1398	13.7
f-50-500_T2700	500	2700	1895	0.9876	2805	2339	13.2
f-50-500_T2900	500	2900	1893	0.9905	4222	3361	11.5
f-50-700_T1700	700	1700	1154	0.0000	0.0	0.0	2.3
f-50-700_T1900	700	1900	1812	-1.1424	235.0	171.8	80.4
f-50-700_T2100	700	2100	1874	0.9676	123.5	95.4	38.1
f-50-700_T2500	700	2500	1894	0.9622	2204	1805	15.8
f-50-700_T2700	700	2700	1893	0.9572	4575	3393	12.6
f-50-700_T2900	700	2900	1894	0.9945	3166	2721	11.5
f-50-900_T2100	900	2100	1876	0.8893	285.8	212.7	428.1
f-50-900_T2500	900	2500	1884	0.9175	2787	2258	46.3
f-50-900_T2900	900	2900	1893	0.9916	3764	3203	18.0
**Repeat: strat46**	**Case: B**	**Model: MLR**					
f-50-1_T1700	1	1700	357	0.0000	19,841	18,266	1,826,566
f-50-1_T1900	1	1900	1318	0.0000	22,571	21,097	2,109,744
f-50-1_T2100	1	2100	1816	-2381.4	15,907	15,426	72,915
f-50-1_T2500	1	2500	1892	0.7349	4504	4132	694.8
f-50-1_T2700	1	2700	1893	0.5154	15,084	12,644	200.2
f-50-1_T2900	1	2900	1892	0.1123	38,113	30,237	121.6
f-50-100_T1500	100	1500	175	0.0000	14,864	12,915	1,291,527
f-50-100_T1700	100	1700	663	0.0000	17,968	16,294	1,629,363
f-50-100_T1900	100	1900	1458	-2.00×10^6	18,776	17,093	1,306,173
f-50-100_T2100	100	2100	1827	-171.0	13,433	12,673	7425
f-50-100_T2500	100	2500	1891	0.9567	2232	1719	299.0
f-50-100_T2700	100	2700	1893	0.9462	5051	3901	221.0
f-50-100_T2900	100	2900	1893	0.3758	34,525	23,743	67.3

continued on next page

Table S12 (continued)

Simulation	$n(O_2)$	T (K)	n_{rows}	R^2	RMSE (molecule·ps)	MAE (molecule·ps)	MAPE (%)
f-50-300_T1500	300	1500	408	0.0000	20,862	17,343	1,734,324
f-50-300_T1700	300	1700	1215	0.0000	12,014	10,061	1,006,150
f-50-300_T1900	300	1900	1562	-3.24×10^8	12,058	9997	969,416
f-50-300_T2100	300	2100	1869	-1816.0	20,825	16,888	50,813
f-50-300_T2300	300	2300	1889	-34.3627	19,596	16,502	2409
f-50-300_T2500	300	2500	1894	0.4848	8090	6420	8484
f-50-300_T2700	300	2700	1893	0.7099	14,243	11,612	288.0
f-50-300_T2900	300	2900	1895	0.3718	36,257	25,921	115.1
f-50-500_T1500	500	1500	434	0.0000	7887	6782	678,240
f-50-500_T2100	500	2100	1886	-985.9	21,425	17,976	205,004
f-50-500_T2300	500	2300	1891	-29.4350	22,002	19,669	8862
f-50-500_T2500	500	2500	1892	-2.4217	18,845	17,789	1208
f-50-500_T2700	500	2700	1895	0.5833	16,259	14,046	682.3
f-50-500_T2900	500	2900	1893	0.1477	39,924	29,076	208.1
f-50-700_T1700	700	1700	1154	0.0000	36,621	32,413	3,241,254
f-50-700_T1900	700	1900	1812	-21,298.1	23,429	21,933	223,129
f-50-700_T2100	700	2100	1874	-959.1	21,270	18,727	275,771
f-50-700_T2500	700	2500	1894	-0.9924	15,996	14,946	2005
f-50-700_T2700	700	2700	1893	0.4317	16,671	14,074	903.0
f-50-700_T2900	700	2900	1894	-0.1599	45,875	34,371	356.8
f-50-900_T2100	900	2100	1876	-234.7	13,188	10,578	559,249
f-50-900_T2500	900	2500	1884	-1.6294	15,731	14,118	7869
f-50-900_T2900	900	2900	1893	0.6288	25,048	20,469	1409
**Repeat: strat46 Case: B Model: DT**							
f-50-1_T1700	1	1700	357	1.0000	0.0	0.0	0.0
f-50-1_T1900	1	1900	1318	0.0000	0.6	0.0	2.7
f-50-1_T2100	1	2100	1816	-821.5	9347	1382	215.1
f-50-1_T2500	1	2500	1892	-0.0501	8964	7065	82.3
f-50-1_T2700	1	2700	1893	-0.0836	22,555	15,895	54.5
f-50-1_T2900	1	2900	1892	0.4509	29,975	22,630	47.6
f-50-100_T1500	100	1500	175	0.0000	11.3	0.9	85.7
f-50-100_T1700	100	1700	663	0.0000	7.5	0.3	29.0
f-50-100_T1900	100	1900	1458	-34.6753	79.3	21.7	51.3
f-50-100_T2100	100	2100	1827	0.0102	1019	739.6	104.6
f-50-100_T2500	100	2500	1891	0.6135	6666	5166	79.2
f-50-100_T2700	100	2700	1893	-0.0682	22,515	16,196	78.9

continued on next page

Table S12 (continued)

Simulation	$n(O_2)$	T (K)	n_{rows}	R^2	RMSE (molecule·ps)	MAE (molecule·ps)	MAPE (%)
f-50-100_T2900	100	2900	1893	0.5992	27,664	19,793	31.8
f-50-300_T1500	300	1500	408	1.0000	0.0	0.0	0.0
f-50-300_T1700	300	1700	1215	0.0000	552.2	158.9	15,888
f-50-300_T1900	300	1900	1562	-3.09×10^6	1178	759.3	73,446
f-50-300_T2100	300	2100	1869	-1340.8	17,896	5667	1073
f-50-300_T2300	300	2300	1889	-1.3795	5083	3184	68.4
f-50-300_T2500	300	2500	1894	-2.8362	22,076	14,814	98.8
f-50-300_T2700	300	2700	1893	0.1836	23,895	16,245	46.0
f-50-300_T2900	300	2900	1895	0.4606	33,596	24,429	39.1
f-50-500_T1500	500	1500	434	1.0000	0.0	0.0	0.0
f-50-500_T2100	500	2100	1886	-32.8988	3971	2489	290.4
f-50-500_T2300	500	2300	1891	-2.3802	7332	4638	114.6
f-50-500_T2500	500	2500	1892	0.4899	7277	5152	44.0
f-50-500_T2700	500	2700	1895	0.2855	21,290	14,883	46.7
f-50-500_T2900	500	2900	1893	0.1940	38,826	26,238	49.5
f-50-700_T1700	700	1700	1154	0.0000	14,850	6593	659,342
f-50-700_T1900	700	1900	1812	-8931.5	15,173	5992	31,519
f-50-700_T2100	700	2100	1874	-2.3669	1260	514.1	85.0
f-50-700_T2500	700	2500	1894	0.5067	7959	4585	36.6
f-50-700_T2700	700	2700	1893	0.5340	15,095	9267	25.5
f-50-700_T2900	700	2900	1894	0.5567	28,359	20,488	35.2
f-50-900_T2100	900	2100	1876	-555.1	20,257	10,603	103,717
f-50-900_T2500	900	2500	1884	-0.1724	10,504	6051	59.6
f-50-900_T2900	900	2900	1893	0.9279	11,035	7787	31.7
**Repeat: strat46 Case: B Model: RF**							
f-50-1_T1700	1	1700	357	0.0000	1343	789.5	78,951
f-50-1_T1900	1	1900	1318	0.0000	773.2	515.9	51,591
f-50-1_T2100	1	2100	1816	-805.7	9256	5353	829.5
f-50-1_T2500	1	2500	1892	0.5030	6167	4914	57.0
f-50-1_T2700	1	2700	1893	0.9280	5813	4230	19.3
f-50-1_T2900	1	2900	1892	0.7170	21,519	14,441	25.3
f-50-100_T1500	100	1500	175	0.0000	4.5	2.0	201.1
f-50-100_T1700	100	1700	663	0.0000	908.8	566.2	56,619
f-50-100_T1900	100	1900	1458	-47,603.8	2898	1971	119,974
f-50-100_T2100	100	2100	1827	-13.3857	3885	1763	136.8
f-50-100_T2500	100	2500	1891	-0.1142	11,318	8403	73.7

continued on next page

Table S12 (continued)

Simulation	$n(O_2)$	T (K)	n_{rows}	R^2	RMSE (molecule·ps)	MAE (molecule·ps)	MAPE (%)
f-50-100_T2700	100	2700	1893	0.9016	6833	5432	28.9
f-50-100_T2900	100	2900	1893	0.7031	23,812	16,463	26.0
f-50-300_T1500	300	1500	408	0.0000	707.5	444.3	44,428
f-50-300_T1700	300	1700	1215	0.0000	382.7	138.7	13,865
f-50-300_T1900	300	1900	1562	-6.85×10^6	1752	1138	105,769
f-50-300_T2100	300	2100	1869	-2055.4	22,155	14,401	1797
f-50-300_T2300	300	2300	1889	-5.4809	8389	6002	124.1
f-50-300_T2500	300	2500	1894	0.8241	4727	4008	124.5
f-50-300_T2700	300	2700	1893	0.5375	17,986	10,813	28.6
f-50-300_T2900	300	2900	1895	0.1129	43,087	29,932	40.2
f-50-500_T1500	500	1500	434	0.0000	3728	3016	301,613
f-50-500_T2100	500	2100	1886	-16.9754	2892	1988	685.3
f-50-500_T2300	500	2300	1891	-0.8182	5378	3169	97.1
f-50-500_T2500	500	2500	1892	0.6790	5772	4230	37.1
f-50-500_T2700	500	2700	1895	0.3211	20,752	14,414	43.1
f-50-500_T2900	500	2900	1893	-0.0572	44,466	33,365	53.8
f-50-700_T1700	700	1700	1154	0.0000	30,597	25,784	2,578,448
f-50-700_T1900	700	1900	1812	-11,546.8	17,251	14,076	47,145
f-50-700_T2100	700	2100	1874	-2.4095	1268	773.3	276.9
f-50-700_T2500	700	2500	1894	0.7099	6104	4090	26.1
f-50-700_T2700	700	2700	1893	0.9190	6293	4253	15.0
f-50-700_T2900	700	2900	1894	0.8211	18,018	13,698	27.5
f-50-900_T2100	900	2100	1876	-68.7007	7172	5568	54,250
f-50-900_T2500	900	2500	1884	-1.8091	16,260	10,978	79.2
f-50-900_T2900	900	2900	1893	0.4483	30,537	17,976	28.1
**Repeat: strat46 Case: B Model: XGBoost**							
f-50-1_T1700	1	1700	357	0.0000	1027	717.5	71,752
f-50-1_T1900	1	1900	1318	0.0000	1200	1006	100,624
f-50-1_T2100	1	2100	1816	-1003.0	10,326	5303	1251
f-50-1_T2500	1	2500	1892	0.2008	7821	6865	195.6
f-50-1_T2700	1	2700	1893	0.7895	9942	5963	38.2
f-50-1_T2900	1	2900	1892	0.7951	18,312	13,649	35.2
f-50-100_T1500	100	1500	175	0.0000	895.7	644.6	64,463
f-50-100_T1700	100	1700	663	0.0000	2482	1385	138,459
f-50-100_T1900	100	1900	1458	-893,175.4	12,551	8217	476,082
f-50-100_T2100	100	2100	1827	-9.9902	3396	1691	159.6

continued on next page

Table S12 (continued)

Simulation	$n(O_2)$	T (K)	n_{rows}	R^2	RMSE (molecule·ps)	MAE (molecule·ps)	MAPE (%)
f-50-100_T2500	100	2500	1891	0.2873	9052	6666	63.3
f-50-100_T2700	100	2700	1893	0.1051	20,608	14,756	57.1
f-50-100_T2900	100	2900	1893	0.8456	17,172	13,473	40.0
f-50-300_T1500	300	1500	408	0.0000	889.9	645.6	64,560
f-50-300_T1700	300	1700	1215	0.0000	6120	3739	373,928
f-50-300_T1900	300	1900	1562	-2.73×10^7	3501	2152	200,909
f-50-300_T2100	300	2100	1869	-974.9	15,262	8086	3190
f-50-300_T2300	300	2300	1889	-7.3134	9501	6579	221.9
f-50-300_T2500	300	2500	1894	0.1445	10,425	7889	564.5
f-50-300_T2700	300	2700	1893	0.2848	22,365	14,965	45.4
f-50-300_T2900	300	2900	1895	0.2322	40,085	26,116	37.3
f-50-500_T1500	500	1500	434	0.0000	1473	898.9	89,890
f-50-500_T2100	500	2100	1886	-13.7370	2618	1722	5117
f-50-500_T2300	500	2300	1891	0.2534	3446	2085	210.9
f-50-500_T2500	500	2500	1892	0.8759	3589	2563	51.2
f-50-500_T2700	500	2700	1895	0.1644	23,023	15,282	46.3
f-50-500_T2900	500	2900	1893	-0.3179	49,646	34,518	53.2
f-50-700_T1700	700	1700	1154	0.0000	19,579	15,886	1,588,556
f-50-700_T1900	700	1900	1812	-2391.1	7852	6533	110,507
f-50-700_T2100	700	2100	1874	-29.2389	3775	2437	5556
f-50-700_T2500	700	2500	1894	0.8724	4049	2613	32.5
f-50-700_T2700	700	2700	1893	0.9731	3626	2449	17.6
f-50-700_T2900	700	2900	1894	0.5644	28,113	17,367	26.0
f-50-900_T2100	900	2100	1876	-28.7603	4686	4024	91,668
f-50-900_T2500	900	2500	1884	-1.0595	13,922	8085	93.2
f-50-900_T2900	900	2900	1893	0.7253	21,549	13,119	51.8
**Repeat: strat46 Case: B Model: CatBoost**							
f-50-1_T1700	1	1700	357	0.0000	1779	1081	108,086
f-50-1_T1900	1	1900	1318	0.0000	844.3	692.6	69,259
f-50-1_T2100	1	2100	1816	-297.4	5629	3333	1235
f-50-1_T2500	1	2500	1892	0.7784	4118	3279	81.2
f-50-1_T2700	1	2700	1893	0.7847	10,053	6436	33.7
f-50-1_T2900	1	2900	1892	0.6887	22,571	18,558	43.7
f-50-100_T1500	100	1500	175	0.0000	1704	933.8	93,379
f-50-100_T1700	100	1700	663	0.0000	1012	660.9	66,088
f-50-100_T1900	100	1900	1458	-262,857.7	6809	3942	168,076

continued on next page

Table S12 (continued)

Simulation	$n(O_2)$	T (K)	n_{rows}	R^2	RMSE (molecule·ps)	MAE (molecule·ps)	MAPE (%)
f-50-100_T2100	100	2100	1827	-8.8647	3217	2363	251.2
f-50-100_T2500	100	2500	1891	-0.3819	12,605	8860	73.4
f-50-100_T2700	100	2700	1893	0.1533	20,046	14,915	59.3
f-50-100_T2900	100	2900	1893	0.9655	8116	6537	22.6
f-50-300_T1500	300	1500	408	0.0000	909.0	705.3	70,532
f-50-300_T1700	300	1700	1215	0.0000	2802	1274	127,424
f-50-300_T1900	300	1900	1562	-9.88×10^6	2104	1458	138,749
f-50-300_T2100	300	2100	1869	-1561.6	19,313	12,142	3379
f-50-300_T2300	300	2300	1889	-6.4025	8966	5843	138.7
f-50-300_T2500	300	2500	1894	0.5059	7923	5672	513.6
f-50-300_T2700	300	2700	1893	0.8454	10,398	5870	32.2
f-50-300_T2900	300	2900	1895	0.3335	37,346	25,269	39.4
f-50-500_T1500	500	1500	434	0.0000	3669	2595	259,464
f-50-500_T2100	500	2100	1886	-8.4879	2101	1620	10,719
f-50-500_T2300	500	2300	1891	-0.1781	4329	2393	129.9
f-50-500_T2500	500	2500	1892	0.8704	3668	2337	44.4
f-50-500_T2700	500	2700	1895	0.3648	20,073	13,851	45.8
f-50-500_T2900	500	2900	1893	-0.3654	50,533	36,841	57.7
f-50-700_T1700	700	1700	1154	0.0000	21,910	18,635	1,863,499
f-50-700_T1900	700	1900	1812	-3243.7	9144	7130	36,217
f-50-700_T2100	700	2100	1874	-2.3792	1262	846.4	870.6
f-50-700_T2500	700	2500	1894	0.6081	7094	4298	34.2
f-50-700_T2700	700	2700	1893	0.9771	3345	2232	19.8
f-50-700_T2900	700	2900	1894	0.5956	27,086	19,125	33.4
f-50-900_T2100	900	2100	1876	-22.8344	4194	3503	59,620
f-50-900_T2500	900	2500	1884	-4.6826	23,126	13,496	90.7
f-50-900_T2900	900	2900	1893	0.6036	25,882	17,095	48.4
**Repeat: strat46**	**Case: B**	**Model: ANN**					
f-50-1_T1700	1	1700	357	0.0000	302.6	250.7	25,068
f-50-1_T1900	1	1900	1318	0.0000	5189	3371	337,094
f-50-1_T2100	1	2100	1816	-1.6034	525.8	464.0	1706
f-50-1_T2500	1	2500	1892	0.7851	4055	3513	75.0
f-50-1_T2700	1	2700	1893	0.9622	4211	2792	23.1
f-50-1_T2900	1	2900	1892	0.9726	6693	4362	12.9
f-50-100_T1500	100	1500	175	0.0000	298.6	250.9	25,091
f-50-100_T1700	100	1700	663	0.0000	593.4	464.3	46,432

continued on next page

Table S12 (continued)

Simulation	$n(O_2)$	T (K)	n_{rows}	R^2	RMSE (molecule·ps)	MAE (molecule·ps)	MAPE (%)
f-50-100_T1900	100	1900	1458	-31.0283	75.2	57.8	4367
f-50-100_T2100	100	2100	1827	-0.4413	1230	1101	680.5
f-50-100_T2500	100	2500	1891	0.9709	1828	1254	53.4
f-50-100_T2700	100	2700	1893	0.9645	4102	2674	20.9
f-50-100_T2900	100	2900	1893	0.9138	12,826	8516	23.5
f-50-300_T1500	300	1500	408	0.0000	1065	820.7	82,066
f-50-300_T1700	300	1700	1215	0.0000	1458	632.9	63,287
f-50-300_T1900	300	1900	1562	-1.72×10^7	2778	982.6	83,485
f-50-300_T2100	300	2100	1869	-547.4	11,441	6631	3274
f-50-300_T2300	300	2300	1889	0.1514	3036	2042	61.3
f-50-300_T2500	300	2500	1894	0.9362	2846	2172	347.6
f-50-300_T2700	300	2700	1893	0.9462	6134	4100	19.3
f-50-300_T2900	300	2900	1895	0.9221	12,767	9364	18.3
f-50-500_T1500	500	1500	434	0.0000	295.3	165.1	16,510
f-50-500_T2100	500	2100	1886	0.6797	386.0	283.1	394.9
f-50-500_T2300	500	2300	1891	0.1199	3741	1786	46.7
f-50-500_T2500	500	2500	1892	0.8917	3353	1796	28.1
f-50-500_T2700	500	2700	1895	0.9751	3974	3088	27.0
f-50-500_T2900	500	2900	1893	0.9846	5372	3927	11.7
f-50-700_T1700	700	1700	1154	0.0000	44,277	37,148	3,714,769
f-50-700_T1900	700	1900	1812	-27,376.9	26,563	20,680	73,437
f-50-700_T2100	700	2100	1874	-0.5826	863.6	549.4	801.5
f-50-700_T2500	700	2500	1894	0.9415	2742	1782	22.0
f-50-700_T2700	700	2700	1893	0.9790	3208	2212	15.4
f-50-700_T2900	700	2900	1894	0.9758	6626	4981	15.7
f-50-900_T2100	900	2100	1876	-0.9544	1201	757.0	9926
f-50-900_T2500	900	2500	1884	0.8772	3400	1789	37.2
f-50-900_T2900	900	2900	1893	0.9590	8323	6007	43.4
**Repeat: strat46 Case: B Model: SVR**							
f-50-1_T1700	1	1700	357	0.0000	457.2	343.7	34,374
f-50-1_T1900	1	1900	1318	0.0000	589.9	571.3	57,125
f-50-1_T2100	1	2100	1816	-178.8	4370	3013	5097
f-50-1_T2500	1	2500	1892	0.8719	3130	2728	144.8
f-50-1_T2700	1	2700	1893	0.3230	17,828	12,066	46.9
f-50-1_T2900	1	2900	1892	-0.2213	44,705	32,455	49.9
f-50-100_T1500	100	1500	175	0.0000	299.3	264.3	26,432

continued on next page

Table S12 (continued)

Simulation	$n(O_2)$	T (K)	n_{rows}	R^2	RMSE (molecule·ps)	MAE (molecule·ps)	MAPE (%)
f-50-100_T1700	100	1700	663	0.0000	199.4	121.5	12,149
f-50-100_T1900	100	1900	1458	-2605.8	678.1	400.1	17,341
f-50-100_T2100	100	2100	1827	-34.1032	6069	4310	687.9
f-50-100_T2500	100	2500	1891	0.9052	3301	2349	42.3
f-50-100_T2700	100	2700	1893	0.6800	12,324	6905	33.9
f-50-100_T2900	100	2900	1893	-0.5057	53,620	38,451	52.7
f-50-300_T1500	300	1500	408	0.0000	2968	2528	252,795
f-50-300_T1700	300	1700	1215	0.0000	1190	905.4	90,542
f-50-300_T1900	300	1900	1562	-5.19×10^6	1525	918.9	86,333
f-50-300_T2100	300	2100	1869	-441.0	10,271	7748	3946
f-50-300_T2300	300	2300	1889	-4.2428	7545	6168	212.5
f-50-300_T2500	300	2500	1894	0.6325	6833	4384	239.2
f-50-300_T2700	300	2700	1893	-0.0957	27,683	18,636	44.3
f-50-300_T2900	300	2900	1895	-0.5307	56,596	40,370	53.6
f-50-500_T1500	500	1500	434	0.0000	679.4	537.2	53,719
f-50-500_T2100	500	2100	1886	-76.3677	5999	4263	4802
f-50-500_T2300	500	2300	1891	-1.1641	5867	4677	356.0
f-50-500_T2500	500	2500	1892	0.8246	4267	2978	51.6
f-50-500_T2700	500	2700	1895	-0.0773	26,142	17,243	46.1
f-50-500_T2900	500	2900	1893	-0.6453	55,473	40,183	57.5
f-50-700_T1700	700	1700	1154	0.0000	14,693	13,860	1,385,980
f-50-700_T1900	700	1900	1812	-3362.2	9310	8770	89,513
f-50-700_T2100	700	2100	1874	-17.1949	2928	1974	4185
f-50-700_T2500	700	2500	1894	0.8514	4369	3024	38.2
f-50-700_T2700	700	2700	1893	0.2794	18,772	11,509	36.9
f-50-700_T2900	700	2900	1894	-0.3874	50,172	34,844	49.2
f-50-900_T2100	900	2100	1876	-13.7331	3297	3006	57,293
f-50-900_T2500	900	2500	1884	0.8235	4075	3096	207.2
f-50-900_T2900	900	2900	1893	-0.3756	48,218	32,775	98.2
**Repeat: strat46**	**Case: B**	**Model: GPR**					
f-50-1_T1700	1	1700	357	0.0000	388.9	260.5	26,049
f-50-1_T1900	1	1900	1318	0.0000	369.4	247.0	24,696
f-50-1_T2100	1	2100	1816	-78.6483	2908	1724	869.1
f-50-1_T2500	1	2500	1892	0.3204	7212	5241	54.7
f-50-1_T2700	1	2700	1893	0.9554	4576	3034	15.1
f-50-1_T2900	1	2900	1892	0.8832	13,823	10,292	19.5

continued on next page

Table S12 (continued)

Simulation	$n(O_2)$	T (K)	n_{rows}	R^2	RMSE (molecule·ps)	MAE (molecule·ps)	MAPE (%)
f-50-100_T1500	100	1500	175	0.0000	169.8	119.2	11,921
f-50-100_T1700	100	1700	663	0.0000	545.3	360.7	36,070
f-50-100_T1900	100	1900	1458	-26,526.6	2163	1296	61,376
f-50-100_T2100	100	2100	1827	-7.0930	2914	2120	291.8
f-50-100_T2500	100	2500	1891	0.9080	3252	1952	23.8
f-50-100_T2700	100	2700	1893	0.8176	9303	6136	19.8
f-50-100_T2900	100	2900	1893	0.9887	4637	3555	10.8
f-50-300_T1500	300	1500	408	0.0000	3573	2473	247,318
f-50-300_T1700	300	1700	1215	0.0000	3498	2389	238,851
f-50-300_T1900	300	1900	1562	-7.26×10^7	5706	4308	418,957
f-50-300_T2100	300	2100	1869	-405.6	9851	6457	2925
f-50-300_T2300	300	2300	1889	0.2584	2838	2124	75.5
f-50-300_T2500	300	2500	1894	0.9482	2566	1891	133.2
f-50-300_T2700	300	2700	1893	0.9472	6079	4115	13.9
f-50-300_T2900	300	2900	1895	0.9262	12,425	9477	18.2
f-50-500_T1500	500	1500	434	0.0000	373.8	269.8	26,982
f-50-500_T2100	500	2100	1886	-12.6533	2520	1747	1717
f-50-500_T2300	500	2300	1891	0.9179	1142	764.3	78.7
f-50-500_T2500	500	2500	1892	0.9405	2485	1408	17.3
f-50-500_T2700	500	2700	1895	0.9497	5651	3356	19.3
f-50-500_T2900	500	2900	1893	0.8659	15,838	9508	13.9
f-50-700_T1700	700	1700	1154	0.0000	30,492	26,249	2,624,864
f-50-700_T1900	700	1900	1812	-4517.8	10,791	9486	40,577
f-50-700_T2100	700	2100	1874	-3.2568	1416	919.2	635.2
f-50-700_T2500	700	2500	1894	0.9076	3445	2225	17.9
f-50-700_T2700	700	2700	1893	0.9753	3475	2384	11.6
f-50-700_T2900	700	2900	1894	0.9150	12,420	9524	18.4
f-50-900_T2100	900	2100	1876	-2.2196	1541	1257	25,295
f-50-900_T2500	900	2500	1884	-0.6284	12,380	7314	52.9
f-50-900_T2900	900	2900	1893	0.8441	16,234	11,949	42.5
**Repeat: strat46**	**Case: B**	**Model: persistence**					
f-50-1_T1700	1	1700	357	1.0000	0.0	0.0	0.0
f-50-1_T1900	1	1900	1318	1.0000	0.0	0.0	0.0
f-50-1_T2100	1	2100	1816	0.9631	62.6	54.9	23.7
f-50-1_T2500	1	2500	1892	0.9646	1647	1468	28.5
f-50-1_T2700	1	2700	1893	0.9644	4090	3642	27.3

continued on next page

Table S12 (continued)

Simulation	$n(O_2)$	T (K)	n_{rows}	R^2	RMSE (molecule·ps)	MAE (molecule·ps)	MAPE (%)
f-50-1_T2900	1	2900	1892	0.9666	7390	6812	25.5
f-50-100_T1500	100	1500	175	1.0000	0.0	0.0	0.0
f-50-100_T1700	100	1700	663	1.0000	0.0	0.0	0.0
f-50-100_T1900	100	1900	1458	0.5926	8.5	3.1	11.9
f-50-100_T2100	100	2100	1827	0.9585	208.7	176.9	28.0
f-50-100_T2500	100	2500	1891	0.9636	2046	1794	29.2
f-50-100_T2700	100	2700	1893	0.9633	4174	3673	26.9
f-50-100_T2900	100	2900	1893	0.9656	8105	7371	25.8
f-50-300_T1500	300	1500	408	1.0000	0.0	0.0	0.0
f-50-300_T1700	300	1700	1215	1.0000	0.0	0.0	0.0
f-50-300_T1900	300	1900	1562	0.0667	0.6	0.1	3.4
f-50-300_T2100	300	2100	1869	0.9635	93.4	80.0	24.3
f-50-300_T2300	300	2300	1889	0.9586	670.4	572.4	28.8
f-50-300_T2500	300	2500	1894	0.9609	2228	1892	31.4
f-50-300_T2700	300	2700	1893	0.9631	5080	4441	28.5
f-50-300_T2900	300	2900	1895	0.9655	8494	7665	26.3
f-50-500_T1500	500	1500	434	1.0000	0.0	0.0	0.0
f-50-500_T2100	500	2100	1886	0.9467	157.5	113.8	27.3
f-50-500_T2300	500	2300	1891	0.9589	808.1	663.3	31.3
f-50-500_T2500	500	2500	1892	0.9581	2085	1751	28.4
f-50-500_T2700	500	2700	1895	0.9622	4897	4216	29.2
f-50-500_T2900	500	2900	1893	0.9655	8037	7244	27.0
f-50-700_T1700	700	1700	1154	1.0000	0.0	0.0	0.0
f-50-700_T1900	700	1900	1812	0.9550	34.0	27.5	21.3
f-50-700_T2100	700	2100	1874	0.9562	143.7	115.1	27.1
f-50-700_T2500	700	2500	1894	0.9619	2213	1916	28.9
f-50-700_T2700	700	2700	1893	0.9621	4303	3713	28.5
f-50-700_T2900	700	2900	1894	0.9644	8040	7131	27.6
f-50-900_T2100	900	2100	1876	0.9632	164.8	133.9	23.0
f-50-900_T2500	900	2500	1884	0.9600	1940	1651	31.2
f-50-900_T2900	900	2900	1893	0.9640	7798	6850	28.8
**Repeat: strat46**	**Case: B**	**Model: physical_MLR**					
f-50-1_T1700	1	1700	357	0.0000	20,821	16,876	1,687,579
f-50-1_T1900	1	1900	1318	0.0000	14,858	12,621	1,262,053
f-50-1_T2100	1	2100	1816	-1844.0	13,998	11,869	135,795
f-50-1_T2500	1	2500	1892	-1.9443	15,010	13,381	434.5

continued on next page

Table S12 (continued)

Simulation	$n(O_2)$	T (K)	n_{rows}	R^2	RMSE (molecule·ps)	MAE (molecule·ps)	MAPE (%)
f-50-1_T2700	1	2700	1893	0.8125	9383	8531	242.4
f-50-1_T2900	1	2900	1892	0.4452	30,131	23,328	180.9
f-50-100_T1500	100	1500	175	0.0000	28,730	24,485	2,448,501
f-50-100_T1700	100	1700	663	0.0000	17,855	14,384	1,438,423
f-50-100_T1900	100	1900	1458	-1.23×10^6	14,742	12,479	977,990
f-50-100_T2100	100	2100	1827	-164.3	13,170	11,279	27,685
f-50-100_T2500	100	2500	1891	0.0758	10,308	9517	301.8
f-50-100_T2700	100	2700	1893	0.7308	11,302	10,322	283.9
f-50-100_T2900	100	2900	1893	0.4302	32,985	24,805	165.3
f-50-300_T1500	300	1500	408	0.0000	29,488	26,077	2,607,651
f-50-300_T1700	300	1700	1215	0.0000	19,241	15,171	1,517,095
f-50-300_T1900	300	1900	1562	-4.80×10^8	14,667	12,096	1,186,258
f-50-300_T2100	300	2100	1869	-982.9	15,325	13,138	278,504
f-50-300_T2300	300	2300	1889	-20.3800	15,237	12,824	4280
f-50-300_T2500	300	2500	1894	-0.0113	11,335	10,320	2013
f-50-300_T2700	300	2700	1893	0.7793	12,424	10,417	272.0
f-50-300_T2900	300	2900	1895	0.4166	34,941	26,454	182.5
f-50-500_T1500	500	1500	434	0.0000	27,270	23,414	2,341,387
f-50-500_T2100	500	2100	1886	-447.2	14,438	12,483	299,089
f-50-500_T2300	500	2300	1891	-12.3838	14,590	12,438	7545
f-50-500_T2500	500	2500	1892	-0.4857	12,418	11,148	315.8
f-50-500_T2700	500	2700	1895	0.7511	12,565	11,016	435.0
f-50-500_T2900	500	2900	1893	0.4150	33,077	25,445	256.3
f-50-700_T1700	700	1700	1154	0.0000	20,178	15,687	1,568,698
f-50-700_T1900	700	1900	1812	-12,140.6	17,689	14,532	683,827
f-50-700_T2100	700	2100	1874	-466.5	14,842	12,791	237,503
f-50-700_T2500	700	2500	1894	0.2203	10,007	9101	340.4
f-50-700_T2700	700	2700	1893	0.7959	9990	9130	290.4
f-50-700_T2900	700	2900	1894	0.5122	29,749	22,929	270.8
f-50-900_T2100	900	2100	1876	-287.8	14,598	12,499	545,043
f-50-900_T2500	900	2500	1884	-0.5807	12,197	11,200	586.3
f-50-900_T2900	900	2900	1893	0.5221	28,419	21,779	370.5
**Repeat: strat46 Case: B Model: physical_RF**							
f-50-1_T1700	1	1700	357	0.0000	66.4	16.1	1607
f-50-1_T1900	1	1900	1318	0.0000	273.8	192.1	19,213
f-50-1_T2100	1	2100	1816	-0.0842	339.3	256.1	80.1

continued on next page

Table S12 (continued)

Simulation	$n(O_2)$	T (K)	n_{rows}	R^2	RMSE (molecule·ps)	MAE (molecule·ps)	MAPE (%)
f-50-1_T2500	1	2500	1892	0.8657	3205	2973	385.2
f-50-1_T2700	1	2700	1893	0.7216	11,433	8409	94.1
f-50-1_T2900	1	2900	1892	0.7994	18,116	14,562	44.8
f-50-100_T1500	100	1500	175	0.0000	129.0	53.9	5392
f-50-100_T1700	100	1700	663	0.0000	60.4	25.6	2558
f-50-100_T1900	100	1900	1458	-3055.1	734.2	486.9	33,662
f-50-100_T2100	100	2100	1827	-0.6567	1318	985.1	88.6
f-50-100_T2500	100	2500	1891	0.9713	1816	1563	252.8
f-50-100_T2700	100	2700	1893	0.8249	9116	6435	24.4
f-50-100_T2900	100	2900	1893	0.8164	18,724	14,066	26.0
f-50-300_T1500	300	1500	408	0.0000	12.9	6.6	660.3
f-50-300_T1700	300	1700	1215	0.0000	143.7	81.3	8135
f-50-300_T1900	300	1900	1562	-905,124.8	636.9	500.9	48,282
f-50-300_T2100	300	2100	1869	-6.5928	1346	894.8	2258
f-50-300_T2300	300	2300	1889	-0.2633	3704	2601	202.5
f-50-300_T2500	300	2500	1894	0.9129	3327	2301	487.3
f-50-300_T2700	300	2700	1893	0.9705	4539	3450	26.4
f-50-300_T2900	300	2900	1895	0.9120	13,572	10,529	21.1
f-50-500_T1500	500	1500	434	0.0000	3962	3032	303,201
f-50-500_T2100	500	2100	1886	-18.9824	3049	2319	3006
f-50-500_T2300	500	2300	1891	0.7833	1856	1293	150.0
f-50-500_T2500	500	2500	1892	0.9366	2565	1819	25.0
f-50-500_T2700	500	2700	1895	0.9956	1680	925.5	19.6
f-50-500_T2900	500	2900	1893	0.9945	3200	2213	12.7
f-50-700_T1700	700	1700	1154	0.0000	956.3	768.6	76,864
f-50-700_T1900	700	1900	1812	-15.2842	647.8	463.4	810.8
f-50-700_T2100	700	2100	1874	0.6220	422.0	301.3	92.9
f-50-700_T2500	700	2500	1894	0.9862	1332	919.2	118.4
f-50-700_T2700	700	2700	1893	0.9672	4004	2331	32.6
f-50-700_T2900	700	2900	1894	0.8948	13,815	10,206	23.0
f-50-900_T2100	900	2100	1876	-65.9683	7030	5511	15,043
f-50-900_T2500	900	2500	1884	-0.5094	11,919	8202	236.4
f-50-900_T2900	900	2900	1893	0.7559	20,313	15,772	40.3

Table S13: Per-simulation test-set metrics of the 11 models on the 9 test simulations (final refit models: majority-vote hyperparameters of Table S11 and the final refit feature sets). Definitions and conventions as in Table S12.

Simulation	$n(O_2)$	T (K)	n_{rows}	R^2	RMSE (molecule·ps)	MAE (molecule·ps)	MAPE (%)
**Case: A Model: MLR**							
f-50-1_T1500	1	1500	87	0.0000	600.4	494.0	49,404
f-50-1_T2300	1	2300	1887	0.9833	477.3	400.2	120.6
f-50-100_T2300	100	2300	1889	0.9871	385.7	280.0	132.2
f-50-500_T1700	500	1700	1309	0.0000	711.0	601.4	60,141
f-50-500_T1900	500	1900	1791	-5.4674	337.7	294.2	9810
f-50-700_T2300	700	2300	1885	0.9049	910.7	795.4	50.7
f-50-900_T1900	900	1900	1838	-15.7373	572.4	554.3	26,165
f-50-900_T2300	900	2300	1882	0.8762	989.4	850.8	138.9
f-50-900_T2700	900	2700	1891	0.9997	379.8	318.9	13.0
**Case: A Model: DT**							
f-50-1_T1500	1	1500	87	0.0000	1.2	1.0	102.4
f-50-1_T2300	1	2300	1887	0.9974	188.2	149.7	22.6
f-50-100_T2300	100	2300	1889	0.9968	193.0	151.5	14.9
f-50-500_T1700	500	1700	1309	0.0000	0.9	0.9	90.0
f-50-500_T1900	500	1900	1791	0.9748	21.1	12.4	105.8
f-50-700_T2300	700	2300	1885	0.9967	169.3	117.3	20.3
f-50-900_T1900	900	1900	1838	0.8904	46.3	26.6	364.0
f-50-900_T2300	900	2300	1882	0.9951	197.1	123.4	28.4
f-50-900_T2700	900	2700	1891	0.9998	337.2	237.8	6.6
**Case: A Model: RF**							
f-50-1_T1500	1	1500	87	0.0000	2.8	1.6	164.2
f-50-1_T2300	1	2300	1887	0.9979	168.0	135.9	18.6
f-50-100_T2300	100	2300	1889	0.9975	170.7	142.6	13.3
f-50-500_T1700	500	1700	1309	0.0000	1.6	1.2	116.1
f-50-500_T1900	500	1900	1791	0.9786	19.4	11.8	136.8
f-50-700_T2300	700	2300	1885	0.9987	105.6	79.9	17.4
f-50-900_T1900	900	1900	1838	0.8991	44.4	26.8	266.2
f-50-900_T2300	900	2300	1882	0.9980	126.6	84.8	20.9
f-50-900_T2700	900	2700	1891	1.0000	163.7	129.3	4.7
**Case: A Model: XGBoost**							
f-50-1_T1500	1	1500	87	0.0000	3.9	1.8	183.4
f-50-1_T2300	1	2300	1887	0.9980	166.6	142.0	12.7
f-50-100_T2300	100	2300	1889	0.9969	188.2	147.7	7.8

continued on next page

Table S13 (continued)

Simulation	$n(O_2)$	T (K)	n_{rows}	R^2	RMSE (molecule·ps)	MAE (molecule·ps)	MAPE (%)
f-50-500_T1700	500	1700	1309	0.0000	1.1	0.8	81.0
f-50-500_T1900	500	1900	1791	0.9665	24.3	13.9	90.5
f-50-700_T2300	700	2300	1885	0.9972	155.0	130.7	15.6
f-50-900_T1900	900	1900	1838	0.9006	44.1	27.7	600.0
f-50-900_T2300	900	2300	1882	0.9978	132.0	109.5	22.5
f-50-900_T2700	900	2700	1891	0.9996	454.5	311.2	2.9
**Case: A Model: CatBoost**							
f-50-1_T1500	1	1500	87	0.0000	45.4	35.6	3557
f-50-1_T2300	1	2300	1887	0.9962	226.4	168.6	9.9
f-50-100_T2300	100	2300	1889	0.9954	231.2	174.3	10.2
f-50-500_T1700	500	1700	1309	0.0000	51.2	40.9	4087
f-50-500_T1900	500	1900	1791	0.7067	71.9	58.9	3234
f-50-700_T2300	700	2300	1885	0.9901	293.5	203.0	13.6
f-50-900_T1900	900	1900	1838	0.5108	97.9	76.6	4628
f-50-900_T2300	900	2300	1882	0.9901	279.8	180.9	19.8
f-50-900_T2700	900	2700	1891	0.9996	491.9	341.9	3.4
**Case: A Model: ANN**							
f-50-1_T1500	1	1500	87	0.0000	97.1	93.6	9357
f-50-1_T2300	1	2300	1887	0.9880	404.3	340.6	37.3
f-50-100_T2300	100	2300	1889	0.9814	462.9	408.4	37.3
f-50-500_T1700	500	1700	1309	0.0000	18.2	14.7	1465
f-50-500_T1900	500	1900	1791	0.3334	108.4	86.0	2498
f-50-700_T2300	700	2300	1885	0.9091	890.1	745.0	35.8
f-50-900_T1900	900	1900	1838	0.8143	60.3	48.4	2290
f-50-900_T2300	900	2300	1882	0.9265	762.4	678.1	118.5
f-50-900_T2700	900	2700	1891	0.9996	455.9	321.2	4.4
**Case: A Model: SVR**							
f-50-1_T1500	1	1500	87	0.0000	839.1	713.3	71,327
f-50-1_T2300	1	2300	1887	0.9757	575.1	514.3	101.3
f-50-100_T2300	100	2300	1889	0.6592	1983	1476	89.2
f-50-500_T1700	500	1700	1309	0.0000	373.6	249.7	24,965
f-50-500_T1900	500	1900	1791	-14.7814	527.6	390.1	18,021
f-50-700_T2300	700	2300	1885	-0.0301	2996	2296	187.6
f-50-900_T1900	900	1900	1838	-81.4425	1270	1145	60,467
f-50-900_T2300	900	2300	1882	-0.7232	3691	2798	1122
f-50-900_T2700	900	2700	1891	0.6533	13,974	8558	82.8

continued on next page

Table S13 (continued)

Simulation	$n(O_2)$	T (K)	n_{rows}	R^2	RMSE (molecule·ps)	MAE (molecule·ps)	MAPE (%)
**Case: A Model: GPR**							
f-50-1_T1500	1	1500	87	0.0000	11.9	6.7	672.8
f-50-1_T2300	1	2300	1887	0.9983	151.7	128.7	10.9
f-50-100_T2300	100	2300	1889	0.9989	112.5	88.3	6.2
f-50-500_T1700	500	1700	1309	0.0000	15.1	11.9	1192
f-50-500_T1900	500	1900	1791	0.8243	55.7	46.2	1258
f-50-700_T2300	700	2300	1885	0.9913	275.5	217.5	15.8
f-50-900_T1900	900	1900	1838	0.7480	70.2	60.3	1900
f-50-900_T2300	900	2300	1882	0.9939	220.1	160.2	12.8
f-50-900_T2700	900	2700	1891	1.0000	158.1	131.3	3.2
**Case: A Model: persistence**							
f-50-1_T1500	1	1500	87	1.0000	0.0	0.0	0.0
f-50-1_T2300	1	2300	1887	0.9633	707.0	626.9	25.9
f-50-100_T2300	100	2300	1889	0.9632	651.4	571.7	26.6
f-50-500_T1700	500	1700	1309	1.0000	0.0	0.0	0.0
f-50-500_T1900	500	1900	1791	0.9136	39.0	23.6	20.2
f-50-700_T2300	700	2300	1885	0.9553	624.5	512.3	27.5
f-50-900_T1900	900	1900	1838	0.9375	35.0	22.4	19.0
f-50-900_T2300	900	2300	1882	0.9521	615.2	494.1	31.1
f-50-900_T2700	900	2700	1891	0.9617	4647	3994	28.8
**Case: A Model: physical_MLR**							
f-50-1_T1500	1	1500	87	0.0000	29,398	25,410	2,540,958
f-50-1_T2300	1	2300	1887	-14.4086	14,482	12,164	1328
f-50-100_T2300	100	2300	1889	-18.1434	14,860	12,472	1609
f-50-500_T1700	500	1700	1309	0.0000	17,940	14,246	1,424,572
f-50-500_T1900	500	1900	1791	-13,293.0	15,313	12,930	900,569
f-50-700_T2300	700	2300	1885	-25.3302	15,149	12,776	2655
f-50-900_T1900	900	1900	1838	-12,818.8	15,842	13,305	964,568
f-50-900_T2300	900	2300	1882	-28.2190	15,199	12,815	7175
f-50-900_T2700	900	2700	1891	0.7948	10,752	9662	315.4
**Case: A Model: physical_RF**							
f-50-1_T1500	1	1500	87	0.0000	0.0	0.0	0.8
f-50-1_T2300	1	2300	1887	0.9537	794.2	637.4	18.1
f-50-100_T2300	100	2300	1889	0.9880	371.7	257.1	14.4
f-50-500_T1700	500	1700	1309	0.0000	0.0	0.0	1.4
f-50-500_T1900	500	1900	1791	-0.3882	156.5	83.3	51.2

continued on next page

Table S13 (continued)

Simulation	$n(O_2)$	T (K)	n_{rows}	R^2	RMSE (molecule·ps)	MAE (molecule·ps)	MAPE (%)
f-50-700_T2300	700	2300	1885	0.7776	1392	1002	28.9
f-50-900_T1900	900	1900	1838	0.5805	90.6	72.8	2456
f-50-900_T2300	900	2300	1882	0.6542	1654	1149	34.8
f-50-900_T2700	900	2700	1891	0.9902	2349	1642	7.2
**Case: B Model: MLR**							
f-50-1_T1500	1	1500	87	0.0000	17,206	15,532	1,553,170
f-50-1_T2300	1	2300	1887	0.4372	2768	2223	577.8
f-50-100_T2300	100	2300	1889	-8.8475	10,658	9911	683.5
f-50-500_T1700	500	1700	1309	0.0000	7726	6624	662,414
f-50-500_T1900	500	1900	1791	-10,446.4	13,575	11,281	397,073
f-50-700_T2300	700	2300	1885	-63.3561	23,685	21,769	5408
f-50-900_T1900	900	1900	1838	-15,872.9	17,628	14,927	1,161,874
f-50-900_T2300	900	2300	1882	-55.0969	21,060	19,483	18,597
f-50-900_T2700	900	2700	1891	0.0698	22,891	17,468	1150
**Case: B Model: DT**							
f-50-1_T1500	1	1500	87	1.0000	0.0	0.0	0.0
f-50-1_T2300	1	2300	1887	-20.6270	17,157	7026	114.5
f-50-100_T2300	100	2300	1889	-28.9593	18,589	5964	82.0
f-50-500_T1700	500	1700	1309	1.0000	0.0	0.0	0.0
f-50-500_T1900	500	1900	1791	0.3585	106.4	76.3	2584
f-50-700_T2300	700	2300	1885	-3.0323	5929	3261	75.3
f-50-900_T1900	900	1900	1838	-1.7366	231.5	149.7	2624
f-50-900_T2300	900	2300	1882	-1.0311	4007	2255	65.4
f-50-900_T2700	900	2700	1891	0.9173	6826	4339	17.5
**Case: B Model: RF**							
f-50-1_T1500	1	1500	87	0.0000	142.8	65.1	6513
f-50-1_T2300	1	2300	1887	0.3440	2988	2276	46.5
f-50-100_T2300	100	2300	1889	-11.1018	11,815	8260	132.8
f-50-500_T1700	500	1700	1309	0.0000	1385	892.4	89,236
f-50-500_T1900	500	1900	1791	-4153.2	8560	5689	32,223
f-50-700_T2300	700	2300	1885	-2.2911	5356	3702	89.0
f-50-900_T1900	900	1900	1838	-28.2401	756.6	451.5	12,195
f-50-900_T2300	900	2300	1882	-0.5349	3483	2139	68.7
f-50-900_T2700	900	2700	1891	0.9634	4541	2931	10.9
**Case: B Model: XGBoost**							
f-50-1_T1500	1	1500	87	0.0000	540.0	397.0	39,703

continued on next page

Table S13 (continued)

Simulation	$n(O_2)$	T (K)	n_{rows}	R^2	RMSE (molecule·ps)	MAE (molecule·ps)	MAPE (%)
f-50-1_T2300	1	2300	1887	-0.5912	4654	3720	116.8
f-50-100_T2300	100	2300	1889	-7.8616	10,110	7593	192.9
f-50-500_T1700	500	1700	1309	0.0000	1545	1098	109,761
f-50-500_T1900	500	1900	1791	-218.9	1970	1279	48,941
f-50-700_T2300	700	2300	1885	-0.0612	3041	2140	129.9
f-50-900_T1900	900	1900	1838	-405.7	2822	1929	35,498
f-50-900_T2300	900	2300	1882	-0.4728	3412	2186	156.4
f-50-900_T2700	900	2700	1891	0.9308	6243	4499	28.0
**Case: B Model: CatBoost**							
f-50-1_T1500	1	1500	87	0.0000	301.6	209.3	20,925
f-50-1_T2300	1	2300	1887	0.6003	2333	1805	57.2
f-50-100_T2300	100	2300	1889	-7.1866	9717	6442	134.5
f-50-500_T1700	500	1700	1309	0.0000	747.0	586.1	58,615
f-50-500_T1900	500	1900	1791	-148.2	1622	937.6	20,392
f-50-700_T2300	700	2300	1885	0.3360	2406	1765	91.9
f-50-900_T1900	900	1900	1838	-31.2406	794.5	520.7	9723
f-50-900_T2300	900	2300	1882	0.2730	2397	1510	104.9
f-50-900_T2700	900	2700	1891	0.8360	9611	5903	23.5
**Case: B Model: ANN**							
f-50-1_T1500	1	1500	87	0.0000	182.2	153.6	15,358
f-50-1_T2300	1	2300	1887	0.8367	1491	1153	43.7
f-50-100_T2300	100	2300	1889	0.7626	1655	1134	45.2
f-50-500_T1700	500	1700	1309	0.0000	284.9	223.1	22,309
f-50-500_T1900	500	1900	1791	-11.3541	466.8	296.6	5099
f-50-700_T2300	700	2300	1885	0.5144	2057	1572	59.5
f-50-900_T1900	900	1900	1838	0.5991	88.6	68.3	4372
f-50-900_T2300	900	2300	1882	0.8145	1211	938.3	57.0
f-50-900_T2700	900	2700	1891	0.9537	5105	3730	22.0
**Case: B Model: SVR**							
f-50-1_T1500	1	1500	87	0.0000	304.1	232.8	23,275
f-50-1_T2300	1	2300	1887	-0.4680	4470	3451	109.3
f-50-100_T2300	100	2300	1889	-2.7675	6592	5329	134.6
f-50-500_T1700	500	1700	1309	0.0000	281.7	190.5	19,049
f-50-500_T1900	500	1900	1791	-248.2	2096	1240	6971
f-50-700_T2300	700	2300	1885	-3.0622	5951	4353	181.6
f-50-900_T1900	900	1900	1838	-34.8306	837.5	820.0	44,461

continued on next page

Table S13 (continued)

Simulation	$n(O_2)$	T (K)	n_{rows}	R^2	RMSE (molecule·ps)	MAE (molecule·ps)	MAPE (%)
f-50-900_T2300	900	2300	1882	-1.1685	4141	2880	602.8
f-50-900_T2700	900	2700	1891	0.3212	19,554	11,866	63.8
**Case: B Model: GPR**							
f-50-1_T1500	1	1500	87	0.0000	132.1	70.2	7021
f-50-1_T2300	1	2300	1887	0.8173	1577	1281	59.2
f-50-100_T2300	100	2300	1889	0.0782	3261	2427	65.2
f-50-500_T1700	500	1700	1309	0.0000	370.3	265.1	26,507
f-50-500_T1900	500	1900	1791	-203.3	1898	1021	17,275
f-50-700_T2300	700	2300	1885	-0.3892	3480	2521	80.8
f-50-900_T1900	900	1900	1838	-65.2737	1139	899.0	19,591
f-50-900_T2300	900	2300	1882	0.8608	1049	712.2	57.9
f-50-900_T2700	900	2700	1891	0.9794	3404	2405	13.6
**Case: B Model: persistence**							
f-50-1_T1500	1	1500	87	1.0000	0.0	0.0	0.0
f-50-1_T2300	1	2300	1887	0.9633	707.0	626.9	25.9
f-50-100_T2300	100	2300	1889	0.9632	651.4	571.7	26.6
f-50-500_T1700	500	1700	1309	1.0000	0.0	0.0	0.0
f-50-500_T1900	500	1900	1791	0.9136	39.0	23.6	20.2
f-50-700_T2300	700	2300	1885	0.9553	624.5	512.3	27.5
f-50-900_T1900	900	1900	1838	0.9375	35.0	22.4	19.0
f-50-900_T2300	900	2300	1882	0.9521	615.2	494.1	31.1
f-50-900_T2700	900	2700	1891	0.9617	4647	3994	28.8
**Case: B Model: physical_MLR**							
f-50-1_T1500	1	1500	87	0.0000	29,398	25,410	2,540,958
f-50-1_T2300	1	2300	1887	-14.4086	14,482	12,164	1328
f-50-100_T2300	100	2300	1889	-18.1434	14,860	12,472	1609
f-50-500_T1700	500	1700	1309	0.0000	17,940	14,246	1,424,572
f-50-500_T1900	500	1900	1791	-13,293.0	15,313	12,930	900,569
f-50-700_T2300	700	2300	1885	-25.3302	15,149	12,776	2655
f-50-900_T1900	900	1900	1838	-12,818.8	15,842	13,305	964,568
f-50-900_T2300	900	2300	1882	-28.2190	15,199	12,815	7175
f-50-900_T2700	900	2700	1891	0.7948	10,752	9662	315.4
**Case: B Model: physical_RF**							
f-50-1_T1500	1	1500	87	0.0000	49.0	18.0	1795
f-50-1_T2300	1	2300	1887	0.7047	2005	1509	73.3
f-50-100_T2300	100	2300	1889	0.6517	2004	1548	56.6

continued on next page

Table S13 (continued)

Simulation	$n(O_2)$	T (K)	n_{rows}	R^2	RMSE (molecule·ps)	MAE (molecule·ps)	MAPE (%)
f-50-500_T1700	500	1700	1309	0.0000	101.8	65.6	6555
f-50-500_T1900	500	1900	1791	-8.2556	404.0	283.7	3629
f-50-700_T2300	700	2300	1885	0.3983	2290	1488	43.0
f-50-900_T1900	900	1900	1838	-45.4988	954.1	702.2	9800
f-50-900_T2300	900	2300	1882	-1.8848	4776	3113	175.5
f-50-900_T2700	900	2700	1891	0.7827	11,065	7313	90.8

S8. SHAP settings

The SHAP analyses of the main text use the following settings. The explained model per case is the model with the highest first-pass out-of-fold R^2 among the eight machine-learning models; if that model is not tree-based, the best tree model is substituted (Case A: GPR $\rightarrow$ RF; Case B: ANN $\rightarrow$ XGBoost). Main-effect attributions are computed with `TreeExplainer` in interventional mode, with a background set of 100 training rows drawn uniformly at random (seed 42). Interaction values are computed with `tree_path_dependent` (SHAP 0.49.1 does not provide interventional interactions) on a random test subsample of 2000 rows (seed 42). The interaction heatmaps are normalized by the largest off-diagonal mean absolute interaction value, and the diagonal is set to 1. Because XGBoost ≥ 3 encodes the model's base score in a way that differs from what SHAP 0.49.1 expects, the pipeline monkey-patches SHAP's UBJSON decoder to restore the scalar base score; after the patch, the additivity check passes with a maximum error of 2.4×10^{-7}. An analogous tree-model substitution for CatBoost is coded in the pipeline but was never triggered.

Figures S4 and S5 show the interaction heatmaps of the Case A random forest and the Case B XGBoost model, respectively.

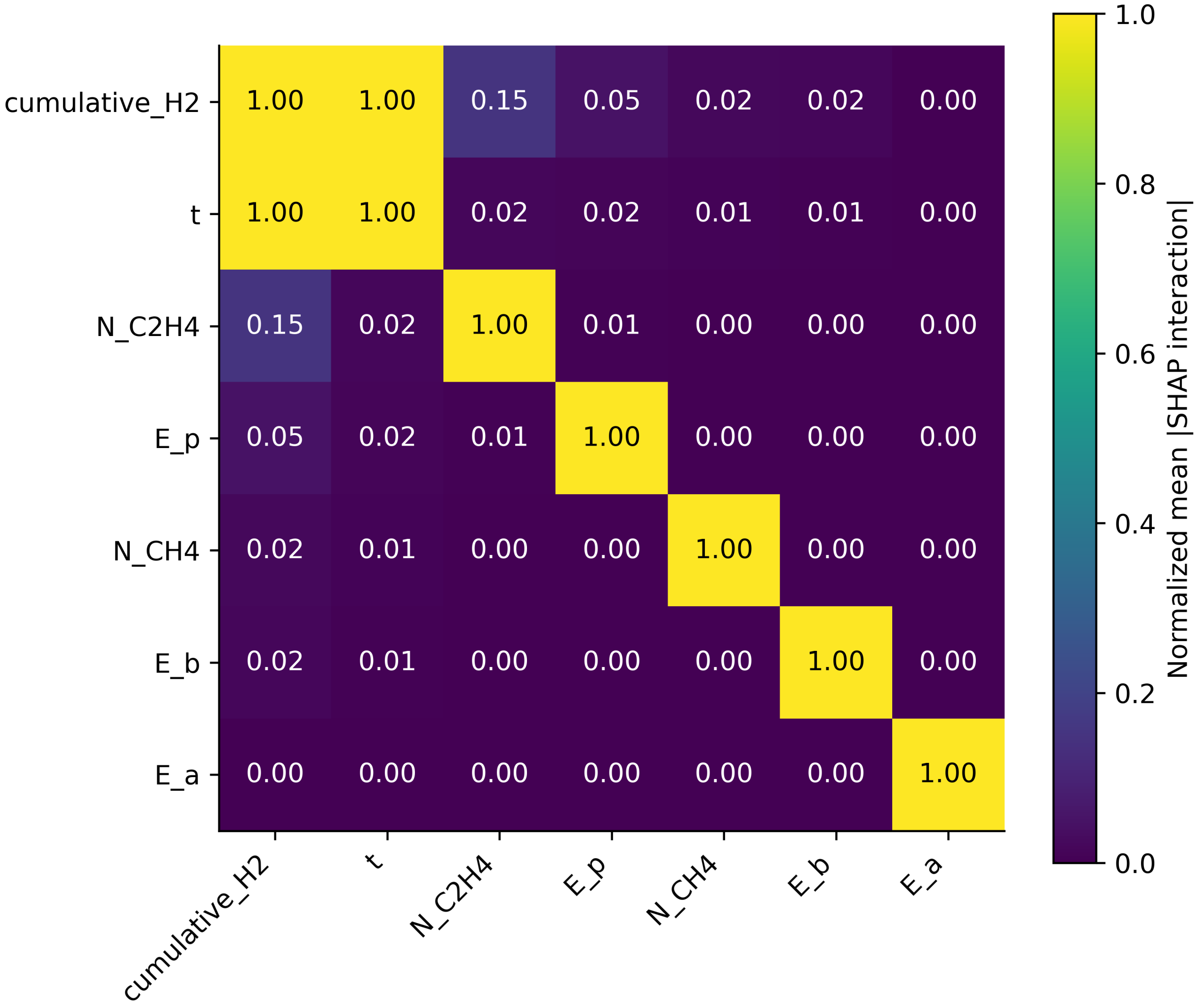

Figure S4: Normalised SHAP interaction heatmap for the Case A random forest: every cell is the mean pairwise |SHAP| interaction divided by the largest off-diagonal mean interaction, so the strongest pair, (`cumulative_H2`, t), reads 1.000; the diagonal (main effects) is set to 1.0 by the normalisation.

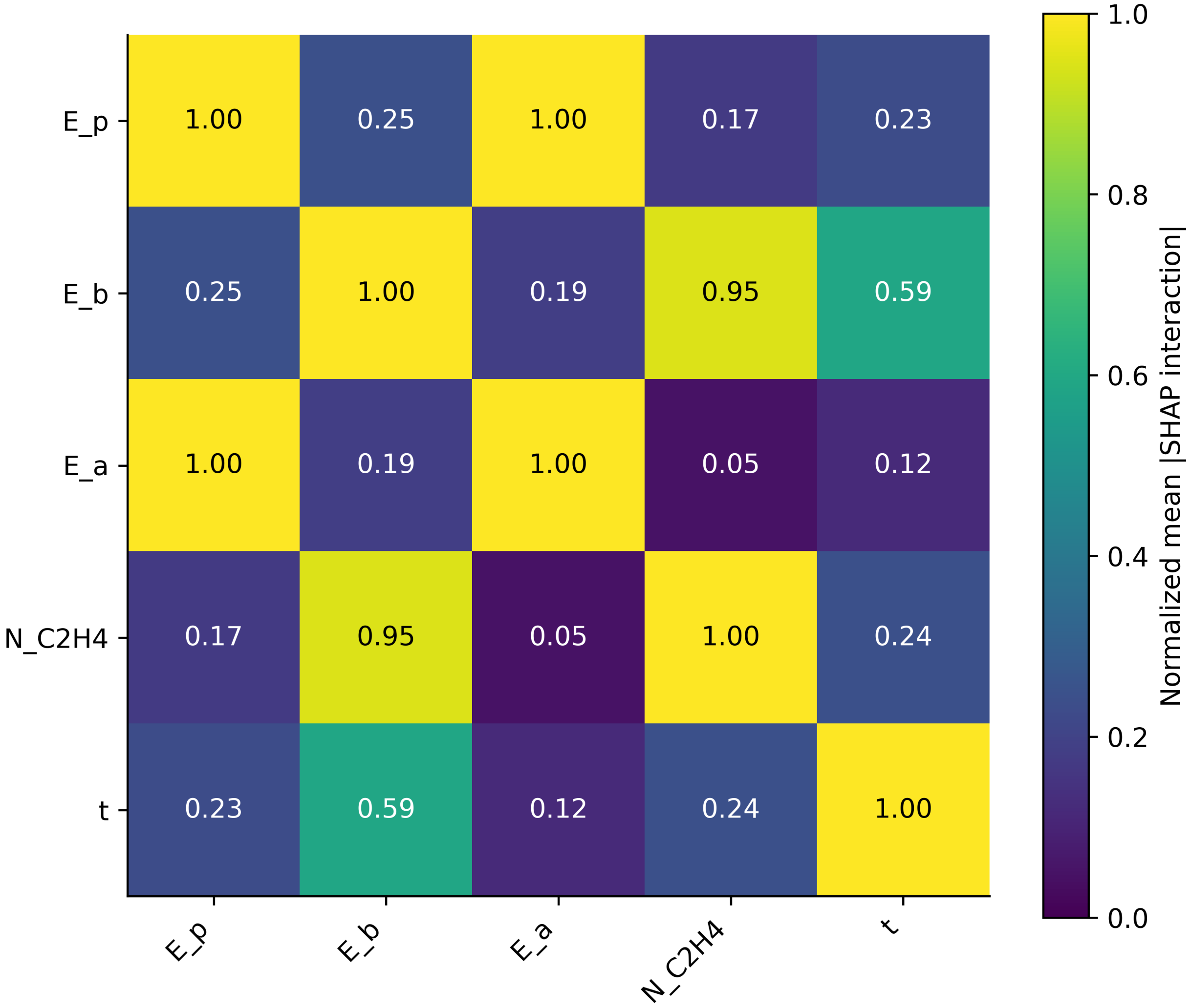

Figure S5: Normalised SHAP interaction heatmap for the Case B XGBoost model, in the format of Figure S4: the strongest off-diagonal interaction is (E_{p}, E_{a}) (normalised 1.000), and the strongest diagonal main effect is E_{p}.

The SHAP dependence plots of the top features of each case, discussed in Section 3.5 of the main text, are collected here as Figures S6 and S7.

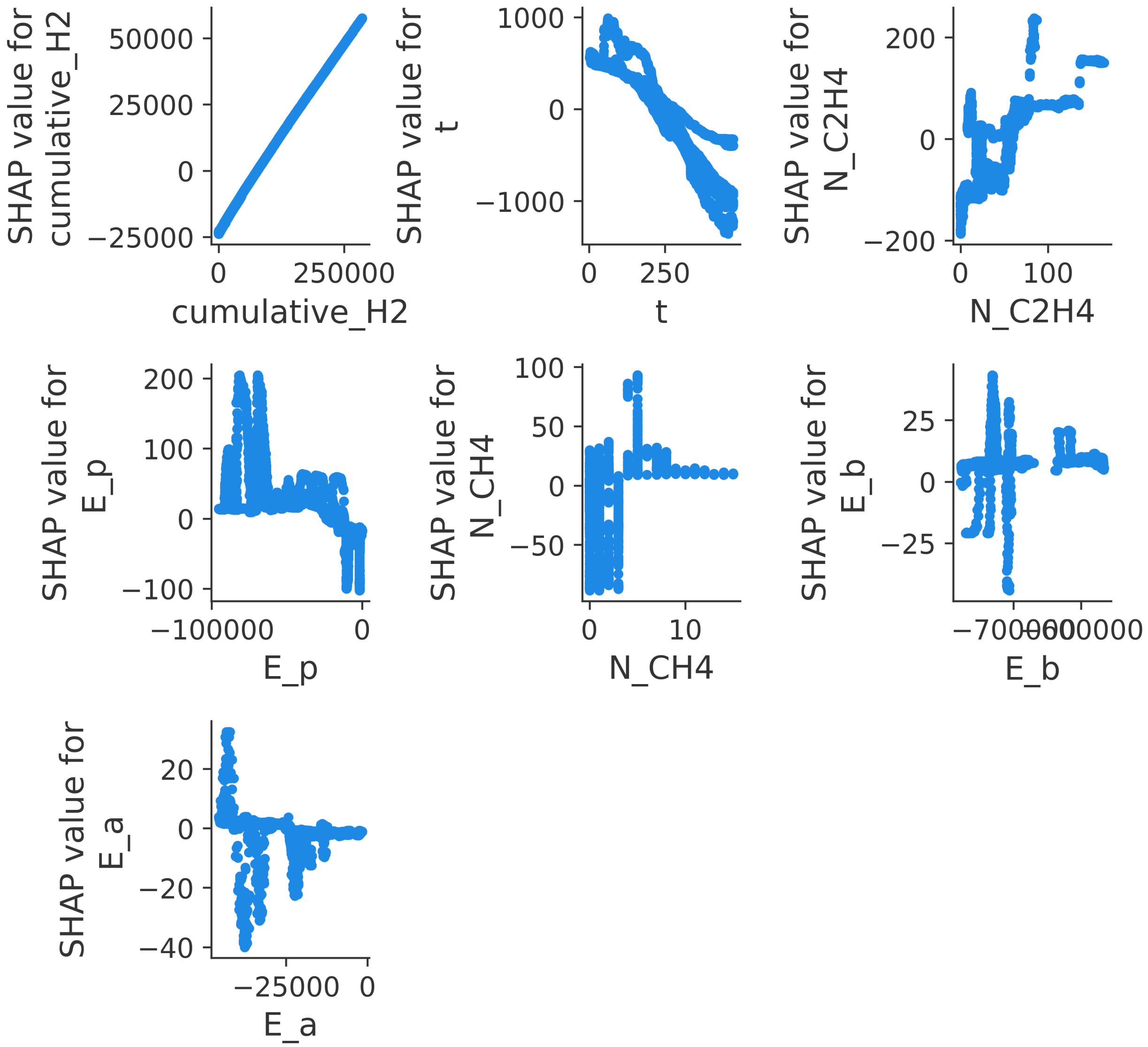

Figure S6: SHAP dependence plots for the top Case A features (`cumulative_H2`, t, $N_{C_2H_4}$, and the remaining final-refit features), same explainer as the Case A beeswarm summary of Section 3.5 of the main text: the SHAP value of `cumulative_H2` increases near-monotonically with the feature value.

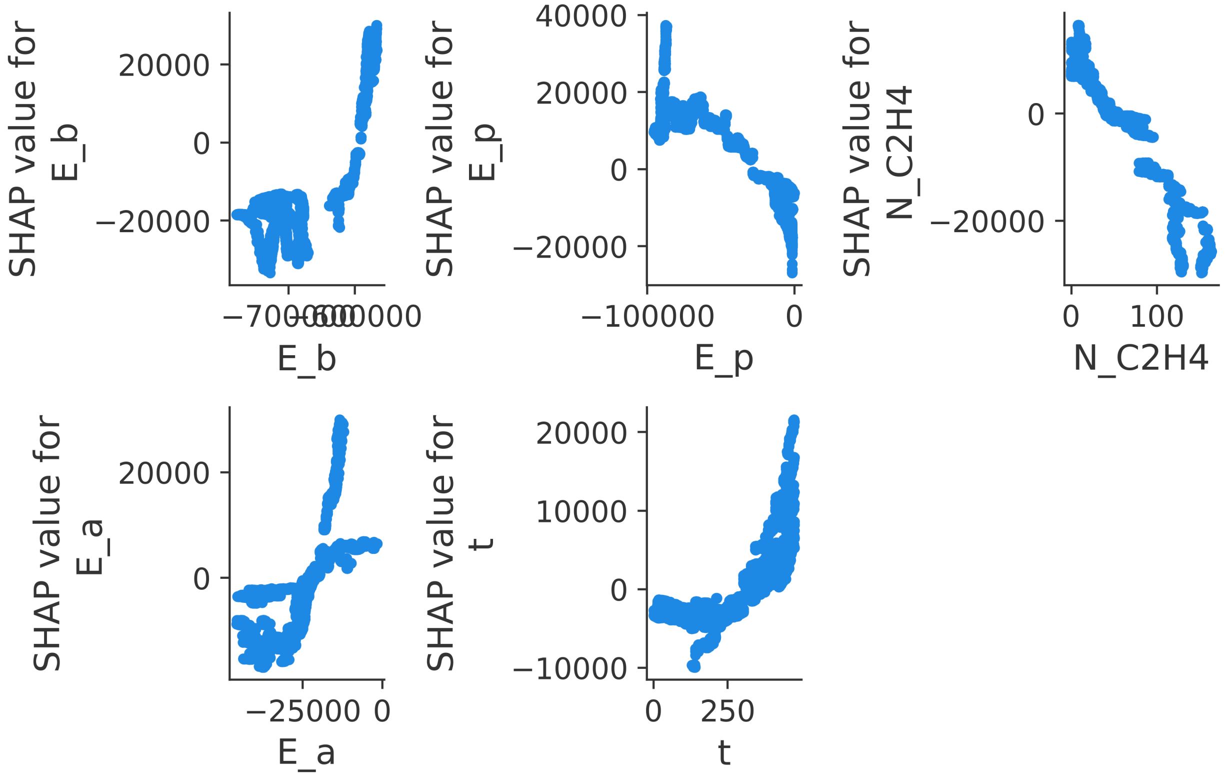

Figure S7: SHAP dependence plots for the top Case B features (E_b, E_p, $N_{C_2H_4}$, and the remaining final-refit features), same explainer as the Case B beeswarm summary of Section 3.5 of the main text; the transitions are steeper and more threshold-like than in Case A (Figure S6).

S9. Software versions

All simulations and analyses were performed with the following software versions:

- Python 3.11.14
- scikit-learn 1.8.0
- NumPy 1.26.4
- pandas 3.0.2
- XGBoost 3.1.1

- CatBoost 1.2.10
- SHAP 0.49.1
- matplotlib 3.8.0
- LAMMPS 23 June 2022 (binary `lmp_intel_cpu_intelmpi`, Intel MPI; Section S1)
- Packmol 20.14.0
